# Genomic basis for RNA alterations in cancer

**DOI:** 10.1038/s41586-020-1970-0

**Published:** 2020-02-05

**Authors:** PCAWG Transcriptome Core Group, Claudia Calabrese, Natalie R. Davidson, Deniz Demircioğlu, Nuno A. Fonseca, Yao He, André Kahles, Kjong-Van Lehmann, Fenglin Liu, Yuichi Shiraishi, Cameron M. Soulette, Lara Urban, Claudia Calabrese, Natalie R. Davidson, Deniz Demircioğlu, Nuno A. Fonseca, Yao He, André Kahles, Kjong-Van Lehmann, Fenglin Liu, Yuichi Shiraishi, Cameron M. Soulette, Lara Urban, Liliana Greger, Siliang Li, Dongbing Liu, Marc D. Perry, Qian Xiang, Fan Zhang, Junjun Zhang, Peter Bailey, Serap Erkek, Katherine A. Hoadley, Yong Hou, Matthew R. Huska, Helena Kilpinen, Jan O. Korbel, Maximillian G. Marin, Julia Markowski, Tannistha Nandi, Qiang Pan-Hammarström, Chandra Sekhar Pedamallu, Reiner Siebert, Stefan G. Stark, Hong Su, Patrick Tan, Sebastian M. Waszak, Christina Yung, Shida Zhu, Philip Awadalla, Chad J. Creighton, Matthew Meyerson, B. F. Francis Ouellette, Kui Wu, Huanming Yang, PCAWG Transcriptome Working Group, Nuno A. Fonseca, André Kahles, Kjong-Van Lehmann, Lara Urban, Cameron M. Soulette, Yuichi Shiraishi, Fenglin Liu, Yao He, Deniz Demircioğlu, Natalie R. Davidson, Claudia Calabrese, Junjun Zhang, Marc D. Perry, Qian Xiang, Liliana Greger, Siliang Li, Dongbing Liu, Stefan G. Stark, Fan Zhang, Samirkumar B. Amin, Peter Bailey, Aurélien Chateigner, Isidro Cortés-Ciriano, Brian Craft, Serap Erkek, Milana Frenkel-Morgenstern, Mary Goldman, Katherine A. Hoadley, Yong Hou, Matthew R. Huska, Ekta Khurana, Helena Kilpinen, Jan O. Korbel, Fabien C. Lamaze, Chang Li, Xiaobo Li, Xinyue Li, Xingmin Liu, Maximillian G. Marin, Julia Markowski, Tannistha Nandi, Morten M. Nielsen, Akinyemi I. Ojesina, Qiang Pan-Hammarström, Peter J. Park, Chandra Sekhar Pedamallu, Jakob S. Pedersen, Reiner Siebert, Hong Su, Patrick Tan, Bin Tean Teh, Jian Wang, Sebastian M. Waszak, Heng Xiong, Sergei Yakneen, Chen Ye, Christina Yung, Xiuqing Zhang, Liangtao Zheng, Jingchun Zhu, Shida Zhu, Philip Awadalla, Chad J. Creighton, Matthew Meyerson, B. F. Francis Ouellette, Kui Wu, Huanming Yang, Jonathan Göke, Roland F. Schwarz, Oliver Stegle, Zemin Zhang, Alvis Brazma, Gunnar Rätsch, Angela N. Brooks, Alvis Brazma, Angela N. Brooks, Jonathan Göke, Gunnar Rätsch, Roland F. Schwarz, Oliver Stegle, Zemin Zhang, PCAWG Consortium, Lauri A. Aaltonen, Federico Abascal, Adam Abeshouse, Hiroyuki Aburatani, David J. Adams, Nishant Agrawal, Keun Soo Ahn, Sung-Min Ahn, Hiroshi Aikata, Rehan Akbani, Kadir C. Akdemir, Hikmat Al-Ahmadie, Sultan T. Al-Sedairy, Fatima Al-Shahrour, Malik Alawi, Monique Albert, Kenneth Aldape, Ludmil B. Alexandrov, Adrian Ally, Kathryn Alsop, Eva G. Alvarez, Fernanda Amary, Samirkumar B. Amin, Brice Aminou, Ole Ammerpohl, Matthew J. Anderson, Yeng Ang, Davide Antonello, Pavana Anur, Samuel Aparicio, Elizabeth L. Appelbaum, Yasuhito Arai, Axel Aretz, Koji Arihiro, Shun-ichi Ariizumi, Joshua Armenia, Laurent Arnould, Sylvia Asa, Yassen Assenov, Gurnit Atwal, Sietse Aukema, J. Todd Auman, Miriam R. R. Aure, Philip Awadalla, Marta Aymerich, Gary D. Bader, Adrian Baez-Ortega, Matthew H. Bailey, Peter J. Bailey, Miruna Balasundaram, Saianand Balu, Pratiti Bandopadhayay, Rosamonde E. Banks, Stefano Barbi, Andrew P. Barbour, Jonathan Barenboim, Jill Barnholtz-Sloan, Hugh Barr, Elisabet Barrera, John Bartlett, Javier Bartolome, Claudio Bassi, Oliver F. Bathe, Daniel Baumhoer, Prashant Bavi, Stephen B. Baylin, Wojciech Bazant, Duncan Beardsmore, Timothy A. Beck, Sam Behjati, Andreas Behren, Beifang Niu, Cindy Bell, Sergi Beltran, Christopher Benz, Andrew Berchuck, Anke K. Bergmann, Erik N. Bergstrom, Benjamin P. Berman, Daniel M. Berney, Stephan H. Bernhart, Rameen Beroukhim, Mario Berrios, Samantha Bersani, Johanna Bertl, Miguel Betancourt, Vinayak Bhandari, Shriram G. Bhosle, Andrew V. Biankin, Matthias Bieg, Darell Bigner, Hans Binder, Ewan Birney, Michael Birrer, Nidhan K. Biswas, Bodil Bjerkehagen, Tom Bodenheimer, Lori Boice, Giada Bonizzato, Johann S. De Bono, Arnoud Boot, Moiz S. Bootwalla, Ake Borg, Arndt Borkhardt, Keith A. Boroevich, Ivan Borozan, Christoph Borst, Marcus Bosenberg, Mattia Bosio, Jacqueline Boultwood, Guillaume Bourque, Paul C. Boutros, G. Steven Bova, David T. Bowen, Reanne Bowlby, David D. L. Bowtell, Sandrine Boyault, Rich Boyce, Jeffrey Boyd, Alvis Brazma, Paul Brennan, Daniel S. Brewer, Arie B. Brinkman, Robert G. Bristow, Russell R. Broaddus, Jane E. Brock, Malcolm Brock, Annegien Broeks, Angela N. Brooks, Denise Brooks, Benedikt Brors, Søren Brunak, Timothy J. C. Bruxner, Alicia L. Bruzos, Alex Buchanan, Ivo Buchhalter, Christiane Buchholz, Susan Bullman, Hazel Burke, Birgit Burkhardt, Kathleen H. Burns, John Busanovich, Carlos D. Bustamante, Adam P. Butler, Atul J. Butte, Niall J. Byrne, Anne-Lise Børresen-Dale, Samantha J. Caesar-Johnson, Andy Cafferkey, Declan Cahill, Claudia Calabrese, Carlos Caldas, Fabien Calvo, Niedzica Camacho, Peter J. Campbell, Elias Campo, Cinzia Cantù, Shaolong Cao, Thomas E. Carey, Joana Carlevaro-Fita, Rebecca Carlsen, Ivana Cataldo, Mario Cazzola, Jonathan Cebon, Robert Cerfolio, Dianne E. Chadwick, Dimple Chakravarty, Don Chalmers, Calvin Wing Yiu Chan, Kin Chan, Michelle Chan-Seng-Yue, Vishal S. Chandan, David K. Chang, Stephen J. Chanock, Lorraine A. Chantrill, Aurélien Chateigner, Nilanjan Chatterjee, Kazuaki Chayama, Hsiao-Wei Chen, Jieming Chen, Ken Chen, Yiwen Chen, Zhaohong Chen, Andrew D. Cherniack, Jeremy Chien, Yoke-Eng Chiew, Suet-Feung Chin, Juok Cho, Sunghoon Cho, Jung Kyoon Choi, Wan Choi, Christine Chomienne, Zechen Chong, Su Pin Choo, Angela Chou, Angelika N. Christ, Elizabeth L. Christie, Eric Chuah, Carrie Cibulskis, Kristian Cibulskis, Sara Cingarlini, Peter Clapham, Alexander Claviez, Sean Cleary, Nicole Cloonan, Marek Cmero, Colin C. Collins, Ashton A. Connor, Susanna L. Cooke, Colin S. Cooper, Leslie Cope, Vincenzo Corbo, Matthew G. Cordes, Stephen M. Cordner, Isidro Cortés-Ciriano, Kyle Covington, Prue A. Cowin, Brian Craft, David Craft, Chad J. Creighton, Yupeng Cun, Erin Curley, Ioana Cutcutache, Karolina Czajka, Bogdan Czerniak, Rebecca A. Dagg, Ludmila Danilova, Maria Vittoria Davi, Natalie R. Davidson, Helen Davies, Ian J. Davis, Brandi N. Davis-Dusenbery, Kevin J. Dawson, Francisco M. De La Vega, Ricardo De Paoli-Iseppi, Timothy Defreitas, Angelo P. Dei Tos, Olivier Delaneau, John A. Demchok, Jonas Demeulemeester, German M. Demidov, Deniz Demircioğlu, Nening M. Dennis, Robert E. Denroche, Stefan C. Dentro, Nikita Desai, Vikram Deshpande, Amit G. Deshwar, Christine Desmedt, Jordi Deu-Pons, Noreen Dhalla, Neesha C. Dhani, Priyanka Dhingra, Rajiv Dhir, Anthony DiBiase, Klev Diamanti, Li Ding, Shuai Ding, Huy Q. Dinh, Luc Dirix, HarshaVardhan Doddapaneni, Nilgun Donmez, Michelle T. Dow, Ronny Drapkin, Oliver Drechsel, Ruben M. Drews, Serge Serge, Tim Dudderidge, Ana Dueso-Barroso, Andrew J. Dunford, Michael Dunn, Lewis Jonathan Dursi, Fraser R. Duthie, Ken Dutton-Regester, Jenna Eagles, Douglas F. Easton, Stuart Edmonds, Paul A. Edwards, Sandra E. Edwards, Rosalind A. Eeles, Anna Ehinger, Juergen Eils, Roland Eils, Adel El-Naggar, Matthew Eldridge, Kyle Ellrott, Serap Erkek, Georgia Escaramis, Shadrielle M. G. Espiritu, Xavier Estivill, Dariush Etemadmoghadam, Jorunn E. Eyfjord, Bishoy M. Faltas, Daiming Fan, Yu Fan, William C. Faquin, Claudiu Farcas, Matteo Fassan, Aquila Fatima, Francesco Favero, Nodirjon Fayzullaev, Ina Felau, Sian Fereday, Martin L. Ferguson, Vincent Ferretti, Lars Feuerbach, Matthew A. Field, J. Lynn Fink, Gaetano Finocchiaro, Cyril Fisher, Matthew W. Fittall, Anna Fitzgerald, Rebecca C. Fitzgerald, Adrienne M. Flanagan, Neil E. Fleshner, Paul Flicek, John A. Foekens, Kwun M. Fong, Nuno A. Fonseca, Christopher S. Foster, Natalie S. Fox, Michael Fraser, Scott Frazer, Milana Frenkel-Morgenstern, William Friedman, Joan Frigola, Catrina C. Fronick, Akihiro Fujimoto, Masashi Fujita, Masashi Fukayama, Lucinda A. Fulton, Robert S. Fulton, Mayuko Furuta, P. Andrew Futreal, Anja Füllgrabe, Stacey B. Gabriel, Steven Gallinger, Carlo Gambacorti-Passerini, Jianjiong Gao, Shengjie Gao, Levi Garraway, Øystein Garred, Erik Garrison, Dale W. Garsed, Nils Gehlenborg, Josep L. L. Gelpi, Joshy George, Daniela S. Gerhard, Clarissa Gerhauser, Jeffrey E. Gershenwald, Mark Gerstein, Moritz Gerstung, Gad Getz, Mohammed Ghori, Ronald Ghossein, Nasra H. Giama, Richard A. Gibbs, Bob Gibson, Anthony J. Gill, Pelvender Gill, Dilip D. Giri, Dominik Glodzik, Vincent J. Gnanapragasam, Maria Elisabeth Goebler, Mary J. Goldman, Carmen Gomez, Santiago Gonzalez, Abel Gonzalez-Perez, Dmitry A. Gordenin, James Gossage, Kunihito Gotoh, Ramaswamy Govindan, Dorthe Grabau, Janet S. Graham, Robert C. Grant, Anthony R. Green, Eric Green, Liliana Greger, Nicola Grehan, Sonia Grimaldi, Sean M. Grimmond, Robert L. Grossman, Adam Grundhoff, Gunes Gundem, Qianyun Guo, Manaswi Gupta, Shailja Gupta, Ivo G. Gut, Marta Gut, Jonathan Göke, Gavin Ha, Andrea Haake, David Haan, Siegfried Haas, Kerstin Haase, James E. Haber, Nina Habermann, Faraz Hach, Syed Haider, Natsuko Hama, Freddie C. Hamdy, Anne Hamilton, Mark P. Hamilton, Leng Han, George B. Hanna, Martin Hansmann, Nicholas J. Haradhvala, Olivier Harismendy, Ivon Harliwong, Arif O. Harmanci, Eoghan Harrington, Takanori Hasegawa, David Haussler, Steve Hawkins, Shinya Hayami, Shuto Hayashi, D. Neil Hayes, Stephen J. Hayes, Nicholas K. Hayward, Steven Hazell, Yao He, Allison P. Heath, Simon C. Heath, David Hedley, Apurva M. Hegde, David I. Heiman, Michael C. Heinold, Zachary Heins, Lawrence E. Heisler, Eva Hellstrom-Lindberg, Mohamed Helmy, Seong Gu Heo, Austin J. Hepperla, José María Heredia-Genestar, Carl Herrmann, Peter Hersey, Julian M. Hess, Holmfridur Hilmarsdottir, Jonathan Hinton, Satoshi Hirano, Nobuyoshi Hiraoka, Katherine A. Hoadley, Asger Hobolth, Ermin Hodzic, Jessica I. Hoell, Steve Hoffmann, Oliver Hofmann, Andrea Holbrook, Aliaksei Z. Holik, Michael A. Hollingsworth, Oliver Holmes, Robert A. Holt, Chen Hong, Eun Pyo Hong, Jongwhi H. Hong, Gerrit K. Hooijer, Henrik Hornshøj, Fumie Hosoda, Yong Hou, Volker Hovestadt, William Howat, Alan P. Hoyle, Ralph H. Hruban, Jianhong Hu, Taobo Hu, Xing Hua, Kuan-lin Huang, Mei Huang, Mi Ni Huang, Vincent Huang, Yi Huang, Wolfgang Huber, Thomas J. Hudson, Michael Hummel, Jillian A. Hung, David Huntsman, Ted R. Hupp, Jason Huse, Matthew R. Huska, Barbara Hutter, Carolyn M. Hutter, Daniel Hübschmann, Christine A. Iacobuzio-Donahue, Charles David Imbusch, Marcin Imielinski, Seiya Imoto, William B. Isaacs, Keren Isaev, Shumpei Ishikawa, Murat Iskar, S. M. Ashiqul Islam, Michael Ittmann, Sinisa Ivkovic, Jose M. G. Izarzugaza, Jocelyne Jacquemier, Valerie Jakrot, Nigel B. Jamieson, Gun Ho Jang, Se Jin Jang, Joy C. Jayaseelan, Reyka Jayasinghe, Stuart R. Jefferys, Karine Jegalian, Jennifer L. Jennings, Seung-Hyup Jeon, Lara Jerman, Yuan Ji, Wei Jiao, Peter A. Johansson, Amber L. Johns, Jeremy Johns, Rory Johnson, Todd A. Johnson, Clemency Jolly, Yann Joly, Jon G. Jonasson, Corbin D. Jones, David R. Jones, David T. W. Jones, Nic Jones, Steven J. M. Jones, Jos Jonkers, Young Seok Ju, Hartmut Juhl, Jongsun Jung, Malene Juul, Randi Istrup Juul, Sissel Juul, Natalie Jäger, Rolf Kabbe, Andre Kahles, Abdullah Kahraman, Vera B. Kaiser, Hojabr Kakavand, Sangeetha Kalimuthu, Christof von Kalle, Koo Jeong Kang, Katalin Karaszi, Beth Karlan, Rosa Karlić, Dennis Karsch, Katayoon Kasaian, Karin S. Kassahn, Hitoshi Katai, Mamoru Kato, Hiroto Katoh, Yoshiiku Kawakami, Jonathan D. Kay, Stephen H. Kazakoff, Marat D. Kazanov, Maria Keays, Electron Kebebew, Richard F. Kefford, Manolis Kellis, James G. Kench, Catherine J. Kennedy, Jules N. A. Kerssemakers, David Khoo, Vincent Khoo, Narong Khuntikeo, Ekta Khurana, Helena Kilpinen, Hark Kyun Kim, Hyung-Lae Kim, Hyung-Yong Kim, Hyunghwan Kim, Jaegil Kim, Jihoon Kim, Jong K. Kim, Youngwook Kim, Tari A. King, Wolfram Klapper, Kortine Kleinheinz, Leszek J. Klimczak, Stian Knappskog, Michael Kneba, Bartha M. Knoppers, Youngil Koh, Jan Komorowski, Daisuke Komura, Mitsuhiro Komura, Gu Kong, Marcel Kool, Jan O. Korbel, Viktoriya Korchina, Andrey Korshunov, Michael Koscher, Roelof Koster, Zsofia Kote-Jarai, Antonios Koures, Milena Kovacevic, Barbara Kremeyer, Helene Kretzmer, Markus Kreuz, Savitri Krishnamurthy, Dieter Kube, Kiran Kumar, Pardeep Kumar, Sushant Kumar, Yogesh Kumar, Ritika Kundra, Kirsten Kübler, Ralf Küppers, Jesper Lagergren, Phillip H. Lai, Peter W. Laird, Sunil R. Lakhani, Christopher M. Lalansingh, Emilie Lalonde, Fabien C. Lamaze, Adam Lambert, Eric Lander, Pablo Landgraf, Luca Landoni, Anita Langerød, Andrés Lanzós, Denis Larsimont, Erik Larsson, Mark Lathrop, Loretta M. S. Lau, Chris Lawerenz, Rita T. Lawlor, Michael S. Lawrence, Alexander J. Lazar, Ana Mijalkovic Lazic, Xuan Le, Darlene Lee, Donghoon Lee, Eunjung Alice Lee, Hee Jin Lee, Jake June-Koo Lee, Jeong-Yeon Lee, Juhee Lee, Ming Ta Michael Lee, Henry Lee-Six, Kjong-Van Lehmann, Hans Lehrach, Dido Lenze, Conrad R. Leonard, Daniel A. Leongamornlert, Ignaty Leshchiner, Louis Letourneau, Ivica Letunic, Douglas A. Levine, Lora Lewis, Tim Ley, Chang Li, Constance H. Li, Haiyan Irene Li, Jun Li, Lin Li, Shantao Li, Siliang Li, Xiaobo Li, Xiaotong Li, Xinyue Li, Yilong Li, Han Liang, Sheng-Ben Liang, Peter Lichter, Pei Lin, Ziao Lin, W. M. Linehan, Ole Christian Lingjærde, Dongbing Liu, Eric Minwei Liu, Fei-Fei Fei Liu, Fenglin Liu, Jia Liu, Xingmin Liu, Julie Livingstone, Dimitri Livitz, Naomi Livni, Lucas Lochovsky, Markus Loeffler, Georgina V. Long, Armando Lopez-Guillermo, Shaoke Lou, David N. Louis, Laurence B. Lovat, Yiling Lu, Yong-Jie Lu, Youyong Lu, Claudio Luchini, Ilinca Lungu, Xuemei Luo, Hayley J. Luxton, Andy G. Lynch, Lisa Lype, Cristina López, Carlos López-Otín, Eric Z. Ma, Yussanne Ma, Gaetan MacGrogan, Shona MacRae, Geoff Macintyre, Tobias Madsen, Kazuhiro Maejima, Andrea Mafficini, Dennis T. Maglinte, Arindam Maitra, Partha P. Majumder, Luca Malcovati, Salem Malikic, Giuseppe Malleo, Graham J. Mann, Luisa Mantovani-Löffler, Kathleen Marchal, Giovanni Marchegiani, Elaine R. Mardis, Adam A. Margolin, Maximillian G. Marin, Florian Markowetz, Julia Markowski, Jeffrey Marks, Tomas Marques-Bonet, Marco A. Marra, Luke Marsden, John W. M. Martens, Sancha Martin, Jose I. Martin-Subero, Iñigo Martincorena, Alexander Martinez-Fundichely, Yosef E. Maruvka, R. Jay Mashl, Charlie E. Massie, Thomas J. Matthew, Lucy Matthews, Erik Mayer, Simon Mayes, Michael Mayo, Faridah Mbabaali, Karen McCune, Ultan McDermott, Patrick D. McGillivray, Michael D. McLellan, John D. McPherson, John R. McPherson, Treasa A. McPherson, Samuel R. Meier, Alice Meng, Shaowu Meng, Andrew Menzies, Neil D. Merrett, Sue Merson, Matthew Meyerson, William Meyerson, Piotr A. Mieczkowski, George L. Mihaiescu, Sanja Mijalkovic, Tom Mikkelsen, Michele Milella, Linda Mileshkin, Christopher A. Miller, David K. Miller, Jessica K. Miller, Gordon B. Mills, Ana Milovanovic, Sarah Minner, Marco Miotto, Gisela Mir Arnau, Lisa Mirabello, Chris Mitchell, Thomas J. Mitchell, Satoru Miyano, Naoki Miyoshi, Shinichi Mizuno, Fruzsina Molnár-Gábor, Malcolm J. Moore, Richard A. Moore, Sandro Morganella, Quaid D. Morris, Carl Morrison, Lisle E. Mose, Catherine D. Moser, Ferran Muiños, Loris Mularoni, Andrew J. Mungall, Karen Mungall, Elizabeth A. Musgrove, Ville Mustonen, David Mutch, Francesc Muyas, Donna M. Muzny, Alfonso Muñoz, Jerome Myers, Ola Myklebost, Peter Möller, Genta Nagae, Adnan M. Nagrial, Hardeep K. Nahal-Bose, Hitoshi Nakagama, Hidewaki Nakagawa, Hiromi Nakamura, Toru Nakamura, Kaoru Nakano, Tannistha Nandi, Jyoti Nangalia, Mia Nastic, Arcadi Navarro, Fabio C. P. Navarro, David E. Neal, Gerd Nettekoven, Felicity Newell, Steven J. Newhouse, Yulia Newton, Alvin Wei Tian Ng, Anthony Ng, Jonathan Nicholson, David Nicol, Yongzhan Nie, G. Petur Nielsen, Morten Muhlig Nielsen, Serena Nik-Zainal, Michael S. Noble, Katia Nones, Paul A. Northcott, Faiyaz Notta, Brian D. O’Connor, Peter O’Donnell, Maria O’Donovan, Sarah O’Meara, Brian Patrick O’Neill, J. Robert O’Neill, David Ocana, Angelica Ochoa, Layla Oesper, Christopher Ogden, Hideki Ohdan, Kazuhiro Ohi, Lucila Ohno-Machado, Karin A. Oien, Akinyemi I. Ojesina, Hidenori Ojima, Takuji Okusaka, Larsson Omberg, Choon Kiat Ong, Stephan Ossowski, German Ott, B. F. Francis Ouellette, Christine P’ng, Marta Paczkowska, Salvatore Paiella, Chawalit Pairojkul, Marina Pajic, Qiang Pan-Hammarström, Elli Papaemmanuil, Irene Papatheodorou, Nagarajan Paramasivam, Ji Wan Park, Joong-Won Park, Keunchil Park, Kiejung Park, Peter J. Park, Joel S. Parker, Simon L. Parsons, Harvey Pass, Danielle Pasternack, Alessandro Pastore, Ann-Marie Patch, Iris Pauporté, Antonio Pea, John V. Pearson, Chandra Sekhar Pedamallu, Jakob Skou Pedersen, Paolo Pederzoli, Martin Peifer, Nathan A. Pennell, Charles M. Perou, Marc D. Perry, Gloria M. Petersen, Myron Peto, Nicholas Petrelli, Robert Petryszak, Stefan M. Pfister, Mark Phillips, Oriol Pich, Hilda A. Pickett, Todd D. Pihl, Nischalan Pillay, Sarah Pinder, Mark Pinese, Andreia V. Pinho, Esa Pitkänen, Xavier Pivot, Elena Piñeiro-Yáñez, Laura Planko, Christoph Plass, Paz Polak, Tirso Pons, Irinel Popescu, Olga Potapova, Aparna Prasad, Shaun R. Preston, Manuel Prinz, Antonia L. Pritchard, Stephenie D. Prokopec, Elena Provenzano, Xose S. Puente, Sonia Puig, Montserrat Puiggròs, Sergio Pulido-Tamayo, Gulietta M. Pupo, Colin A. Purdie, Michael C. Quinn, Raquel Rabionet, Janet S. Rader, Bernhard Radlwimmer, Petar Radovic, Benjamin Raeder, Keiran M. Raine, Manasa Ramakrishna, Kamna Ramakrishnan, Suresh Ramalingam, Benjamin J. Raphael, W. Kimryn Rathmell, Tobias Rausch, Guido Reifenberger, Jüri Reimand, Jorge Reis-Filho, Victor Reuter, Iker Reyes-Salazar, Matthew A. Reyna, Sheila M. Reynolds, Esther Rheinbay, Yasser Riazalhosseini, Andrea L. Richardson, Julia Richter, Matthew Ringel, Markus Ringnér, Yasushi Rino, Karsten Rippe, Jeffrey Roach, Lewis R. Roberts, Nicola D. Roberts, Steven A. Roberts, A. Gordon Robertson, Alan J. Robertson, Javier Bartolomé Rodriguez, Bernardo Rodriguez-Martin, F. Germán Rodríguez-González, Michael H. A. Roehrl, Marius Rohde, Hirofumi Rokutan, Gilles Romieu, Ilse Rooman, Tom Roques, Daniel Rosebrock, Mara Rosenberg, Philip C. Rosenstiel, Andreas Rosenwald, Edward W. Rowe, Romina Royo, Steven G. Rozen, Yulia Rubanova, Mark A. Rubin, Carlota Rubio-Perez, Vasilisa A. Rudneva, Borislav C. Rusev, Andrea Ruzzenente, Gunnar Rätsch, Radhakrishnan Sabarinathan, Veronica Y. Sabelnykova, Sara Sadeghi, S. Cenk Sahinalp, Natalie Saini, Mihoko Saito-Adachi, Gordon Saksena, Adriana Salcedo, Roberto Salgado, Leonidas Salichos, Richard Sallari, Charles Saller, Roberto Salvia, Michelle Sam, Jaswinder S. Samra, Francisco Sanchez-Vega, Chris Sander, Grant Sanders, Rajiv Sarin, Iman Sarrafi, Aya Sasaki-Oku, Torill Sauer, Guido Sauter, Robyn P. M. Saw, Maria Scardoni, Christopher J. Scarlett, Aldo Scarpa, Ghislaine Scelo, Dirk Schadendorf, Jacqueline E. Schein, Markus B. Schilhabel, Matthias Schlesner, Thorsten Schlomm, Heather K. Schmidt, Sarah-Jane Schramm, Stefan Schreiber, Nikolaus Schultz, Steven E. Schumacher, Roland F. Schwarz, Richard A. Scolyer, David Scott, Ralph Scully, Raja Seethala, Ayellet V. Segre, Iris Selander, Colin A. Semple, Yasin Senbabaoglu, Subhajit Sengupta, Elisabetta Sereni, Stefano Serra, Dennis C. Sgroi, Mark Shackleton, Nimish C. Shah, Sagedeh Shahabi, Catherine A. Shang, Ping Shang, Ofer Shapira, Troy Shelton, Ciyue Shen, Hui Shen, Rebecca Shepherd, Ruian Shi, Yan Shi, Yu-Jia Shiah, Tatsuhiro Shibata, Juliann Shih, Eigo Shimizu, Kiyo Shimizu, Seung Jun Shin, Yuichi Shiraishi, Tal Shmaya, Ilya Shmulevich, Solomon I. Shorser, Charles Short, Raunak Shrestha, Suyash S. Shringarpure, Craig Shriver, Shimin Shuai, Nikos Sidiropoulos, Reiner Siebert, Anieta M. Sieuwerts, Lina Sieverling, Sabina Signoretti, Katarzyna O. Sikora, Michele Simbolo, Ronald Simon, Janae V. Simons, Jared T. Simpson, Peter T. Simpson, Samuel Singer, Nasa Sinnott-Armstrong, Payal Sipahimalani, Tara J. Skelly, Marcel Smid, Jaclyn Smith, Karen Smith-McCune, Nicholas D. Socci, Heidi J. Sofia, Matthew G. Soloway, Lei Song, Anil K. Sood, Sharmila Sothi, Christos Sotiriou, Cameron M. Soulette, Paul N. Span, Paul T. Spellman, Nicola Sperandio, Andrew J. Spillane, Oliver Spiro, Jonathan Spring, Johan Staaf, Peter F. Stadler, Peter Staib, Stefan G. Stark, Lucy Stebbings, Ólafur Andri Stefánsson, Oliver Stegle, Lincoln D. Stein, Alasdair Stenhouse, Chip Stewart, Stephan Stilgenbauer, Miranda D. Stobbe, Michael R. Stratton, Jonathan R. Stretch, Adam J. Struck, Joshua M. Stuart, Henk G. Stunnenberg, Hong Su, Xiaoping Su, Ren X. Sun, Stephanie Sungalee, Hana Susak, Akihiro Suzuki, Fred Sweep, Monika Szczepanowski, Holger Sültmann, Takashi Yugawa, Angela Tam, David Tamborero, Benita Kiat Tee Tan, Donghui Tan, Patrick Tan, Hiroko Tanaka, Hirokazu Taniguchi, Tomas J. Tanskanen, Maxime Tarabichi, Roy Tarnuzzer, Patrick Tarpey, Morgan L. Taschuk, Kenji Tatsuno, Simon Tavaré, Darrin F. Taylor, Amaro Taylor-Weiner, Jon W. Teague, Bin Tean Teh, Varsha Tembe, Javier Temes, Kevin Thai, Sarah P. Thayer, Nina Thiessen, Gilles Thomas, Sarah Thomas, Alan Thompson, Alastair M. Thompson, John F. F. Thompson, R. Houston Thompson, Heather Thorne, Leigh B. Thorne, Adrian Thorogood, Grace Tiao, Nebojsa Tijanic, Lee E. Timms, Roberto Tirabosco, Marta Tojo, Stefania Tommasi, Christopher W. Toon, Umut H. Toprak, David Torrents, Giampaolo Tortora, Jörg Tost, Yasushi Totoki, David Townend, Nadia Traficante, Isabelle Treilleux, Jean-Rémi Trotta, Lorenz H. P. Trümper, Ming Tsao, Tatsuhiko Tsunoda, Jose M. C. Tubio, Olga Tucker, Richard Turkington, Daniel J. Turner, Andrew Tutt, Masaki Ueno, Naoto T. Ueno, Christopher Umbricht, Husen M. Umer, Timothy J. Underwood, Lara Urban, Tomoko Urushidate, Tetsuo Ushiku, Liis Uusküla-Reimand, Alfonso Valencia, David J. Van Den Berg, Steven Van Laere, Peter Van Loo, Erwin G. Van Meir, Gert G. Van den Eynden, Theodorus Van der Kwast, Naveen Vasudev, Miguel Vazquez, Ravikiran Vedururu, Umadevi Veluvolu, Shankar Vembu, Lieven P. C. Verbeke, Peter Vermeulen, Clare Verrill, Alain Viari, David Vicente, Caterina Vicentini, K. VijayRaghavan, Juris Viksna, Ricardo E. Vilain, Izar Villasante, Anne Vincent-Salomon, Tapio Visakorpi, Douglas Voet, Paresh Vyas, Ignacio Vázquez-García, Nick M. Waddell, Nicola Waddell, Claes Wadelius, Lina Wadi, Rabea Wagener, Jeremiah A. Wala, Jian Wang, Jiayin Wang, Linghua Wang, Qi Wang, Wenyi Wang, Yumeng Wang, Zhining Wang, Paul M. Waring, Hans-Jörg Warnatz, Jonathan Warrell, Anne Y. Warren, Sebastian M. Waszak, David C. Wedge, Dieter Weichenhan, Paul Weinberger, John N. Weinstein, Joachim Weischenfeldt, Daniel J. Weisenberger, Ian Welch, Michael C. Wendl, Johannes Werner, Justin P. Whalley, David A. Wheeler, Hayley C. Whitaker, Dennis Wigle, Matthew D. Wilkerson, Ashley Williams, James S. Wilmott, Gavin W. Wilson, Julie M. Wilson, Richard K. Wilson, Boris Winterhoff, Jeffrey A. Wintersinger, Maciej Wiznerowicz, Stephan Wolf, Bernice H. Wong, Tina Wong, Winghing Wong, Youngchoon Woo, Scott Wood, Bradly G. Wouters, Adam J. Wright, Derek W. Wright, Mark H. Wright, Chin-Lee Wu, Dai-Ying Wu, Guanming Wu, Jianmin Wu, Kui Wu, Yang Wu, Zhenggang Wu, Liu Xi, Tian Xia, Qian Xiang, Xiao Xiao, Rui Xing, Heng Xiong, Qinying Xu, Yanxun Xu, Hong Xue, Shinichi Yachida, Sergei Yakneen, Rui Yamaguchi, Takafumi N. Yamaguchi, Masakazu Yamamoto, Shogo Yamamoto, Hiroki Yamaue, Fan Yang, Huanming Yang, Jean Y. Yang, Liming Yang, Lixing Yang, Shanlin Yang, Tsun-Po Yang, Yang Yang, Xiaotong Yao, Marie-Laure Yaspo, Lucy Yates, Christina Yau, Chen Ye, Kai Ye, Venkata D. Yellapantula, Christopher J. Yoon, Sung-Soo Yoon, Fouad Yousif, Jun Yu, Kaixian Yu, Willie Yu, Yingyan Yu, Ke Yuan, Yuan Yuan, Denis Yuen, Christina K. Yung, Olga Zaikova, Jorge Zamora, Marc Zapatka, Jean C. Zenklusen, Thorsten Zenz, Nikolajs Zeps, Cheng-Zhong Zhang, Fan Zhang, Hailei Zhang, Hongwei Zhang, Hongxin Zhang, Jiashan Zhang, Jing Zhang, Junjun Zhang, Xiuqing Zhang, Xuanping Zhang, Yan Zhang, Zemin Zhang, Zhongming Zhao, Liangtao Zheng, Xiuqing Zheng, Wanding Zhou, Yong Zhou, Bin Zhu, Hongtu Zhu, Jingchun Zhu, Shida Zhu, Lihua Zou, Xueqing Zou, Anna deFazio, Nicholas van As, Carolien H. M. van Deurzen, Marc J. van de Vijver, L. van’t Veer, Christian von Mering

**Affiliations:** 2 European Molecular Biology Laboratory, European Bioinformatics Institute, Hinxton, UK; 3 ETH Zurich, Zurich, Switzerland; 4 Memorial Sloan Kettering Cancer Center, New York, NY USA; 5 Weill Cornell Medical College, New York, NY USA; 6 SIB Swiss Institute of Bioinformatics, Lausanne, Switzerland; 7 University Hospital Zurich, Zurich, Switzerland; 8 National University of Singapore, Singapore, Singapore; 9 Genome Institute of Singapore, Singapore, Singapore; 10 Peking University, Beijing, China; 11 The University of Tokyo, Minato-ku, Japan; 12 University of California, Santa Cruz, Santa Cruz, CA USA; 13 BGI-Shenzhen, Shenzhen, China; 14 China National GeneBank-Shenzhen, Shenzhen, China; 15 Ontario Institute for Cancer Research, Toronto, Ontario, Canada; 16 University of California, San Francisco, San Francisco, CA USA; 17 University of Glasgow, Glasgow, UK; 18 European Molecular Biology Laboratory, Genome Biology Unit, Heidelberg, Germany; 19 The University of North Carolina at Chapel Hill, Chapel Hill, NC USA; 20 Berlin Institute for Medical Systems Biology, Max Delbruck Center for Molecular Medicine, Berlin, Germany; 21 University College London, London, UK; 22 Karolinska Institutet, Stockholm, Sweden; 23 Broad Institute, Cambridge, MA USA; 24 Ulm University and Ulm University Medical Center, Ulm, Germany; 25 Duke-NUS Medical School, Singapore, Singapore; 26 University of Toronto, Toronto, Ontario Canada; 27 Baylor College of Medicine, Houston, TX USA; 28 Dana-Farber Cancer Institute, Boston, MA USA; 29 Harvard Medical School, Boston, MA USA; 30 University of Toronto, Toronto, Ontario, Canada; 31 National Cancer Centre Singapore, Singapore, Singapore; 32 German Cancer Consortium (DKTK), partner site Berlin, Germany; 33 German Cancer Research Center (DKFZ), Heidelberg, Germany; 37 The UT MD Anderson Cancer Center, Houston, TX USA; 38 BioForA, French National Insitute for Agriculture, Food, and Environment (INRAE), ONF, Orléans, France; 39 Ludwig Center at Harvard, Boston, MA USA; 40 University of Cambridge, Cambridge, UK; 41 The Azrieli Faculty of Medicine, Bar-Ilan University, Safed, Israel; 42 Aarhus University, Aarhus, Denmark; 43 HudsonAlpha Institute for Biotechnology, Huntsville, AL USA; 44 University of Alabama at Birmingham, Birmingham, AL USA; 200 Applied Tumor Genomics Research Program, Research Programs Unit, University of Helsinki, Helsinki, Finland; 201 Wellcome Sanger Institute, Wellcome Genome Campus, Hinxton, UK; 202 Memorial Sloan Kettering Cancer Center, New York, NY USA; 203 Genome Science Division, Research Center for Advanced Science and Technology, University of Tokyo, Tokyo, Japan; 204 Department of Surgery, University of Chicago, Chicago, IL USA; 205 Department of Surgery, Division of Hepatobiliary and Pancreatic Surgery, School of Medicine, Keimyung University Dongsan Medical Center, Daegu, South Korea; 206 Department of Oncology, Gil Medical Center, Gachon University, Incheon, South Korea; 207 Hiroshima University, Hiroshima, Japan; 208 Department of Bioinformatics and Computational Biology, The University of Texas MD Anderson Cancer Center, Houston, TX USA; 209 University of Texas MD Anderson Cancer Center, Houston, TX USA; 210 King Faisal Specialist Hospital and Research Centre, Al Maather, Riyadh, Saudi Arabia; 211 Bioinformatics Unit, Spanish National Cancer Research Centre (CNIO), Madrid, Spain; 212 Bioinformatics Core Facility, University Medical Center Hamburg, Hamburg, Germany; 213 Heinrich Pette Institute, Leibniz Institute for Experimental Virology, Hamburg, Germany; 214 Ontario Tumour Bank, Ontario Institute for Cancer Research, Toronto, ON Canada; 215 Department of Pathology, The University of Texas MD Anderson Cancer Center, Houston, TX USA; 216 Laboratory of Pathology, Center for Cancer Research, National Cancer Institute, Bethesda, MD USA; 217 Department of Cellular and Molecular Medicine and Department of Bioengineering, University of California San Diego, La Jolla, CA USA; 218 UC San Diego Moores Cancer Center, San Diego, CA USA; 219 Canada’s Michael Smith Genome Sciences Centre, BC Cancer, Vancouver, BC Canada; 220 Sir Peter MacCallum Department of Oncology, Peter MacCallum Cancer Centre, University of Melbourne, Melbourne, VIC Australia; 221 Centre for Research in Molecular Medicine and Chronic Diseases (CiMUS), Universidade de Santiago de Compostela, Santiago de Compostela, Spain; 222 Department of Zoology, Genetics and Physical Anthropology, (CiMUS), Universidade de Santiago de Compostela, Santiago de Compostela, Spain; 223 The Biomedical Research Centre (CINBIO), Universidade de Vigo, Vigo, Spain; 224 Royal National Orthopaedic Hospital - Bolsover, London, UK; 225 Department of Genomic Medicine, The University of Texas MD Anderson Cancer Center, Houston, TX USA; 226 Quantitative and Computational Biosciences Graduate Program, Baylor College of Medicine, Houston, TX USA; 227 The Jackson Laboratory for Genomic Medicine, Farmington, CT USA; 228 Genome Informatics Program, Ontario Institute for Cancer Research, Toronto, ON Canada; 229 Institute of Human Genetics, Christian-Albrechts-University, Kiel, Germany; 230 Institute of Human Genetics, Ulm University and Ulm University Medical Center, Ulm, Germany; 231 Queensland Centre for Medical Genomics, Institute for Molecular Bioscience, University of Queensland, St. Lucia, Brisbane, QLD Australia; 232 Salford Royal NHS Foundation Trust, Salford, UK; 233 Department of Surgery, Pancreas Institute, University and Hospital Trust of Verona, Verona, Italy; 234 Molecular and Medical Genetics, OHSU Knight Cancer Institute, Oregon Health and Science University, Portland, OR USA; 235 Department of Molecular Oncology, BC Cancer Research Centre, Vancouver, BC Canada; 236 The McDonnell Genome Institute at Washington University, St. Louis, MO USA; 237 University College London, London, UK; 238 Division of Cancer Genomics, National Cancer Center Research Institute, National Cancer Center, Tokyo, Japan; 239 DLR Project Management Agency, Bonn, Germany; 240 Tokyo Women’s Medical University, Tokyo, Japan; 241 Center for Molecular Oncology, Memorial Sloan Kettering Cancer Center, New York, NY USA; 242 Los Alamos National Laboratory, Los Alamos, NM USA; 243 Department of Pathology, University Health Network, Toronto General Hospital, Toronto, ON Canada; 244 Nottingham University Hospitals NHS Trust, Nottingham, UK; 245 Epigenomics and Cancer Risk Factors, German Cancer Research Center (DKFZ), Heidelberg, Germany; 246 Computational Biology Program, Ontario Institute for Cancer Research, Toronto, ON Canada; 247 Department of Molecular Genetics, University of Toronto, Toronto, ON Canada; 248 Vector Institute, Toronto, ON Canada; 249 Hematopathology Section, Institute of Pathology, Christian-Albrechts-University, Kiel, Germany; 250 Department of Pathology and Laboratory Medicine, School of Medicine, University of North Carolina at Chapel Hill, Chapel Hill, NC USA; 251 Department of Cancer Genetics, Institute for Cancer Research, Oslo University Hospital, The Norwegian Radium Hospital, Oslo, Norway; 252 Pathology, Hospital Clinic, Institut d’Investigacions Biomèdiques August Pi i Sunyer (IDIBAPS), University of Barcelona, Barcelona, Spain; 253 Department of Veterinary Medicine, Transmissible Cancer Group, University of Cambridge, Cambridge, UK; 254 Alvin J. Siteman Cancer Center, Washington University School of Medicine, St. Louis, MO USA; 255 Wolfson Wohl Cancer Research Centre, Institute of Cancer Sciences, University of Glasgow, Glasgow, UK; 256 Lineberger Comprehensive Cancer Center, University of North Carolina at Chapel Hill, Chapel Hill, NC USA; 257 Broad Institute of MIT and Harvard, Cambridge, MA USA; 258 Dana-Farber/Boston Children’s Cancer and Blood Disorders Center, Boston, MA USA; 259 Department of Pediatrics, Harvard Medical School, Boston, MA USA; 260 Leeds Institute of Medical Research @ St. James’s, University of Leeds, St. James’s University Hospital, Leeds, UK; 261 Department of Pathology and Diagnostics, University and Hospital Trust of Verona, Verona, Italy; 262 Department of Surgery, Princess Alexandra Hospital, Brisbane, QLD Australia; 263 Surgical Oncology Group, Diamantina Institute, University of Queensland, Brisbane, QLD Australia; 264 Department of Population and Quantitative Health Sciences, Case Western Reserve University School of Medicine, Cleveland, OH USA; 265 Research Health Analytics and Informatics, University Hospitals Cleveland Medical Center, Cleveland, OH USA; 266 Gloucester Royal Hospital, Gloucester, UK; 267 European Molecular Biology Laboratory, European Bioinformatics Institute (EMBL-EBI), Cambridge, UK; 268 Diagnostic Development, Ontario Institute for Cancer Research, Toronto, ON Canada; 269 Barcelona Supercomputing Center (BSC), Barcelona, Spain; 270 Arnie Charbonneau Cancer Institute, University of Calgary, Calgary, AB Canada; 271 Departments of Surgery and Oncology, University of Calgary, Calgary, AB Canada; 272 Department of Pathology, Oslo University Hospital, The Norwegian Radium Hospital, Oslo, Norway; 273 PanCuRx Translational Research Initiative, Ontario Institute for Cancer Research, Toronto, ON Canada; 274 Department of Oncology, Sidney Kimmel Comprehensive Cancer Center at Johns Hopkins University School of Medicine, Baltimore, MD USA; 275 University Hospital Southampton NHS Foundation Trust, Southampton, UK; 276 Royal Stoke University Hospital, Stoke-on-Trent, UK; 277 Genome Sequence Informatics, Ontario Institute for Cancer Research, Toronto, ON Canada; 278 Human Longevity Inc, San Diego, CA USA; 279 Olivia Newton-John Cancer Research Institute, La Trobe University, Heidelberg, VIC Australia; 280 Computer Network Information Center, Chinese Academy of Sciences, Beijing, China; 281 Genome Canada, Ottawa, ON Canada; 282 CNAG-CRG, Centre for Genomic Regulation (CRG), Barcelona Institute of Science and Technology (BIST), Barcelona, Spain; 283 Universitat Pompeu Fabra (UPF), Barcelona, Spain; 284 Buck Institute for Research on Aging, Novato, CA USA; 285 Duke University Medical Center, Durham, NC USA; 286 Department of Human Genetics, Hannover Medical School, Hannover, Germany; 287 Center for Bioinformatics and Functional Genomics, Cedars-Sinai Medical Center, Los Angeles, CA USA; 288 Department of Biomedical Sciences, Cedars-Sinai Medical Center, Los Angeles, CA USA; 289 The Hebrew University Faculty of Medicine, Jerusalem, Israel; 290 Barts Cancer Institute, Barts and the London School of Medicine and Dentistry, Queen Mary University of London, London, UK; 291 Department of Computer Science, Bioinformatics Group, University of Leipzig, Leipzig, Germany; 292 Interdisciplinary Center for Bioinformatics, University of Leipzig, Leipzig, Germany; 293 Transcriptome Bioinformatics, LIFE Research Center for Civilization Diseases, University of Leipzig, Leipzig, Germany; 294 Department of Medical Oncology, Dana-Farber Cancer Institute, Boston, MA USA; 295 Department of Cancer Biology, Dana-Farber Cancer Institute, Boston, MA USA; 296 Harvard Medical School, Boston, MA USA; 297 USC Norris Comprehensive Cancer Center, University of Southern California, Los Angeles, CA USA; 298 Department of Diagnostics and Public Health, University and Hospital Trust of Verona, Verona, Italy; 299 Department of Mathematics, Aarhus University, Aarhus, Denmark; 300 Department of Molecular Medicine (MOMA), Aarhus University Hospital, Aarhus N, Denmark; 301 Instituto Carlos Slim de la Salud, Mexico City, Mexico; 302 Department of Medical Biophysics, University of Toronto, Toronto, ON Canada; 303 Cancer Division, Garvan Institute of Medical Research, Kinghorn Cancer Centre, University of New South Wales (UNSW Sydney), Sydney, NSW Australia; 304 South Western Sydney Clinical School, Faculty of Medicine, University of New South Wales (UNSW Sydney), Liverpool, NSW Australia; 305 West of Scotland Pancreatic Unit, Glasgow Royal Infirmary, Glasgow, UK; 306 Center for Digital Health, Berlin Institute of Health and Charitè - Universitätsmedizin Berlin, Berlin, Germany; 307 Heidelberg Center for Personalized Oncology (DKFZ-HIPO), German Cancer Research Center (DKFZ), Heidelberg, Germany; 308 The Preston Robert Tisch Brain Tumor Center, Duke University Medical Center, Durham, NC USA; 309 Massachusetts General Hospital, Boston, MA USA; 310 National Institute of Biomedical Genomics, Kalyani, West Bengal India; 311 Institute of Clinical Medicine and Institute of Oral Biology, University of Oslo, Oslo, Norway; 312 University of North Carolina at Chapel Hill, Chapel Hill, NC USA; 313 ARC-Net Centre for Applied Research on Cancer, University and Hospital Trust of Verona, Verona, Italy; 314 The Institute of Cancer Research, London, UK; 315 Centre for Computational Biology, Duke-NUS Medical School, Singapore, Singapore; 316 Programme in Cancer and Stem Cell Biology, Duke-NUS Medical School, Singapore, Singapore; 317 Division of Oncology and Pathology, Department of Clinical Sciences Lund, Lund University, Lund, Sweden; 318 Department of Pediatric Oncology, Hematology and Clinical Immunology, Heinrich-Heine-University, Düsseldorf, Germany; 319 Laboratory for Medical Science Mathematics, RIKEN Center for Integrative Medical Sciences, Yokohama, Japan; 320 RIKEN Center for Integrative Medical Sciences, Yokohama, Japan; 321 Department of Internal Medicine/Hematology, Friedrich-Ebert-Hospital, Neumünster, Germany; 322 Departments of Dermatology and Pathology, Yale University, New Haven, CT USA; 323 Centre for Genomic Regulation (CRG), The Barcelona Institute of Science and Technology, Barcelona, Spain; 324 Radcliffe Department of Medicine, University of Oxford, Oxford, UK; 325 Canadian Center for Computational Genomics, McGill University, Montreal, QC Canada; 326 Department of Human Genetics, McGill University, Montreal, QC Canada; 327 Department of Human Genetics, University of California Los Angeles, Los Angeles, CA USA; 328 Department of Pharmacology, University of Toronto, Toronto, ON Canada; 329 Faculty of Medicine and Health Technology, Tampere University and Tays Cancer Center, Tampere University Hospital, Tampere, Finland; 330 Haematology, Leeds Teaching Hospitals NHS Trust, Leeds, UK; 331 Translational Research and Innovation, Centre Léon Bérard, Lyon, France; 332 Fox Chase Cancer Center, Philadelphia, PA USA; 333 International Agency for Research on Cancer, World Health Organization, Lyon, France; 334 Earlham Institute, Norwich, UK; 335 Norwich Medical School, University of East Anglia, Norwich, UK; 336 Department of Molecular Biology, Faculty of Science, Radboud Institute for Molecular Life Sciences, Radboud University, Nijmegen, HB The Netherlands; 337 CRUK Manchester Institute and Centre, Manchester, UK; 338 Department of Radiation Oncology, University of Toronto, Toronto, ON Canada; 339 Division of Cancer Sciences, Manchester Cancer Research Centre, University of Manchester, Manchester, UK; 340 Radiation Medicine Program, Princess Margaret Cancer Centre, Toronto, ON Canada; 341 Department of Pathology, Brigham and Women’s Hospital, Harvard Medical School, Boston, MA USA; 342 Department of Surgery, Division of Thoracic Surgery, The Johns Hopkins University School of Medicine, Baltimore, MD USA; 343 Division of Molecular Pathology, The Netherlands Cancer Institute, Oncode Institute, Amsterdam, CX The Netherlands; 344 Department of Biomolecular Engineering, University of California Santa Cruz, Santa Cruz, CA USA; 345 UC Santa Cruz Genomics Institute, University of California Santa Cruz, Santa Cruz, CA USA; 346 Division of Applied Bioinformatics, German Cancer Research Center (DKFZ), Heidelberg, Germany; 347 German Cancer Consortium (DKTK), German Cancer Research Center (DKFZ), Heidelberg, Germany; 348 National Center for Tumor Diseases (NCT) Heidelberg, Heidelberg, Germany; 349 Center for Biological Sequence Analysis, Department of Bio and Health Informatics, Technical University of Denmark, Lyngby, Denmark; 350 Novo Nordisk Foundation Center for Protein Research, University of Copenhagen, Copenhagen, Denmark; 351 Institute for Molecular Bioscience, University of Queensland, St. Lucia, Brisbane, QLD Australia; 352 Biomedical Engineering, Oregon Health and Science University, Portland, OR USA; 353 Division of Theoretical Bioinformatics, German Cancer Research Center (DKFZ), Heidelberg, Germany; 354 Institute of Pharmacy and Molecular Biotechnology and BioQuant, Heidelberg University, Heidelberg, Germany; 355 Federal Ministry of Education and Research, Berlin, Germany; 356 Melanoma Institute Australia, University of Sydney, Sydney, NSW Australia; 357 Pediatric Hematology and Oncology, University Hospital Muenster, Muenster, Germany; 358 Department of Pathology, Johns Hopkins University School of Medicine, Baltimore, MD USA; 359 McKusick-Nathans Institute of Genetic Medicine, Sidney Kimmel Comprehensive Cancer Center at Johns Hopkins University School of Medicine, Baltimore, MD USA; 360 Foundation Medicine, Inc, Cambridge, MA USA; 361 Department of Biomedical Data Science, Stanford University School of Medicine, Stanford, CA USA; 362 Department of Genetics, Stanford University School of Medicine, Stanford, CA USA; 363 Bakar Computational Health Sciences Institute and Department of Pediatrics, University of California, San Francisco, CA USA; 364 Institute of Clinical Medicine, Faculty of Medicine, University of Oslo, Oslo, Norway; 365 National Cancer Institute, National Institutes of Health, Bethesda, MD USA; 366 Royal Marsden NHS Foundation Trust, London and Sutton, UK; 367 Genome Biology Unit, European Molecular Biology Laboratory (EMBL), Heidelberg, Germany; 368 Department of Oncology, University of Cambridge, Cambridge, UK; 369 Li Ka Shing Centre, Cancer Research UK Cambridge Institute, University of Cambridge, Cambridge, UK; 370 Institut Gustave Roussy, Villejuif, France; 371 Cambridge University Hospitals NHS Foundation Trust, Cambridge, UK; 372 Department of Haematology, University of Cambridge, Cambridge, UK; 373 Anatomia Patológica, Hospital Clinic, Institut d’Investigacions Biomèdiques August Pi i Sunyer (IDIBAPS), University of Barcelona, Barcelona, Spain; 374 Spanish Ministry of Science and Innovation, Madrid, Spain; 375 University of Michigan Comprehensive Cancer Center, Ann Arbor, MI USA; 376 Department for BioMedical Research, University of Bern, Bern, Switzerland; 377 Department of Medical Oncology, Inselspital, University Hospital and University of Bern, Bern, Switzerland; 378 Graduate School for Cellular and Biomedical Sciences, University of Bern, Bern, Switzerland; 379 University of Pavia, Pavia, Italy; 380 University of Alabama at Birmingham, Birmingham, AL USA; 381 UHN Program in BioSpecimen Sciences, Toronto General Hospital, Toronto, ON Canada; 382 Department of Urology, Icahn School of Medicine at Mount Sinai, New York, NY USA; 383 Centre for Law and Genetics, University of Tasmania, Sandy Bay Campus, Hobart, TAS Australia; 384 Faculty of Biosciences, Heidelberg University, Heidelberg, Germany; 385 Department of Biochemistry, Microbiology and Immunology, Faculty of Medicine, University of Ottawa, Ottawa, ON Canada; 386 Division of Anatomic Pathology, Mayo Clinic, Rochester, MN USA; 387 Division of Cancer Epidemiology and Genetics, National Cancer Institute, National Institutes of Health, Bethesda, MD USA; 388 Illawarra Shoalhaven Local Health District L3 Illawarra Cancer Care Centre, Wollongong Hospital, Wollongong, NSW Australia; 389 BioForA, French National Institute for Agriculture, Food, and Environment (INRAE), ONF, Orléans, France; 390 Department of Biostatistics, Bloomberg School of Public Health, Johns Hopkins University, Baltimore, MD USA; 391 University of California San Diego, San Diego, CA USA; 392 Division of Experimental Pathology, Mayo Clinic, Rochester, MN USA; 393 Centre for Cancer Research, The Westmead Institute for Medical Research, University of Sydney, Sydney, NSW Australia; 394 Department of Gynaecological Oncology, Westmead Hospital, Sydney, NSW Australia; 395 PDXen Biosystems Inc, Seoul, South Korea; 396 Korea Advanced Institute of Science and Technology, Daejeon, South Korea; 397 Electronics and Telecommunications Research Institute, Daejeon, South Korea; 398 Institut National du Cancer (INCA), Boulogne-Billancourt, France; 399 Department of Genetics, Informatics Institute, University of Alabama at Birmingham, Birmingham, AL USA; 400 Division of Medical Oncology, National Cancer Centre, Singapore, Singapore; 401 Medical Oncology, University and Hospital Trust of Verona, Verona, Italy; 402 Department of Pediatrics, University Hospital Schleswig-Holstein, Kiel, Germany; 403 Hepatobiliary/Pancreatic Surgical Oncology Program, University Health Network, Toronto, ON Canada; 404 School of Biological Sciences, University of Auckland, Auckland, New Zealand; 405 Department of Surgery, University of Melbourne, Parkville, VIC Australia; 406 The Murdoch Children’s Research Institute, Royal Children’s Hospital, Parkville, VIC Australia; 407 Walter and Eliza Hall Institute, Parkville, VIC Australia; 408 Vancouver Prostate Centre, Vancouver, Canada; 409 Lunenfeld-Tanenbaum Research Institute, Mount Sinai Hospital, Toronto, ON Canada; 410 University of East Anglia, Norwich, UK; 411 Norfolk and Norwich University Hospital NHS Trust, Norwich, UK; 412 Victorian Institute of Forensic Medicine, Southbank, VIC Australia; 413 Department of Biomedical Informatics, Harvard Medical School, Boston, MA USA; 414 Department of Chemistry, Centre for Molecular Science Informatics, University of Cambridge, Cambridge, UK; 415 Ludwig Center at Harvard Medical School, Boston, MA USA; 416 Human Genome Sequencing Center, Baylor College of Medicine, Houston, TX USA; 417 Peter MacCallum Cancer Centre, University of Melbourne, Melbourne, VIC Australia; 418 Physics Division, Optimization and Systems Biology Lab, Massachusetts General Hospital, Boston, MA USA; 419 Department of Medicine, Baylor College of Medicine, Houston, TX USA; 420 University of Cologne, Cologne, Germany; 421 International Genomics Consortium, Phoenix, AZ USA; 422 Genomics Research Program, Ontario Institute for Cancer Research, Toronto, ON Canada; 423 Barking Havering and Redbridge University Hospitals NHS Trust, Romford, UK; 424 Children’s Hospital at Westmead, University of Sydney, Sydney, NSW Australia; 425 Department of Medicine, Section of Endocrinology, University and Hospital Trust of Verona, Verona, Italy; 426 Computational Biology Center, Memorial Sloan Kettering Cancer Center, New York, NY USA; 427 Department of Biology, ETH Zurich, Zürich, Switzerland; 428 Department of Computer Science, ETH Zurich, Zurich, Switzerland; 429 SIB Swiss Institute of Bioinformatics, Lausanne, Switzerland; 430 Weill Cornell Medical College, New York, NY USA; 431 Academic Department of Medical Genetics, University of Cambridge, Addenbrooke’s Hospital, Cambridge, UK; 432 MRC Cancer Unit, University of Cambridge, Cambridge, UK; 433 Departments of Pediatrics and Genetics, University of North Carolina at Chapel Hill, Chapel Hill, NC USA; 434 Seven Bridges Genomics, Charlestown, MA USA; 435 Annai Systems, Inc, Carlsbad, CA USA; 436 Department of Pathology, General Hospital of Treviso, Department of Medicine, University of Padua, Treviso, Italy; 437 Department of Computational Biology, University of Lausanne, Lausanne, Switzerland; 438 Department of Genetic Medicine and Development, University of Geneva Medical School, Geneva, CH Switzerland; 439 Swiss Institute of Bioinformatics, University of Geneva, Geneva, CH Switzerland; 440 The Francis Crick Institute, London, UK; 441 University of Leuven, Leuven, Belgium; 442 Institute of Medical Genetics and Applied Genomics, University of Tübingen, Tübingen, Germany; 443 Computational and Systems Biology, Genome Institute of Singapore, Singapore, Singapore; 444 School of Computing, National University of Singapore, Singapore, Singapore; 445 Big Data Institute, Li Ka Shing Centre, University of Oxford, Oxford, UK; 446 Biomedical Data Science Laboratory, Francis Crick Institute, London, UK; 447 Bioinformatics Group, Department of Computer Science, University College London, London, UK; 448 The Edward S. Rogers Sr. Department of Electrical and Computer Engineering, University of Toronto, Toronto, ON Canada; 449 Breast Cancer Translational Research Laboratory JC Heuson, Institut Jules Bordet, Brussels, Belgium; 450 Department of Oncology, Laboratory for Translational Breast Cancer Research, KU Leuven, Leuven, Belgium; 451 Institute for Research in Biomedicine (IRB Barcelona), The Barcelona Institute of Science and Technology, Barcelona, Spain; 452 Research Program on Biomedical Informatics, Universitat Pompeu Fabra, Barcelona, Spain; 453 Division of Medical Oncology, Princess Margaret Cancer Centre, Toronto, ON Canada; 454 Department of Physiology and Biophysics, Weill Cornell Medicine, New York, NY USA; 455 Institute for Computational Biomedicine, Weill Cornell Medicine, New York, NY USA; 456 Department of Pathology, UPMC Shadyside, Pittsburgh, PA USA; 457 Independent Consultant, Wellesley, USA; 458 Department of Cell and Molecular Biology, Science for Life Laboratory, Uppsala University, Uppsala, Sweden; 459 Department of Medicine and Department of Genetics, Washington University School of Medicine, St. Louis, St. Louis, MO USA; 460 Hefei University of Technology, Anhui, China; 461 Translational Cancer Research Unit, GZA Hospitals St.-Augustinus, Center for Oncological Research, Faculty of Medicine and Health Sciences, University of Antwerp, Antwerp, Belgium; 462 Simon Fraser University, Burnaby, BC Canada; 463 University of Pennsylvania, Philadelphia, PA USA; 464 Faculty of Science and Technology, University of Vic—Central University of Catalonia (UVic-UCC), Vic, Spain; 465 The Wellcome Trust, London, UK; 466 The Hospital for Sick Children, Toronto, ON Canada; 467 Department of Pathology, Queen Elizabeth University Hospital, Glasgow, UK; 468 Department of Genetics and Computational Biology, QIMR Berghofer Medical Research Institute, Brisbane, QLD Australia; 469 Department of Oncology, Centre for Cancer Genetic Epidemiology, University of Cambridge, Cambridge, UK; 470 Department of Public Health and Primary Care, Centre for Cancer Genetic Epidemiology, University of Cambridge, Cambridge, UK; 471 Prostate Cancer Canada, Toronto, ON Canada; 472 University of Cambridge, Cambridge, UK; 473 Department of Laboratory Medicine, Translational Cancer Research, Lund University Cancer Center at Medicon Village, Lund University, Lund, Sweden; 474 Heidelberg University, Heidelberg, Germany; 475 New BIH Digital Health Center, Berlin Institute of Health (BIH) and Charité - Universitätsmedizin Berlin, Berlin, Germany; 476 CIBER Epidemiología y Salud Pública (CIBERESP), Madrid, Spain; 477 Research Group on Statistics, Econometrics and Health (GRECS), UdG, Barcelona, Spain; 478 Quantitative Genomics Laboratories (qGenomics), Barcelona, Spain; 479 Icelandic Cancer Registry, Icelandic Cancer Society, Reykjavik, Iceland; 480 State Key Laboratory of Cancer Biology, and Xijing Hospital of Digestive Diseases, Fourth Military Medical University, Shaanxi, China; 481 Department of Medicine (DIMED), Surgical Pathology Unit, University of Padua, Padua, Italy; 482 Rigshospitalet, Copenhagen, Denmark; 483 Center for Cancer Genomics, National Cancer Institute, National Institutes of Health, Bethesda, MD USA; 484 Department of Biochemistry and Molecular Medicine, University of Montreal, Montreal, QC Canada; 485 Australian Institute of Tropical Health and Medicine, James Cook University, Douglas, QLD Australia; 486 Department of Neuro-Oncology, Istituto Neurologico Besta, Milano, Italy; 487 Bioplatforms Australia, North Ryde, NSW Australia; 488 Department of Pathology (Research), University College London Cancer Institute, London, UK; 489 Department of Surgical Oncology, Princess Margaret Cancer Centre, Toronto, ON Canada; 490 Department of Medical Oncology, Josephine Nefkens Institute and Cancer Genomics Centre, Erasmus Medical Center, Rotterdam, CN The Netherlands; 491 The University of Queensland Thoracic Research Centre, The Prince Charles Hospital, Brisbane, QLD Australia; 492 CIBIO/InBIO - Research Center in Biodiversity and Genetic Resources, Universidade do Porto, Vairão, Portugal; 493 HCA Laboratories, London, UK; 494 University of Liverpool, Liverpool, UK; 495 The Azrieli Faculty of Medicine, Bar-Ilan University, Safed, Israel; 496 Department of Neurosurgery, University of Florida, Gainesville, FL USA; 497 Department of Pathology, Graduate School of Medicine, University of Tokyo, Tokyo, Japan; 498 University of Milano Bicocca, Monza, Italy; 499 BGI-Shenzhen, Shenzhen, China; 500 Department of Pathology, Oslo University Hospital Ulleval, Oslo, Norway; 501 Center for Biomedical Informatics, Harvard Medical School, Boston, MA USA; 502 Department Biochemistry and Molecular Biomedicine, University of Barcelona, Barcelona, Spain; 503 Office of Cancer Genomics, National Cancer Institute, National Institutes of Health, Bethesda, MD USA; 504 Cancer Epigenomics, German Cancer Research Center (DKFZ), Heidelberg, Germany; 505 Department of Cancer Biology, The University of Texas MD Anderson Cancer Center, Houston, TX USA; 506 Department of Surgical Oncology, The University of Texas MD Anderson Cancer Center, Houston, TX USA; 507 Department of Computer Science, Yale University, New Haven, CT USA; 508 Department of Molecular Biophysics and Biochemistry, Yale University, New Haven, CT USA; 509 Program in Computational Biology and Bioinformatics, Yale University, New Haven, CT USA; 510 Center for Cancer Research, Massachusetts General Hospital, Boston, MA USA; 511 Department of Pathology, Massachusetts General Hospital, Boston, MA USA; 512 Department of Pathology, Memorial Sloan Kettering Cancer Center, New York, NY USA; 513 Division of Gastroenterology and Hepatology, Mayo Clinic, Rochester, MN USA; 514 University of Sydney, Sydney, NSW Australia; 515 University of Oxford, Oxford, UK; 516 Department of Surgery, Academic Urology Group, University of Cambridge, Cambridge, UK; 517 Department of Medicine II, University of Würzburg, Wuerzburg, Germany; 518 Sylvester Comprehensive Cancer Center, University of Miami, Miami, FL USA; 519 Institut Hospital del Mar d’Investigacions Mèdiques (IMIM), Barcelona, Spain; 520 Genome Integrity and Structural Biology Laboratory, National Institute of Environmental Health Sciences (NIEHS), Durham, NC USA; 521 St. Thomas’s Hospital, London, UK; 522 Osaka International Cancer Center, Osaka, Japan; 523 Department of Pathology, Skåne University Hospital, Lund University, Lund, Sweden; 524 Department of Medical Oncology, Beatson West of Scotland Cancer Centre, Glasgow, UK; 525 National Human Genome Research Institute, National Institutes of Health, Bethesda, MD USA; 526 Centre for Cancer Research, Victorian Comprehensive Cancer Centre, University of Melbourne, Melbourne, VIC Australia; 527 Department of Medicine, Section of Hematology/Oncology, University of Chicago, Chicago, IL USA; 528 German Center for Infection Research (DZIF), Partner Site Hamburg-Borstel-Lübeck-Riems, Hamburg, Germany; 529 Bioinformatics Research Centre (BiRC), Aarhus University, Aarhus, Denmark; 530 Department of Biotechnology, Ministry of Science and Technology, Government of India, New Delhi, Delhi India; 531 National Cancer Centre Singapore, Singapore, Singapore; 532 Brandeis University, Waltham, MA USA; 533 Department of Urologic Sciences, University of British Columbia, Vancouver, BC Canada; 534 Department of Internal Medicine, Stanford University, Stanford, CA USA; 535 The University of Texas Health Science Center at Houston, Houston, TX USA; 536 Imperial College NHS Trust, Imperial College, London, INY UK; 537 Senckenberg Institute of Pathology, University of Frankfurt Medical School, Frankfurt, Germany; 538 Department of Medicine, Division of Biomedical Informatics, UC San Diego School of Medicine, San Diego, CA USA; 539 Center for Precision Health, School of Biomedical Informatics, The University of Texas Health Science Center, Houston, TX USA; 540 Oxford Nanopore Technologies, New York, NY USA; 541 Institute of Medical Science, University of Tokyo, Tokyo, Japan; 542 Howard Hughes Medical Institute, University of California Santa Cruz, Santa Cruz, CA USA; 543 Wakayama Medical University, Wakayama, Japan; 544 Department of Internal Medicine, Division of Medical Oncology, Lineberger Comprehensive Cancer Center, University of North Carolina at Chapel Hill, Chapel Hill, NC USA; 545 University of Tennessee Health Science Center for Cancer Research, Memphis, TN USA; 546 Department of Histopathology, Salford Royal NHS Foundation Trust, Salford, UK; 547 Faculty of Biology, Medicine and Health, University of Manchester, Manchester, UK; 548 BIOPIC, ICG and College of Life Sciences, Peking University, Beijing, China; 549 Peking-Tsinghua Center for Life Sciences, Peking University, Beijing, China; 550 Children’s Hospital of Philadelphia, Philadelphia, PA USA; 551 Department of Bioinformatics and Computational Biology and Department of Systems Biology, The University of Texas MD Anderson Cancer Center, Houston, TX USA; 552 Karolinska Institute, Stockholm, Sweden; 553 The Donnelly Centre, University of Toronto, Toronto, ON Canada; 554 Department of Medical Genetics, College of Medicine, Hallym University, Chuncheon, South Korea; 555 Department of Experimental and Health Sciences, Institute of Evolutionary Biology (UPF-CSIC), Universitat Pompeu Fabra, Barcelona, Spain; 556 Health Data Science Unit, University Clinics, Heidelberg, Germany; 557 Massachusetts General Hospital Center for Cancer Research, Charlestown, MA USA; 558 Hokkaido University, Sapporo, Japan; 559 Department of Pathology and Clinical Laboratory, National Cancer Center Hospital, Tokyo, Japan; 560 Department of Genetics, University of North Carolina at Chapel Hill, Chapel Hill, NC USA; 561 Computational Biology, Leibniz Institute on Aging - Fritz Lipmann Institute (FLI), Jena, Germany; 562 University of Melbourne Centre for Cancer Research, Melbourne, VIC Australia; 563 University of Nebraska Medical Center, Omaha, NE USA; 564 Syntekabio Inc, Daejeon, South Korea; 565 Department of Pathology, Academic Medical Center, Amsterdam, AZ The Netherlands; 566 China National GeneBank-Shenzhen, Shenzhen, China; 567 Division of Molecular Genetics, German Cancer Research Center (DKFZ), Heidelberg, Germany; 568 Division of Life Science and Applied Genomics Center, Hong Kong University of Science and Technology, Clear Water Bay, Hong Kong, China; 569 Icahn School of Medicine at Mount Sinai, New York, NY USA; 570 Geneplus-Shenzhen, Shenzhen, China; 571 School of Computer Science and Technology, Xi’an Jiaotong University, Xi’an, China; 572 AbbVie, North Chicago, IL USA; 573 Institute of Pathology, Charité – University Medicine Berlin, Berlin, Germany; 574 Centre for Translational and Applied Genomics, British Columbia Cancer Agency, Vancouver, BC Canada; 575 Edinburgh Royal Infirmary, Edinburgh, UK; 576 Berlin Institute for Medical Systems Biology, Max Delbrück Center for Molecular Medicine, Berlin, Germany; 577 Department of Pediatric Immunology, Hematology and Oncology, University Hospital, Heidelberg, Germany; 578 German Cancer Research Center (DKFZ), Heidelberg, Germany; 579 Heidelberg Institute for Stem Cell Technology and Experimental Medicine (HI-STEM), Heidelberg, Germany; 580 Institute for Computational Biomedicine, Weill Cornell Medical College, New York, NY USA; 581 New York Genome Center, New York, NY USA; 582 Department of Urology, James Buchanan Brady Urological Institute, Johns Hopkins University School of Medicine, Baltimore, MD USA; 583 Department of Preventive Medicine, Graduate School of Medicine, The University of Tokyo, Tokyo, Japan; 584 Department of Molecular and Cellular Biology, Baylor College of Medicine, Houston, TX USA; 585 Department of Pathology and Immunology, Baylor College of Medicine, Houston, TX USA; 586 Michael E. DeBakey Veterans Affairs Medical Center, Houston, TX USA; 587 Technical University of Denmark, Lyngby, Denmark; 588 Department of Pathology, College of Medicine, Hanyang University, Seoul, South Korea; 589 Academic Unit of Surgery, School of Medicine, College of Medical, Veterinary and Life Sciences, University of Glasgow, Glasgow Royal Infirmary, Glasgow, UK; 590 Department of Pathology, Asan Medical Center, College of Medicine, Ulsan University, Songpa-gu, Seoul South Korea; 591 Science Writer, Garrett Park, MD USA; 592 International Cancer Genome Consortium (ICGC)/ICGC Accelerating Research in Genomic Oncology (ARGO) Secretariat, Ontario Institute for Cancer Research, Toronto, ON Canada; 593 University of Ljubljana, Ljubljana, Slovenia; 594 Department of Public Health Sciences, University of Chicago, Chicago, IL USA; 595 Research Institute, NorthShore University HealthSystem, Evanston, IL USA; 596 Department for Biomedical Research, University of Bern, Bern, Switzerland; 597 Centre of Genomics and Policy, McGill University and Génome Québec Innovation Centre, Montreal, QC Canada; 598 Carolina Center for Genome Sciences, University of North Carolina at Chapel Hill, Chapel Hill, NC USA; 599 Hopp Children’s Cancer Center (KiTZ), Heidelberg, Germany; 600 Pediatric Glioma Research Group, German Cancer Research Center (DKFZ), Heidelberg, Germany; 601 Cancer Research UK, London, UK; 602 Indivumed GmbH, Hamburg, Germany; 603 Genome Integration Data Center, Syntekabio, Inc, Daejeon, South Korea; 604 University Hospital Zurich, Zurich, Switzerland; 605 Clinical Bioinformatics, Swiss Institute of Bioinformatics, Geneva, Switzerland; 606 Institute for Pathology and Molecular Pathology, University Hospital Zurich, Zurich, Switzerland; 607 Institute of Molecular Life Sciences, University of Zurich, Zurich, Switzerland; 608 MRC Human Genetics Unit, MRC IGMM, University of Edinburgh, Edinburgh, UK; 609 Women’s Cancer Program at the Samuel Oschin Comprehensive Cancer Institute, Cedars-Sinai Medical Center, Los Angeles, CA USA; 610 Department of Biology, Bioinformatics Group, Division of Molecular Biology, Faculty of Science, University of Zagreb, Zagreb, Croatia; 611 Department for Internal Medicine II, University Hospital Schleswig-Holstein, Kiel, Germany; 612 Genetics and Molecular Pathology, SA Pathology, Adelaide, SA Australia; 613 Department of Gastric Surgery, National Cancer Center Hospital, Tokyo, Japan; 614 Department of Bioinformatics, Division of Cancer Genomics, National Cancer Center Research Institute, Tokyo, Japan; 615 A.A. Kharkevich Institute of Information Transmission Problems, Moscow, Russia; 616 Oncology and Immunology, Dmitry Rogachev National Research Center of Pediatric Hematology, Moscow, Russia; 617 Skolkovo Institute of Science and Technology, Moscow, Russia; 618 Department of Surgery, The George Washington University, School of Medicine and Health Science, Washington, DC USA; 619 Endocrine Oncology Branch, Center for Cancer Research, National Cancer Institute, National Institutes of Health, Bethesda, MD USA; 620 Melanoma Institute Australia, Macquarie University, Sydney, NSW Australia; 621 MIT Computer Science and Artificial Intelligence Laboratory, Massachusetts Institute of Technology, Cambridge, MA USA; 622 Tissue Pathology and Diagnostic Oncology, Royal Prince Alfred Hospital, Sydney, NSW Australia; 623 Cholangiocarcinoma Screening and Care Program and Liver Fluke and Cholangiocarcinoma Research Centre, Faculty of Medicine, Khon Kaen University, Khon Kaen, Thailand; 624 Controlled Department and Institution, New York, NY USA; 625 Englander Institute for Precision Medicine, Weill Cornell Medicine, New York, NY USA; 626 National Cancer Center, Gyeonggi, South Korea; 627 Department of Biochemistry, College of Medicine, Ewha Womans University, Seoul, South Korea; 628 Health Sciences Department of Biomedical Informatics, University of California San Diego, La Jolla, CA USA; 629 Research Core Center, National Cancer Centre Korea, Goyang-si, South Korea; 630 Department of Health Sciences and Technology, Sungkyunkwan University School of Medicine, Seoul, South Korea; 631 Samsung Genome Institute, Seoul, South Korea; 632 Breast Oncology Program, Dana-Farber/Brigham and Women’s Cancer Center, Boston, MA USA; 633 Department of Surgery, Memorial Sloan Kettering Cancer Center, New York, NY USA; 634 Division of Breast Surgery, Brigham and Women’s Hospital, Boston, MA USA; 635 Integrative Bioinformatics Support Group, National Institute of Environmental Health Sciences (NIEHS), Durham, NC USA; 636 Department of Clinical Science, University of Bergen, Bergen, Norway; 637 Center For Medical Innovation, Seoul National University Hospital, Seoul, South Korea; 638 Department of Internal Medicine, Seoul National University Hospital, Seoul, South Korea; 639 Institute of Computer Science, Polish Academy of Sciences, Warsawa, Poland; 640 Functional and Structural Genomics, German Cancer Research Center (DKFZ), Heidelberg, Germany; 641 Laboratory of Translational Genomics, Division of Cancer Epidemiology and Genetics, National Cancer Institute, , National Institutes of Health, Bethesda, MD USA; 642 Institute for Medical Informatics Statistics and Epidemiology, University of Leipzig, Leipzig, Germany; 643 Morgan Welch Inflammatory Breast Cancer Research Program and Clinic, The University of Texas MD Anderson Cancer Center, Houston, TX USA; 644 Department of Hematology and Oncology, Georg-Augusts-University of Göttingen, Göttingen, Germany; 645 Institute of Cell Biology (Cancer Research), University of Duisburg-Essen, Essen, Germany; 646 King’s College London and Guy’s and St. Thomas’ NHS Foundation Trust, London, UK; 647 Center for Epigenetics, Van Andel Research Institute, Grand Rapids, MI USA; 648 The University of Queensland Centre for Clinical Research, Royal Brisbane and Women’s Hospital, Herston, QLD Australia; 649 Department of Pediatric Oncology and Hematology, University of Cologne, Cologne, Germany; 650 University of Düsseldorf, Düsseldorf, Germany; 651 Department of Pathology, Institut Jules Bordet, Brussels, Belgium; 652 Institute of Biomedicine, Sahlgrenska Academy at University of Gothenburg, Gothenburg, Sweden; 653 Children’s Medical Research Institute, Sydney, NSW Australia; 654 ILSbio, LLC Biobank, Chestertown, MD USA; 655 Division of Genetics and Genomics, Boston Children’s Hospital, Harvard Medical School, Boston, MA USA; 656 Institute for Bioengineering and Biopharmaceutical Research (IBBR), Hanyang University, Seoul, South Korea; 657 Department of Statistics, University of California Santa Cruz, Santa Cruz, CA USA; 658 National Genotyping Center, Institute of Biomedical Sciences, Academia Sinica, Taipei, Taiwan; 659 Department of Vertebrate Genomics/Otto Warburg Laboratory Gene Regulation and Systems Biology of Cancer, Max Planck Institute for Molecular Genetics, Berlin, Germany; 660 McGill University and Genome Quebec Innovation Centre, Montreal, QC Canada; 661 biobyte solutions GmbH, Heidelberg, Germany; 662 Gynecologic Oncology, NYU Laura and Isaac Perlmutter Cancer Center, New York University, New York, NY USA; 663 Division of Oncology, Stem Cell Biology Section, Washington University School of Medicine, St. Louis, MO USA; 664 Department of Systems Biology, The University of Texas MD Anderson Cancer Center, Houston, TX USA; 665 Harvard University, Cambridge, MA USA; 666 Urologic Oncology Branch, Center for Cancer Research, National Cancer Institute, National Institutes of Health, Bethesda, MD USA; 667 University of Oslo, Oslo, Norway; 668 University of Toronto, Toronto, ON Canada; 669 Peking University, Beijing, China; 670 School of Life Sciences, Peking University, Beijing, China; 671 Leidos Biomedical Research, Inc, McLean, VA USA; 672 Hematology, Hospital Clinic, Institut d’Investigacions Biomèdiques August Pi i Sunyer (IDIBAPS), University of Barcelona, Barcelona, Spain; 673 Second Military Medical University, Shanghai, China; 674 Chinese Cancer Genome Consortium, Shenzhen, China; 675 Department of Medical Oncology, Beijing Hospital, Beijing, China; 676 Laboratory of Molecular Oncology, Key Laboratory of Carcinogenesis and Translational Research (Ministry of Education), Peking University Cancer Hospital and Institute, Beijing, China; 677 School of Medicine/School of Mathematics and Statistics, University of St. Andrews, St, Andrews, Fife UK; 678 Institute for Systems Biology, Seattle, WA USA; 679 Department of Biochemistry and Molecular Biology, Faculty of Medicine, University Institute of Oncology-IUOPA, Oviedo, Spain; 680 Institut Bergonié, Bordeaux, France; 681 Cancer Unit, MRC University of Cambridge, Cambridge, UK; 682 Department of Pathology and Laboratory Medicine, Center for Personalized Medicine, Children’s Hospital Los Angeles, Los Angeles, CA USA; 683 John Curtin School of Medical Research, Canberra, ACT Australia; 684 MVZ Department of Oncology, PraxisClinic am Johannisplatz, Leipzig, Germany; 685 Department of Information Technology, Ghent University, Ghent, Belgium; 686 Department of Plant Biotechnology and Bioinformatics, Ghent University, Ghent, Belgium; 687 Institute for Genomic Medicine, Nationwide Children’s Hospital, Columbus, OH USA; 688 Computational Biology Program, School of Medicine, Oregon Health and Science University, Portland, OR USA; 689 Department of Surgery, Duke University, Durham, NC USA; 690 Institució Catalana de Recerca i Estudis Avançats (ICREA), Barcelona, Spain; 691 Institut Català de Paleontologia Miquel Crusafont, Universitat Autònoma de Barcelona, Barcelona, Spain; 692 University of Glasgow, Glasgow, UK; 693 Institut d’Investigacions Biomèdiques August Pi i Sunyer (IDIBAPS), Barcelona, Spain; 694 Division of Oncology, Washington University School of Medicine, St. Louis, MO USA; 695 Department of Surgery and Cancer, Imperial College, London, INY UK; 696 Applications Department, Oxford Nanopore Technologies, Oxford, UK; 697 Department of Obstetrics, Gynecology and Reproductive Services, University of California San Francisco, San Francisco, CA USA; 698 Department of Biochemistry and Molecular Medicine, University California at Davis, Sacramento, CA USA; 699 STTARR Innovation Facility, Princess Margaret Cancer Centre, Toronto, ON Canada; 700 Discipline of Surgery, Western Sydney University, Penrith, NSW Australia; 701 Yale School of Medicine, Yale University, New Haven, CT USA; 702 Department of Genetics, Lineberger Comprehensive Cancer Center, University of North Carolina at Chapel Hill, Chapel Hill, NC USA; 703 Departments of Neurology and Neurosurgery, Henry Ford Hospital, Detroit, MI USA; 704 Precision Oncology, OHSU Knight Cancer Institute, Oregon Health and Science University, Portland, OR USA; 705 Institute of Pathology, University Medical Center Hamburg-Eppendorf, Hamburg, Germany; 706 Department of Health Sciences, Faculty of Medical Sciences, Kyushu University, Fukuoka, Japan; 707 Heidelberg Academy of Sciences and Humanities, Heidelberg, Germany; 708 Department of Clinical Pathology, University of Melbourne, Melbourne, VIC, Australia; 709 Department of Pathology, Roswell Park Cancer Institute, Buffalo, NY USA; 710 Department of Computer Science, University of Helsinki, Helsinki, Finland; 711 Institute of Biotechnology, University of Helsinki, Helsinki, Finland; 712 Organismal and Evolutionary Biology Research Programme, University of Helsinki, Helsinki, Finland; 713 Department of Obstetrics and Gynecology, Division of Gynecologic Oncology, Washington University School of Medicine, St. Louis, MO USA; 714 Penrose St. Francis Health Services, Colorado Springs, CO USA; 715 Institute of Pathology, Ulm University and University Hospital of Ulm, Ulm, Germany; 716 National Cancer Center, Tokyo, Japan; 717 Genome Institute of Singapore, Singapore, Singapore; 718 32Program in Computational Biology and Bioinformatics, Yale University, New Haven, CT USA; 719 German Cancer Aid, Bonn, Germany; 720 Programme in Cancer and Stem Cell Biology, Centre for Computational Biology, Duke-NUS Medical School, Singapore, Singapore; 721 The Chinese University of Hong Kong, Shatin, NT, Hong Kong China; 722 Fourth Military Medical University, Shaanxi, China; 723 The University of Cambridge School of Clinical Medicine, Cambridge, UK; 724 St. Jude Children’s Research Hospital, Memphis, TN USA; 725 University Health Network, Princess Margaret Cancer Centre, Toronto, ON Canada; 726 Center for Biomolecular Science and Engineering, University of California Santa Cruz, Santa Cruz, CA USA; 727 Department of Medicine, University of Chicago, Chicago, IL USA; 728 Department of Neurology, Mayo Clinic, Rochester, MN USA; 729 Cambridge Oesophagogastric Centre, Cambridge University Hospitals NHS Foundation Trust, Cambridge, UK; 730 Department of Computer Science, Carleton College, Northfield, MN USA; 731 Institute of Cancer Sciences, College of Medical Veterinary and Life Sciences, University of Glasgow, Glasgow, UK; 732 Department of Epidemiology, University of Alabama at Birmingham, Birmingham, AL USA; 733 HudsonAlpha Institute for Biotechnology, Huntsville, AL USA; 734 O’Neal Comprehensive Cancer Center, University of Alabama at Birmingham, Birmingham, AL USA; 735 Department of Pathology, Keio University School of Medicine, Tokyo, Japan; 736 Department of Hepatobiliary and Pancreatic Oncology, National Cancer Center Hospital, Tokyo, Japan; 737 Sage Bionetworks, Seattle, WA USA; 738 Lymphoma Genomic Translational Research Laboratory, National Cancer Centre, Singapore, Singapore; 739 Department of Clinical Pathology, Robert-Bosch-Hospital, Stuttgart, Germany; 740 Department of Cell and Systems Biology, University of Toronto, Toronto, ON Canada; 741 Department of Biosciences and Nutrition, Karolinska Institutet, Stockholm, Sweden; 742 Center for Liver Cancer, Research Institute and Hospital, National Cancer Center, Gyeonggi, South Korea; 743 Division of Hematology-Oncology, Samsung Medical Center, Sungkyunkwan University School of Medicine, Seoul, South Korea; 744 Samsung Advanced Institute for Health Sciences and Technology, Sungkyunkwan University School of Medicine, Seoul, South Korea; 745 Cheonan Industry-Academic Collaboration Foundation, Sangmyung University, Cheonan, South Korea; 746 NYU Langone Medical Center, New York, NY USA; 747 Department of Hematology and Medical Oncology, Cleveland Clinic, Cleveland, OH USA; 748 Department of Radiation Oncology, University of California San Francisco, San Francisco, CA USA; 749 Department of Health Sciences Research, Mayo Clinic, Rochester, MN USA; 750 Helen F. Graham Cancer Center at Christiana Care Health Systems, Newark, DE USA; 751 Heidelberg University Hospital, Heidelberg, Germany; 752 CSRA Incorporated, Fairfax, VA USA; 753 Research Department of Pathology, University College London Cancer Institute, London, UK; 754 Department of Research Oncology, Guy’s Hospital, King’s Health Partners AHSC, King’s College London School of Medicine, London, UK; 755 Faculty of Medicine and Health Sciences, Macquarie University, Sydney, NSW Australia; 756 University Hospital of Minjoz, INSERM UMR 1098, Besançon, France; 757 Spanish National Cancer Research Centre, Madrid, Spain; 758 Center of Digestive Diseases and Liver Transplantation, Fundeni Clinical Institute, Bucharest, Romania; 759 Cureline, Inc, South San Francisco, CA USA; 760 St. Luke’s Cancer Centre, Royal Surrey County Hospital NHS Foundation Trust, Guildford, UK; 761 Cambridge Breast Unit, Addenbrooke’s Hospital, Cambridge University Hospital NHS Foundation Trust and NIHR Cambridge Biomedical Research Centre, Cambridge, UK; 762 East of Scotland Breast Service, Ninewells Hospital, Aberdeen, UK; 763 Department of Genetics, Microbiology and Statistics, University of Barcelona, IRSJD, IBUB, Barcelona, Spain; 764 Department of Obstetrics and Gynecology, Medical College of Wisconsin, Milwaukee, WI USA; 765 Hematology and Medical Oncology, Winship Cancer Institute of Emory University, Atlanta, GA USA; 766 Department of Computer Science, Princeton University, Princeton, NJ USA; 767 Vanderbilt Ingram Cancer Center, Vanderbilt University, Nashville, TN USA; 768 Ohio State University College of Medicine and Arthur G. James Comprehensive Cancer Center, Columbus, OH USA; 769 Department of Surgery, Yokohama City University Graduate School of Medicine, Kanagawa, Japan; 770 Division of Chromatin Networks, German Cancer Research Center (DKFZ) and BioQuant, Heidelberg, Germany; 771 Research Computing Center, University of North Carolina at Chapel Hill, Chapel Hill, NC USA; 772 School of Molecular Biosciences and Center for Reproductive Biology, Washington State University, Pullman, WA USA; 773 Finsen Laboratory and Biotech Research and Innovation Centre (BRIC), University of Copenhagen, Copenhagen, Denmark; 774 Department of Laboratory Medicine and Pathobiology, University of Toronto, Toronto, ON Canada; 775 Department of Pathology, Human Oncology and Pathogenesis Program, Memorial Sloan Kettering Cancer Center, New York, NY USA; 776 University Hospital Giessen, Pediatric Hematology and Oncology, Giessen, Germany; 777 Oncologie Sénologie, ICM Institut Régional du Cancer, Montpellier, France; 778 Institute of Clinical Molecular Biology, Christian-Albrechts-University, Kiel, Germany; 779 Institute of Pathology, University of Wuerzburg, Wuerzburg, Germany; 780 Department of Urology, North Bristol NHS Trust, Bristol, UK; 781 SingHealth, Duke-NUS Institute of Precision Medicine, National Heart Centre Singapore, Singapore, Singapore; 782 Department of Computer Science, University of Toronto, Toronto, ON Canada; 783 Bern Center for Precision Medicine, University Hospital of Bern, University of Bern, Bern, Switzerland; 784 Englander Institute for Precision Medicine, Weill Cornell Medicine and New York Presbyterian Hospital, New York, NY USA; 785 Meyer Cancer Center, Weill Cornell Medicine, New York, NY USA; 786 Pathology and Laboratory, Weill Cornell Medical College, New York, NY USA; 787 Vall d’Hebron Institute of Oncology: VHIO, Barcelona, Spain; 788 General and Hepatobiliary-Biliary Surgery, Pancreas Institute, University and Hospital Trust of Verona, Verona, Italy; 789 National Centre for Biological Sciences, Tata Institute of Fundamental Research, Bangalore, India; 790 Indiana University, Bloomington, IN USA; 791 Department of Pathology, GZA-ZNA Hospitals, Antwerp, Belgium; 792 Analytical Biological Services, Inc, Wilmington, DE USA; 793 Sydney Medical School, University of Sydney, Sydney, NSW Australia; 794 cBio Center, Dana-Farber Cancer Institute, Harvard Medical School, Boston, MA USA; 795 Department of Cell Biology, Harvard Medical School, Boston, MA USA; 796 Advanced Centre for Treatment Research and Education in Cancer, Tata Memorial Centre, Navi Mumbai, Maharashtra India; 797 School of Environmental and Life Sciences, Faculty of Science, The University of Newcastle, Ourimbah, NSW Australia; 798 Department of Dermatology, University Hospital of Essen, Essen, Germany; 799 Bioinformatics and Omics Data Analytics, German Cancer Research Center (DKFZ), Heidelberg, Germany; 800 Department of Urology, Charité Universitätsmedizin Berlin, Berlin, Germany; 801 Martini-Clinic, Prostate Cancer Center, University Medical Center Hamburg-Eppendorf, Hamburg, Germany; 802 Department of General Internal Medicine, University of Kiel, Kiel, Germany; 803 German Cancer Consortium (DKTK), Partner site Berlin, Berlin, Germany; 804 Cancer Research Institute, Beth Israel Deaconess Medical Center, Boston, MA USA; 805 University of Pittsburgh, Pittsburgh, PA USA; 806 Department of Ophthalmology and Ocular Genomics Institute, Massachusetts Eye and Ear, Harvard Medical School, Boston, MA USA; 807 Center for Psychiatric Genetics, NorthShore University HealthSystem, Evanston, IL USA; 808 Van Andel Research Institute, Grand Rapids, MI USA; 809 Laboratory of Molecular Medicine, Human Genome Center, Institute of Medical Science, University of Tokyo, Tokyo, Japan; 810 Japan Agency for Medical Research and Development, Tokyo, Japan; 811 Korea University, Seoul, South Korea; 812 Murtha Cancer Center, Walter Reed National Military Medical Center, Bethesda, MD USA; 813 Human Genetics, University of Kiel, Kiel, Germany; 814 Department of Oncologic Pathology, Dana-Farber Cancer Institute, Harvard Medical School, Boston, MA USA; 815 Oregon Health and Science University, Portland, OR USA; 816 Center for RNA Interference and Noncoding RNA, The University of Texas MD Anderson Cancer Center, Houston, TX USA; 817 Department of Experimental Therapeutics, The University of Texas MD Anderson Cancer Center, Houston, TX USA; 818 Department of Gynecologic Oncology and Reproductive Medicine, The University of Texas MD Anderson Cancer Center, Houston, TX USA; 819 University Hospitals Coventry and Warwickshire NHS Trust, Coventry, UK; 820 Department of Radiation Oncology, Radboud University Nijmegen Medical Centre, Nijmegen, GA The Netherlands; 821 Institute for Genomics and Systems Biology, University of Chicago, Chicago, IL USA; 822 Clinic for Hematology and Oncology, St.-Antonius-Hospital, Eschweiler, Germany; 823 Computational and Systems Biology Program, Memorial Sloan Kettering Cancer Center, New York, NY USA; 824 University of Iceland, Reykjavik, Iceland; 825 Division of Computational Genomics and Systems Genetics, German Cancer Research Center (DKFZ), Heidelberg, Germany; 826 Dundee Cancer Centre, Ninewells Hospital, Dundee, UK; 827 Department for Internal Medicine III, University of Ulm and University Hospital of Ulm, Ulm, Germany; 828 Institut Curie, INSERM Unit 830, Paris, France; 829 Department of Gastroenterology and Hepatology, Yokohama City University Graduate School of Medicine, Kanagawa, Japan; 830 Department of Laboratory Medicine, Radboud University Nijmegen Medical Centre, Nijmegen, GA The Netherlands; 831 Division of Cancer Genome Research, German Cancer Research Center (DKFZ), Heidelberg, Germany; 832 Department of General Surgery, Singapore General Hospital, Singapore, Singapore; 833 Cancer Science Institute of Singapore, National University of Singapore, Singapore, Singapore; 834 Department of Medical and Clinical Genetics, Genome-Scale Biology Research Program, University of Helsinki, Helsinki, Finland; 835 East Anglian Medical Genetics Service, Cambridge University Hospitals NHS Foundation Trust, Cambridge, UK; 836 Irving Institute for Cancer Dynamics, Columbia University, New York, NY USA; 837 Institute of Molecular and Cell Biology, Singapore, Singapore; 838 Laboratory of Cancer Epigenome, Division of Medical Science, National Cancer Centre Singapore, Singapore, Singapore; 839 Universite Lyon, INCa-Synergie, Centre Léon Bérard, Lyon, France; 840 Department of Urology, Mayo Clinic, Rochester, MN USA; 841 Royal National Orthopaedic Hospital - Stanmore, Stanmore, Middlesex UK; 842 Department of Biochemistry, Genetics and Immunology, University of Vigo, Vigo, Spain; 843 Giovanni Paolo II / I.R.C.C.S. Cancer Institute, Bari, BA Italy; 844 Neuroblastoma Genomics, German Cancer Research Center (DKFZ), Heidelberg, Germany; 845 Fondazione Policlinico Universitario Gemelli IRCCS, Rome, Italy, Rome, Italy; 846 University of Verona, Verona, Italy; 847 Centre National de Génotypage, CEA - Institute de Génomique, Evry, France; 848 CAPHRI Research School, Maastricht University, Maastricht, ER The Netherlands; 849 Department of Biopathology, Centre Léon Bérard, Lyon, France; 850 Université Claude Bernard Lyon 1, Villeurbanne, France; 851 Core Research for Evolutional Science and Technology (CREST), JST, Tokyo, Japan; 852 Department of Biological Sciences, Laboratory for Medical Science Mathematics, Graduate School of Science, University of Tokyo, Yokohama, Japan; 853 Department of Medical Science Mathematics, Medical Research Institute, Tokyo Medical and Dental University (TMDU), Tokyo, Japan; 854 Cancer Ageing and Somatic Mutation Programme, Wellcome Sanger Institute, Hinxton, UK; 855 University Hospitals Birmingham NHS Foundation Trust, Birmingham, UK; 856 Centre for Cancer Research and Cell Biology, Queen’s University, Belfast, UK; 857 Breast Medical Oncology, The University of Texas MD Anderson Cancer Center, Houston, TX USA; 858 Department of Surgery, Johns Hopkins University School of Medicine, Baltimore, MD USA; 859 Department of Oncology-Pathology, Science for Life Laboratory, Karolinska Institute, Stockholm, Sweden; 860 School of Cancer Sciences, Faculty of Medicine, University of Southampton, Southampton, UK; 861 Department of Gene Technology, Tallinn University of Technology, Tallinn, Estonia; 862 Genetics and Genome Biology Program, SickKids Research Institute, The Hospital for Sick Children, Toronto, ON Canada; 863 Departments of Neurosurgery and Hematology and Medical Oncology, Winship Cancer Institute and School of Medicine, Emory University, Atlanta, GA USA; 864 Department of Clinical and Molecular Medicine, Faculty of Medicine and Health Sciences, Norwegian University of Science and Technology, Trondheim, Norway; 865 Argmix Consulting, North Vancouver, BC Canada; 866 Department of Information Technology, Ghent University, Interuniversitair Micro-Electronica Centrum (IMEC), Ghent, Belgium; 867 Nuffield Department of Surgical Sciences, John Radcliffe Hospital, University of Oxford, Oxford, UK; 868 Institute of Mathematics and Computer Science, University of Latvia, Riga, LV Latvia; 869 Discipline of Pathology, Sydney Medical School, University of Sydney, Sydney, NSW Australia; 870 Department of Applied Mathematics and Theoretical Physics, Centre for Mathematical Sciences, University of Cambridge, Cambridge, UK; 871 Department of Epidemiology and Biostatistics, Memorial Sloan Kettering Cancer Center, New York, NY USA; 872 Department of Statistics, Columbia University, New York, NY USA; 873 Department of Immunology, Genetics and Pathology, Science for Life Laboratory, Uppsala University, Uppsala, Sweden; 874 School of Electronic and Information Engineering, Xi’an Jiaotong University, Xi’an, China; 875 Department of Histopathology, Cambridge University Hospitals NHS Foundation Trust, Cambridge, UK; 876 Oxford NIHR Biomedical Research Centre, University of Oxford, Oxford, UK; 877 Georgia Regents University Cancer Center, Augusta, GA USA; 878 Wythenshawe Hospital, Manchester, UK; 879 Department of Genetics, Washington University School of Medicine, St.Louis, MO USA; 880 Department of Biological Oceanography, Leibniz Institute of Baltic Sea Research, Rostock, Germany; 881 Wellcome Centre for Human Genetics, University of Oxford, Oxford, UK; 882 Department of Molecular and Human Genetics, Baylor College of Medicine, Houston, TX USA; 883 Thoracic Oncology Laboratory, Mayo Clinic, Rochester, MN USA; 884 Institute for Genomic Medicine, Nationwide Children’s Hospital, Columbus, OH USA; 885 Department of Obstetrics and Gynecology, Division of Gynecologic Oncology, Mayo Clinic, Rochester, MN USA; 886 International Institute for Molecular Oncology, Poznań, Poland; 887 Poznan University of Medical Sciences, Poznań, Poland; 888 Genomics and Proteomics Core Facility High Throughput Sequencing Unit, German Cancer Research Center (DKFZ), Heidelberg, Germany; 889 NCCS-VARI Translational Research Laboratory, National Cancer Centre Singapore, Singapore, Singapore; 890 Edison Family Center for Genome Sciences and Systems Biology, Washington University, St. Louis, MO USA; 891 MRC-University of Glasgow Centre for Virus Research, Glasgow, UK; 892 Department of Medical Informatics and Clinical Epidemiology, Division of Bioinformatics and Computational Biology, OHSU Knight Cancer Institute, Oregon Health and Science University, Portland, OR USA; 893 School of Electronic Information and Communications, Huazhong University of Science and Technology, Wuhan, China; 894 Department of Applied Mathematics and Statistics, Johns Hopkins University, Baltimore, MD USA; 895 Department of Cancer Genome Informatics, Graduate School of Medicine, Osaka University, Osaka, Japan; 896 Institute of Computer Science, Heidelberg University, Heidelberg, Germany; 897 School of Mathematics and Statistics, University of Sydney, Sydney, NSW Australia; 898 Ben May Department for Cancer Research, University of Chicago, Chicago, IL USA; 899 Department of Human Genetics, University of Chicago, Chicago, IL USA; 900 Tri-Institutional PhD Program in Computational Biology and Medicine, Weill Cornell Medicine, New York, NY USA; 901 The First Affiliated Hospital, Xi’an Jiaotong University, Xi’an, China; 902 Department of Medicine and Therapeutics, The Chinese University of Hong Kong, Shatin, NT, Hong Kong China; 903 Department of Biostatistics, The University of Texas MD Anderson Cancer Center, Houston, TX USA; 904 Duke-NUS Medical School, Singapore, Singapore; 905 Department of Surgery, Ruijin Hospital, Shanghai Jiaotong University School of Medicine, Shanghai, China; 906 School of Computing Science, University of Glasgow, Glasgow, UK; 907 Division of Orthopaedic Surgery, Oslo University Hospital, Oslo, Norway; 908 Eastern Clinical School, Monash University, Melbourne, VIC Australia; 909 Epworth HealthCare, Richmond, VIC Australia; 910 Department of Biostatistics and Computational Biology, Dana-Farber Cancer Institute and Harvard Medical School, Boston, MA USA; 911 Department of Biomedical Informatics, College of Medicine, The Ohio State University, Columbus, OH USA; 912 The Ohio State University Comprehensive Cancer Center (OSUCCC – James), Columbus, OH USA; 913 The University of Texas School of Biomedical Informatics (SBMI) at Houston, Houston, TX USA; 914 Department of Biostatistics, University of North Carolina at Chapel Hill, Chapel Hill, NC USA; 915 Department of Biochemistry and Molecular Genetics, Feinberg School of Medicine, Northwestern University, Chicago, IL USA; 916 Faculty of Medicine and Health, University of Sydney, Sydney, NSW Australia; 917 Department of Pathology, Erasmus Medical Center Rotterdam, Rotterdam, GD The Netherlands; 918 Division of Molecular Carcinogenesis, The Netherlands Cancer Institute, Amsterdam, CX The Netherlands; 919 Institute of Molecular Life Sciences and Swiss Institute of Bioinformatics, University of Zurich, Zurich, Switzerland

**Keywords:** Cancer genomics, Data integration

## Abstract

Transcript alterations often result from somatic changes in cancer genomes^1^. Various forms of RNA alterations have been described in cancer, including overexpression^2^, altered splicing^3^ and gene fusions^4^; however, it is difficult to attribute these to underlying genomic changes owing to heterogeneity among patients and tumour types, and the relatively small cohorts of patients for whom samples have been analysed by both transcriptome and whole-genome sequencing. Here we present, to our knowledge, the most comprehensive catalogue of cancer-associated gene alterations to date, obtained by characterizing tumour transcriptomes from 1,188 donors of the Pan-Cancer Analysis of Whole Genomes (PCAWG) Consortium of the International Cancer Genome Consortium (ICGC) and The Cancer Genome Atlas (TCGA)^5^. Using matched whole-genome sequencing data, we associated several categories of RNA alterations with germline and somatic DNA alterations, and identified probable genetic mechanisms. Somatic copy-number alterations were the major drivers of variations in total gene and allele-specific expression. We identified 649 associations of somatic single-nucleotide variants with gene expression in *cis*, of which 68.4% involved associations with flanking non-coding regions of the gene. We found 1,900 splicing alterations associated with somatic mutations, including the formation of exons within introns in proximity to Alu elements. In addition, 82% of gene fusions were associated with structural variants, including 75 of a new class, termed ‘bridged’ fusions, in which a third genomic location bridges two genes. We observed transcriptomic alteration signatures that differ between cancer types and have associations with variations in DNA mutational signatures. This compendium of RNA alterations in the genomic context provides a rich resource for identifying genes and mechanisms that are functionally implicated in cancer.

## Abstract (web summary)

Integrative analyses of transcriptome and whole-genome sequencing data for 1,188 tumours across 27 types of cancer are used to provide a comprehensive catalogue of RNA-level alterations in cancer.

## Main

**Extended Data Fig. 1:**
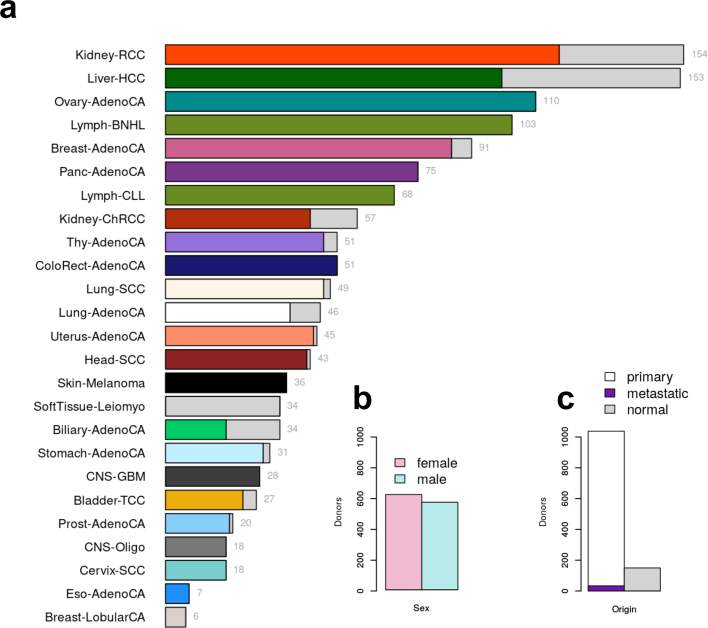
Pan-cancer expression profiling of 1,188 PCAWG donors. **a**, Tumour and normal RNA-seq data from 27 histotypes. The total number of samples is shown to the right of the bars. Grey bars denote matched healthy samples. **b**, Number of female versus male donors. **c**, Total number of tumour and matched healthy samples from the PCAWG study. A subset of tumours (dark violet) was metastatic.

For a more extensive study of cancer genome alterations, particularly in non-coding regions, the PCAWG project was formed to analyse the large number of whole-genome samples that were contributed to the ICGC and TCGA projects^5^. Individual projects did not use the same methods for key analyses; therefore, a major focus for each of the 16 PCAWG Working Groups was the unified analysis of the PCAWG data. For example, the PCAWG Technical Working Group led raw data collection, realignment of whole-genome sequencing data and implemented core somatic mutation calling pipelines^5^. Other PCAWG working groups focused on unified analyses of copy-number variation^6^, structural variants^7,8^, germline variants^5^, mutational signatures^9^ and identification of driver genes^8^, among others^5^. Here, we report the joint analysis of available matched transcriptome and genome profiling for 1,188 samples from 27 tumour types by the PCAWG Transcriptome Working Group^5^, providing the largest, to our knowledge, resource of RNA phenotypes and their underlying genetic changes in cancer so far (Extended Data Fig. 1, Methods, Supplementary Results, Supplementary Table 23). We demonstrate the importance of transcriptomics data in understanding how different dimensions of specific DNA alterations contribute to carcinogenesis and map out the landscape of cancer-related RNA alterations.

## Cancer-specific germline *cis*-eQTLs

**Extended Data Fig. 2:**
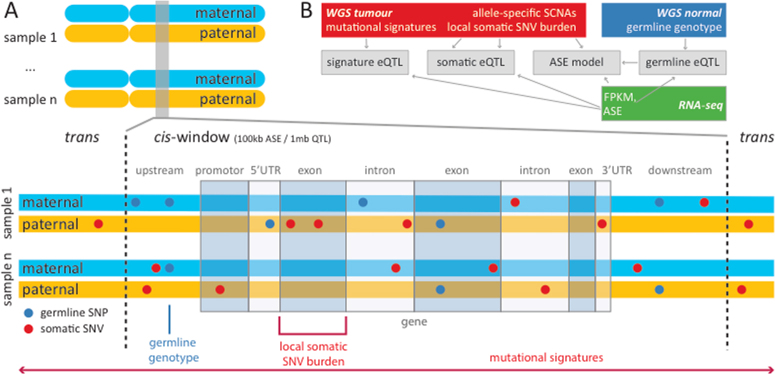
Overview of the different sources of genetic variation considered in the analysis. **a**, For analyses of *cis* regulation, mono-allelic single-nucleotide germline variants (single nucleotide polymorphisms (SNPs), blue) were individually tested for association with total gene expression using standard eQTL approaches. Owing to their low recurrence in the cohort, somatic SNVs were aggregated in burden categories depending on their position relative to the gene tested (for example, promoter, 5′ UTR or intron). Local SNV burdens were then tested for association with ASE globally across all genes, as well as with total expression on a per-gene level using eQTL approaches. *Trans* effects were estimated by testing total gene expression for association with mutational and epigenetic signatures. Window sizes were 1 Mb for all somatic *cis*-eQTL analyses, and 100 kb for ASE and germline *cis*-eQTL. **b**, Overview of the different datasets and their contributions to the analyses described in **a**. Germline genotypes were derived from the matched healthy whole-genome sequencing (WGS) samples. Allele-specific SCNAs, mutational signatures and local SNV burdens were derived from the tumour WGS in comparison to the unaffected WGS samples. ASE and total expression (FPKM) were derived from the tumour and normal RNA-seq data. Arrows indicate dependencies between individual analyses carried out.

**Extended Data Fig. 3:**
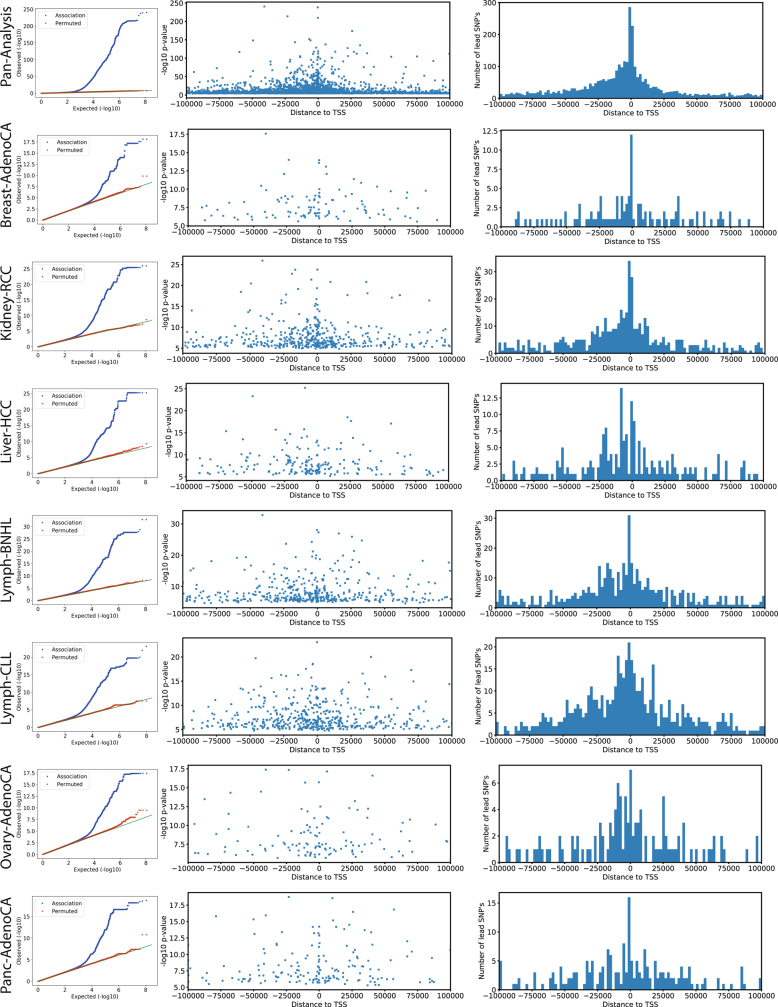
Germline eQTL lead variants. Left, quantile–quantile (Q–Q) plot of *P* values of germline eQTL lead variants in the pan-cancer and histotype-specific analysis (FDR ≤ 5%, blue) and *P* values of the same analysis after permutation (random permutation of patients, red). Middle and right, distributions of distance to the respective TSS of all germline eQTL lead variants in the pan-cancer and histotype-specific analysis.

To investigate the underlying mechanisms of different types of RNA alteration, we first focused on changes in the gene expression level (Extended Data Fig. 2). We initially considered common germline variants (minor allele frequency ≥ 1%) proximal to individual genes (±100 kb), and mapped expression quantitative trait loci (eQTL) across the cohort (Extended Data Fig. 3, Supplementary Table 1). This pan-cancer analysis identified 3,532 genes with an eQTL (false discovery rate (FDR) ≤ 5%, hereafter denoted eGenes) (Supplementary Table 2), enriched in proximal regions of transcription start sites (TSSs) (Extended Data Fig. 3).

**Fig. 1:**
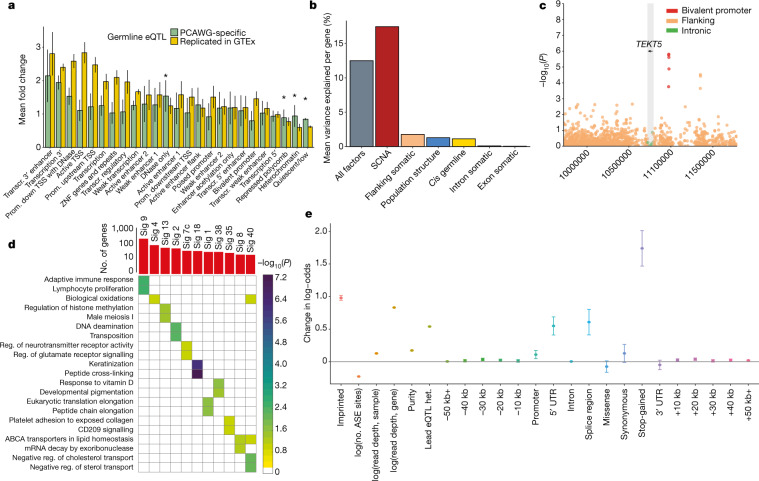
Germline and somatic SNVs associated with expression. **a**, Epigenetics Roadmap enrichment analysis, showing the average fold change in Roadmap factors across cell lines in PCAWG-specific eQTLs of the pan-analysis as well as eQTLs that replicate in GTEx tissues. *P < 0.05/25, one-sided Wilcoxon rank-sum test in PCAWG-specific eQTLs corrected for the number of Roadmap factors used (that is, 25). Data are mean and s.d. **b**, Variance component analysis for gene expression levels, showing the average proportion of variance explained by different germline and somatic factors for different sets of genes including the mean effect across all factors: (1) all genetic factors (germline and somatic); (2) SCNAs; (3) somatic variants in flanking regions; (4) population structure; (5) *cis*-germline effects; and (6) somatic intron and exon mutation effects. **c**, Manhattan plot showing nominal *P* values of association for *TEKT5* (highlighted in grey), considering flanking, intronic and exonic intervals. The leading somatic burden is associated with increased *TEKT5* expression (*P* = 1.61 × 10^−6^) and overlaps an upstream bivalent promoter (red dots; annotated in 81 Roadmap cell lines, including 8 embryonic stem cells, 9 embryonic-stem-cell-derived and 5 induced pluripotent stem-cell lines). **d**, Summary of significant associations between mutational signatures (Sig) and gene expression. Top, the total number of associated genes per signature (FDR ≤ 10%). Bottom, enriched GO categories or Reactome pathways for genes associated with each signature (FDR ≤ 10%, significance level encoded in colour, −log_10_-transformed adjusted *P* value). **e**, Standardized effect sizes on the presence of AEI, taking only SCNAs, germline eQTLs, coding and non-coding mutations into account. Data are the estimate and standard error of the estimate of the effect size.

**Extended Data Fig. 4:**
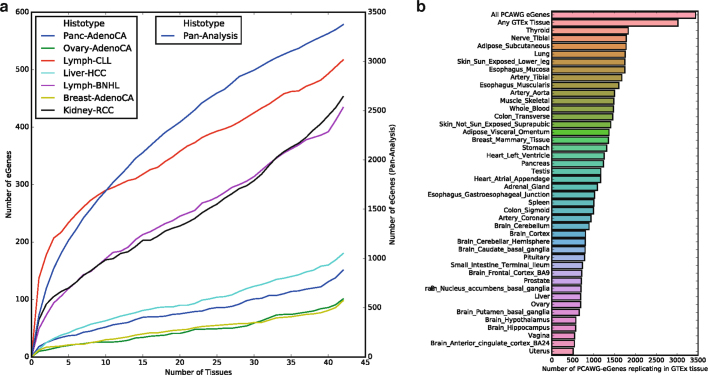
PCAWG-specific eGenes. **a**, Number of PCAWG-specific eGenes in relation to eQTL replication in various numbers of GTEx tissues. **b**, Number of eGenes of the PCAWG pan-analysis replicating in corresponding GTEx tissues.

To identify cancer-specific regulatory variants, we compared our eQTLs to eQTLs from the Genotype-Tissue Expression (GTEx) project^10^, adopting previous strategies to assess eQTL replication^11^, and probed lead eQTL variants for marginal significance in GTEx tissues (*P* ≤ 0.01, Bonferroni-adjusted). Although most lead variants could be detected in GTEx samples (3,110 out of 3,532 eQTL variants), we identified 422 eQTLs that did not correspond to GTEx tissues, which suggests cancer-specific regulation (Extended Data Fig. 4, Supplementary Table 3). The corresponding eQTL lead variants were enriched for heterochromatic regions (Fig. 1a). Overall, this analysis revealed that the germline framework of gene expression regulation is largely conserved in cancer tissues.

## Somatic *cis*-eQTLs in non-coding regions

**Extended Data Fig. 5:**
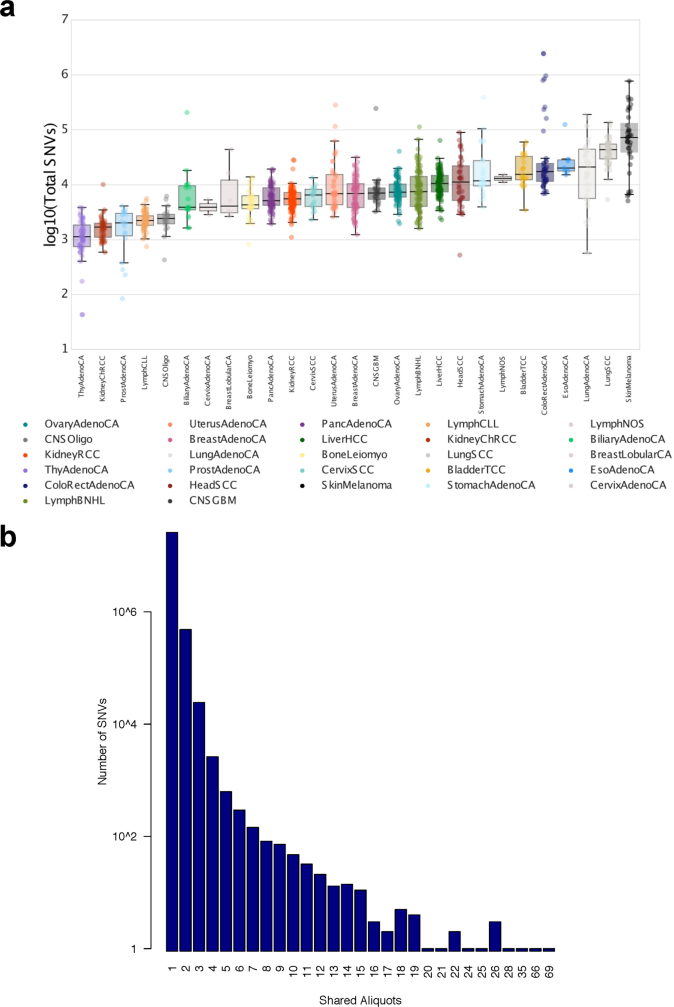
*Cis*-mutational somatic burden. **a**, Total number of somatic mutational load per cancer type. Median numbers of SNVs range from 1,139 in thyroid adenocarcinoma to 72,804 in skin melanoma. **b**, Number of recurrent somatic SNVs shared by increasing numbers of patients. A small fraction of 86 SNVs is detected in more than 1% of the cohort (12 patients).

**Extended Data Fig. 6:**
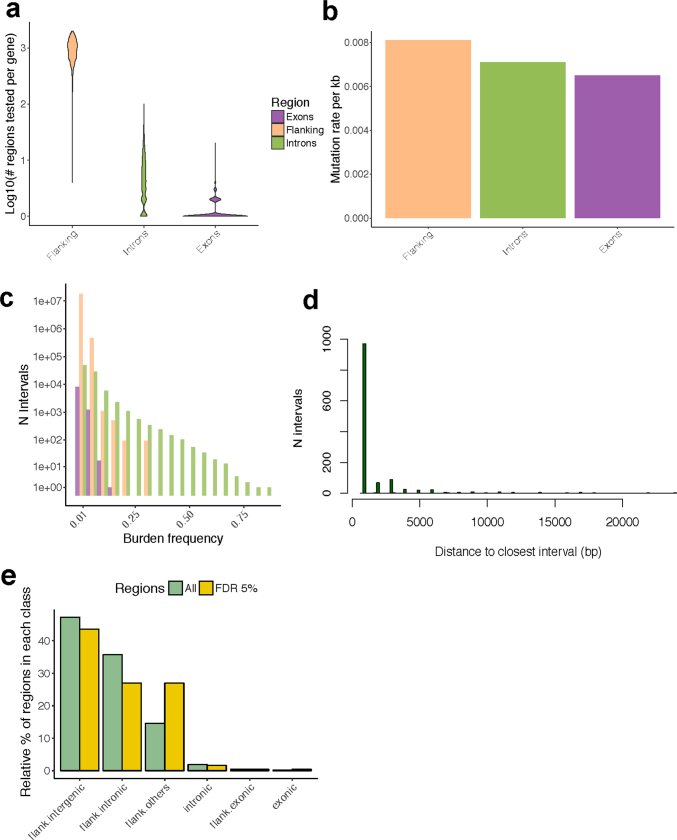
Somatic mutation rate and burden frequency by type of region tested. **a**, Number of mutated regions tested per gene with somatic burden frequency ≥ 1%. **b**, Mutation rate per kilobase. **c**, Burden frequency, stratified by the type of interval tested (flanking, exonic or intronic). **d**, Distribution of distances (bp) of the leading intervals (FDR ≤ 5%) to the closest (left and right) interval such that the association *P* value decreases by at least one order of magnitude (99% of the distribution is shown). **e**, Breakdown of all genomic regions tested (*n* = 1,049,102 with burden frequency ≥ 1%) and of the 567 genomic regions that underlie the observed somatic *cis*-eQTL at a FDR of 5% (intronic denotes eGene intron; exonic denotes eGene exon; flank. denotes 2-kb flanking region within 1 Mb distance to the eGene start and end; flank.intergenic denotes flanking region in a genomic location without gene annotations; flank.intronic denotes flanking region overlapping an intron of a nearby gene; and flank.others denotes flanking region partially overlapping several annotations of a nearby gene).

Previous studies have described the landscape of non-coding mutations in cancer^1^, particularly in promoter regions, and also their regulatory effects on gene expression^12,13^. Here, we looked at possible somatic DNA changes, across the whole genome, that underlie alterations in gene expression. We estimated local mutation burdens by aggregating single-nucleotide variants (SNVs) in 2-kb intervals adjacent to genes (flanking), as well as in exons and introns (Extended Data Figs. 2, 5, 6). Next, we decomposed the expression variation of individual genes, considering common mutation burdens in *cis*, as well as *cis* germline variants and somatic copy-number alterations (SCNAs). This identified SCNAs as the major driver of expression variation (17%), followed by somatic SNVs in gene flanking regions (1.8%) and germline variants (1.3%) (Fig. 1b).

**Extended Data Fig. 7:**
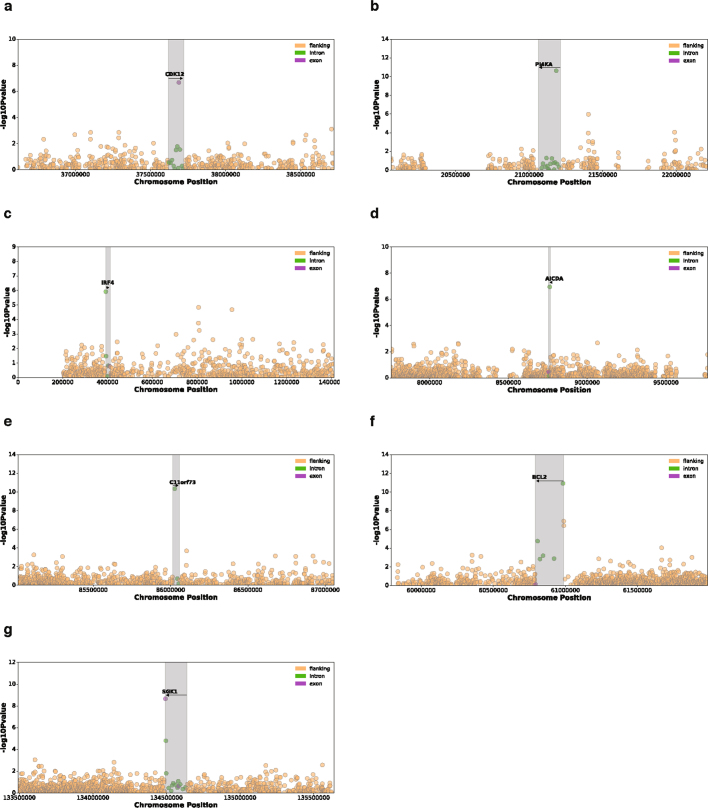
Manhattan plots of seven somatic eGenes associated with genic lead burden. Altogether, 11 genic somatic eQTLs showed significant changes in gene expression associated with somatic burdens within the gene boundaries (intronic or exonic). The seven genes shown here are known to be important in the pathogenesis of specific cancers. **a**, *CDK12*. **b**, *PI4KA*. **c**, *IRF4*. **d**, *AICDA*. **e**, *C11orf73* (also known as *HIKESHI*). **f**, *BCL2*. **g**, *SGK1*.

**Extended Data Fig. 8:**
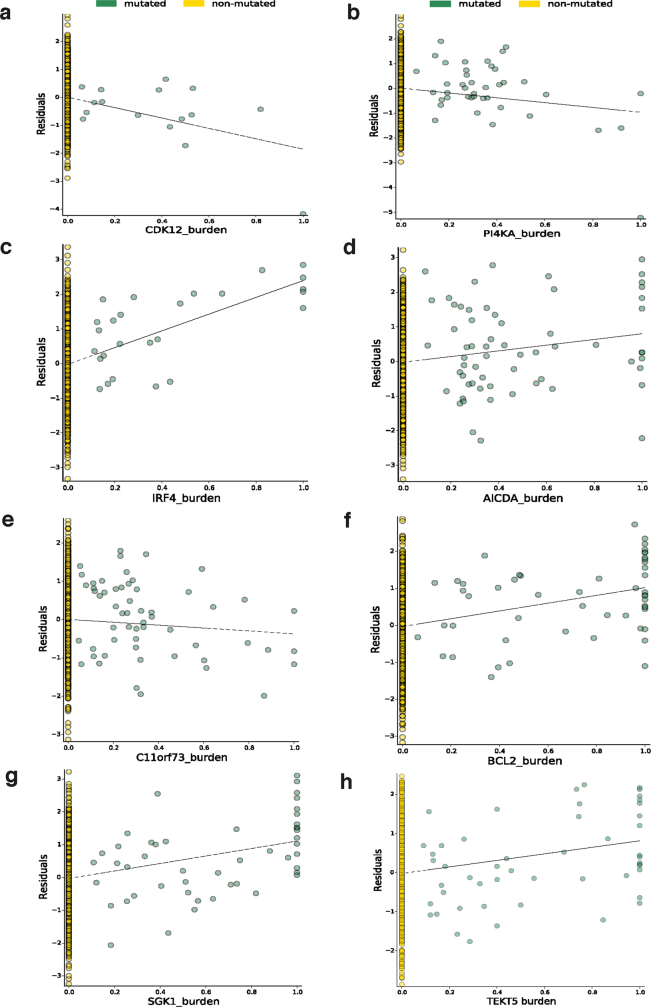
Scatter plots of eight somatic eGenes. Plots show the effect of the lead weighted burden on the gene expression residuals (obtained as described in the Methods) of these genes. **a**, *CDK12*. **b**, *PI4KA*. **c**, *IRF4*. **d**, *AICDA*. **e**, *C11orf73*. **f**, *BCL2*. **g**, *SGK1*. **h**, *TEKT5*.

**Extended Data Fig. 9:**
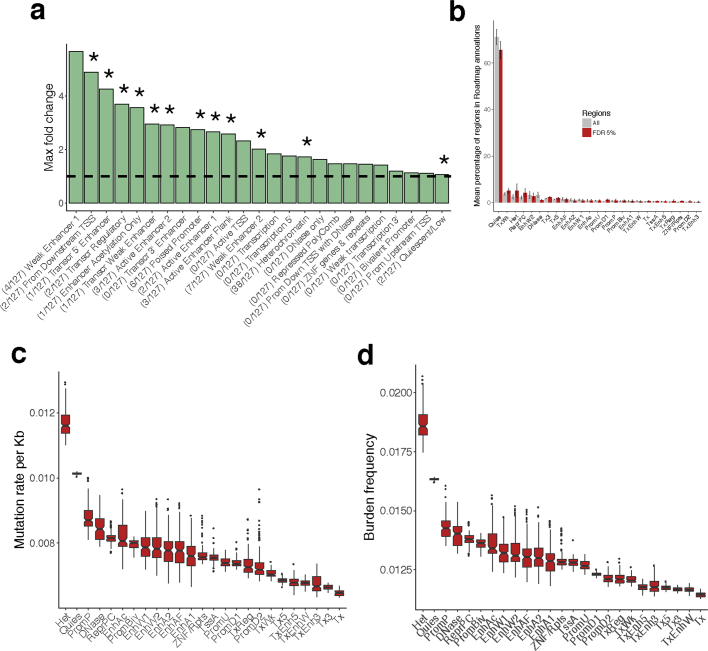
Roadmap epigenome marks overlapping flanking intervals with somatic burden. **a**, Maximum fold enrichment of epigenetic marks from the Roadmap Epigenomics Project across 127 cell lines. The number of cell lines with significant enrichments is indicated in parentheses (FDR ≤ 10%); asterisks denote significant enrichments in at least one cell line. **b**, Mean percentages (over the 127 cell lines) of regions overlapping (by at least 10% of their length) Roadmap epigenome marks, calculated using all genomic flanking regions (*n* = 1,637,638) and the subset of 556 flanking intervals associated with somatic eQTL (FDR ≤ 5%). **c**, Mutation rate per kilobase. **d**, Burden frequency (across the 127 cell lines) of the 556 flanking intervals in somatic eQTLs (FDR ≤ 5%), overlapping 25 Roadmap epigenome marks. DNase, DNase only; EnhA, active enhancer; EnhAc, enhancer acetylation only; EnhAF, active enhancer flank; EnhW, weak enhancer; Het, heterochromatin; PromBiv, bivalent promoters; PromD, promoter downstream; PromP, poised promoters; PromU, promoter upstream; Quies, quiescent/low; ReprPC, repressed PolyComb; TssA, active TSS; TxReg, transcription regulatory; ZNF/Rpts, ZNF genes and repeats; Tx, transcription; Tx3, transcription 3′, Tx5, transcription 5′; TxEnh3, transcription 3′ enhancer; TxEnh5, transcription 5′ enhancer; TxEnhW, transcription weak enhancer; TxWk, weak transcription.

We also tested for associations between all common mutation burdens and gene expression across the whole genome. We identified 649 genes with a somatic eQTL (FDR ≤ 5%) (Supplementary Table 5). Of these, 11 associations were located in introns or exons of the respective eGene, including genes with known roles in the pathogenesis of specific cancers such as *CDK12* in ovarian cancer^14^ and *IRF4* in chronic lymphocytic leukaemia^15^ (Extended Data Figs. 7, 8). Most eQTLs (68.4%) involved associations with flanking non-coding mutation burdens (Extended Data Fig. 6e). Next, we considered eQTLs in flanking regions (*n* = 556) and tested for enrichment in cell-type-specific annotations from the Epigenetics Roadmap^16^. This identified 13 enriched annotations (FDR ≤ 10%) (Extended Data Fig. 9, Supplementary Table 6), including poised promoters, weak and active enhancers, and heterochromatin, but notably no enrichment for transcription-factor-binding sites (Supplementary Table 7). This enrichment in transcriptionally inactive regions may be due to an increased mutation rate in these regions (Extended Data Fig. 9), which has previously been reported in cancer^17^.

We also looked at the functional characterization of somatic eGenes and observed an enrichment for somatic eQTLs in bivalent promoters for cancer testis genes (*P* = 0.04, Fisher’s exact test) such as *TEKT5*^18^ (Fig. 1c, Extended Data Fig. 8h). Furthermore, we found a global enrichment (FDR ≤ 10%) for Gene Ontology (GO) categories related to cell differentiation and developmental processes (Supplementary Table 8). Overall, somatic eQTL analysis identified mostly non-coding regions associated with changes in local gene expression and, similar to cancer-specific germline eQTLs, showed enrichment for transcriptionally inactive regions such as heterochromatin.

## Expression and mutational signatures

Global variations in mutational patterns can be quantified using mutational signatures, which tag mutational processes specific to their tissue-of-origin and environmental exposures^19^. However, the extraction of mutational signatures is an intrinsically statistical process that requires a posteriori functional annotation. We performed a pan-cancer association analysis between genome-wide mutational signatures and gene expression levels to decipher the molecular processes that accompany the presence of mutational signatures.

**Extended Data Fig. 10:**
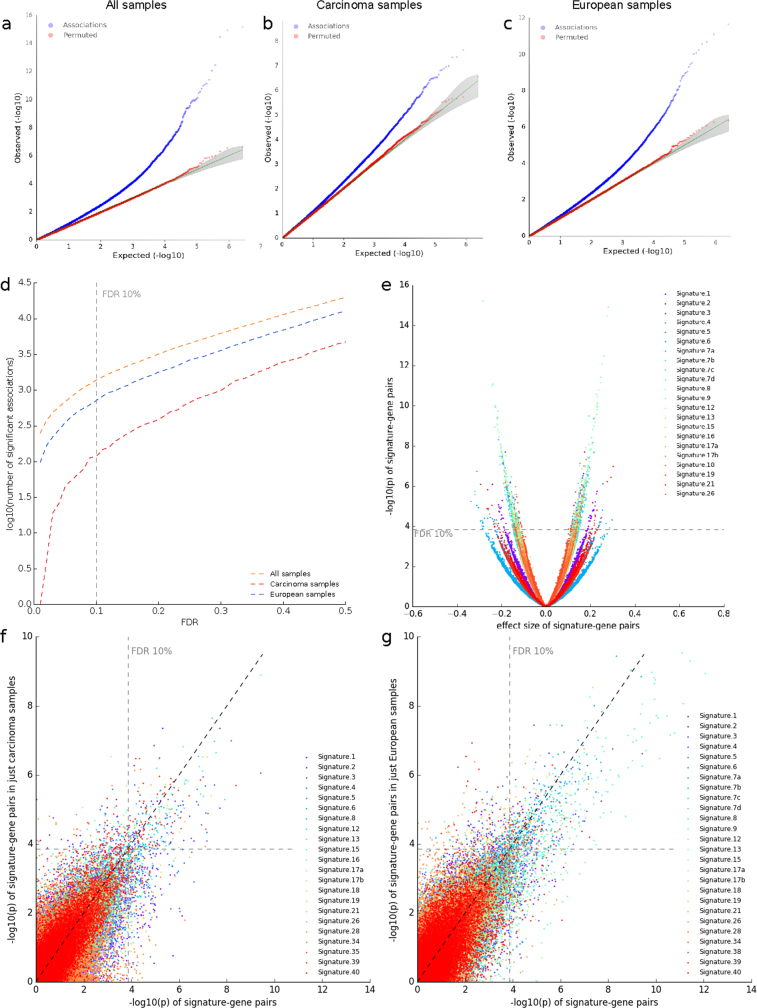
Quality control of the association studies between gene expression and mutational signatures. **a**–**c**, Q–Q plots of the *P* values of the linear model to associate expression of 18,831 genes with 28 mutational signatures across all 1,159 patients (**a**), 877 patients with carcinoma (**b**), or 891 European patients (**c**). **d**, Number of significant associations (log_10_-transformed) at different FDR thresholds (across all patients, patients with carcinoma and European patients). **e**, Volcano plot of directionality of effects in the analysis of all patients. **f**, **g**, Comparison of analyses between all patients and patients with carcinoma (**f**) and between all patients and European patients (**g**). The −log_10_(*P* values) per signature–gene pair are correlated (*r* = 0.763 (**f**) and *r* = 0.789 (**g**), Pearson correlation coefficient), especially above an FDR threshold of 10%.

We considered 28 mutational signatures derived using non-negative matrix factorization of context-specific mutation frequencies^9^. We tested for association between signature prevalence in donors and total gene expression, accounting for total mutational burden, cancer type, and other technical and biological confounders. This identified 1,176 genes associated with at least one signature (FDR ≤ 10%) (Extended Data Fig. 10, Supplementary Table 19).

**Extended Data Fig. 11:**
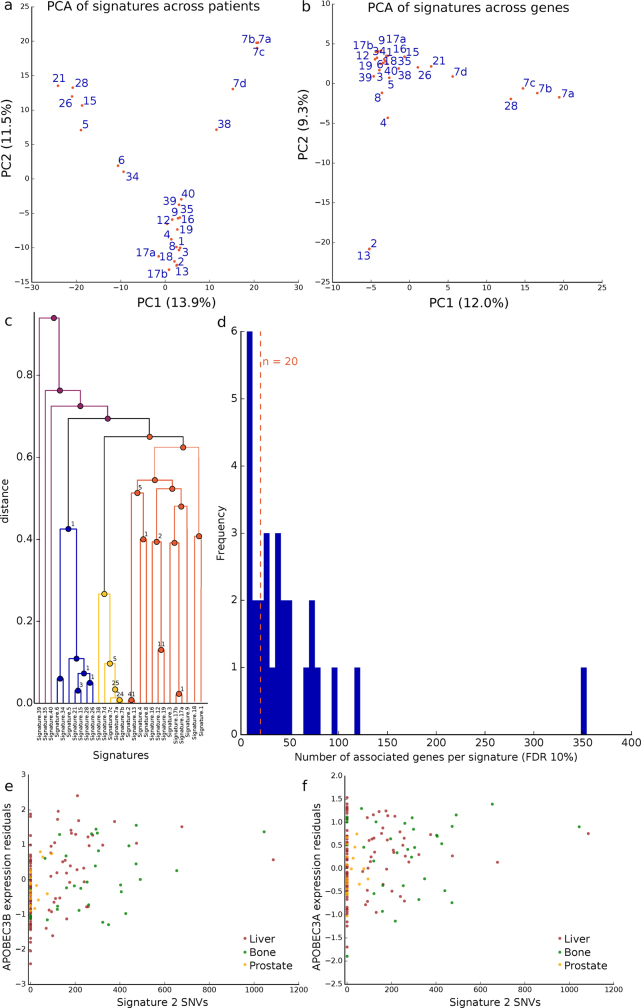
Relationship between mutational signatures and gene expression patterns. **a**, **b**, Principal component analysis (PCA) of signatures across 1,159 patients (PCA on signature-specific SNVs per patient) (**a**) and signature–gene expression associations across 18,831 genes (PCA on adjusted *P* values of signature–gene expression associations) (**b**). The PCA on the SNVs recapitulates known interdependencies, for example, between signatures 7, whereas the PCA on the signature–gene association studies also emphasizes functional relatedness, for example, between signatures 2 and 13. **c**, Hierarchical clustering of signatures. The numbers at the nodes indicate the number of genes commonly associated with two to four respective signatures. The dendrogram shows genes that are associated with more than one signature mostly owing to similar SNV patterns of these signatures across patients. **d**, Frequency of number of significantly associated genes per signature (FDR ≤ 10%). Although many signatures are significantly associated with a few genes, 18 signatures are associated with more than 20 genes. Signature 9 is associated with more than 350 genes. Vice versa, 1,009 genes are associated with only one signature, 129 with two, 32 with three, 5 with four and 1 with five signatures. **e**, **f**, Mutational signature–gene associations, depicting positive associations between the expression of the canonical APOBEC pathway genes *APOBEC3B* (**e**) and *APOBEC3A* (**f**) and signature 2. The associations within the three cancer type with the strongest correlation between signature and gene expression (hepatocellular carcinoma (Liver–HCC), bone leiomyosarcoma (Bone–Leiomyo) and prostate adenocarcinoma (Prost–AdenoCA)) are shown.

We considered 18 signatures with 20 or more associated genes for further annotation (Extended Data Fig. 11) and assessed enrichment using GO categories^20^ and Reactome pathways^21^. We found that 11 signatures were enriched for at least one category (FDR ≤ 10%) (Supplementary Table 19), revealing associations consistent with known and unknown aetiologies (Fig. 1d). For example, signature 38, which is correlated with the canonical UV signature 7 (*r*^2^ = 0.375, *P* = 5 × 10^−40^) (Extended Data Fig. 11c), was linked to melanin processes (Fig. 1d). The synthesis of melanin causes oxidative stress to melanocytes^22^, and we found signature 38 associated with the oxidative-stress-promoting gene *TYR*^23^ (*P* = 1.0 × 10^−4^). A hallmark of signature 38 genes are C>A mutations, a typical product of reactive oxygen species^24^. This suggests that signature 38 may capture DNA damage that is indirectly caused by UV-induced oxidative damage after direct sun exposure^25^, with *TYR* as a possible mediator of the effect.

## Genomic basis of allelic expression

**Extended Data Fig. 12:**
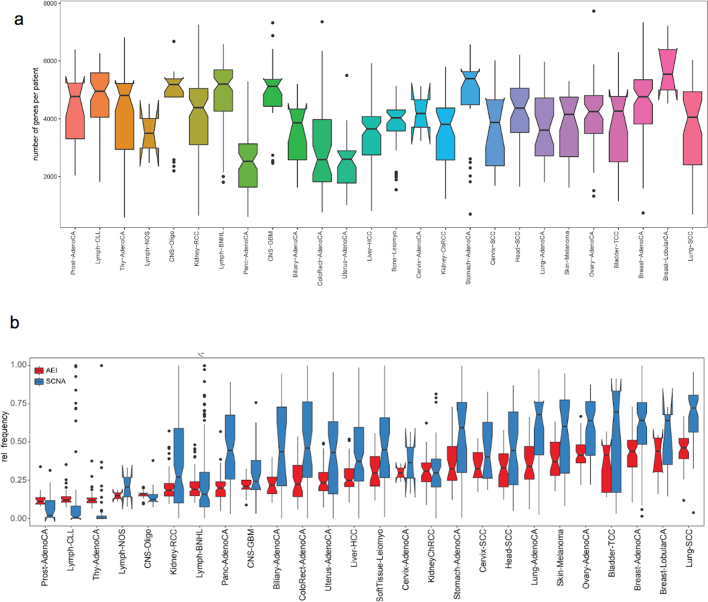
ASE analysis. **a**, All types of cancer are ordered by the average AEI frequency. The numbers of genes per patient for which ASE could be quantified are shown, stratified according to cancer type, resulting in between 588 and 7,728 genes per patient. **b**, Distribution of the fraction of genes with AEI (red) and SCNAs (blue) over the number of measurable genes for each patient across the cohort. Cancer types with high chromosomal instability also exhibit highest amounts of AEI.

**Extended Data Fig. 13:**
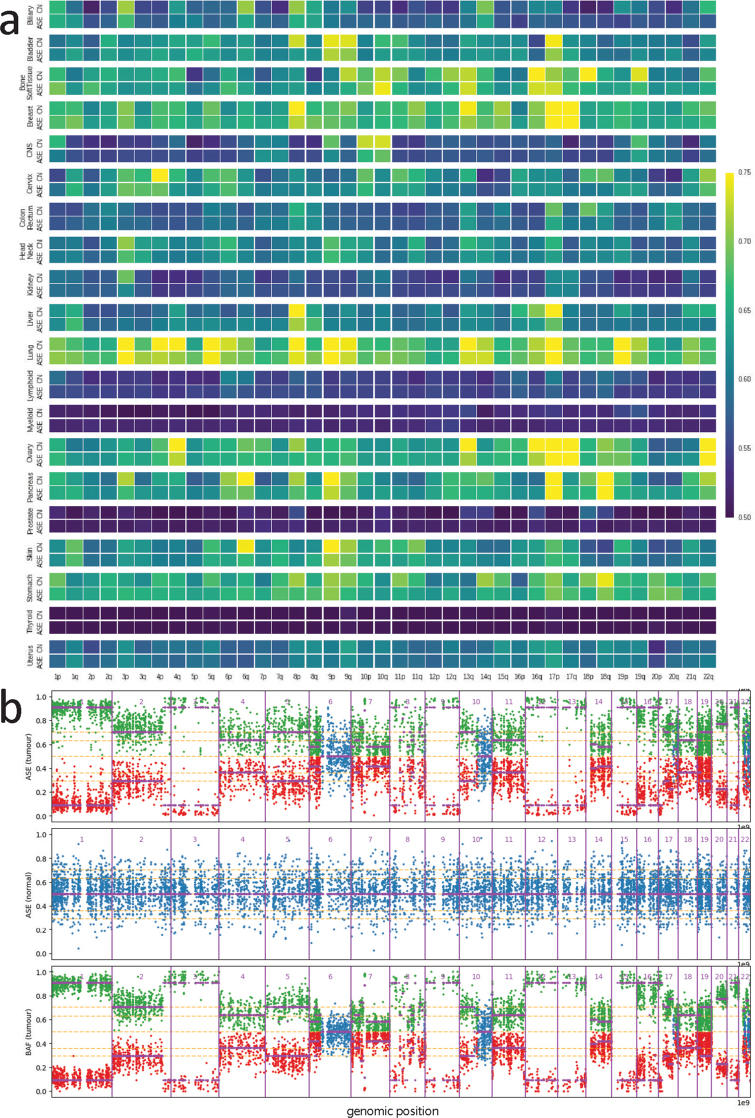
SCNAs as major driver for allelic dysregulation in cancer. **a**, Absolute allelic expression imbalance closely follows allelic imbalance at the genomic level. Values of 0.5 (blue) denote equal number of reads from both alleles. Values of 1 (yellow) reflect mono-allelic expression or regions with loss of heterozygosity. **b**, Comparison between B-allele frequency (BAF) and ASE ratios from a single patient with lung cancer (LUAD-US) with profound chromosomal instability shows strong correlation between allelic imbalance on expression and genomic levels.

To analyse expression at the level of individual haplotypes, we tested for allelic expression imbalance (AEI) (FDR ≤ 5%, binomial test). We observed substantial differences in the fraction of genes with AEI between different types of cancer (Extended Data Fig. 12), and between cancer and the corresponding healthy tissues, with a high observed concordance between allelic imbalance at the DNA and RNA levels (Extended Data Fig. 13).

**Extended Data Fig. 14:**
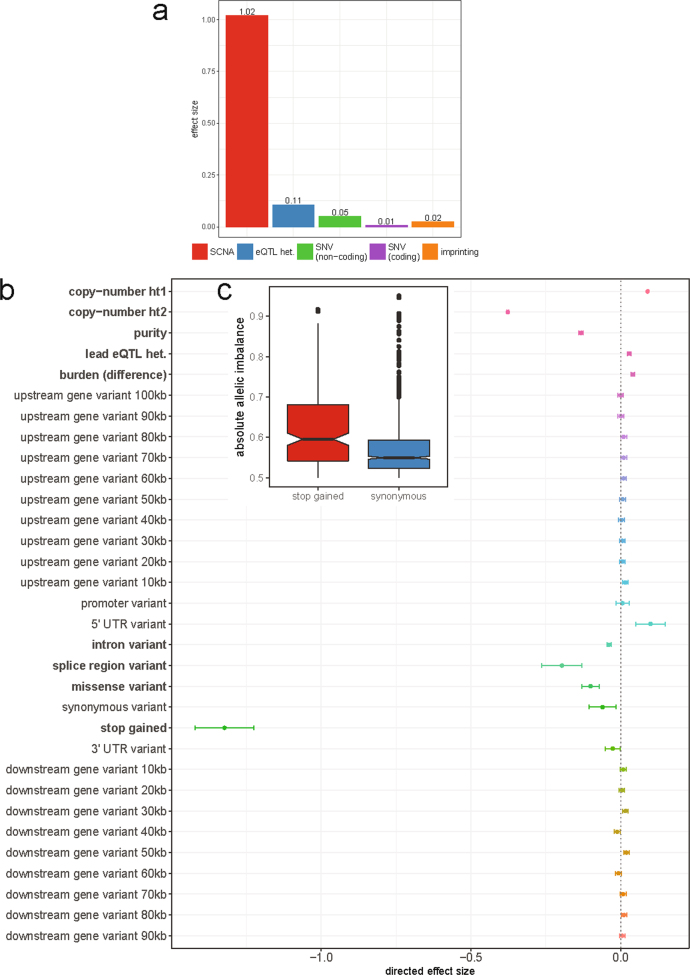
Determinants of AEI. **a**, Standardized effect sizes on the presence of AEI, taking only SCNAs, germline eQTLs, coding and non-coding mutations into account. In summary, SCNAs accounted for 86.1% of the total effect size, followed by germline eQTLs (9.0%) and somatic SNVs (4.8%). **b**, Relevance of individual somatic mutation types (‘copy-number ht1’ and ‘copy-number ht2’ as local allele-specific SCNAs of haplotypes 1 and 2, respectively), germline eQTLs and other covariates for the ASE ratio. Significant covariates (FDR ≤ 5%) are highlighted in bold. **c**, Comparison of the effect of protein-truncating variants (stop-gained) and synonymous variants on the ASE ratio.

We used a logistic regression model to identify the determinants of AEI, accounting for known imprinting status^26^, the germline eQTL genotype, SCNAs and the weighted mutational burden of proximal somatic SNVs stratified into functional categories (Extended Data Fig. 2). In aggregate, SCNAs accounted for 84.3% of the total explained variation, which confirmed our findings from the somatic eQTL analysis, followed by germline eQTL lead variants (9.1%), somatic SNVs (4.9%) and imprinting status (1.7%) (Extended Data Fig. 14). Although cumulatively, non-coding variants were more relevant than coding variants, somatic protein-truncating variants (‘stop-gained’ variants) that triggered nonsense-mediated decay^27^ were the most predictive individually. SNVs within splice regions, 5′ untranslated regions (UTRs) and promoters were also strongly associated with the presence of AEI, and we observed a global trend of decreasing relevance of variants with increasing distance from the TSS (Fig. 1e, Extended Data Fig. 14).

Gene-centric attribution of AEI to individual sources of genetic variation (Supplementary Table 9) revealed an enrichment of somatically induced AEI in several known cancer-driver genes, as well as new candidates, such as the mismatch-repair-related gene *EXO1* that is associated with survival in colorectal adenocarcinoma (log-rank *P* = 0.022, hazard ratio = 0.57) (Supplementary Results). We further observed a strong enrichment in the AEI score of cancer testis genes based on somatic SNVs only (*χ*^2^ test *P* = 6 × 10^−3^). In summary, we identify somatic and germline genetic variation that is associated with allele-specific dysregulation of genes across cancer types.

## Mutations associated with promoter usage

**Extended Data Fig. 15:**
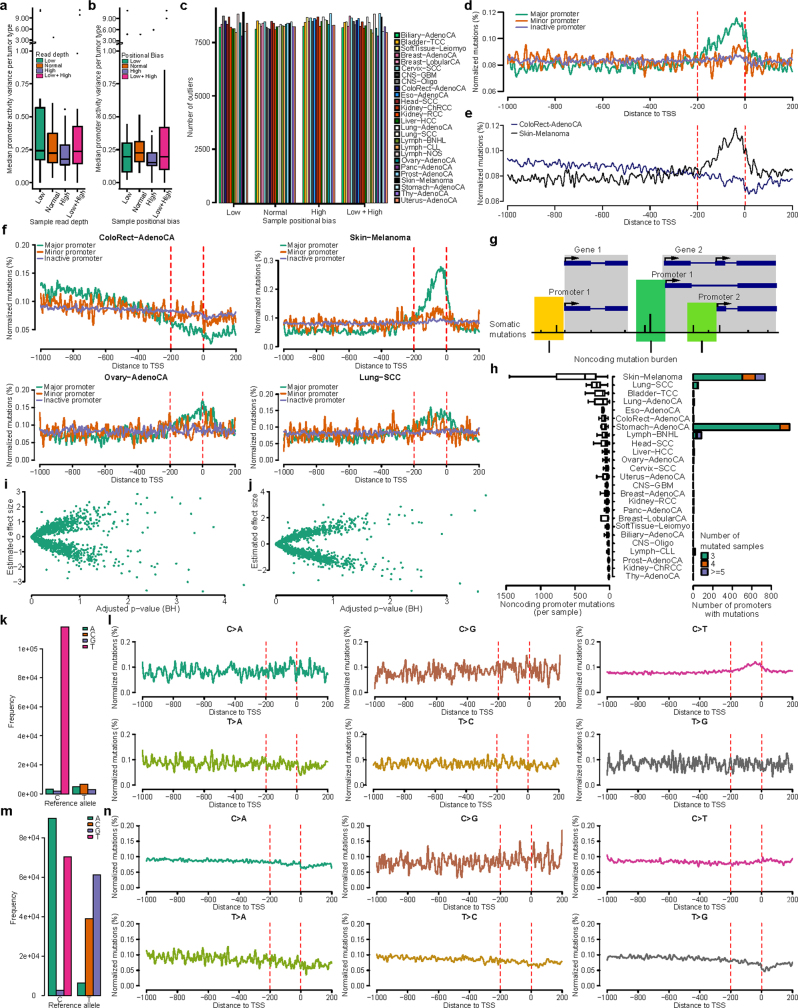
Overview of estimations of promoter activity and non-coding promoter mutations associations and patterns. **a**, **b**, The technical variation of the promoter activity estimates across varying library depth (**a**) and positional bias (**b**). **c**, The number of outlier promoters per tumour type according to promoter activity variance (variance larger than 1.5 × the interquartile range). **d**, Distribution of promoter mutations around promoters across the PCAWG cohort for major, minor and inactive promoters. Red lines indicate the window 200-bp upstream of a TSS, in which major promoters show an enrichment of mutations whereas minor and inactive promoters do not. **e**, Distribution of promoter mutations around promoters for the top two most mutated types of cancer (skin melanoma and colorectal adenocarcinoma (ColoRect–AdenoCA)). Colorectal adenocarcinoma displays a very different mutational pattern from other types of cancer. **f**, Distribution of promoter mutations around major, minor and inactive promoters across several types of cancer. Red lines indicate the window 200-bp upstream of a TSS, in which major promoters show an enrichment of mutations whereas minor and inactive promoters do not. **g**, Schematic of the calculation of non-coding promoter mutational burden. **h**, Overview of non-coding promoter mutations per sample and the number of mutated promoters per tumour type for promoters with at least three mutated samples. **i**, **j**, Association of absolute (**i**) and relative (**j**) promoter activity with promoter mutations across all samples. **k**, **l**, Overview of promoter mutations for skin melanoma tumours. **k**, Most promoter mutations are C>T, which indicates UV-induced DNA damage. **l**, Distribution of promoter mutations for each mutation class reveals the enrichment of C>T mutations around the 200-bp window upstream. **m**, **n**, Overview of promoter mutations for colorectal adenocarcinoma tumours. **m**, Most promoter mutations are C>A and C>T. **n**, Distribution of promoter mutations for each mutation class does not display an enrichment of mutations around the 200-bp window upstream, differing from the mutation pattern of skin melanoma tumours.

**Extended Data Fig. 16:**
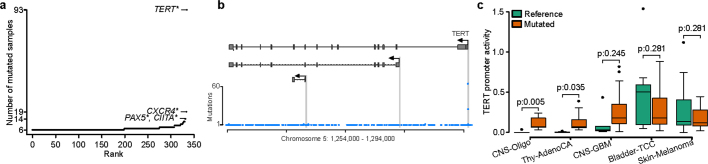
*TERT* promoter mutations. **a**, Promoters ranked by the number of mutated samples across all types of cancer in a 200-bp window. Asterisk indicates cancer census genes. **b**, The *TERT* locus and number of mutations observed at each position. The first promoter shows a highly recurrent non-coding mutation reported previously^118,119^. **c**, Comparison of *TERT* promoter activity for mutated and non-mutated samples per tumour type.

We considered promoter activity^28–30^ as another molecular phenotype to study the effect of promoter mutations. Although cancer-specific alternative promoter usage has previously been shown^28^, the association of underlying genomic alterations with promoter activities have not been broadly explored. To estimate the activity of individual gene promoters, we combined the expression of isoforms initiated in TSSs that are identical or nearby, assuming that these are transcribed from the same promoter (Extended Data Fig. 15a–c). We divided promoters into three categories: (1) inactive promoters (activity < 1 fragment per kilobase of transcript per million mapped reads (FPKM)), (2) major promoters (most active per gene) and (3) minor (all remaining) promoters, and examined the rates of mutation across varying activity levels. We observed an increase in the number of mutations near the TSS of major promoters compared with minor or inactive promoters (Extended Data Fig. 15d). This pattern is most prominent in skin melanoma, in which it has been attributed to impaired nucleotide-excision repair (Extended Data Fig. 15e, f, k, l). The cancer type that shows the strongest deviation from this pattern is colorectal adenocarcinoma, which highlights the tissue-specificity of mutational patterns at promoters (Extended Data Fig. 15e, f, m, n). Only 171 promoters show mutations in more than 5 samples per tumour type in a 200-bp window upstream of the promoter (Extended Data Fig. 15g, h). Most mutations occur in skin melanoma and lymphoma, which is expected owing to reduced nucleotide-excision repair and activation-induced cytidine deaminase (Extended Data Fig. 15h). We did not find significant pan-cancer associations between promoter mutational burden and promoter activity (Extended Data Fig. 15i, j). However, *TERT* has the highest number of promoter mutations^1,5,31^ (Extended Data Fig. 16a), and these mutations have previously been reported to be associated with *TERT* expression^1^; therefore, we investigated the *TERT* locus in more detail (Extended Data Fig. 16b). Although *TERT* does not show a significant association in the pan-cancer analysis, we found an association with increased promoter activity in individual types of cancer^1^ (Extended Data Fig. 16c).

## Mutations associated with splicing

**Fig. 2:**
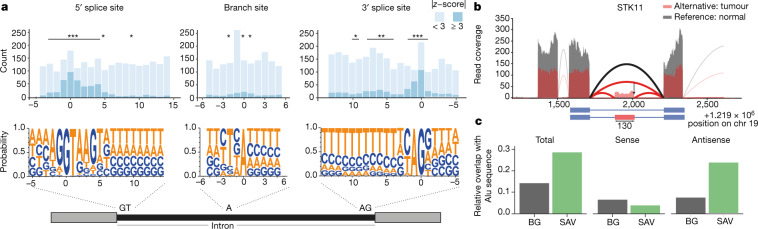
Position-specific effect of somatic mutations on alternative splicing. **a**, Top, proportion of mutations near exon–intron junctions and at branch sites that are associated with exon-skipping events. Mutations with associated splicing changes are those in which the percentage spliced in-derived |*z*-score| is ≥ 3 (dark blue). Asterisks denote intron positions significantly enriched for splicing changes relative to background based on a permutation test. *P < 0.05, **P < 0.01, ***P < 0.001. Bottom, sequence motifs of regions. **b**, Example of an exonization event in the tumour-suppressor gene *STK11*. The RNA-seq read coverage for a part of the gene is shown in red for a donor carrying the alternative allele, and in grey for a random donor with reference allele. The cassette exon event is shown as a schematic below. **c**, Enrichment of SINE elements in SAVs compared to sequence background (BG). Shown for SINE elements overlapping in sense (middle) and antisense (right) directions.

**Extended Data Fig. 17:**
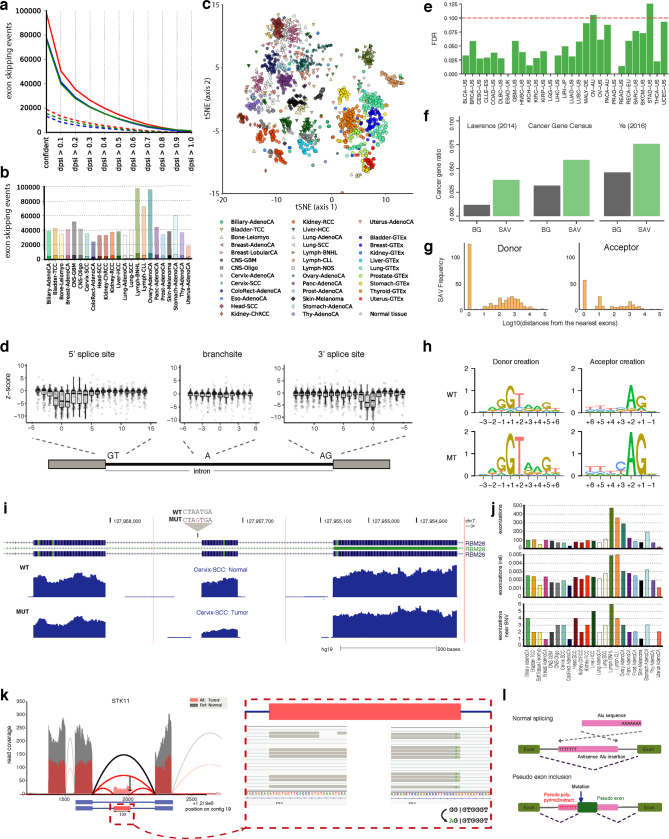
Alternative splicing and association with somatic mutations. **a**, Number of exon-skipping events confirmed at different ΔPSI thresholds in tumour (red), matched healthy (green) and GTEx (blue) samples for liver tissue. Dashed lines show the subset of exon-skipping events that only contain annotated introns. **b**, Number of exon-skipping events confirmed at a ΔPSI level of greater than 0.3 for the individual histotypes. Transparent section of bars represents the fraction of novel events, containing at least one unannotated intron. **c**, Splicing landscape for exon-skipping events. *t*-SNE analysis based on exon-skipping PSI values for all ICGC tumour and healthy samples together with tissue-matched GTEx samples. **d**, Position-specific effect of somatic mutations on alternative splicing. Magnitude and direction of mutation-associated splicing alterations. **e**, Permutation-based FDR values for SAV detection based on the different types of cancer. **f**, Cancer gene set enrichment for SAV sets, shown for cancer census gene set (middle) and sets determined in ref. ^48^ (left) and ref. ^120^ (right). **g**, Positional distributions (logarithms of distance from the nearest exons) of somatic variant creating novel splicing donors and acceptors. **h**, Sequence motif logos around somatic mutation creating novel splicing motifs. **i**, Example splicing effect of a branch-point mutation. UCSC genome browser RNA-seq coverage plots of cassette exon event in *RBM28* between mutant and wild type. Mutant (bottom track) contains an A>G mutation 29 nucleotides upstream from the acceptor site of an affected exon. **j**, Distribution of new cassette exon events detected only within the PCAWG cohort. Top, number of events per histology type. Middle, events normalized to the total number of cassette exons detected in the histology types. Bottom, the number of exonization events per histotype for the subset with the novel cassette exons colocated to a somatic alteration near the acceptor or donor of the exon. **k**, Example of an exonization event in the tumour-suppressor gene *STK11*. RNA-seq read coverage for a part of the gene is shown in red for a donor carrying the alternate allele and in grey for a random donor with reference allele. The cassette exon event is shown as a schematic below, with blue (red) boxes denoting constitutive (alternative) exons and blue solid lines denoting introns. Magnified panels at the bottom show details from Integrative Genomics Viewer visualization, highlighting a somatic mutation at the 3′ end of the cassette exon. The associated sequencing change is illustrated on the bottom right corner, in which the vertical bar denotes the exon–intron boundary. **l**, Alu-based exonization mechanism. Top, the presence of an Alu element in an intron in antisense alone will still result in normal splicing. Bottom, specific mutations of the Alu sequence creates new splice sites and results in exonization.

Extending the classical hallmarks of cancer, alternative splicing is seen as increasingly relevant to explain cancer heterogeneity^32^. On the basis of our observations of a globally changing splicing landscape (Extended Data Fig. 17a–c), we sought to specifically understand the relationship between splicing changes and somatic mutations within introns. Focusing on cassette exon events, we integrated the quantification of splice events with somatic variants and identified 5,282 mutations near exon–intron boundaries, 1,800 (34%) of which were associated with a change in splicing (|*z*-score| ≥ 3) (Supplementary Table 10). Consistent with previous findings using exome sequencing^33,34^, most mutations overlapping the essential dinucleotide motifs of the acceptor or donor site are associated with a splicing change—61% or 57%, respectively (Fig. 2a). Nearly one-third of all mutations (226 out of 469) in a 5-nucleotide window downstream of the 5′ site were significantly enriched for splicing changes (Fig. 2a). Almost all changes significantly associated with somatic mutations had a negative effect on splicing (96%) (Extended Data Fig. 17d). For mutations in or near the poly-pyrimidine tract, we found a significant (permutation test, *P* < 0.05) enrichment for mutations linked to outlier splicing (Fig. 2a). We also found an enrichment (*P* < 0.05, fold change > 2) of splicing outliers at branch-site adenosines (Fig. 2a middle, Extended Data Fig. 17d, Supplementary Table 11). Together, these results suggest that somatic mutations in the extended splice site region, poly-pyrimidine tract and branch point can affect splicing.

We also identified 1,900 rare splicing-associated variants (SAVs) that appear in only a small number of samples using the SAVNet approach^35^ (Extended Data Fig. 17e; see ‘Data availability’ in the Methods). Notably, 862 SAVs affected canonical splice sites, whereas the other 1,038 disrupted non-canonical sites or created new splice sites. Notably, we find a twofold enrichment of cancer genes in SAVs (Extended Data Fig. 17f).

Although we find that those SAVs that create splice sites strongly concentrate near exon–intron boundaries (Extended Data Fig. 17g), 45.9% of SAVs are further than 100 bp away from the nearest annotated exon. Mutations at those sites generally changed the sequences towards the donor or acceptor motif consensus (Extended Data Fig. 17h). Focusing on novel splice sites deep in introns, we analysed the extent of exonizations—that is, the formation of new exons within an intron (Extended Data Fig. 17j, Supplementary Tables 13, 14). More than one-fifth of these new exons (9 out of 43) occur in cancer-related genes, such as the well-known tumour-suppressor gene *STK11*. As expected, the exonization event would cause a frameshift in *STK11* (Fig. 2b, Extended Data Fig. 17k).

Alu elements that are inserted in an antisense direction have sequences that resemble consensus splice sites that, together with activating mutations, can lead to the formation of a new exon^36^ (Extended Data Fig. 17l). We found a significant enrichment of splice-site-creating SAVs within annotated Alu sequences (*P* = 2.8 × 10^−9^), particularly in the antisense direction (*P* = 2.6 × 10^−15^) (Fig. 2c). Our results indicate that the exonization of Alu sequences, which has been extensively studied in the context of primate genome evolution, is also observed in cancer genome evolution.

## Patterns of gene fusions across cancer

**Fig. 3:**
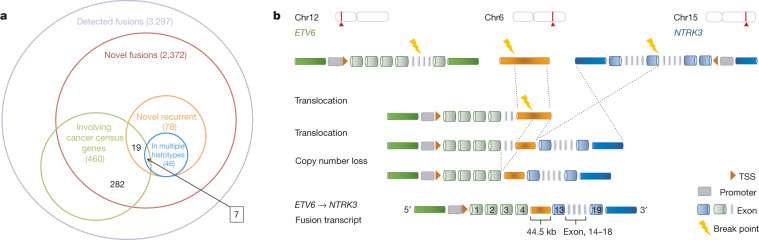
Structural rearrangements associated with RNA fusions. **a**, The number of all detected and new fusions and their overlap with the cancer census genes. **b**, Schematic of an example of bridged fusions. Bridged fusions are those composite fusions formed by a third genomic segment that bridges two genes. Only one of the possible orders of genomic arrangement is depicted in each case, with break points highlighted as thunderbolts.

Gene fusions are an important class of cancer-driving event with therapeutic and diagnostic value^37^. We identified a total of 925 known and 2,372 new cancer-specific gene fusions by combining the output of two fusion discovery methods as well as genomic rearrangement (structural variants) information and excluding artefacts or fusions in non-cancer samples^38^ (Fig. 3a). For the 3,540 identified fusion events representing 3,297 unique gene fusions, we categorized them on the basis of novelty, recurrence and known oncogenic gene partners (Fig. 3a).

**Extended Data Fig. 18:**
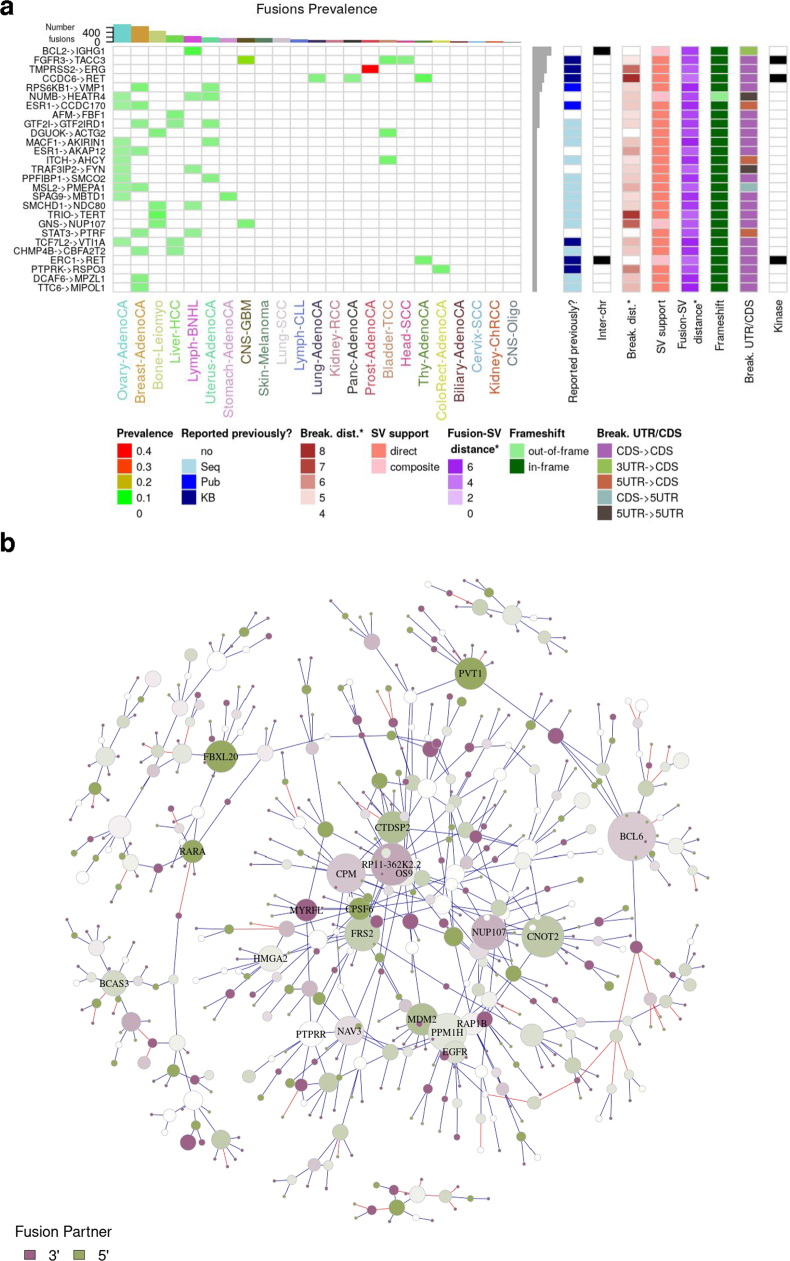
Recurrent and promiscuous RNA fusions. **a**, Features of the 27 most recurrent in-frame or open-reading-frame-retaining fusions. Kinase column indicates whether one of the gene partners is a kinase gene **b**, Network with connected clusters of at least 10 genes. Genes are represented as nodes, and the size of a node is proportional to the number of gene-fusion partners. Two nodes are connected if one fusion was detected involving the two genes: an edge is coloured blue if the fusion has evidence for matched structural rearrangements and is coloured red otherwise. Nodes and connections are shown only between promiscuous genes. The colour intensity indicates whether a gene is involved more often in a fusion as a 3′ (purple) or 5′ (green) gene or both (white).

Only 149 (approximately 5%) of the fusions occur in more than one sample, among which 78 are novel. Most of these (46 out of 78) were found across several histotypes. Of the 27 most recurrent gene fusions (Extended Data Fig. 18a), 8 have previously been reported (for example, *CCDC6-RET*^39^, *FGFR3-TACC3*^40^ and *PTPRK-RSPO3*) or independently detected in the TCGA cohort^41^, whereas 6 were new (such as *NUMB-HEATR4*, *ESR1-AKAP12* and *TRAF3IP2-FYN*). In total, 105 fusion transcripts involved the UTR region of one gene and the complete coding sequences of another gene, possibly resulting from structural variation in promoter regions.

Although most genes involved in fusions engaged with only one fusion partner, 35 genes had more than 5 partners. These ‘promiscuous’ genes tended to be selective in being either a 5′ or a 3′ partner with conserved break points and positions (3′ or 5′), and were overrepresented in cancer census genes and the PCAWG cancer-driver genes (one-tailed Fisher’s exact test, odds ratio = 8.66, *P* ≤ 1.1 × 10^−15^, and odds ratio = 12.27, *P* ≤ 2.2 × 10^−16^, respectively). Network analysis of promiscuous genes and their partners revealed several large gene clusters containing at least 10 genes (Extended Data Fig. 18b), enriched in cancer-related pathways (Benjamini–Hochberg corrected *P* ≤ 0.01) and in protein–protein interactions (*P* ≤ 1.0 × 10^−7^), which suggests a possible functional role in cancer.

Notably, a large number of fusions, including known fusions, could not be associated with only a single structural-variation event. For example, the *ETV6-NTRK3* gene fusion^42^ was present in a head and neck thyroid carcinoma sample, linking exon 4 of *ETV6* to exon 12 of *NTRK3*. We found three separate structural variants in the same sample: (1) a translocation of *ETV6* to chromosome 6; (2) a translocation of *NTRK3* also to chromosome 6; and (3) an additional copy-number loss spanning from intron 5 of *ETV6* to the exact structural variant break points, jointly bringing *ETV6* within 45 kb upstream of *NTRK3*—a distance that would allow transcriptional read-through^43^ or splicing^44^ to yield the *ETV6-NTRK3* fusion^45^ (Fig. 3b). Thus, the short chromosome-6 segment appeared to function as a bridge, which linked two genomic locations to facilitate a gene fusion. We term such products ‘bridged fusions’. This class of fusion is not uncommon. Out of a total of 436 gene fusions supported by 2 separate structural variants, 75 are bridged fusions (Supplementary Table 15).

**Extended Data Fig. 19:**
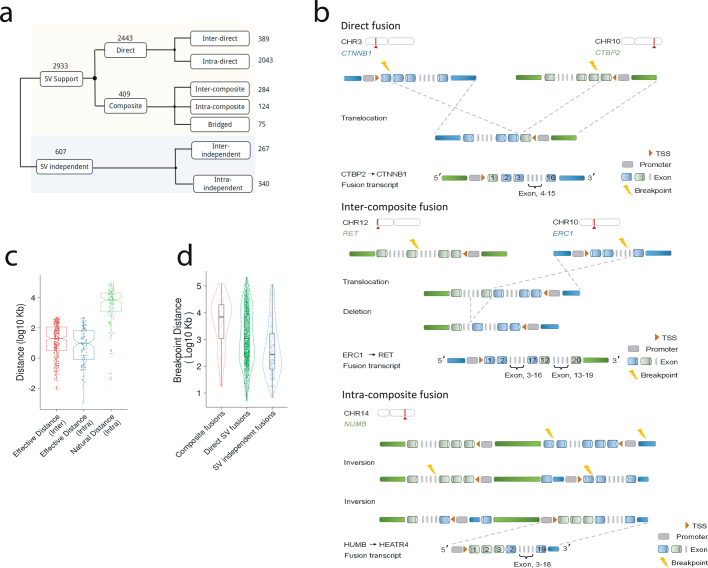
Structural rearrangements associated with RNA fusions. **a**, Systematic classification scheme of all gene fusions based on underlying structural variants (SVs). Numbers of fusion events of different classes are shown to the right. **b**, Schematic of examples of different types of structural-variant-supported fusions: (1) direct fusions; (2) intercomposite fusions; and (3) intracomposite fusions. Bridged fusions are shown in Fig. 3b. Only one of the possible orders of genomic arrangement is depicted in each case, with break points highlighted by thunderbolts. **c**, Supported rearrangements for composite fusions bring the fused segments of two genes significantly closer. Natural distance indicates the native distance between two related structural variant break points. Effective distance indicates the distance between the final two break points of the intra- and intercomposite fusions. **d**, The break points of structural-variant-independent fusions are typically closer than those for other interchromosomal fusions, which indicates that at least some of the structural-variant-independent fusions may occur directly at the RNA level, mediated either by *trans*-splicing or read-through events.

On the basis of the nature of the underlying genomic rearrangements, we propose a unified fusion classification system (Extended Data Fig. 19a). Aside from bridged fusions, 344 additional fusions are linked to more than one structural variant in the same sample. These multi- structural variant fusions are collectively termed ‘composite fusions’ (Extended Data Fig. 19a, b). We find 284 intercomposite fusions (interchromosomal translocation) and 124 intracomposite fusions (intrachromosomal rearrangement), exemplified by *ERC1-RET1* and *NUMB-HEATR4* fusions, respectively (Extended Data Fig. 19b). Composite rearrangements bring the fusion partners significantly closer to each other, from the median natural distance of 6.8 Mb to the median of 7.9 kb (Wilcoxon rank-sum test, *P* ≤ 2.2 × 10^−16^; Extended Data Fig. 19c) after translocation. For 18% of fusions, no evidence of structural variation was found. Given that 340 structural-variant-independent, intrachromosomal fusions had significantly closer break points than those with structural variation (Extended Data Fig. 19d), it is possible that they could result from RNA read-through events. The other possibility is that the underlying supporting structural variants escaped detection, as shown by the observation that known gene fusions that are driven by structural variation, such as *TMPRSS2-ERG*^46^, did not have consistent evidence for structural variation in matching samples.

## Landscape of RNA alterations in cancer

**Extended Data Fig. 20:**
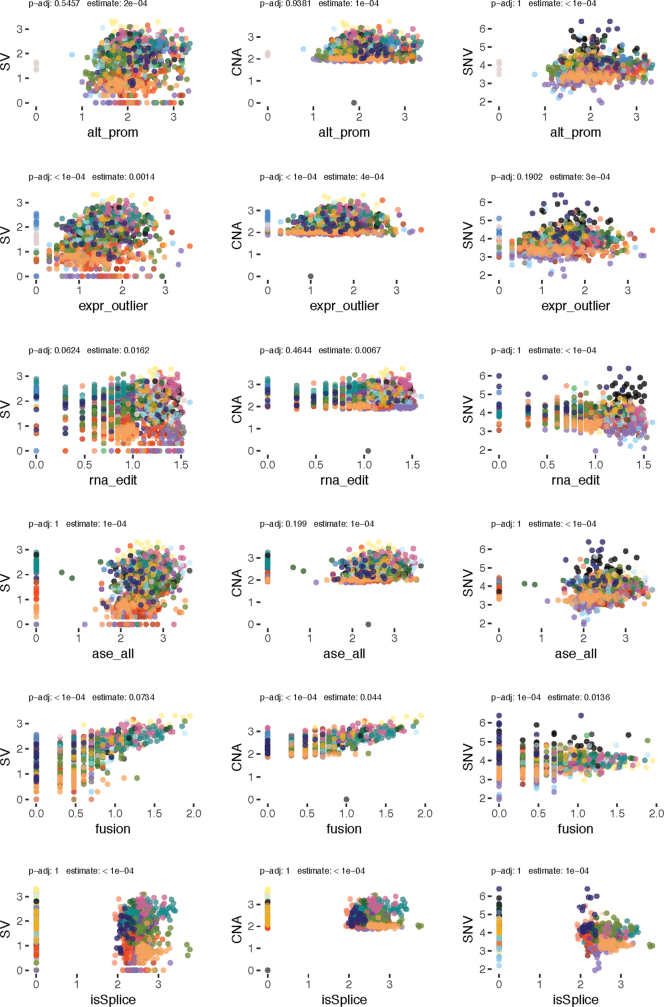
Correlation of the number of somatic genomic alterations with RNA alterations. Scatter plots of log_10_-transformed frequency of DNA alterations versus log_10_-transformed frequency of RNA alterations, in which each row is a DNA alteration in the following order: structural variants, copy-number aberrations and non-synonymous variants. Each row is an RNA alteration in the following order: expression outliers, RNA editing, ASE, fusions and splicing. Each point is a sample coloured by histotype, and its position is the log-transformed number of aberrations found in each sample. The Benjamini–Hochberg-adjusted *P* values are calculated from a likelihood ratio test assuming negative binomial distribution; histotype is used as a confounder.

Given our comprehensive set of RNA alterations, we sought to characterize the heterogeneous mechanisms of cancer genome and transcriptome alterations. To enable joint analyses of RNA and DNA alterations, we created a gene-level table, which indicates the presence or absence of possible functional changes to RNA or DNA for each gene and donor. After stringent filtering, we identified 1,523,098 alteration events, in which an event is a gene–sample–alteration triplet (Extended Data Table 1, Supplementary Table 14). It should be noted that we chose to include only RNA alterations with potential functional effects or with the strongest quantitative affect, resembling similar strategies for filtering DNA alterations^47^. Recurrence analysis across several alteration types helped us to further enrich for functionally relevant genes. Building on the gene-centric table, we characterized gene alterations at the RNA level and contrasted these with DNA alterations (non-synonymous SNVs or SCNAs)^5^. On the basis of the calculated association between each RNA- and DNA-level alteration across all histotypes, we found that half of the RNA alterations significantly correlated with DNA alterations (likelihood ratio test, FDR < 1 × 10^−4^) (Extended Data Fig. 20).

**Fig. 4:**
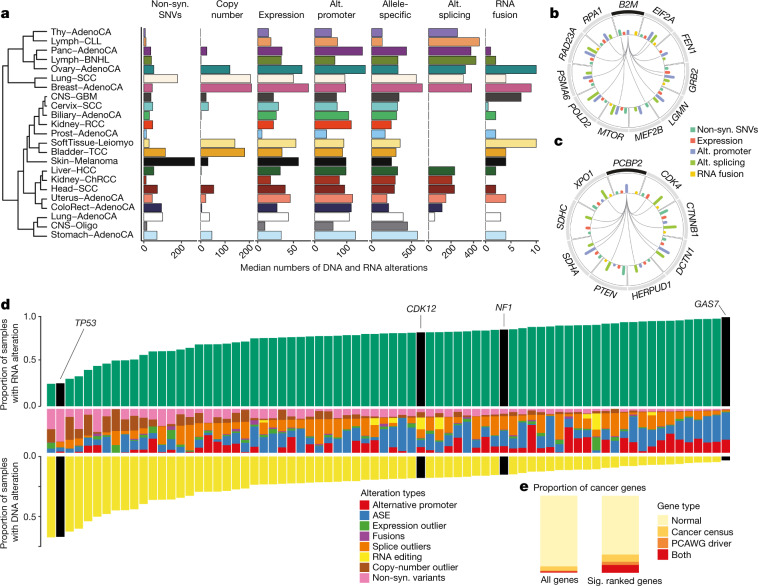
Global view of DNA and RNA alterations that affect tumours. **a**, The median numbers of different alterations across histotypes. Histotypes are ordered by hierarchical clustering based on the pattern of different types of alteration. Only histotypes with more than 10 donors are shown. Alt., alternative; non-syn, non-synonymous. Cancer-type abbreviations are listed in Supplementary Table 23. **b**, **c**, Circular representations of the selected genes significantly co-occurred with *B2M* (**b**) and *PCBP2* (**c**). Connecting lines indicate the specific types of co-occurrence of alteration pairs. The inner histograms indicate the frequencies of incidences of different alteration types shown in different colours. **d**, All 74 Catalogue of Somatic Mutations in Cancer (COSMIC) cancer census genes or PCAWG driver genes that are both frequently and heterogeneously altered across both RNA- and DNA-level alterations. Yellow bars indicate the proportion of samples that had DNA-level alterations, and green bars indicate the proportion of samples with RNA-level alterations. Middle column is the proportion of each alteration type observed for that gene. **e**, The enrichment of cancer genes within our list of significantly recurrent genes.

When comparing gene alteration frequencies across all histotypes (Fig. 4a), we note that different types of cancer contain distinct combinations of DNA- and RNA-level alterations (Fig. 4a, Supplementary Table 17). Although, as expected, skin melanoma significantly exceeds other cancers in the number of non-synonymous SNVs^48^ (Wilcoxon rank-sum test, *P* < 0.012), lymphatic cancers have low numbers of SNVs (Wilcoxon rank-sum test, *P* = 5.3 × 10^−15^), but high incidences of alternative splicing outliers (Wilcoxon rank-sum test, *P* = 4.9 × 10^−47^), which suggests that transcriptomic alterations can be relatively more pronounced in certain cancer types.

**Extended Data Fig. 21:**
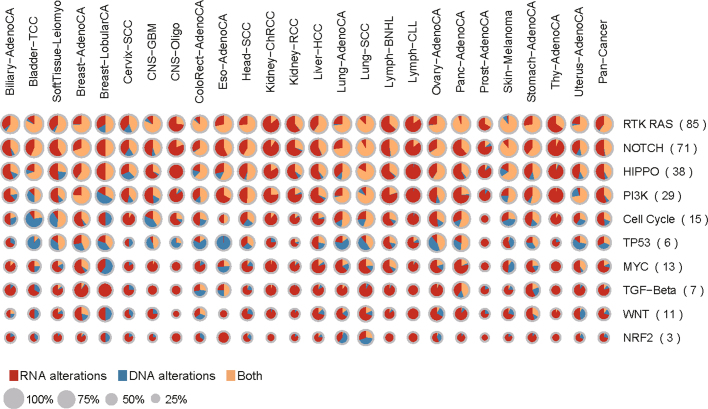
Global view of DNA and RNA alterations affecting cancer pathways. Composite pie charts showing the percentages of RNA alterations, DNA alterations or both, affecting sets of genes in well-characterized cancer pathways and known to be functionally altered in cancer. The sizes of circles represent the percentages of patients affected based on the given gene set. The columns indicate different types of cancer. The numbers in parenthesis indicate the number of genes analysed for the specific pathway.

**Extended Data Fig. 22:**
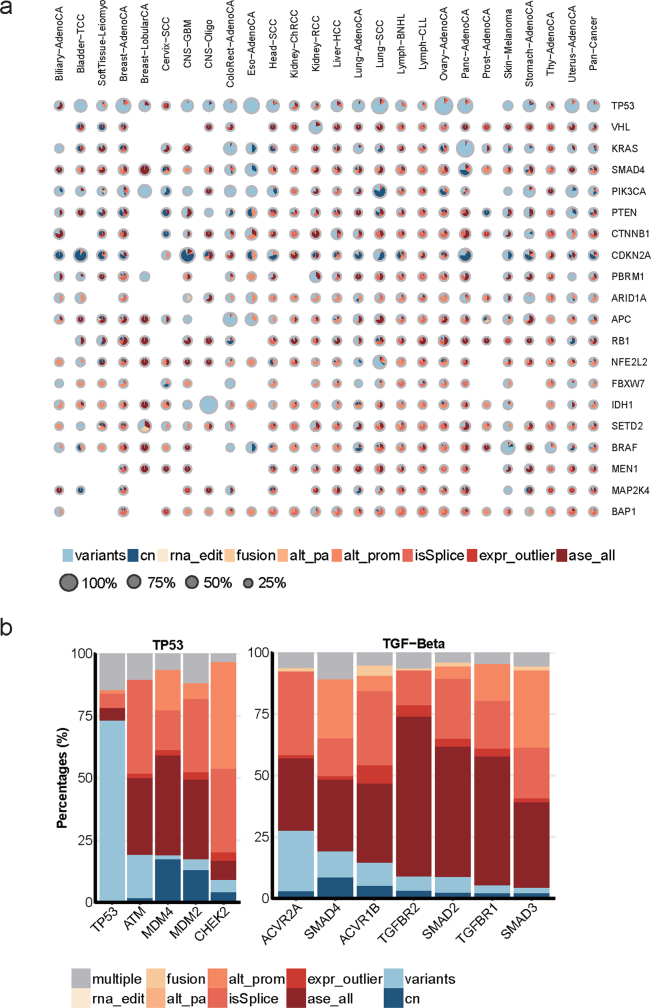
Breakdown of DNA and RNA alterations of cancer genes. **a**, Composite pie charts showing percentages of DNA and RNA alterations for top cancer-driver genes. The 20 most significant cancer-driver genes identified by the PCAWG group in pan-cancer level are depicted, with the sizes of the pie charts indicating the percentages of patients carrying alterations in the given driver gene. The areas represent the relative percentages of patients exhibiting different alterations depicted by corresponding colours. When several types of alteration in one pathway affect the same patient, only a fraction is counted towards each type of alteration. **b**, Proportional bar plots showing the distribution of gene alterations for genes in the *TP53* and *TGFB* pathways.

To evaluate to which extent RNA changes provide additional mechanisms for cancer gene alterations, we examined DNA- and RNA-level alterations both in sets of genes in pathways (Extended Data Fig. 21) and in individual genes with known roles in cancer (Extended Data Fig. 22). We found that RNA alterations occur at a high proportion in many pathways, including the NOTCH and TGF-β pathways. In addition, *KRAS* exhibits more RNA alterations than DNA alterations in some types of cancer. Given the recent finding that alternative splicing of *KRAS* expanded the prognostic affect beyond mutation status in colorectal cancer^49^, our data further support several modes of alteration for *KRAS* in tumours.

## Co-occurrence of RNA and DNA alterations

**Extended Data Fig. 23:**
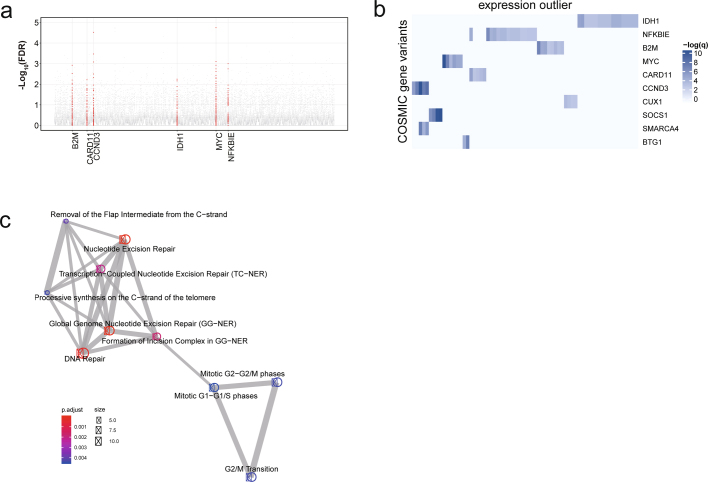
*Trans*-associations found by co-occurrence analyses. **a**, Scatter plot for association of gene expression outliers with cancer gene variants. Each dot represents an alteration pair. The *x* axis shows all COSMIC genes ordered alphabetically and the *y* axis represents the FDR-adjusted *P* values (*q* values) based on Fisher’s exact tests. COSMIC genes with more than five significant associations (FDR < 5%) are coloured in red and labelled. **b**, Heat map showing the extent of associations between COSMIC gene somatic mutations and expression outliers of all genes. Each row indicates one gene, and the colour intensity shows the significance of *trans*-association. COSMIC genes labelled to the right are ordered by the number of significant associations. Only the top 10 genes are shown. **c**, Enrichment map showing the significant (FDR ≤ 0.01) pathways based on the top 100 significant genes associated with *B2M* alterations. Colour intensity represents enrichment significance, node sizes the number of analysed genes belonging to the given pathway and edge sizes the degree of overlap between two gene sets. Only the top 10 enriched terms are shown.

The diverse types of alteration in this study enabled us to investigate *trans*-associations between different genetic and expression characteristics involving cancer-related genes (FDR < 5%) (Supplementary Table 18). By investigating whether somatic mutations of known cancer genes are associated with the expression of other genes, we found *IDH1* and *NFKBIE* to be widely linked to the dysregulation of many genes (Extended Data Fig. 23a, b). Notable co-occurrences were present in several types of cancer. For example, *B2M* and *EIF4G2* alterations were simultaneously observed in both B-cell non-Hodgkin lymphoma and lung squamous cell carcinoma. Pathway enrichment analysis of the top 100 genes associated with all *B2M* alterations indicates that the most affected genes are involved in DNA repair (FDR ≤ 1%), and approximately two-thirds of those associations were significant in more than one cancer type (Fig. 4b, Extended Data Fig. 23c).

We also examined how cancer genes could be affected by other genes by co-occurrence analyses. Expression outliers of *PCBP2* co-occurred with aberrant splicing of a large number of cancer-related genes, including *CTNNB1* and *CDK4* (Fig. 4c). *PCBP2* has been reported to enhance the splicing of cassette exons^50^. Our results thus further support the possible role of *PCBP2* in regulating the splicing of cancer-related genes.

## Recurrent RNA alterations in driver genes

**Extended Data Fig. 24:**
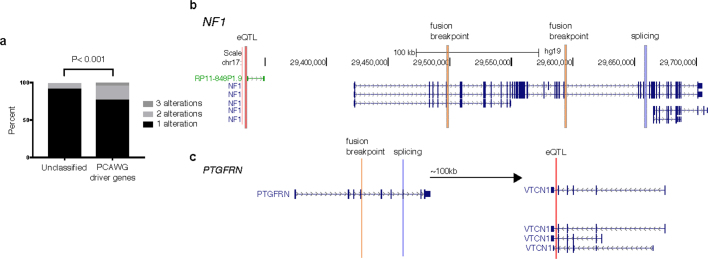
Genes can be altered in *cis* by several mechanisms. **a**, Genes with at least one type of RNA alteration that also has an associated change at the DNA-level in *cis*. Genes are either classified as a PCAWG driver gene or not classified as a driver gene or a cancer gene from the cancer gene census. **b**, **c**, Examples of a known cancer gene, *NF1* (**b**), and an unclassified gene, *PTGFRN* (**c**), having heterogeneous mechanisms of alterations.

In our analyses of *cis*-acting mutations that are associated with these individual RNA phenotypes, the vast majority were observed rarely in the PCAWG cohort. Many cancer genes (such as *MET*^51,52^) are known to be somatically altered by heterogeneous mechanisms such as gene fusions, splicing mutations and non-synonymous mutations; therefore, examining genes that are altered by several *cis*-acting mechanisms may help to identify cancer genes in which an individual alteration type is rare. A total of 5,413 genes were altered by gene expression, allele-specific expression (ASE), splicing and/or gene fusion, and had an associated DNA-level mutation in *cis* (Supplementary Table 20). PCAWG-defined driver genes^8^ tended to have more diverse mechanisms of RNA-level alterations when compared to genes that have not previously been identified as a cancer gene (*P* < 0.001) (Extended Data Fig. 24a). We identified, for example, a somatic eQTL, a splicing-associated variant and fusions in the known tumour-suppressor *NF1* in the MAPK pathway (Extended Data Fig. 24b).

**Extended Data Fig. 25:**
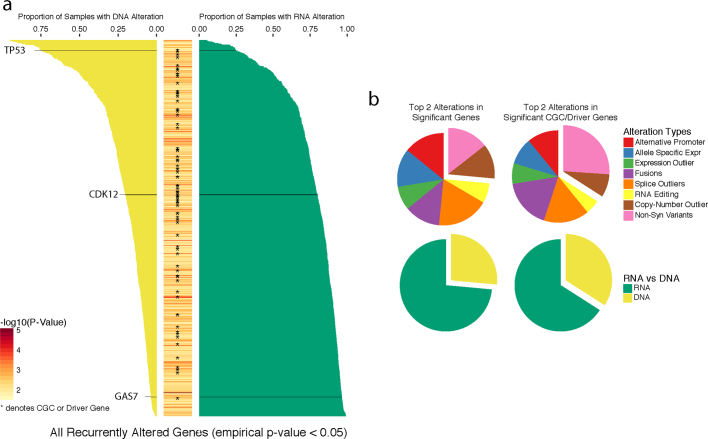
Proportion of genes with DNA or RNA alterations. **a**, Full list of 731 genes that are both frequently and heterogeneously altered across both RNA- and DNA-level alterations. Yellow bars to the left indicate the proportion of samples that had DNA-level alterations, whereas green bars to the right indicate the proportion of samples with RNA-level alterations. Middle column is a heat map corresponding to the −log_10_(*P* value). Asterisks indicate a COSMIC Cancer Gene Census (CGC) gene or PCAWG driver genes. **b**, Distribution of alteration types among all significant genes or just CGC or PCAWG driver genes.

Owing to the fact that most somatic mutations are rare^5^, it is difficult to statistically distinguish functionally relevant, potential driver alterations from passenger alterations. Therefore, we aimed to identify genes that are both recurrently and heterogeneously altered, under the hypothesis that these genes have increased functional relevance. This analysis identified 731 genes with significant recurrent aberrations (FDR < 5%) (Extended Data Fig. 25a), with the top-ranking genes carrying both RNA and DNA alterations. RNA alterations accounted for 0.05–99.14% (mean: 78.23%) of all identified alterations in each gene (Extended Data Fig. 25a, Supplementary Table 21). This ranking is enriched for the union of cancer census genes^53^ (60 out of 603) and PCAWG-defined driver genes (33 out of 157, unioned: 74 out of 674 *P* = 4.6 × 10^−13^, enrichment: 2.45) (Fig. 4d, e).

**Extended Data Fig. 26:**
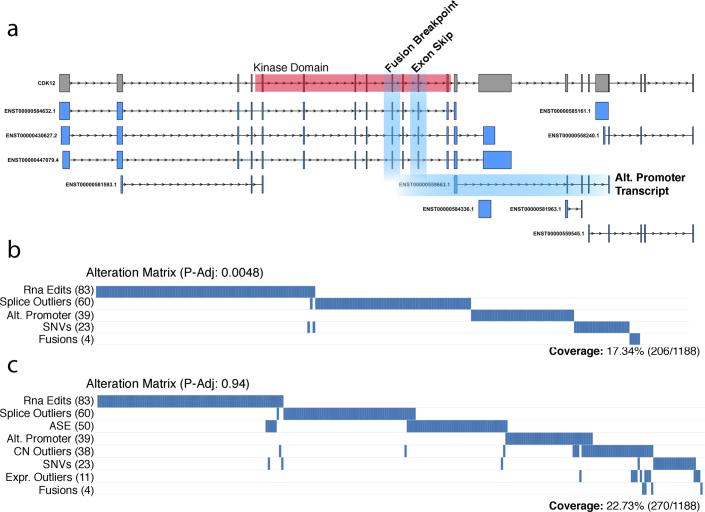
Outlier events in *CDK12*. **a**, Fusion, splicing and alternative promoter outlier events of the RNA alterations that lead to either partial or full removal of the kinase domain in *CDK12*. **b**, All outlier events in *CDK12*, including those not contained directly within the kinase domain, across all 1,188 samples. Each column is a sample and each row is the alteration type. Although not directly searching for mutually exclusive events across all genes, we find that *CDK12* is marginally mutually exclusive in RNA editing, splicing outliers, alternative promoters, non-synonymous variants and fusions (4.810^−3^, unweighted WExT). **c**, All alteration events that occur within *CDK12* across all 1,188 samples, which is not mutually exclusive.

Among the top 10% of our ranked genes is *CDK12* (rank 55). We find 91 samples that have an alteration involving its protein kinase domain, which has been implicated in DNA repair dysregulation^54^. Many of these samples have no DNA-level alterations in *CDK12* (46%) (Extended Data Fig. 26a). Furthermore, splicing, alternative promoter, SNV, RNA-editing and fusion alterations in this gene are mutually exclusive (adjusted *P* = 4.8 × 10^−3^) (Extended Data Fig. 26b, c). Upon further investigation, we find that somatic eQTL mutations in *CDK12* are associated with a tandem duplicator phenotype^55^. Although this association was not replicated with other RNA alterations, it provides evidence that somatic *CDK12* mutations may alter its function through gene expression changes. This example illustrates that performing a recurrence analysis over diverse RNA and DNA alterations can help to identify genes known to be important in tumorigenesis.

## Discussion

Here we present a comprehensive catalogue of RNA-level alterations in cancer, spanning 27 different tumour types, and provide a harmonized resource of matched transcriptome and whole-genome sequences. We identified 731 genes that were recurrently altered by several mechanisms, jointly enriched for known cancer census and PCAWG driver genes^8^. The list includes genes that are primarily altered at the DNA level (such as *TP53*), but also genes for which the alteration most frequently manifests in RNA (such as *GAS7*). Out of 87 samples from the PCAWG study that did not have a driver alteration at the DNA level^5^, and had RNA-sequencing (RNA-seq) data, every sample had an RNA-level alteration identified. Although cancer is thought to be driven by changes in DNA primarily, some driver alterations may manifest themselves via changes in RNA rather than DNA sequence mutations.

We identified germline eQTLs for around 20% of expressed genes. The number of eGenes found is generally low compared with some other studies, reflecting the heterogeneity of our samples. Only 422 genes appeared to be specific to cancer; this is likely to be an underestimate owing to the heterogeneity, small sample numbers and the rather conservative strategy chosen. We have also mapped linkages between genes and somatic aberrations in *cis*, in which 68.4% of associations were between non-coding somatic variants and gene expression. Allelic copy-number imbalance is a major determinant of ASE dysregulation in cancer. We found mutations associated with splicing changes including novel cancer-specific exons that can be partially explained by mutation-driven exonization. We systematically compared gene fusions with whole-genome rearrangements across many tumour types and found 82% of detected fusions were associated with specific genomic rearrangements. For the remaining fusions, it is possible that the relevant genomic rearrangements have not been detected, or that some fusions happen directly at the RNA level, as *trans*-splicing or read-through events. The availability of whole-genome sequences allowed us to develop a systematic classification of fusion events and to propose a new bridged fusion mechanism.

Because global differences in RNA expression phenotypes are largely tissue-specific, our ability to associate mutations in *cis* or *trans* are limited by the small and variable sample sizes within each histotype. Further work is needed to investigate other mechanisms of genome alteration that can lead to changes in RNA such as epigenetic changes^56^ or enhancer hijacking^57^. Our work will help to prioritize further investigations.

Overall, our analyses show diverse modes of alteration of cancer genes and pathways at the DNA and RNA levels, and demonstrate that RNA analyses reveal cancer-associated pathway alterations that have not yet been detected via DNA-only approaches. These insights illustrate the power of integrated transcriptome and whole-genome sequencing analysis for cancer studies.

## Methods

### RNA-seq alignment and quality-control analysis

Tumour and healthy ICGC RNA-seq data, included in the PCAWG cohort^5^, was aligned to the human reference genome (GRCh37.p13) using two read aligners: STAR^58^ (v.2.4.0i, two-pass), performed at MSKCC and ETH Zürich, and TopHat2^59^ (v.2.0.12), performed at the European Bioinformatics Institute. Both tools used Gencode (release 19)^60^ as the reference gene annotation. For the STAR two-pass alignment, an initial alignment run was performed on each sample to generate a list of splice junctions derived from the RNA-seq data. These junctions were then used to build an augmented index of the reference genome per sample. In a second pass, the augmented index was used for a more sensitive alignment. Alignment parameters have been fixed to the values reported in https://github.com/ICGC-TCGA-PanCancer/pcawg3-rnaseq-align-star. The TopHat2 alignment strategies also followed the two-pass alignment principle, but was performed in a single alignment step with the respective parameter set. For the TopHat2 alignments, the irap analysis suite^61^ was used. The full set of parameters is available along with the alignment code in https://hub.docker.com/r/nunofonseca/irap_pcawg/. For both aligners, the resulting files in BAM format were sorted by alignment position, indexed and are available for download in the GDC portal (https://portal.gdc.cancer.gov/) and the ICGC Data Portal (https://dcc.icgc.org/). The individual accession numbers and download links can be found in the PCAWG data release table: http://pancancer.info/data_releases/may2016/release_may2016.v1.4.tsv. Cancer-type abbreviations are listed in Supplementary Table 23. Histology was derived from an older version released by the PCAWG Pathology and Clinical Correlates Working Group. Assignments of donor to histology used in this study can be found in the file rnaseq.extended.metadata.aliquot_id.V4.tsv.gz at https://dcc.icgc.org/releases/PCAWG/transcriptome/metadata/.

Quality control of all datasets was performed at three main levels: (1) assessment of initial raw data using FastQC^62^ (v.0.11.3) (Supplementary Fig. 4); (2) assessment of aligned data (percentage of mapped and unmapped reads for both alignment approaches); and (3) quantification (by correlating the expression values produced by the STAR and TopHat2 based expression pipelines) (Supplementary Fig. 2). In total, we defined six quality-control criteria to assess the quality of the samples. We marked a sample as a candidate for exclusion if: (1) 3 out of 5 main FastQC measures (base-wise quality, *k*-mer overrepresentation, guanine-cytosine content, content of *N* bases and sequence quality) did not pass; (2) more than 50% of reads were unmapped or fewer than 1 million reads could be mapped in total using the STAR pipeline; (3) more than 50% of reads were unmapped or fewer than 1 million reads could be mapped in total using the TopHat2 pipeline; (4) we measured a degradation score^63^ greater than 10; (5) the fragment count in the aligned sample (averaged over STAR and TopHat2) was <5 million; and (6) the correlation between the expression counts of both pipelines was <0.95. If a sample did not pass one of these six criteria it was marked as problematic and placed on a greylist. If more than two criteria were not passed, we excluded the sample.

A subset of 722 libraries from the projects ESAD-UK, OV-AU, PACA-AU and STAD-US were identified as technical replicates generated from the same sample aliquot. These libraries were integrated post-alignment for both the STAR and the TopHat2 pipelines using samtools^64^ into combined alignment files. Further analysis was based on these files. Read counts of the individual libraries were integrated to a sample-level count by adding the read counts of the technical replicates.

Initially, a total of 2,217 RNA-seq libraries were fully processed by the pipeline. Quality-control filtering and integration of technical replicates (722 libraries) gave a final number of 1,359 fully processed RNA-seq sample aliquots from 1,188 donors.

### GTEx data analysis

For a panel of RNA-seq data from a variety of healthy tissues, data from 3,274 samples from GTEx (phs000424.v4.p1) were used and analysed with the same pipeline as PCAWG data for quantifying gene expression. A list of GTEx identifiers are provided at https://dcc.icgc.org/releases/PCAWG/transcriptome/metadata.

### Quantification and normalization of transcript and gene expression

STAR and TopHat2 alignments were used as input for HTSeq^65^ (v.0.6.1p1) to produce gene expression counts. Gencode v.19^60^ was used as the gene annotation reference. Quantification on a per-transcript level was performed with Kallisto^66^ (v.0.42.1). This implementation is available as a Docker container at https://hub.docker.com/r/nunofonseca/irap_pcawg. The implementation of the STAR and TopHat2 quantification is available as docker containers in: https://github.com/ICGC-TCGA-PanCancer/pcawg3-rnaseq-align-star and https://hub.docker.com/r/nunofonseca/irap_pcawg/, respectively. Quantification of consensus expression was performed by taking the average expression based on STAR and TopHat2 alignments. Gene counts were normalized by adjusting the counts to FPKM^67^ as well as FPKM with upper quartile normalization (FPKM-UQ) in which the total read counts in the FPKM definition has been replaced by the upper quartile of the read count distribution multiplied by the total number of protein-coding genes.

The FPKM and FPKM-UQ calculations were as follows. FPKM = (*C* × 10^9^)/(*NL*), in which *N* denotes the total fragment count to protein-coding genes, *L* denotes the length of the gene and *C* denotes the fragment count. FPKM-UQ = (*C* × 10^9^)/(*ULG*), in which *U* denotes the upper quartile of fragment counts to protein-coding genes on autosomes unequal to zero, and *G* denotes the number of protein-coding genes on autosomes.

### *t*-Distributed stochastic neighbour embedding analysis

The *t*-distributed stochastic neighbour embedding (*t*-SNE) plots in Supplementary Figs. 5 and 6 were produced using the RTsne package^68^ (with a perplexity value of 3) based on the Pearson correlation of the aggregated expression (log + 1) of the 1,500 most variable genes. FPKM expression values per gene were aggregated (median) by tissue (GTEx) and study (PCAWG). Coefficient of variation for each gene was also computed per tissue (GTEx) and study (PCAWG) to determine the 1,500 most variable genes. Purity values were previously described^69^.

The *t*-SNE plot in Extended Data Fig. 17c is based on all exon-skipping events in protein-coding genes confirmed by SplAdder^70^. Each event was quantified in both the PCAWG and GTEx cohort. All events with more than 1% of missing percentage spliced in (PSI) values across the concatenated PCAWG and GTEx samples were removed. The remaining missing values were imputed as the mean over the non-missing samples. The centred data were then visualized using the TSNE package from the Scikit Learn toolkit^71^ with a perplexity value of 100, random state 0 and an initialization with PCA.

### Associations between genetic variation and gene expression: patient cohort

To associate genetic variation with gene expression, we analysed whole-genome sequencing (WGS) of the 1,188 donors with matched whitelisted RNA-seq data from the PCAWG cohort. Germline genotypes, SNV calls and segmented allele-specific SCNA calls were previously reported^5^. We matched 1,188 tumour RNA-seq IDs^5^ to WGS whitelist tumour IDs (synapse entry syn10389164). For patients with multiple WGS IDs (2 out of 1,188) or RNA-seq aliquot IDs (17 out of 1,188), we resolved the matching by pairing samples with the same ‘tumor_wgs_submitter_specimen_id’ (Supplementary Table 1). The 1,188 patients are spread across 27 types of cancer and 29 project codes and include 899 carcinomas; 34 patients are metastatic and 13 recurrent with the remaining patients being primary tumours (Supplementary Table 1).

We used the data of these 1,188 patients for performing somatic and germline eQTL mapping, ASE analysis and association studies between gene expression and mutational signatures.

### Gene expression filtering

Gene expression values (measured in FPKM; https://dcc.icgc.org/releases/PCAWG/transcriptome/gene_expression) from consensus expression quantification as described above were used for this analysis.

Genes with FPKM ≥ 0.1 in at least 1% of the patients (12 patients) were retained, resulting in 47,730 genes. Only 18,898 protein-coding genes (according to the ‘gene_type’ biotype reported in Gencode v.19^60^) were used for the subsequent QTL analyses. The log_2_-transformed expression values (FPKM + 1) were subjected to peer analysis^72^ to account for hidden covariates (syn7850427; https://dcc.icgc.org/releases/PCAWG/transcriptome/eQTL/phenotype). To balance the number of covariates, statistical power and available sample sizes per cancer type, we followed the GTEx protocol and estimated 15, 30 and 35 hidden covariates to be used depending on sample size^73^ (*n* < 150, 150 ≤ *n* < 250, *n* ≥ 250). Peer residuals were then rank-standardized across patients. The FPKM cut-off values and peer correction were also applied to the subset of 899 patients with carcinoma, yielding 18,837 protein-coding genes after filtering. Furthermore, we used ordinary least-squares regression to correlate each of the 35 peer factors with per-sample covariates, including cancer project codes, gender, tumour purity, somatic burden and several sequence metrics (Supplementary Notes), to understand the proportion of variance explained by known biological and technical covariates.

### Covariates

In all linear models, we accounted for known confounding factors by modelling them as fixed effects. In all association studies, we accounted for sex, project code (describing cancer type and country of origin) and per-gene copy-number status (Supplementary Table 1 for the list of per patient covariates; syn7253568 and syn7253569 for sex and project codes; syn9661460 for per gene copy number). Per-gene copy-number alterations were derived as the average copy number across all copy-number aberrations called within the annotated gene boundaries based on syn8042988.

The somatic eQTL, ASE and mutational signature analyses also accounted for total somatic mutation burden (number of SNVs and short insertions and deletions (indels)) and sample purity (Supplementary Table 1). Purity was estimated based on copy-number segmentation. In addition, the somatic eQTL and ASE analyses accounted for local SNV burden calculated in a 1-Mb window from the gene coordinates (https://dcc.icgc.org/api/v1/download?fn=/PCAWG/transcriptome/eQTL/covariates/pergene.somatic.snv.cis.burden.1188.wl.donors.tsv.gz).

The germline eQTL analysis also modelled the population structure as random effect. The population structure was assessed by a kinship matrix that was calculated based on every twentieth germline variant, processed as described below (see ‘Germline eQTL variants’). The kinship matrix was then calculated as an empirical patient-by-patient covariance matrix.

Different covariates were accounted for per-analysis method (Supplementary Table 1). The project code describes cancer type and country-of-origin. Somatic burden is the total number of SNVs and indels. Purity was estimated based on copy-number segmentation. Local somatic burden is the number of SNVs in a 1-Mb window around the gene coordinates. Local copy number was defined as the average copy-number state across all SCNAs called within the annotated gene boundaries.

### GO and Reactome pathway enrichment

We performed GO^74,75^ and Reactome pathway^20,21^ enrichment with the Bioconductor packages biomaRt^76,77^, clusterProfiler^78^ and ReactomePA^79^ (FDR ≤ 10%). The number of genes used as background set is described per analysis method.

### Germline eQTL variants

PCAWG variant calls v.0.1^5^ were downloaded from GNOS and processed following the PCAWG-8 protocol: (1) VCF files were indexed and merged using bcftools^80^. (2) All variants were filtered for ‘PASS’ flag. (3) All variants were filtered for quality larger than 20. (4) Only bi-allelic sites were considered.

HDF5 files for each 100-kb chunk of the VCF files were generated, assuming additivity that was numerically encoded as 0, 1 or 2 for homozygous reference, heterozygous or homozygous alternative state, respectively. For indels, we encoded the presence or absence of the variant as 0 or 1, respectively. Each variant was normalized to mean 0 and standard deviation 1. Missing variants were mean-imputed. To create our eQTL release set v.1.0, the resulting HDF5 files were subsequently merged into a global HDF5 file and all variants which follow any of the following conditions were removed: (1) minor allele frequency ≤ 1%; and (2) missing values ≥ 5%

### Germline eQTL analysis

In the germline eQTL analyses, we used the processed gene expression dataset from 1,178 patients for which germline variant calls (eQTL release set v.1.0, see ‘Germline eQTL variants’) were available. Linear mixed models were used to model the correlation between germline variants (within 100 kb of gene boundaries) and gene expression values (see ‘Gene expression filtering’) using the limix package^81^. Known covariates were modelled as fixed effects and population structure as random effect (see ‘Covariates’).

A two-step approach was used to adjust for multiple testing. First, for each gene, we adjusted for the number of independent tests estimated based on local linkage disequilibrium^82^. Second, we performed a global correction across the lead variants, that is, the most significant SNPs, per eQTL. Germline eGenes were defined as genes with an eQTL with global FDR ≤ 5%.

### GTEx comparative analysis

The GTEx comparative eQTL analysis was based on the eQTL maps v.6p^10^. We mapped the positions and alleles of our PCAWG-specific eQTL to the eQTL in all GTEx tissues. To determine whether a lead eQTL variant is replicated in a given GTEx tissue, we followed the previously described strategy^10^. For each eGene, we considered the eQTL lead variant and assessed the replicability of the signal in the GTEx cohort based on marginal association statistics using 42 GTEx tissues without cell lines (*P* < 0.00024 = 0.01/42, corrected for the number of GTEx tissues—that is, 42)). If the lead variant did not replicate or was not tested, we determined replication based on the variant with the smallest *P* value within the linkage disequilibrium block (*r*^2^ ≥ 0.8 estimated based on UK10K project) of the lead variant across 25 (or 42) tissue-matched GTEx analyses. If neither lead nor any variant within the linkage disequilibrium block was tested, we determined replication based on the smallest *P* value of any variant within the 100-kb window tested within the GTEx cohort. We also derived less stringent sets of PCAWG-specific eGenes by allowing replication in up to 1, 5 or 10 GTEx tissues.

### Tissue sharing of germline eGenes between histotypes

Using the R package qvalue (https://github.com/StoreyLab/qvalue, v.2.14.0), we generated *π*_1_ statistics comparing the lead variants of one histotype against their *P* value distribution in the other histotypes. Because *π*_1_ statistics are known to be confounded by sample size and number of eQTL found, we subsampled the eQTL lead variants to a randomly selected set of 100 variants. After 20 rounds of subsampling, we derived the same *π*_1_ statistics as mentioned earlier and reported the average.

### Roadmap enrichment of germline eGenes

For each lead variant, we generated a matching background set of 1,000 variants using SNPsnap^83^. Each variant (background and foreground) was intersected with the location of 25 Roadmap factors^16^ in 127 cell types. From this we derived fold change and *P* values. Significant changes of fold change between PCAWG-specific and unspecific eQTLs is based on a one-sided Wilcoxon rank-sum test.

### Enrichment analysis

Enrichment of Reactome pathways of PCAWG-specific eGenes was performed using the Bioconductor package ReactomePA^79^.

### Somatic calls and mutational burden

We used the set of consensus SNVs somatic calls provided by PCAWG (syn7357330) based on three core caller pipelines and MuSE^84^. On average, we counted 22,144 somatic SNVs per patient, with different median numbers of SNVs per cancer type, ranging from 1,139 in thyroid adenocarcinoma to 72,804 SNVs in skin melanoma (Extended Data Fig. 5a). Owing to the low frequency of somatic SNVs across the cohort (Extended Data Fig. 5b), we collapsed the variants by genomic regions defined by gene annotations (Gencode v.19^60^). Specifically, we generated a set of disjoint gene exons by collapsing overlapping exon annotations into single features using bedtools^85^. The set of disjoint introns was generated using bedtools by subtracting the collapsed exonic regions from the gene regions. To map local effects of somatic mutations in flanking features outside the gene body, we binned the surrounding regions (plus and minus 1 Mb from the gene boundaries) into 2-kb windows (flanking) overlapping by 1 kb.

We defined three different types of aggregated somatic burden to assess differences in power in detecting somatic eGenes and *P* value calibration. The burden in a genomic region was defined as (1) a binary value that indicates presence or absence of SNVs; (2) the aggregated burden as sum of SNVs; or as (3) weighted burden, that is, sum of variant allele frequencies of the SNVs (Supplementary Fig. 10a) to take into account their clonality (https://dcc.icgc.org/releases/PCAWG/transcriptome/eQTL/genotypes). We assessed calibration of all three analyses with Q–Q plots of nominal and permuted *P* values (permutation of the patients in the gene expression matrix) (Supplementary Fig. 10b–d). Moreover, for the linear regression analysis, genotypes were standardized across patients (to mean zero and standard deviation one) and standardized effect sizes are provided in Supplementary Table 5.

Overall, somatic burden within flanking regions was the most prevalent type of burden tested per gene (Extended Data Fig. 6a). We found similar average relative mutation density per type of genomic region (flanking = 0.008 mutations per kb; introns = 0.007 mutations per kb; exons = 0.006 mutations per kb) (Extended Data Fig. 6b) and average recurrence of the same mutated region across the cohort was rather low (flanking = 1.4%; exons = 1.7%; introns = 4%) (Extended Data Fig. 6c).

### Somatic eQTL analysis

Linear models were used to model the correlation between recurrent somatic burden and gene expression of up to 18,898 protein-coding genes, using the limix package^81^ (see ‘Gene expression filtering’). Gene expression was corrected for 35 hidden Peer factors. Known covariates were modelled as fixed effects (see ‘Covariates’). We considered only somatic burdens with frequency greater than 1%, including exonic and intronic burdens, as well as flanking burdens, within 1 Mb from gene boundaries.

The somatic eQTL analysis was performed on all 1,188 patients and on the subset of 899 patients with carcinoma (representing 20 of the 27 types of cancer) to replicate the analysis on a more homogeneous set of tumours. A *cis* window of 1 Mb from the gene boundaries was used to find mutated genomic intervals with a burden frequency ≥ 1% in the cohort (at least 12 patients in the full cohort and 9 patients in the carcinoma cohort). Together, 18,708 of the genes had at least one mutated interval at that frequency and were included in the analysis and 1,049,102 regions showed a burden frequency ≥ 1%

Bonferroni correction was applied to correct for multiple *cis* windows tested within the same gene. Then, Benjamini–Hochberg correction was applied to adjust the *P* values of the lead genomic regions across genes. Somatic eGenes were defined as genes with an eQTL at a FDR ≤ 5%.

### Somatic cis-eQTL comparative analysis

We compared our 649 somatic eQTL set with three previous cancer studies^86–88^ to identify independent evidence of interaction between our eGenes and the associated *cis-*genomic regions with somatic burden. Studies were chosen if they provided lists of cancer regulatory elements linked to genes or regulatory elements with somatic mutations linked to gene expression deregulation in cancer. All the three studies examined were based on TCGA cancers. For this, we checked perfect overlaps with both the somatic burden location and the eGene. Moreover, we looked at the overlap between somatic eQTL and 72,987 GeneHancer^89^ enhancers-to-genes interactions, with at least two independent supporting methods (called ‘double-elite’), downloaded from the UCSC hg19 GeneHancer track^90^. We then compared this overlap with a set of nulls generated by 1,000 random permutations of the GeneHancer regulatory elements with nearby genes located within 1 Mb. We then retrieved an empirical *P* value of enrichment by counting the number of random nulls (*N*) showing greater number of overlaps than those found between the somatic eQTL set and the GeneHancer set (*P* = (*N* + 1)/(1,000 + 1)).

### Functional enrichment in somatic *cis*-eQTL

To identify putative regulatory sites enriched for somatic eQTL, we retrieved functional annotations of the lead genomic flanking intervals of the somatic eQTL (556 intervals linked to 638 somatic eQTL). Therefore, we mapped somatic eQTL to 25 Roadmap Epigenomics chromatin marks of 127 different cell types^16^ and ENCODE transcription-factor binding site annotations in 9 cell types (including 8 cancer and one embryonic stem-cell lines^91^) (Supplementary Tables 6 and 7). We compared annotations in the significant set of eQTLs with a null distribution based on 1,000 random samplings of a matched set of genomic intervals. To define the matched sets of genomic intervals, we selected flanking genomic intervals from the whole set of tested genes that showed a similar distance from the gene start (exact distance ± 2 kb) and that matched the exact burden frequency of the corresponding interval in the significant associations. We then overlapped the 1,000 matched sets with Roadmap Epigenomics and ENCODE annotations. To avoid ambiguous overlaps (with multiple annotations), we retained only genomic intervals showing a minimum overlap of 10% of their length.

We retrieved an empirical *P* value of enrichment for each annotation by counting the number of randomly sampled flanking intervals (*N*) showing greater number of overlaps compared to the eQTL set (*P* = (*N* + 1)/(1,000 + 1)). Benjamini–Hochberg correction was applied to the empirical *P* values (over 25 marks in 127 cell lines for Roadmap Epigenomics annotations and over 149 transcription-factor-binding sites for 9 ENCODE cell lines). We then computed the fold change per annotation and cell line as a ratio of annotated lead flanking intervals and mean number of annotated matched random flanking intervals over the 1,000 samplings.

Furthermore, we performed GO^74,75^ and Reactome pathway^20,21^ enrichment with the Bioconductor packages biomaRt^76,77^, clusterProfiler^78^ and ReactomePA^79^ (FDR ≤ 10%) and also looked at enrichment within high-confidence cancer testis genes previously described^92^, using 18,708 genes with at least one mutated interval as background.

### Variance component analysis

Limix was used to perform variance decomposition using the same covariates as in the somatic variant analyses except for local copy-number state (see ‘Covariates’). The random effects were based on the following common germline variants and somatic burden (frequency > 1%) (see ‘Somatic calls and mutational burden’ for detailed description of burden): (1) *cis*-somatic intronic: weighted burden in introns; (2) *cis*-somatic exonic: weighted burden in exons; (3) *cis*-somatic flanking: weighted burden in 1-kb-overlapping regions of 2 kb within 1 Mb from gene boundaries; (4) somatic intergenic: weighted burden in 1-kb-overlapping regions of 2 kb outside the 1 Mb window; (5) *cis*-germline: germline variants within 100 kb from gene boundaries; (6) *trans*-germline: genome-wide population structure (see ‘Covariates’); and (7) local copy-number variation (see ‘Covariates’).

All the data was mean-centred and standardized. For each of the random effects, a linear kernel was computed and used as covariance matrix. The resulting variance components were normalized to add up to one.

### Mutational signature associations

We obtained 39 mutational signatures from PCAWG-7 beta 2 release^9^ and used linear models to associate the mutational signatures with gene expression of up to 18,898 protein-coding genes across 1,159 patients while accounting for known covariates (see ‘Covariates’) (quality control) (Extended Data Fig. 10a–e). The 1,159 patients were a subset of the total 1,188 patients, for whom mutational signature profiles were available. Gene expression was corrected for 35 hidden peer factors (see ‘Gene expression filtering’).

We retained 18,888 genes that showed a minimum FPKM of 0.1 in at least 1% of 1,159 the patients (see ‘Gene expression filtering’). Signatures with zero variance and a prevalence below 1% were filtered, and we obtained 28 signatures. We applied linear models to associate expression of these genes with the signatures across all 1,159 patients, a subset of 877 patients with carcinoma or a subset of 891 European patients to assess consistency of the associations (Extended Data Fig. 10f, g).

Across all patients, we found 1,176 significantly associated genes after Benjamini–Hochberg correction (we used an FDR ≤ 10% for enrichment analyses, multiple testing was applied across all signature–gene pairs) (Supplementary Tables 19a–c). We performed gene enrichment analyses of the significant genes per signature (see ‘GO and Reactome pathway enrichment’) (here 18,831 background genes, multiple testing correction across all ontologies per signature FDR ≤ 10%) (Supplementary Table 19d). Whereas most signatures were associated with only few genes, 18 showed recurrent *trans* effects and affected expression of over 20 genes (Extended Data Fig. 11d, Supplementary Table 19e). We further found that the vast majority of genes (85.8%) were associated with only one signature (1,009 genes); 129 genes were associated with two, 32 with three, 5 with four and 1 with five signatures.

To assess how tissue-specific both mutational signatures and their associations with gene expression are, we analysed the occurrence of each signature in each of the types of cancer. We assessed the presence (at least one SNV of a signature in at least one patient with a specific cancer type) and mean prevalence (mean number of SNVs of a certain signature across all patients of a specific cancer type) of the signatures in the types of cancer (Extended Data Fig. 13c, d). We defined cancer-type-specific signatures to occur in up to four types of cancer (signatures 4, 7, 9, 12, 16, 38 and 39) and common signatures to be missing in up to five types of cancer (signatures 2, 13 and 18). For each of these signatures, we performed cancer-type-specific analyses, that is, we assessed the association between the respective signature and gene expression in just the patients who are of a cancer type that shows mutations of the respective signature (Extended Data Fig. 13c, left heat map). We then correlated the *P* values of these cancer-type-specific analyses with the *P* values of the analysis across all patients and calculated the Pearson correlation coefficients (Supplementary Fig. 24a–e). We show that the correlation between cancer-type-specific and whole-cohort *P* values is dependent on the sample size of the respective analysis (*r*^2^ = 0.671) (Supplementary Fig. 1f).

We further performed PCA on the signatures across both, patients (PCA on signature-specific SNVs per patient) and genes (PCA on adjusted *P* values of signature-gene expression associations) (Extended Data Fig. 11a, b).

To assess significance of the functional annotation of SNVs by mutational signatures, we also associated gene expression with the total number of SNVs and correlated the *P* values (−log_10_(*P*)) of the associations with the respective signature-specific *P* values. The absolute Pearson correlation coefficients remain below 0.1 (Supplementary Table 19f).

To establish causality of signature–gene expression associations, we included the germline eQTL into the analysis using linear mixed models; 197 of our 1,176 signature-associated genes were also germline eGenes. These 197 associations involved 26 of the 28 mutational signatures. We associated the lead variants of these eGenes with the rank-standardized signature SNVs across 2,507 patients. We used the subset of the 2,818 WGS patients for which mutational signature profiles and all known covariates were available. We accounted for the same fixed covariates as in the mutational signature–gene expression association studies and, in addition, for kinship as a random effect (see ‘Covariates’).

We then performed proportional colocalization analysis with Bayesian model averaging using the R package coloc^93^ to test whether gene expression and mutational signatures share common causal genetic variants in a given gene region. A proportional colocalization analysis tests the null hypothesis of colocalization by assuming that two phenotypes that share causal variants will have proportional regression coefficients for either phenotype with any variant selection in the vicinity of the causal variant. We applied the Bayesian model averaging approach, with each tested model consisting of a selection of two variants. The *P* values are then averaged over all models to generate posterior predictive *P* values^93^. We filtered variants so that no pair of variants showed *r*^2^ > 0.95 and each variant’s marginal posterior probability of inclusion with one of the phenotypes was greater than 0.01. The nominal *P* values of rejecting the null hypothesis of colocalization are listed in Supplementary Table 19e.

We then performed mediation analysis^94,95^ to assess directionality of the effect between germline eQTL, gene expression and mutational signature. First, causal mediation analysis was applied to each of the triples of eQTL lead variant, gene and mutational signature using a structural equation model from the R package lavaan^96^. Then, we used the R package mediation^97^ to assess significance of mediation and estimate the proportion of mediated effect by non-parametric bootstrap confidence intervals (1,000 simulations).

### ASE analysis: assembling phased germline and somatic variants

To understand the precise effect of somatic variations in their genomic context and for subsequent allele-specific analyses, both germline and somatic variants were phased. For assembling phased germline genotypes, we used the Sanger 1000G callset^6^, and applied IMPUTE2^98^ for phasing of heterozygous germline variants. The IMPUTE2 output was corrected using results from the Battenberg CN calling algorithm^99^ to ascertain that no haplotype switches occur within regions of consecutive copy-number gain. The resulting phased germline genotypes were arranged such that haplotype 1 always corresponded to the amplified alleles in regions with SCNAs (major allele). In cases in which both co-occur on the same NGS read (approximately 10 million variants, 20% of all SNVs), we phased individual somatic variants to the nearest germline heterozygous site. For downstream analyses, we considered only SNVs that were phased by at least three reads to the respective germline variant (approximately 6 million out of 10 million SNVs).

All phased SNVs were aggregated into functional categories based on their genomic regions defined by gene annotations (upstream, downstream, promoter, 5′ UTR, intron, synonymous, missense, stop gain and 3′ UTR) and mapped to the nearest gene within a *cis* window of 100 kb using the Variant Effect Predictor (VEP) tool^100^. Promoter variants were defined as 1-kb upstream of the TSS. We included flanking regions by using the VEP ‘UpDownDistance’ plugin with a maximum range parameter of 100 kb. We divided the upstream and downstream variant categories into disjoint categories using 10-kb windows from 10 to 100 kb. We integrated ‘splice donor’ and ‘splice acceptor’ variants into the general ‘splice region’ variant category and mapped ‘stop retained’ variants to the ‘synonymous’ variant category. We averaged transcript-level annotations to gene-level annotations to retrieve the expected functional effect of a variant for a given gene. We analysed the relationship between SNV variant allele frequency and SCNAs at the same locus to determine whether variants occurred before (‘early’) or after (‘late’) the corresponding SCNA (PCAWG-11). We computed a weighted *cis*-mutational burden per category by estimating the cancer cell fraction of each SNV and aggregating SNVs to a total localized burden weighted by their respective cancer cell fraction.

### ASE read counts

The positional information of the heterozygous germline variants was used together with the RNA-seq BAM files as input to the GATK ASEReadCounter^101^ algorithms for counting ASE reads. We considered reads with a minimum mapping quality of 20 and a minimum base quality of 10. Only heterozygous variants with a minimum coverage of eight RNA-seq reads were considered for all further analyses.

The raw ASE read counts were post-processed as follows: (1) ASE sites were converted to BED files and aligned against the ENCODE 50-mer mappability track (wgEncodeCrgMapabilityAlign50mer.bigWig) to extract mappability scores for all sites. All sites with mappability scores unequal to 1 were removed. (2) All sites with allelic read counts less or equal to 1 were removed to prevent genotyping error to influence ASE quantification. (3) All sex chromosomes were dropped for further analysis. (4) We estimated sequencing error per patient as the sum of non-reference and non-alternative bases over the total number of bases. We assessed statistical mono-allelicity through a binomial test using the estimated sequencing error probabilities, corrected using the Benjamini–Hochberg step down procedure. All sites that appeared to be statistically mono-allelic were removed. (5) For each ASE site, copy-number states were retrieved from the Sanger copy-number consensus callset (PCAWG-11). Purity estimates for each patients were retrieved from the accompanying purity tables.

To aggregate site-level ASE to a gene-level readout and to allow for estimation of effect directionality, we used the phased germline genotypes. Gene mapping was performed against ENSEMBL release 75 using the pyEnsembl Python library. We retrieved all genes at each ASE site and summed up the read counts on the respective haplotypes to gene-level haplotype-specific read counts. We further averaged haplotype-specific copy-number states to a mean haplotype-specific copy-number state per gene and computed the gene-level copy-number ratio as the major over total ratio of those averages. To allow for a robust assessment of gene-level ASE, we considered only genes with at least 15 reads total, yielding 4,379,378 gene–patient pairs of 1,120 patients and 17,009 unique genes across 12,441,502 accessible sites in total. Every remaining gene was tested for AEI using a binomial test against an expected read ratio of 0.5 to derive nominal *P* values, and a binomial test against the expected copy-number ratio modified by tumour purity to derive copy-number-corrected *P* values. Nominal and copy-number-corrected *P* values were adjusted separately for multiple testing using the Benjamini–Hochberg procedure. Significant AEI was called at FDR ≤ 5%. We further annotated each gene with the number of ASE sites used for aggregation. For all downstream analyses, we considered only genes annotated as protein coding (ENSEMBL biotype = ‘protein_coding’).

### Generalized linear models

Across all 4,379,378 gene–patient pairs, we trained multivariate linear models using (i) logistic regression against a binary indicator of AEI absence or presence in a gene, or (ii) standard linear regression against the phased ASE ratio of a gene to assess the directionality of the regulatory change. For (i), haplotype-specific mutations were summed up to a total burden per category, whereas for (ii) we used the difference in burden between the haplotypes 1 and 2. The consistency of the phasing map between somatic variants and ASE sites ensured that model coefficients kept their directionality independent of the arbitrary labelling of haplotypes as 1 or 2. The full set of considered factors is as follows: (1) copy-number ratio at the gene locus (0.5 ≤ *x* ≤ 1); (2) sample purity (0 < *x* < 1); (3) natural logarithm of total gene length (*x* > 0); (4) natural logarithm of the length of the canonical transcript (*x* > 0); (5) heterozygosity of the lead eQTL variant (*x* = 0 if homozygous, *x* = 1 if not homozygous); (6) all mutational burden categories as determined by VEP annotations (upstream in 10-kb windows, downstream in 10-kb windows, promoter, 5′ UTR, intron, synonymous, missense, stop gain and 3′ UTR; *x* ≥ 0 for logistic model, *x* ∈ ℝ for directed model).

To compare global effects and different contributions of SCNA, germline eQTL, coding and non-coding SNVs, a simplified logistic model was trained after accumulating all coding and non-coding variants to separate categories and reporting standardised effect sizes (Fig. 1e).

### Cancer gene enrichment

Cancer gene enrichment was conducted on the COSMIC census^53^ using Fisher’s exact test and gene set enrichment analysis as previously described^102^. For enrichment, the average score of a gene was computed across the cohort and only genes with at least five replicates in the cohort were kept, yielding a total of 16,078 genes.

### Chromosomal distribution of ASE

We calculated the recurrence of ASE genes in each tumour type. To examine the chromosomal distribution of ASE genes, we calculated the average recurrence of all genes for every 200-gene window with a 10-gene step, and then subtracted the average ASE occurrence in each tumour type to obtain the peaks of ASE surplus across all chromosomes. The recurrence of copy-number genes was calculated in an analogous manner.

### Estimation of alternative promoter activity

We estimated promoter activities using RNA-seq data and Gencode (release 19) annotations for 70,937 promoters in 20,738 genes. We grouped transcripts with overlapping first exons under the assumption that they are regulated by the same promoter^103^. TSSs that are located within internal exons, or which overlap with splice acceptor sites, were removed from this analysis as these promoters are difficult to estimate from RNA-seq data^28^. Promoter activity can be estimated using exon usage^29^, spliced reads^28^ or isoform-based estimates^30^. Here we used an isoform-based approach to quantify promoter activity. We quantified the expression of each transcript from the RNA-seq data using Kallisto^66^ and calculated the sum of expression of the transcripts initiated at each promoter to obtain an estimate of promoter activity. To obtain the relative activity for each promoter, we normalized each promoter’s activity by the overall gene’s expression. We divided the promoters of each gene into three categories based on their average pan-cancer promoter activity. The promoters with <1 FPKM average activity are called inactive promoters, and the most active promoter of each gene is called the major promoter. The remaining active promoters of the gene are called minor promoters.

The association between promoter activities and promoter mutation burden was estimated using the same framework as the somatic eQTL analysis. We examined associations for the promoters of expressed multi-promoter genes with a burden frequency ≥ 1% in the cohort (at least 12 patients in the full cohort). The weighted burden of the region 1-kb upstream of the TSS—that is, the sum of variant allele frequencies of the SNVs for each gene—was used as the genotype for the promoters of the respective genes. We used linear models to study the associations between the recurrent somatic burden and the promoter activity (both for the relative activity and the log_2_-transformed absolute activity). Similar to the somatic eQTL analysis, the known covariates and the 35 hidden peer factors were provided as cofactors to the linear models. We adjusted the *P* values using Benjamini–Hochberg correction method and looked for associations with FDR ≤ 5%.

### Identification of alternative splicing

We used the alignments based on the STAR pipeline to collect and quantify alternative splicing events with SplAdder^70^. The software has been run with its default parameters with confidence level 3. We generated individual splicing graphs for each RNA-seq sample for both tumour samples as well as matched healthy samples (when available). All graphs were then integrated into a merged graph to comprehensively reflect all splice junctions observed in all samples together. On the basis of this combined graph, SplAdder was used to extract alternative splicing events of the following types: alternative 3′ splice site, alternative 5′ splice site, cassette exon, intron retention, mutually exclusive exons, coordinated exon skip (see supplementary figure 3 in ref. ^70^). Each identified event was then quantified in all samples by counting split alignments for each splice junction in any previously identified event and the average read coverage of each exonic segment involved in the event was determined. We then computed a PSI value for each event that was then used for further analysis. We further generated different subsets of events, filtered at different levels of confidence, in which confidence is defined by the SplAdder confidence level (generally 2), the number of aligned reads supporting each event, the number of samples that were found to support the event by SplAdder, and the number of samples that passed the minimum aligned read threshold.

### Enrichment of outlier splicing associated with splice sites and branchpoint motifs

We assessed the significance of mutational enrichment for 5′ and 3′ splice sites, and branch-point^104,105^ intronic regions using a permutation-based approach. Impactful mutations were defined as mutations overlapping exons and introns involved in cassette exon events, in which the PSI-derived *z*-score was ≥ 3 or ≤ −3. For each intronic site, we compared the frequency of observed impactful mutations against frequencies of randomly sampled intronic regions (number of iterations = 1,000). For exonic sites, the null distribution was established from randomly sampled exonic sites. Randomly sampled sites were within a 100-bp window around the 5′ and 3′ splice site. For branch-point regions, sampled sites were within a 50-bp window around the branch-point sequence. The *P* value was computed as the number of randomly sampled frequencies greater or equal to the observed frequency.

### SAVNet analysis for identifying rare SAVs

The SAVNet approach^35^ was designed for identifying somatic variants associated with local aberrant splicing alterations from matched genome and transcriptome sequencing data. It uses permutations to calculate an FDR and by restricting to two classes of relationships between somatic mutations and splicing alterations to focus: (1) splice site disruption, in which exon skipping, alternative 5′ or 3′ splice site, or intron retention is associated with a mutation in a splice site motif; and (2) splice site creation, in which alternative 5′ or 3′ splice sites are associated with mutations that create a novel splice motif (FDR ≤ 10%) (Extended Data Fig. 17e).

### Identification of RNA fusions

Gene fusions between any two genes were identified based on two gene fusions detection pipelines: FusionMap (v.2015-03-31) pipeline^106^ and FusionCatcher (v.0.99.6a)/STAR-Fusion (v.0.8.0) pipeline^107^. ChimerDB 3.0 was used as a reference of previously reported gene fusions. The database contains 32,949 fusion genes split into three groups: (1) KB: 1,067 fusion genes manually curated based on public resources of fusion genes with experimental evidences; (2) Pub: 2,770 fusion genes obtained from text mining of PubMed abstracts; and (3) Seq: archive with 30,001 fusion gene candidates from deep-sequencing data. This set includes fusions found by re-analysing the RNA-seq data of the TCGA project encompassing 4,569 patients from 23 types of cancer.

In brief, FusionMap was applied to all unaligned reads from the PCAWG aligned TopHat2 RNA-seq BAM files for each aliquot to detect gene fusions. In the FusionCatcher/STAR-Fusion pipeline, for each aliquot with paired-end RNA-seq reads FusionCatcher was applied to the raw reads, with the genome reference. Specifically, for each aliquot with paired-end RNA-seq reads FusionCatcher was applied to the raw reads. The ‘-U True; -V True’ runtime options were used. For each aliquot with single-end RNA-seq reads, STAR-Fusion was applied to the raw reads, with the same reference genome and gene models as FusionCatcher and with default settings. In parallel, FusionMap was applied to all unaligned reads from the PCAWG aligned TopHat2 RNA-seq BAM files for each aliquot to detect gene fusions with the following non-default options values: MinimalHit = 4; OutputFusionReads = True; RnaMode = True; FileFormat = BAM.

To reduce the number of false-positive fusions, the two sets of fusions were filtered to exclude fusions based on the number of supporting junction reads, sequence homology, and occurrence in normal samples (from the GTEx and the PCAWG cohort). To get a high-confident consensus fusion call set from these two pipelines, a fusion to be included in the final set of fusions had to: (i) be detected by both fusion detection tools in at least one sample; and/or (ii) be detected by one of the methods and have a matched structural variant in at least one sample. The consensus WGS-based somatic structural variants (v.1.6) were obtained from the PCAWG repository in https://dcc.icgc.org/releases/PCAWG.

For integration with matched structural variant evidence, a fusion was considered to match a structural variant if the absolute distance between the fusion break points and structural variant break points did not exceed 500 kb (the distance was considered infinite when the chromosomes of the fusion and structural variant break point differ). When there was no evidence for a direct structural variant fusion, the search was expanded to look for composite fusions. In this case, an exhaustive search was performed to look for two structural variants with break points close to the fusion break points and with an effective distance smaller than 250 kb.

Finally, 3,540 fusion events were included as the consensus fusion call set, from these 2,268 were detected by both FusionCatcher/STAR-Fusion and FusionMap (from these, 1,821 had matched structural variant evidence) and 1,112 were detected by only one method and had matched structural variance evidence.

In total, approximately 36% of all detected fusion transcripts were predicted to be in-frame, several UTR-mediated fusion transcripts preserve complete coding sequences of one fusion partner. These include a known fusion *TBL1XR1-PIK3CA* in a breast tumour and a notable new example *CTBP2-CTNNB1* in a gastric tumour.

All fusions are available in Synapse: https://dcc.icgc.org/releases/PCAWG/transcriptome/fusion.

### Identification of RNA-editing events

We used an RNA-editing events calling pipeline, which is an improved version of that previously published^108^. First, we summarized the base calls of pre-processed aligned RNA reads to the human reference in pileup format. Second, the initially identified editing sites were then filtered by the following quality-aware steps: (1) the depth of candidate editing site, base quality, mapping quality and the frequency of variation were taken into account to do a basic filter: the candidate variant sites should be with base-quality ≥ 20, mapping quality ≥ 50, mapped reads ≥ 4, variant-supporting reads ≥ 3, and mismatch frequencies (variant-supporting-reads/mapped-reads) ≥ 0.1. (2) Statistical tests based on the binomial distribution *B*(*n*, *p*) were used to distinguish true variants from sequencing errors on every mismatch site^109^, in which *p* denotes the background mismatch rate of each transcriptome sequencing, and *n* denotes sequencing depth on this site. (3) Discard the sites present in combined DNA SNP datasets (dbSNP v.138, 1000 Genome SNP phase 3, human Dutch populations^110^, and BGI in-house data; combined datasets deposited at: ftp://ftp.genomics.org.cn/pub/icgc-pcawg3). (4) Estimate strand bias and filter out variants with strand bias based on two-tailed Fisher’s exact test. (5) Estimate and filter out variants with position bias, such as sites only found at the 3′ end or at 5′ end of a read. (6) Discard the variation site in simple repeat region or homopolymer region or <5 bp from splicing site. (7) To reduce false positives introduced by misalignment of reads to highly similar regions of the reference genome, we performed a realignment filtering. Specifically, we extracted variant-supporting reads on candidate variant sites and realign them against a combination reference (hg19 genome plus Ensembl transcript reference v.75) by bwa0.5.9-r16. We retain a candidate variant site if at least 90% of its variant-supporting reads are realigned to this site. Finally, all high confident RNA-editing sites were annotated by ANNOVAR^111^. (8) To remove the possibility of an RNA-editing variant being a somatic variant, the variant sites are positionally filtered against PCAWG WGS somatic variant calls (9). The final two steps of filtering are designed to enrich the number of functional RNA editing sites. First, we keep only events that occur more than two times in at least one cancer type. Second, we keep only events that occur in exonic regions with a predicted function of missense, nonsense or stop-loss. The final step of filtering within exonic regions with a specific predicted function induces the largest difference in observed frequencies of RNA-editing events between our analysis and the published one^108^. A comparative depiction of the frequencies of RNA-editing events identified in our analysis (Supplementary Table 24) and the previously published analysis^108^ is seen in Supplementary Fig. 23.

### Gene-centric table creation

To perform joint analysis across RNA and DNA alterations, each alteration type was condensed into a binary gene-centric format. Because alterations occur at many different scales (nucleotide, exonic, gene or transcript), to make them comparable we projected each alteration type onto the gene body. We summarized each alteration type by its presence or absence within a single gene, yielding a binary value per type for each gene-sample pair.

The events we included in this analysis were: RNA editing, non-synonymous variants, expression, splicing alterations, copy-number alterations, fusions and alternative promoters. Each alteration type was summarized differently owing to their inherent differences.

RNA-editing events and non-synonymous variants can occur several times within a single gene body, so these events were denoted as 1 if they occurred at least once within a gene–sample pair.

For copy number, to obtain a single numerical value per gene-sample pair, the copy-number alteration was averaged over the gene body. Because we do not have matched normal samples against which to compare, we instead consider outlying events within each histotype as significant. Thus, a value of 1 was given to average copy-number alterations larger than 6 or smaller than 1.

Similar to non-synonymous variants, multiple splice events can occur within a gene body. The event with the most extreme PSI value within the gene body is selected as the candidate event for the gene. The candidate’s PSI value for a gene is compared over all samples within a histotype and it is set to 1 (that is, significant) only if it the absolute value of its *z*-score is larger than 6 and the standard deviation is larger than 0.01 within that histotype.

Similar to expression outliers, we calculate a *z*-score using the log-transformed upper-quartile normalized FPKM values with a pseudo-count of 1. All genes within a histotype with a standard deviation larger than zero and an absolute value larger than three were identified as an outlier. Alternative promoter outliers were calculated based on relative promoter activity within each cancer type. To binarize the promoter activity, a *z*-score cut-off of two over the relative expression distribution within each cancer type was used.

For ASE outliers, only genes with significant allelic imbalance (FDR ≤ 5% and allelic imbalance > 0.2, binomial test) were denoted as 1. All ASE events that were identified were further filtered to keep only genes that have not been identified as imprinted^26^.

In addition to the *z*-score-filtering mentioned above, we further filtered non-synonymous SNVs, RNA-editing events and splicing events such that they either induce a frameshift or the alternative region contains an HGMD variant^112^ of the category ‘damaging’.

It must be noted that in many cases, the *z*-score calculated is not from a Gaussian distribution, so some events may be missed or falsely included. Through our choice of very stringent *z*-score thresholds and functional filters, we hope that spurious outlier events are minimized.

### Pathway analysis

For our pathway analysis, we used the TCGA pathway definitions to examine genes and pathways that have several alterations at both the DNA and RNA level^113^.

### Co-occurrence analysis

The co-occurrence analysis was also performed on the aforementioned binarized gene-centric table, but only including variants, expression outliers, alternative promoters, alternative splicing and fusions. SCNA and ASE are excluded owing to a large number of anticipated co-occurrence. In this analysis, we required at least one gene of a given alteration pair to be a COSMIC gene. For each alteration pair, based on the number of donors with both alterations, one alteration only and neither alterations in a set of cancer samples, we performed Fisher’s exact test to determine whether the alteration pair was independent of each other. Such tests were followed by Benjamini–Hochberg multiple testing correction to obtain the FDR (or *q* values). To rule out the potential false-positive association caused by tissue-specific alterations, we performed the same analysis for each of the tumour types with at least 50 patients, and retained only those alteration pairs that were significantly associated in both the pan-cancer analysis and in at least one specific cancer indication. Among the significantly associated alteration pairs, the co-occurred pairs were those with odds ratio greater than 1. Pathway enrichment and visualization^21,114^ were conducted using the R package ReactomePA^79^. The circos plots were generated using the R package circlize^115^. The splicing related genes were derived from the genes annotated as ‘REACTOME_MRNA_SPLICING’ or ‘REACTOME_MRNA_SPLICING_MINOR_PATHWAY’ in the Molecular Signatures Database (MSigDB)^116^.

### Identifying genes with heterogeneous mechanisms of alterations in *cis*

Genes with multiple heterogeneous mechanisms of RNA alteration were identified from associations of *cis* variants with gene expression, ASE, fusions and splicing. For gene expression, genes associated with somatic eQTL with FDR < 5% were selected. For ASE, the top 5% of genes ranked by the predicted contribution of somatic variants on ASE. For fusions, all RNA fusions with structural variant support were selected. For splicing, genes having somatic mutations within 10 bp of an annotated splice site or 3 bp of a branch point and associated splicing were selected. These associated splicing events also had to have a |*z*-score| greater than or equal to 3 and the difference of percent spliced in the outlier event was greater than or equal to 10%.

### Recurrence analysis

The recurrence analysis was performed on the binarized gene-centric table for all nine alteration types. The recurrence analysis was performed in three main steps: (1) Aggregate within each alteration type across all samples. This results in a sum for each gene-alteration pair. (2) Convert the counts to ranks within each alteration. The smallest rank goes to the most frequently altered genes. Ranks are split evenly across ties. (3) To generate a single score for each gene, the second smallest rank across alterations is used as the score. To identify a score cut-off value for significantly altered genes, a null distribution was generated through permutation. The permutations were performed over the samples within each gene-alteration pair, this was done over all genes and samples 1,000 times, concatenating together all observations, results in 16.8 million permuted scores. *P* < 0.05 as derived from the null distribution was defined as significant, resulting in a score greater than or equal to 774 considered as significant.

WExT^117^ was used to test the significance of mutually exclusivity of RNA and DNA alterations. As further evidence that *CDK12* alterations may have a functional affect, we find evidence of the previously detected link^55^ between a large tandem duplicator phenotype (here defined as more than 10 tandem duplications of size greater than 100 kb) and *CDK12* somatic eQTL mutation (7 out of 18 somatic eQTL carriers are also among the 215 large tandem duplicator cases, *P* = 0.032, hypergeometric test).

### Statistical tests

All common statistical tests are two-sided unless otherwise specified. No statistical methods were used to predetermine sample size. The experiments were not randomized and investigators were not blinded to allocation during experiments and outcome assessment.

### Reporting summary

Further information on research design is available in the Nature Research Reporting Summary linked to this paper.

## Online content

Any methods, additional references, Nature Research reporting summaries, source data, extended data, supplementary information, acknowledgements, peer review information; details of author contributions and competing interests; and statements of data and code availability are available at 10.1038/s41586-020-1970-0.

## Supplementary information

Supplementary Information This file contains Supplementary Results, 26 Supplementary Figures, and Supplementary Notes.

Reporting Summary

Supplementary Tables This zipped file contains Supplementary Tables 1-24 and a Supplementary Table Guide.

Supplementary Information This file contains a full list of the PCAWG consortium.

## Figures and Tables

**Extended Data Table 1:**
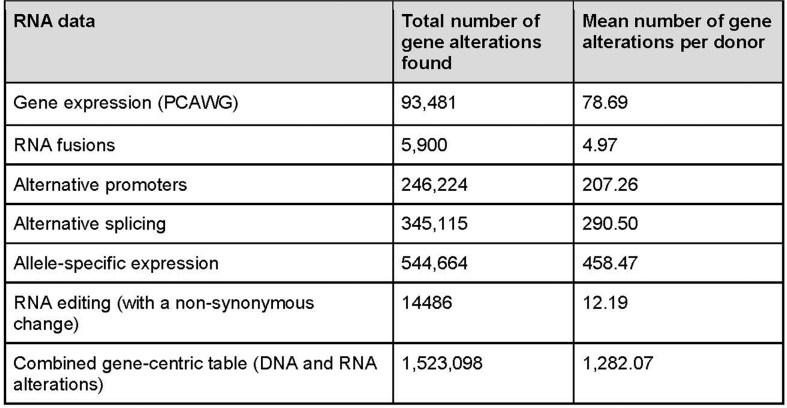
RNA alteration data

## Data Availability

Somatic and germline variant calls, mutational signatures, subclonal reconstructions, and other core data generated by the ICGC and TCGA PCAWG Consortium are described in an accompanying Article^5^ and are available for download at https://dcc.icgc.org/releases/PCAWG. Additional information on accessing the data, including raw read files, can be found at https://docs.icgc.org/pcawg/data/. In accordance with the data access policies of the ICGC and TCGA projects, most molecular, clinical and specimen data are in an open tier that does not require access approval. To access potentially identification information, such as germline alleles and underlying sequencing data, researchers will need to apply to the TCGA data access committee via dbGaP (https://dbgap.ncbi.nlm.nih.gov/aa/wga.cgi?page=login) for access to the TCGA portion of the dataset, and to the ICGC data access compliance office (http://icgc.org/daco) for the ICGC portion of the dataset. In addition, to access somatic SNVs derived from TCGA donors, researchers will also need to obtain dbGaP authorization. Data derived specifically from RNA-seq analysis can be found at https://dcc.icgc.org/releases/PCAWG/transcriptome. Subfolders contain identification and quantification of alternative promoter usage, alternative splicing, RNA fusions, gene expression, transcript-level expression and RNA editing. Identified eQTLs are in https://dcc.icgc.org/releases/PCAWG/transcriptome/eQTL and a binarized table indicating all RNA and DNA alterations for each gene can be found in the subfolder https://dcc.icgc.org/releases/PCAWG/transcriptome/recurrence_analyses/. In addition, quality-control metrics and metadata are also included. Some datasets are denoted with synXXXXX accession numbers and available at Synapse (https://www.synapse.org/).

## Code availability

The core computational pipelines used by the PCAWG Consortium for alignment, quality control and variant calling are available to the public at https://dockstore.org/search?search=pcawg under the GNU General Public License v.3.0, which allows for reuse and distribution. Further details on code availability are in the Supplementary Information.

## References

[1] Weinhold, N., Jacobsen, A., Schultz, N., Sander, C. & Lee, W. Genome-wide analysis of noncoding regulatory mutations in cancer. *Nat. Genet*. **46**, 1160–1165 (2014). 25261935 10.1038/ng.3101 PMC4217527

[2] Owens, M. A., Horten, B. C. & Da Silva, M. M. HER2 amplification ratios by fluorescence in situ hybridization and correlation with immunohistochemistry in a cohort of 6556 breast cancer tissues. *Clin. Breast Cancer* **5**, 63–69 (2004). 15140287 10.3816/cbc.2004.n.011

[3] Climente-González, H., Porta-Pardo, E., Godzik, A. & Eyras, E. The functional impact of alternative splicing in cancer. *Cell Reports* **20**, 2215–2226 (2017). 28854369 10.1016/j.celrep.2017.08.012

[4] Faderl, S. et al. The biology of chronic myeloid leukemia. *N. Engl. J. Med*. **341**, 164–172 (1999). 10403855 10.1056/NEJM199907153410306

[5] The ICGC/TCGA Pan-Cancer Analysis of Whole Genomes Network. Pan-cancer analysis of whole genomes. *Nature*10.1038/s41586-020-1969-6 (2020).

[6] Gerstung, M. et al. The evolutionary history of 2,658 cancers. *Nature*10.1038/s41586-019-1907-7 (2020). 10.1038/s41586-019-1907-7 PMC7054212 32025013

[7] Li, Y. et al. Patterns of somatic structural variation in human cancer genomes. *Nature*10.1038/s41586-019-1913-9 (2020). 10.1038/s41586-019-1913-9 PMC7025897 32025012

[8] Rheinbay, E. et al. Analyses of non-coding somatic drivers in 2,693 cancer whole genomes. *Nature*10.1038/s41586-020-1965-x (2020). 10.1038/s41586-020-1965-x PMC7054214 32025015

[9] Alexandrov, L. B. et al. The repertoire of mutational signatures in human cancer. *Nature*10.1038/s41586-020-1943-3 (2020). 10.1038/s41586-020-1943-3 PMC7054213 32025018

[10] GTEx Consortium. Genetic effects on gene expression across human tissues. *Nature* **550**, 204–213 (2017). 29022597 10.1038/nature24277 PMC5776756

[11] Kilpinen, H. et al. Common genetic variation drives molecular heterogeneity in human iPSCs. *Nature* **546**, 370–375 (2017). 28489815 10.1038/nature22403 PMC5524171

[12] Fredriksson, N. J., Ny, L., Nilsson, J. A. & Larsson, E. Systematic analysis of noncoding somatic mutations and gene expression alterations across 14 tumor types. *Nat. Genet*. **46**, 1258–1263 (2014). 25383969 10.1038/ng.3141

[13] Gong, J. et al. PancanQTL: systematic identification of *cis*-eQTLs and *trans*-eQTLs in 33 cancer types. *Nucleic Acids Res*. **46**, D971–D976 (2018). 29036324 10.1093/nar/gkx861 PMC5753226

[14] Bajrami, I. et al. Genome-wide profiling of genetic synthetic lethality identifies CDK12 as a novel determinant of PARP1/2 inhibitor sensitivity. *Cancer Res*. **74**, 287–297 (2014). 24240700 10.1158/0008-5472.CAN-13-2541 PMC4886090

[15] Havelange, V. et al. IRF4 mutations in chronic lymphocytic leukemia. *Blood* **118**, 2827–2829 (2011). 21791429 10.1182/blood-2011-04-350579 PMC3172799

[16] Roadmap Epigenomics Consortium et al. Integrative analysis of 111 reference human epigenomes. *Nature* **518**, 317–330 (2015). 25693563 10.1038/nature14248 PMC4530010

[17] Zheng, C. L. et al. Transcription restores DNA repair to heterochromatin, determining regional mutation rates in cancer genomes. *Cell Reports* **9**, 1228–1234 (2014). 25456125 10.1016/j.celrep.2014.10.031 PMC4254608

[18] Hanafusa, T., Mohamed, A. E. A., Domae, S., Nakayama, E. & Ono, T. Serological identification of Tektin5 as a cancer/testis antigen and its immunogenicity. *BMC Cancer* **12**, 520 (2012). 23151147 10.1186/1471-2407-12-520 PMC3522552

[19] Alexandrov, L. B. et al. Signatures of mutational processes in human cancer. *Nature* **500**, 415–421 (2013). 23945592 10.1038/nature12477 PMC3776390

[20] Milacic, M. et al. Annotating cancer variants and anti-cancer therapeutics in reactome. *Cancers (Basel)* **4**, 1180–1211 (2012). 10.3390/cancers4041180 PMC3712731 24213504

[21] Fabregat, A. et al. The Reactome pathway Knowledgebase. *Nucleic Acids Res*. **44**, D481–D487 (2016). 26656494 10.1093/nar/gkv1351 PMC4702931

[22] Kvam, E. & Tyrrell, R. M. The role of melanin in the induction of oxidative DNA base damage by ultraviolet A irradiation of DNA or melanoma cells. *J. Invest. Dermatol*. **113**, 209–213 (1999). 10469305 10.1046/j.1523-1747.1999.00653.x

[23] Jimbow, K., Chen, H., Park, J. S. & Thomas, P. D. Increased sensitivity of melanocytes to oxidative stress and abnormal expression of tyrosinase-related protein in vitiligo. *Br. J. Dermatol*. **144**, 55–65 (2001). 11167683 10.1046/j.1365-2133.2001.03952.x

[24] Pilger, A. & Rüdiger, H. W. 8-Hydroxy-2′-deoxyguanosine as a marker of oxidative DNA damage related to occupational and environmental exposures. *Int. Arch. Occup. Environ. Health* **80**, 1–15 (2006). 16685565 10.1007/s00420-006-0106-7

[25] Premi, S. & Brash, D. E. Unanticipated role of melanin in causing carcinogenic cyclobutane pyrimidine dimmers. *Mol. Cell. Oncol*. **3**, e1033588 (2015). 27308551 10.1080/23723556.2015.1033588 PMC4845191

[26] Morison, I. M., Ramsay, J. P. & Spencer, H. G. A census of mammalian imprinting. *Trends Genet*. **21**, 457–465 (2005). 15990197 10.1016/j.tig.2005.06.008

[27] Lindeboom, R. G. H., Supek, F. & Lehner, B. The rules and impact of nonsense-mediated mRNA decay in human cancers. *Nat. Genet*. **48**, 1112–1118 (2016). 27618451 10.1038/ng.3664 PMC5045715

[28] Demircioğlu, D. et al. A pan-cancer transcriptome analysis reveals pervasive regulation through alternative promoters. *Cell* **178**, 1465–1477.e17 (2019). 31491388 10.1016/j.cell.2019.08.018

[29] Reyes, A. & Huber, W. Alternative start and termination sites of transcription drive most transcript isoform differences across human tissues. *Nucleic Acids Res*. **46**, 582–592 (2018). 29202200 10.1093/nar/gkx1165 PMC5778607

[30] Feng, G. et al. Ubiquitously expressed genes participate in cell-specific functions via alternative promoter usage. *EMBO Rep*. **17**, 1304–1313 (2016). 27466324 10.15252/embr.201541476 PMC5007564

[31] Huang, F. W. et al. Highly recurrent *TERT* promoter mutations in human melanoma. *Science* **339**, 957–959 (2013). 23348506 10.1126/science.1229259 PMC4423787

[32] Oltean, S. & Bates, D. O. Hallmarks of alternative splicing in cancer. *Oncogene* **33**, 5311–5318 (2014). 24336324 10.1038/onc.2013.533

[33] Jung, H. et al. Intron retention is a widespread mechanism of tumor-suppressor inactivation. *Nat. Genet*. **47**, 1242–1248 (2015). 26437032 10.1038/ng.3414

[34] Kahles, A. et al. Comprehensive analysis of alternative splicing across tumors from 8,705 patients. *Cancer Cell* **34**, 211–224.e6 (2018). 30078747 10.1016/j.ccell.2018.07.001 PMC9844097

[35] Shiraishi, Y. et al. A comprehensive characterization of *cis*-acting splicing-associated variants in human cancer. *Genome Res*. **28**, 1111–1125 (2018). 30012835 10.1101/gr.231951.117 PMC6071634

[36] Sorek, R. The birth of new exons: mechanisms and evolutionary consequences. *RNA* **13**, 1603–1608 (2007). 17709368 10.1261/rna.682507 PMC1986822

[37] Mertens, F., Johansson, B., Fioretos, T. & Mitelman, F. The emerging complexity of gene fusions in cancer. *Nat. Rev. Cancer* **15**, 371–381 (2015). 25998716 10.1038/nrc3947

[38] Melé, M. et al. Human genomics. The human transcriptome across tissues and individuals. *Science* **348**, 660–665 (2015). 25954002 10.1126/science.aaa0355 PMC4547472

[39] Matsubara, D. et al. Identification of *CCDC6-RET* fusion in the human lung adenocarcinoma cell line, LC-2/ad. *J. Thorac. Oncol*. **7**, 1872–1876 (2012). 23154560 10.1097/JTO.0b013e3182721ed1

[40] Carneiro, B. A. et al. *FGFR3-TACC3*: a novel gene fusion in cervical cancer. *Gynecol Oncol Rep* **13**, 53–56 (2015). 26425723 10.1016/j.gore.2015.06.005 PMC4563584

[41] Lee, M. et al. ChimerDB 3.0: an enhanced database for fusion genes from cancer transcriptome and literature data mining. *Nucleic Acids Res*. **45** (D1), D784–D789 (2017). 27899563 10.1093/nar/gkw1083 PMC5210563

[42] Knezevich, S. R., McFadden, D. E., Tao, W., Lim, J. F. & Sorensen, P. H. A novel *ETV6-NTRK3* gene fusion in congenital fibrosarcoma. *Nat. Genet*. **18**, 184–187 (1998). 9462753 10.1038/ng0298-184

[43] Nacu, S. et al. Deep RNA sequencing analysis of readthrough gene fusions in human prostate adenocarcinoma and reference samples. *BMC Med. Genomics* **4**, 11 (2011). 21261984 10.1186/1755-8794-4-11 PMC3041646

[44] Jia, Y., Xie, Z. & Li, H. Intergenically spliced chimeric RNAs in cancer. Trends *Cancer* **2**, 475–484 (2016). 10.1016/j.trecan.2016.07.006 PMC5305119 28210711

[45] Greger, L. et al. Tandem RNA chimeras contribute to transcriptome diversity in human population and are associated with intronic genetic variants. *PLoS ONE* **9**, e104567 (2014). 25133550 10.1371/journal.pone.0104567 PMC4136775

[46] Tomlins, S. A. et al. Recurrent fusion of *TMPRSS2* and ETS transcription factor genes in prostate cancer. *Science* **310**, 644–648 (2005). 16254181 10.1126/science.1117679

[47] Koboldt, D. C. et al. VarScan 2: somatic mutation and copy number alteration discovery in cancer by exome sequencing. *Genome Res*. **22**, 568–576 (2012). 22300766 10.1101/gr.129684.111 PMC3290792

[48] Lawrence, M. S. et al. Mutational heterogeneity in cancer and the search for new cancer-associated genes. *Nature* **499**, 214–218 (2013). 23770567 10.1038/nature12213 PMC3919509

[49] Eilertsen, I. A. et al. Alternative splicing expands the prognostic impact of KRAS in microsatellite stable primary colorectal cancer. *Int. J. Cancer* **144**, 841–847 (2019). 30121958 10.1002/ijc.31809 PMC6587976

[50] Ji, X. et al. αCP binding to a cytosine-rich subset of polypyrimidine tracts drives a novel pathway of cassette exon splicing in the mammalian transcriptome. *Nucleic Acids Res*. **44**, 2283–2297 (2016). 26896798 10.1093/nar/gkw088 PMC4797308

[51] Stransky, N., Cerami, E., Schalm, S., Kim, J. L. & Lengauer, C. The landscape of kinase fusions in cancer. *Nat. Commun*. **5**, 4846 (2014). 25204415 10.1038/ncomms5846 PMC4175590

[52] Cancer Genome Atlas Research Network. Comprehensive molecular profiling of lung adenocarcinoma. *Nature* **511**, 543–550 (2014). 25079552 10.1038/nature13385 PMC4231481

[53] Forbes, S. A. et al. COSMIC: somatic cancer genetics at high-resolution. *Nucleic Acids Res*. **45** (D1), D777–D783 (2017). 27899578 10.1093/nar/gkw1121 PMC5210583

[54] Blazek, D. et al. The Cyclin K/Cdk12 complex maintains genomic stability via regulation of expression of DNA damage response genes. *Genes Dev*. **25**, 2158–2172 (2011). 22012619 10.1101/gad.16962311 PMC3205586

[55] Menghi, F. et al. The tandem duplicator phenotype is a prevalent genome-wide cancer configuration driven by distinct gene mutations. *Cancer Cell* **34**, 197–210.e5 (2018). 30017478 10.1016/j.ccell.2018.06.008 PMC6481635

[56] Dawson, M. A. & Kouzarides, T. Cancer epigenetics: from mechanism to therapy. *Cell* **150**, 12–27 (2012). 22770212 10.1016/j.cell.2012.06.013

[57] Zhang, X. et al. Identification of focally amplified lineage-specific super-enhancers in human epithelial cancers. *Nat. Genet*. **48**, 176–182 (2016). 26656844 10.1038/ng.3470 PMC4857881

[58] Dobin, A. et al. STAR: ultrafast universal RNA-seq aligner. *Bioinformatics* **29**, 15–21 (2013). 10.1093/bioinformatics/bts635 PMC3530905 23104886

[59] Kim, D. et al. TopHat2: accurate alignment of transcriptomes in the presence of insertions, deletions and gene fusions. *Genome Biol*. **14**, R36 (2013). 23618408 10.1186/gb-2013-14-4-r36 PMC4053844

[60] Harrow, J. et al. GENCODE: the reference human genome annotation for The ENCODE Project. *Genome Res*. **22**, 1760–1774 (2012). 22955987 10.1101/gr.135350.111 PMC3431492

[61] Fonseca, N. A., Petryszak, R., Marioni, J. & Brazma, A. iRAP - an integrated RNA-seq analysis pipeline. Preprint at https://www.bioRxiv.org/content/10.1101/005991v1 (2014).

[62] Bioinformatics, B. FastQC: a quality control tool for high throughput sequence data; http://www.bioinformatics.babraham.ac.uk/projects/fastqc (2011).

[63] Cancer Genome Atlas Research Network. The molecular taxonomy of primary prostate cancer. *Cell* **163**, 1011–1025 (2015). 26544944 10.1016/j.cell.2015.10.025 PMC4695400

[64] Li, H. et al. The Sequence Alignment/Map format and SAMtools. *Bioinformatics* **25**, 2078–2079 (2009). 19505943 10.1093/bioinformatics/btp352 PMC2723002

[65] Anders, S., Pyl, P. T. & Huber, W. HTSeq—a Python framework to work with high-throughput sequencing data. *Bioinformatics* **31**, 166–169 (2015). 25260700 10.1093/bioinformatics/btu638 PMC4287950

[66] Bray, N. L., Pimentel, H., Melsted, P. & Pachter, L. Near-optimal probabilistic RNA-seq quantification. *Nat. Biotechnol*. **34**, 525–527 (2016). 27043002 10.1038/nbt.3519

[67] Mortazavi, A., Williams, B. A., McCue, K., Schaeffer, L. & Wold, B. Mapping and quantifying mammalian transcriptomes by RNA-Seq. *Nat. Methods* **5**, 621–628 (2008). 18516045 10.1038/nmeth.1226 PMC13303166

[68] Krijthe, J. H. Rtsne: t-distributed stochastic neighbor embedding using barnes-hut implementation; https://github.com/jkrijthe/Rtsne (2015).

[69] Dentro, S. C. et al. Portraits of genetic intra-tumour heterogeneity and subclonal selection across cancer types. Preprint at https://www.biorxiv.org/content/10.1101/312041v4 (2018).

[70] Kahles, A., Ong, C. S., Zhong, Y. & Rätsch, G. *SplAdder*: identification, quantification and testing of alternative splicing events from RNA-Seq data. *Bioinformatics* **32**, 1840–1847 (2016). 26873928 10.1093/bioinformatics/btw076 PMC4908322

[71] Pedregosa, F. et al. Scikit-learn: machine learning in python. *J. Mach. Learn. Res*. **12**, 2825–2830 (2011).

[72] Stegle, O., Parts, L., Piipari, M., Winn, J. & Durbin, R. Using probabilistic estimation of expression residuals (PEER) to obtain increased power and interpretability of gene expression analyses. *Nat. Protocols* **7**, 500–507 (2012). 22343431 10.1038/nprot.2011.457 PMC3398141

[73] GTEx Consortium. The Genotype-Tissue Expression (GTEx) project. *Nat. Genet*. **45**, 580–585 (2013). 23715323 10.1038/ng.2653 PMC4010069

[74] The Gene Ontology Consortium. Gene Ontology: tool for the unification of biology. *Nat. Genet*. **25**, 25–29 (2000). 10802651 10.1038/75556 PMC3037419

[75] The Gene Ontology Consortium. Gene Ontology Consortium: going forward. *Nucleic Acids Res*. **43**, D1049–D1056 (2015). 25428369 10.1093/nar/gku1179 PMC4383973

[76] Durinck, S., Spellman, P. T., Birney, E. & Huber, W. Mapping identifiers for the integration of genomic datasets with the R/Bioconductor package biomaRt. *Nat. Protocols* **4**, 1184–1191 (2009). 19617889 10.1038/nprot.2009.97 PMC3159387

[77] Durinck, S. et al. BioMart and Bioconductor: a powerful link between biological databases and microarray data analysis. *Bioinformatics* **21**, 3439–3440 (2005). 16082012 10.1093/bioinformatics/bti525

[78] Yu, G., Wang, L.-G., Han, Y. & He, Q.-Y. clusterProfiler: an R package for comparing biological themes among gene clusters. *OMICS* **16**, 284–287 (2012). 22455463 10.1089/omi.2011.0118 PMC3339379

[79] Yu, G. & He, Q.-Y. ReactomePA: an R/Bioconductor package for reactome pathway analysis and visualization. *Mol. Biosyst*. **12**, 477–479 (2016). 26661513 10.1039/c5mb00663e

[80] Li, H. A statistical framework for SNP calling, mutation discovery, association mapping and population genetical parameter estimation from sequencing data. *Bioinformatics* **27**, 2987–2993 (2011). 21903627 10.1093/bioinformatics/btr509 PMC3198575

[81] Lippert, C., Casale, F. P., Rakitsch, B. & Stegle, O. LIMIX: genetic analysis of multiple traits. Preprint at https://www.bioRxiv.org/content/ 10.1101/003905v2 (2014).

[82] Davis, J. R. et al. An efficient multiple-testing adjustment for eQTL studies that accounts for linkage disequilibrium between variants. *Am. J. Hum. Genet*. **98**, 216–224 (2016). 26749306 10.1016/j.ajhg.2015.11.021 PMC4716687

[83] Pers, T. H., Timshel, P. & Hirschhorn, J. N. SNPsnap: a Web-based tool for identification and annotation of matched SNPs. *Bioinformatics* **31**, 418–420 (2015). 25316677 10.1093/bioinformatics/btu655 PMC4308663

[84] Fan, Y. et al. MuSE: accounting for tumor heterogeneity using a sample-specific error model improves sensitivity and specificity in mutation calling from sequencing data. *Genome Biol*. **17**, 178 (2016). 27557938 10.1186/s13059-016-1029-6 PMC4995747

[85] Quinlan, A. R. & Hall, I. M. BEDTools: a flexible suite of utilities for comparing genomic features. *Bioinformatics* **26**, 841–842 (2010). 20110278 10.1093/bioinformatics/btq033 PMC2832824

[86] Corces, M. R. et al. The chromatin accessibility landscape of primary human cancers. *Science* **362**, 362 (2018). 10.1126/science.aav1898 PMC6408149 30361341

[87] Zhang, W. et al. A global transcriptional network connecting noncoding mutations to changes in tumor gene expression. *Nat. Genet*. **50**, 613–620 (2018). 29610481 10.1038/s41588-018-0091-2 PMC5893414

[88] Smith, K. S. et al. Signatures of accelerated somatic evolution in gene promoters in multiple cancer types. *Nucleic Acids Res*. **43**, 5307–5317 (2015). 25934800 10.1093/nar/gkv419 PMC4477653

[89] Fishilevich, S. et al. GeneHancer: genome-wide integration of enhancers and target genes in GeneCards. *Database* **2017**, 2017 (2017). 10.1093/database/bax028 PMC5467550 28605766

[90] Haeussler, M. et al. The UCSC Genome Browser database: 2019 update. *Nucleic Acids Res*. **47**, D853–D858 (2019). 30407534 10.1093/nar/gky1095 PMC6323953

[91] ENCODE Project Consortium. An integrated encyclopedia of DNA elements in the human genome. *Nature* **489**, 57–74 (2012). 22955616 10.1038/nature11247 PMC3439153

[92] Wang, C. et al. Systematic identification of genes with a cancer-testis expression pattern in 19 cancer types. *Nat. Commun*. **7**, 10499 (2016). 26813108 10.1038/ncomms10499 PMC4737856

[93] Wallace, C. Statistical testing of shared genetic control for potentially related traits. *Genet. Epidemiol*. **37**, 802–813 (2013). 24227294 10.1002/gepi.21765 PMC4158901

[94] Baron, R. M. & Kenny, D. A. The moderator-mediator variable distinction in social psychological research: conceptual, strategic, and statistical considerations. *J. Pers. Soc. Psychol*. **51**, 1173–1182 (1986). 3806354 10.1037//0022-3514.51.6.1173

[95] Preacher, K. J. & Hayes, A. F. SPSS and SAS procedures for estimating indirect effects in simple mediation models. *Behav. Res. Methods Instrum. Comput*. **36**, 717–731 (2004). 15641418 10.3758/bf03206553

[96] Rosseel, Y. lavaan: AnRPackage for structural equation modeling. *J. Stat. Softw*. **48**, 2 (2012).

[97] Tingley, D., Yamamoto, T., Hirose, K., Keele, L. & Imai, K. mediation:RPackage for causal mediation analysis. *J. Stat. Softw*. **59**, 5 (2014).

[98] Howie, B. N., Donnelly, P. & Marchini, J. A flexible and accurate genotype imputation method for the next generation of genome-wide association studies. *PLoS Genet*. **5**, e1000529 (2009). 19543373 10.1371/journal.pgen.1000529 PMC2689936

[99] Nik-Zainal, S. et al. The life history of 21 breast cancers. *Cell* **149**, 994–1007 (2012). 22608083 10.1016/j.cell.2012.04.023 PMC3428864

[100] McLaren, W. et al. The Ensembl variant effect predictor. *Genome Biol*. **17**, 122 (2016). 27268795 10.1186/s13059-016-0974-4 PMC4893825

[101] Castel, S. E., Levy-Moonshine, A., Mohammadi, P., Banks, E. & Lappalainen, T. Tools and best practices for data processing in allelic expression analysis. *Genome Biol*. **16**, 195 (2015). 26381377 10.1186/s13059-015-0762-6 PMC4574606

[102] Subramanian, A. et al. Gene set enrichment analysis: a knowledge-based approach for interpreting genome-wide expression profiles. *Proc. Natl Acad. Sci. USA* **102**, 15545–15550 (2005). 16199517 10.1073/pnas.0506580102 PMC1239896

[103] Frith, M. C. et al. A code for transcription initiation in mammalian genomes. *Genome Res*. **18**, 1–12 (2008). 18032727 10.1101/gr.6831208 PMC2134772

[104] Signal, B., Gloss, B. S., Dinger, M. E. & Mercer, T. R. Machine learning annotation of human branchpoints. *Bioinformatics* **34**, 920–927 (2018). 29092009 10.1093/bioinformatics/btx688

[105] Mercer, T. R. et al. Genome-wide discovery of human splicing branchpoints. *Genome Res*. **25**, 290–303 (2015). 25561518 10.1101/gr.182899.114 PMC4315302

[106] Ge, H. et al. FusionMap: detecting fusion genes from next-generation sequencing data at base-pair resolution. *Bioinformatics* **27**, 1922–1928 (2011). 21593131 10.1093/bioinformatics/btr310

[107] Nicorici, D. et al. FusionCatcher - a tool for finding somatic fusion genes in paired-end RNA-sequencing data. Preprint at https://www.bioRxiv.org/content/10.1101/011650v1 (2014).

[108] Han, L. et al. The Genomic Landscape and Clinical Relevance of A-to-I RNA Editing in Human Cancers. *Cancer Cell* **28**, 515–528 (2015). 26439496 10.1016/j.ccell.2015.08.013 PMC4605878

[109] Li, Q. et al. Caste-specific RNA editomes in the leaf-cutting ant *Acromyrmex echinatior*. *Nat. Commun*. **5**, 4943 (2014). 25266559 10.1038/ncomms5943 PMC4200514

[110] Genome of the Netherlands Consortium. Whole-genome sequence variation, population structure and demographic history of the Dutch population. *Nat. Genet*. **46**, 818–825 (2014). 24974849 10.1038/ng.3021

[111] Wang, K., Li, M. & Hakonarson, H. ANNOVAR: functional annotation of genetic variants from high-throughput sequencing data. *Nucleic Acids Res*. **38**, e164 (2010). 20601685 10.1093/nar/gkq603 PMC2938201

[112] Stenson, P. D. et al. The Human Gene Mutation Database: towards a comprehensive repository of inherited mutation data for medical research, genetic diagnosis and next-generation sequencing studies. *Hum. Genet*. **136**, 665–677 (2017). 28349240 10.1007/s00439-017-1779-6 PMC5429360

[113] Sanchez-Vega, F. et al. Oncogenic signaling pathways in the cancer genome atlas. *Cell* **173**, 321–337.e10 (2018). 29625050 10.1016/j.cell.2018.03.035 PMC6070353

[114] Merico, D., Isserlin, R., Stueker, O., Emili, A. & Bader, G. D. Enrichment map: a network-based method for gene-set enrichment visualization and interpretation. *PLoS ONE* **5**, e13984 (2010). 21085593 10.1371/journal.pone.0013984 PMC2981572

[115] Gu, Z., Gu, L., Eils, R., Schlesner, M. & Brors, B. circlize implements and enhances circular visualization in R. *Bioinformatics* **30**, 2811–2812 (2014). 24930139 10.1093/bioinformatics/btu393

[116] Liberzon, A. et al. Molecular signatures database (MSigDB) 3.0. *Bioinformatics* **27**, 1739–1740 (2011). 21546393 10.1093/bioinformatics/btr260 PMC3106198

[117] Leiserson, M. D. M., Reyna, M. A. & Raphael, B. J. A weighted exact test for mutually exclusive mutations in cancer. *Bioinformatics* **32**, i736–i745 (2016). 27587696 10.1093/bioinformatics/btw462 PMC5013919

[118] Rafnar, T. et al. Sequence variants at the *TERT-CLPTM1L* locus associate with many cancer types. *Nat. Genet*. **41**, 221–227(2009). 19151717 10.1038/ng.296 PMC4525478

[119] Bojesen, S. E. et al. Multiple independent variants at the *TERT* locus are associated with telomere length and risks of breast and ovarian cancer. *Nat. Genet*. **45**, 371–384 (2013). 23535731 10.1038/ng.2566 PMC3670748

[120] Ye, K. et al. Systematic discovery of complex insertions and deletions in human cancers. *Nat. Med*. **22**, 97–104 (2016). 26657142 10.1038/nm.4002 PMC5003782

