# Supplementary material for: Genomic basis for RNA alterations in cancer
Source: Nature. 2020 Feb 5;578(7793):129–36. doi: 10.1038/s41586-020-1970-0 (PMC7054216; doi:10.1038/s41586-020-1970-0)
Supplement: Supplementary file 1 — This file contains Supplementary Results, 26 Supplementary Figures, and Supplementary Notes. [file 41586_2020_1970_MOESM1_ESM.pdf]

nature

https://doi.org/10.1038/s41586-020-1970-0

Supplementary information

Genomic basis for RNA alterations in cancer

In the format provided by the authors and unedited

Supplementary Information

Genomic basis of RNA alterations in cancer.

PCAWG Transcriptome Working Group

Supplementary Results 2
- Unified data processing for PCAWG RNA sequencing data 2
- Cancer-specific cis germline regulatory variants highlight changes in regulatory landscape 2
- Somatic cis eQTL mapping in PCAWG 3
- Associations between somatic mutational signatures and gene expression 4
- Allele-specific expression captures cancer-specific dysregulation 5
- Promoter mutations associated with changes in promoter activity 7
- Intronic mutations associated with splicing and exonization 7
- Patterns of gene fusions across cancers 8
- Pan-cancer unified analysis reveal diverse modes of RNA-level alterations 9
- Co-occurrence of RNA and DNA alterations 10
- Known and novel candidate driver genes are recurrently altered at the RNA-level 11

Supplementary code availability 13

References 15

Supplementary Results

Unified data processing for PCAWG RNA sequencing data

For a unified transcriptome analysis, we processed all tumor samples with RNA-Seq data from the PCAWG consortium provided by 30 TCGA/ICGC projects. To harmonize these data across studies, we reanalyzed a total of 2,217 RNA-Seq libraries using a unified RNA-Seq analysis pipeline developed for this project (see **Software Availability**). Core components of this pipeline were spliced alignment of RNA-Seq data followed by gene expression quantification (**Supplementary Figure 1a**). We compared alternative alignment strategies using STAR[1] and TopHat2[2] (**Supplementary Figure 1a**), which yielded highly consistent gene expression quantifications (gene-level counts based on HTSeq[3], **Supplementary Figure 2a**). Thus, we generated consensus gene expression measurements by averaging read counts for each gene, normalized by gene length, followed by upper-quartile normalization (FPKM-UQ)[4,5] (**Supplementary Figure 2b**). FPKM-UQ quantification across the subset of TCGA samples were highly correlated (median correlation 0.95) with TCGA-reported gene expression using RSEM[6] (**Supplementary Figure 3**). Transcript isoform-specific expression levels were estimated using Kallisto[7].

After quality control filtering and merging of technical replicates, we obtained 1,359 RNA-seq profiles from 1,188 unique patients (**Supplementary Figure 4**), with between two and 154 samples per histotype (**Extended Data Figure 1a**) and approximately equal numbers of male and female patients (**Extended Data Figure 1b**). For 13 out of the 27 histotypes, matched, adjacent normal tissue samples were available, giving rise to a total of 150 normal tissue samples (**Extended Data Figure 1a,c)**. For additional normal coverage, we processed RNA-Seq data from 3,274 samples from the Genotype-Tissue Expression (GTEx) Consortium (version phs000424.v4.p1). We used the same computational pipeline used for the PCAWG RNA-seq dataset and applied quantile normalization. While we observed differences between GTEx and PCAWG samples, likely due to differences in processing methods and batch variation, we found that overall the tissue dominates the expression patterns (**Supplementary Figure 5, Supplementary Figure 5a**). For eQTL analysis we generated an adjusted expression dataset using PEER[8] to account for unknown and technical covariates). Tumor purity varied across samples and was considered an additional covariate of expression patterns (**Supplementary Figure 6**).

Cancer-specific *cis* germline regulatory variants highlight changes in regulatory landscape

Aside from the pan-cancer analysis we also identified germline eQTLs in individual tissues across the seven histotypes with 60 or more patients. We identified between 118 (Breast-AdenoCA) and 551 eGenes (Lymph-CLL) (**Supplementary Figure 7a, Supplementary Table 2**). We estimated the fraction of shared eQTLs across histotypes (**Supplementary Figure 7b,c**) and assessed the

overlap of lead eQTL variants with Epigenetics Roadmap annotations[9] (**Supplementary Figure 7b-d**), identifying an enrichment of active regulatory marks.

Our comparison of PCAWG-eQTL's to GTEx eQTLs revealed interesting examples like *SLAMF9*, which is a member of the CD2 subfamily with known roles in immune response and cancer[10] (**Supplementary Figure 8a**). More generally, this set of genes with PCAWG-specific eQTLs was enriched for cancer-relevant pathways (**Supplemental Figure 8b**, **Supplementary Table 4**). Only a small fraction of these PCAWG-specific eQTLs were in overexpressed genes compared to matched normal tissues (94/422 genes with >= 2-fold increase in expression). These genes were enriched for immunoglobulin genes (27/94) and cancer/testis antigen encoding genes (CT genes; 8/94; **Supplementary Figure 8c**). Overall, this analysis suggests that the germline framework of gene expression regulation is largely conserved in cancer tissues with no notable difference in distance to TSS, p-Value and effect size distribution between PCAWG specific eQTL's and unspecific eQTL's (**Supplementary Figure 9).**

Somatic *cis* eQTL mapping in PCAWG

We set out to systematically characterize somatic eQTL mapping in the PCAWG dataset. There was a low allele frequency for individual somatic mutations (86 SNVs with a recurrence frequency >1%); therefore we considered aggregated burdens of somatic SNVs across different regions of the whole genome. While non-coding driver elements have been identified in the PCAWG cohort[11] using whole-genome sequencing data, only in a few cases these have also been associated with changes in expression of neighbouring genes. Therefore, we here looked at possible somatic DNA changes, across the whole genome, that underlie gene expression alterations. Before performing a more in depth functional characterization of the somatic eQTL identified, we compared alternative strategies to quantify local mutation burdens, assessing the number of somatic eQTL discoveries. We found that weighting individual SNVs by their variant allele frequency (VAF) yielded the largest number of discoveries (**Supplementary Figure 10).**

We therefore focused only on the set of somatic eQTL (N=649) identified by associating VAF-based burdens with gene expression. We assessed the mapping resolution of this analysis, and found that most somatic eQTL could be linked to a single genomic region or a small number of them (median resolution of 1kb; **Extended Data Figure 6d**). Also, mutation burdens in association with gene expression tended to be mutated across multiple cancers (**Supplementary Figure 11, Supplementary Table 5**). We did not find any relationship between structural variants (SVs) and associated burdens (**Supplementary Notes; Supplementary Table 5**) and only 4% of somatic eGenes (28/649) with at least one adjacent PCAWG non-coding driver element [11], suggesting that only a small fraction of somatic eQTLs can be linked to putative driver mutations. We also looked at the functional characterization of somatic eGenes and found that cancer-testis (CT) genes were marginally more frequent among genes with somatic eQTLs than expected (45/982, P=0.07, Fisher's exact test).

Unlike the germline eQTL map, we also observed a larger number of associations distal to the TSS (≥20kb, 88%), which were primarily associated with rare burdens (<5% frequency, 97%; **Supplementary Figure 12**). Distances to TSS showed a weak correlation with eQTL effects (Spearman rho = -0.11; P = 0.006), with the strongest effects located very far from a TSS (>40.5kb). This is consistent with another large-scale study that also found no correlation between distances to a TSS and the effect size of somatic eQTLs[12]. Although this could point to mechanisms of regulation specific to somatic SNVs, this pattern is quite unexpected for *cis* associations and warrants further studies. Indeed, despite the stringent correction we performed by adding multiple known covariates as fixed effects in the association model, we cannot fully rule out further confounding effects from unpredicted factors acting on specific cancer types like, for example, somatic hypermutations in lymphomas[13], that might have led to spurious associations.

Finally, we determined the fraction of somatic eQTLs identified in previous studies and overlapping interactions detected with independent methods[12,14,15]. We found a small but significant overlap with GeneHancer[16] interactions and our somatic eQTLs (33/649, P = 0.001), some of which were also replicated in previous studies (**Supplementary Table 5).**

Overall, disentangling the causal hierarchies between gene expression changes and non-coding mutations remains still a great challenge in somatic eQTL analysis and further investigations will be required to validate our findings.

Associations between somatic mutational signatures and gene expression

We identified global *trans* associations between mutational signatures and gene expression levels and derived *de novo* annotations of signatures with previously unknown roles. We tested for association between signature prevalence in patients and total gene expression, accounting for total mutational burden, cancer type and other technical and biological confounders, and most importantly, for cancer type of the patients, which ensures that the detected associations do not only reflect differences between cancer types. This identified 1,176 genes associated with at least one signature (FDR ≤ 10%, **Extended Data Figure 10**, **Supplementary Table 19**), a markedly different set of genes compared to associations with total mutational burden alone (**Supplementary Table 19**). Lymphoma Signature 9 showed the largest number of associations, followed by the smoking-related Signature 4 (**Figure 1d, Supplementary Table 19**).

To annotate signatures which were not fully characterized, we considered 18 signatures with 20 or more associated genes (**Extended Data Figure 11**) and assessed enrichment using GO categories[17,18] and Reactome Pathways[17,18]. We found that 11 signatures were enriched for at least one category (FDR ≤ 10%, **Supplementary Table 19**), revealing associations consistent with known aetiologies (**Figure 1d**). For example, Lymphoma Signature 9 was associated with

354 genes enriched for lymphocyte/leukocyte-related processes and immune response, including *TCL1A*, *LMO2* and *TERT* ($P=1.2 \cdot 10^{-10}$, $6.8 \cdot 10^{-10}$, $2.0 \cdot 10^{-09}$). The smoking Signature 4 was associated with 119 genes enriched for biological oxidation processes, including *CYP24A1*, a gene that is known to be down-regulated in tobacco-smoke exposed tissue[19] (**Supplementary Figure 13a**). The 70 genes associated with APOBEC Signature 2 were significantly enriched for DNA deaminase pathways, and included the key APOBEC pathway-related genes *APOBEC3B* and *APOBEC3A* (**Extended Data Figure 11e-f**). We also found associations of signatures with unknown aetiology, including associations between Signature 38 and melanin processes (**Figure 1d**), and between Signature 8, which has been found to be prevalent in medulloblastoma, and 25 genes enriched for ABCA-transporter pathways. Drugs targeting these pathways are currently in clinical trials for treating medulloblastoma[18,20].

We then utilized germline eQTL lead variants of signature-associated genes as an anchor to gain directed mechanistic insight by testing for associations between these variants and the signature. This eQTL-based approach entails substantially fewer tests than genome-wide analyses[21,22]. Among 1,176 signature-linked genes, 197 had a germline eQTL. We found *APOBEC3A/B* eQTL rs12628403 to be associated with the corresponding Signature 2 ($P=5.1 \cdot 10^{-7}$, **Supplementary Figure 13**, FDR ≤ 10%, multiple testing over 197 tests, **Supplementary Table 19**), confirming it as a risk variant for Signature 2 prevalence[23]. Colocalisation[24] and mediation[25,26] analyses confirmed the variant as a plausible genetic determinant of *APOBEC3A/B* expression and Signature 2 prevalence (**Supplementary Table 19**), with a remarkable 87.11% of the genetic effect conferred to the signature by *APOBEC3B* expression (**Supplementary Figure 14**).

In summary, we identified global *trans* associations between mutational signatures and gene expression levels and thereby derived *de novo* annotations of signatures with previously unknown roles.

Allele-specific expression captures cancer-specific dysregulation

We quantified allele-specific expression (ASE), adapting established quality control steps to cancer tissues[27] and pooled ASE counts across heterozygous variants within genes to maximize detection power. This allowed us to quantify ASE for between 588 and 7,728 genes per patient (median=4,112 genes with 15 or more ASE reads; considering data from 1,120 patients, **Extended Data Figure 12**).

To robustly identify genes with haplotype-specific dysregulation, we considered ASE[27,28] to test for allelic expression imbalance (AEI) (FDR ≤ 5%, binomial test, Methods). Across the cohort, we observed substantial differences in the fraction of genes with AEI between cancer types (**Extended Data Figures 12**), and between cancer and the corresponding normal tissues, with a high concordance between allelic imbalance on the DNA and RNA levels - owing to SCNAs as we will show in the following (**Extended Data Figure 13**).

We used a logistic regression model to identify the determinants of AEI, accounting for known imprinting status[29], the germline eQTL genotype, SCNAs and the weighted mutational burden of proximal somatic SNVs stratified into functional categories (**Extended Data Figure 2**). We additionally correct for sample purity, the number of accessible ASE sites per gene and both gene-level and sample-level read depth. While cumulatively, non-coding variants were more relevant than coding variants, somatic protein truncating variants ('stop-gained') triggering nonsense-mediated decay[28] were the most predictive individually (**Figure 1e**). This was confirmed by a quantitative model on ASE ratios (**Extended Data Figure 14**). SNVs within splice regions, 5' UTR and promoters were also strongly associated with AEI presence and we observed a global trend of decreasing relevance of variants with increasing distance from the TSS (**Figure 1e**).

Our model allows for attributing AEI to germline SNPs, SCNAs and somatic SNVs by computing average scores derived from predicting AEI individually from SNPs, SCNAs and SNVs (**Supplementary Table 9**). The average prediction score identified somatic AEI as predictive for genes with relevance in cancer (**Supplementary Figure 15a**), which consisted of mostly known cancer genes but also a few genes not previously associated with cancer, such as *EXO1*, a mismatch repair-related gene. *EXO1* exhibited significant AEI for both a potentially deleterious missense and a nonsense mutation in a colorectal adenocarcinoma sample. TCGA colorectal adenocarcinoma patients with lower expression of *EXO1* showed worse overall survival (Log rank P=0.022, HR=0.57, **Supplementary Figure 15b**), implicating *EXO1* as a potential tumor suppressor in colorectal cancer. When calculating fraction of tumour samples with AEI for all candidate PCAWG driver genes[11], we found that driver genes tended to exhibit higher percentages of AEI in the cancer types they influence (Wilcoxon Rank Sum Test, P=3.64·10-9, **Supplementary Figure 15c**), implying AEI as a common mechanism for cancer genes to exert their functions. However, not all mutations in a given gene exhibit the same AEI properties. For *TP53*, almost all known hotspot mutations showed uniformly high mutant allele selectivity across cancer indications. By contrast, for *PIK3CA*, the H1047R mutation exhibited strong mutant allele preference in breast and lung cancers, while the E545K mutation only showed mutant allele selectivity in melanoma (**Supplementary Figure 15d**). Such AEI pattern differences could account for some of the phenotypic variability of cancer mutations in different cancer types.

Motivated by the observed cancer-specific germline regulation of CT genes, we also used these model components to investigate sources of AEI in CT genes. Notably, CT genes were depleted when considering the full somatic score including SCNAs (25/476 CT genes in the top 10% of genes, 48 expected, χ^2 test, P=6·10-4), but enriched in the AEI score based on SNVs only (66/476 CT genes in the top 10% of genes, 48 expected, χ^2test, P=6·10-3). One potential explanation is that repressed CT genes have to undergo somatic re-activation by SNVs before CN amplification. To elucidate this, we used mutation timing data[30], stratifying SNVs into the categories early and late (SNV occurred before and after SCNA at the same locus, respectively) and found strong over-representation of early SNVs in 329 out of 7,525 CT gene-patient pairs (216 expected, χ^2test, P=4·10-14).

In summary, we identify somatic and germline genetic variation associated with allele-specific dysregulation of genes across cancer types. We demonstrate the power of ASE as an integrator of different sources of transcriptional dysregulation in *cis*, and as a sensitive readout for

identification of novel tissue-specific candidate driver genes. In particular, our analysis suggests recurrent somatic reactivation of CT genes, warranting further investigation into their role in carcinogenesis and tumour progression.

Promoter mutations associated with changes in promoter activity

Estimation of transcript abundance is less robust compared to gene-level expression estimation, in particular, 3' bias and sequencing depth contribute to technical variation (**Extended Data Figure 15a-c**). While there is increased uncertainty for individual promoters compared to gene expression, promoter activity estimates in samples from the same cancer type show high levels of similarity [31]. Across all samples we identified 44,586 active promoters (FPKM > 0.1 in at least 1% of the patient cohort).

Intronic mutations associated with splicing and exonization

Exome-only studies are limited in identifying mutations that could affect splicing due to low read support deep in the intron. To further investigate the role of splicing aberrations within the context of whole genome variation, we identified and quantified alternative splicing using *SplAdder*[32], focussing on six splicing events types. We found an increase of unannotated alternative splicing events in tumor samples compared to non-tumor samples; for example, there are ≈30% more detected cassette exon events in liver tumor samples than in matched normals or tissue matched GTEx samples (316,522 tumor, 279,148 normal, 234,710 GTEx; **Extended Data Figure 17a**). In total, *SplAdder* detected 595,041 alternative 3' splice site, 386,734 alternative 5' splice site, 1,226,253 cassette exon, 755,589 intron retention/novel intron, 47,889 coordinated exon skip and 505,515 mutually exclusive exon events in at least one sample of the cohort with Lymph-BNHL, Lymph-CLL, and Ovary-AdenoCA having the most novel events **(Extended Data Figure 17b).** While splicing of samples from the same histotype covaries, we observe differences between GTEx and PCAWG cohorts **(Extended Data Figure 17c)**.

Although it is known that *trans* factors that assist in branch site recognition, like *SF3B1*, are recurrently mutated in various cancer types[33–36], a pan-cancer analysis of the impact of branch site associated mutations in *cis* has not been performed. Further, we measured positive selection for somatic mutations associated with splicing alterations at a gene-level using a permutation test. Our analysis recovered two known tumor suppressor genes, *TP53* and *FANCA* (FDR ≤ 1%) (**Supplementary Notes**, **Supplementary Table 12**).

We also implemented the SAVNet approach[37] to identify rare splicing associated variants (SAV) that appear in only a small number of samples. In total, we could identify 1,901 SAVs (see **Data Availability**): 555/827 acceptor/donor disruptions, 155/364 acceptor/donor creations.

To estimate the number of exonization events within the PCAWG cohort, we filtered all cassette exon events to retain only those that do not occur in the annotation, appear in matched normal

samples or in GTEx samples. Out of 67,254 possible novel cassette exons, we characterized 3,941 (6%) in 2,434 distinct genes as exonization events with 47 (in 43 genes) being located within 25nt of a somatic mutation **(Extended Data Figure 17j bottom, Supplementary Table 13 and 14)** and 20/47 were found to be scSAVs by SAVNet. Interestingly, 43/47 exonization events near a somatic mutation would have been missed by exome capturing probes.

Using pairwise alignments of each Alu sequence overlapping an SAV against the Alu consensus as a reference coordinate system, we found several hotspots of newly created splicing donor and acceptor sites, especially at position 279 close to the poly-T stretch **(Supplementary Figure 16)**.

Patterns of gene fusions across cancers

The availability of both RNA and whole-genome data for over a thousand cancer samples creates an unprecedented opportunity to study the genetic basis of gene fusions (**Figure 3a**). We found that the average number of gene fusions per histological type is highly correlated with the average number of SVs (Pearson correlation 0.95), supporting SVs as a major cause of gene fusions **(Supplementary Figure 17a)**. By examining somatic rearrangements and fusions simultaneously, we found 2,618 fusion events (~82%) that could be explained by genomic rearrangements, with duplication as the predominant type. By contrast, of 19,144 SVs that joined two distinct genes, only 6.3% (1,199) could be detected on the RNA level. This may be the result of the fusion partners not being expressed or having a reduced RNA stability as a mechanism of altering tumor suppressors. Gene fusion discovery directly on RNA-seq data is clearly a more direct approach.

Although most fusions involved genes engaged with only one fusion partner, 35 genes had more than five partners. These "promiscuous" genes tended to be selective in being either a 5' or 3' partner, and were overrepresented in cancer census genes and in PCAWG's cancer driver genes (one tailed Fisher's exact test, odds ratio (OR)=8.66, $P \leq 1.1e-15$ and (OR)=12.27, $P \leq 2.2e-16$, respectively). Network analysis of promiscuous genes and their partners revealed that most genes belonged to small clusters but several larger clusters emerged. Focusing on clusters with at least 10 genes **(Extended Figure 18b)**, we found that they were significantly enriched in cancer-related pathways (Benjamini-Hochberg corrected $P \leq 0.01$) and in protein-protein interactions ($P \leq 1.0e-7$). For example, the known oncogene *BCL6* was involved in 15 different fusions, mostly as a 3' partner with the breakpoints conserved. All such fusions contained the intact exon 2 of *BCL6* and seemed to co-opt the regulatory sequences of their 5' fusion partners. This pattern had been reported previously in B-cell lymphoma[38]. In general, the breakpoints and their positions (3' or 5') were often conserved in promiscuous genes and did not show association with other genomic features such as common fragile sites[39] **(Supplementary Figure 17b)**, indicating that these genes tend to selectively fuse to other genes. Taken together the data suggests that at least some of the promiscuous fusion partners might play a functional role in cancer progression.

The comparison of gene fusions on RNA level and genome rearrangements also allowed us to introduce a classification of gene fusions based on the type of genome rearrangements that may lead to them (**Extended Data Figure 19a**) as well as to introduce the new concept of *bridged fusions* (**Figure 3b**). A large number of fusions, including known fusions, for instance ETV6-

NTRK3, could not be associated with any single SV event. For instance, we found three separate SVs in the same sample: i) a translocation of *ETV6* (chr12:12,099,706) to chromosome 6 (chr6:125,106,892); ii) a translocation of *NTRK3* (chr15:88,694,049) also to chromosome 6 (chr6:125,062,387); and iii) an additional copy number loss (chr12:12,032,501 - chr12:12,099,705) spanning from *ETV6* intron 5 to the exact SV breakpoints (chr12:12,099,706), jointly bringing *ETV6* within 45 kb upstream of *NTRK3*, a distance that would allow transcriptional read-through[40] or splicing[41] to yield the *ETV6-NTRK3* fusion[42]**.** The median length of bridges in bridged fusions was 3.7 kb.

Aside from the bridged fusions, 344 additional fusions are linked to more than one SV in the same sample. These multi-SV fusions are collectively termed *composite fusions*. For example, the known ERC1-RET fusion was only supported by an inter-chromosomal translocation and an intra-chromosomal rearrangement, resulting in the connection of ERC1 to the exon 12 of RET (**Extended Data Figure 19b, middle**). While fusion transcripts formed by two adjacent genes are often thought to be derived from transcription-induced chimeras, such chimera formation could be facilitated by composite DNA rearrangements. For one of the tumours with the recurrent NUMB-HEATR4 fusion, we detected two consecutive inversions, bringing the NUMB exon 3 within 381 bp of the HEATR4 exon 2 (**Extended Data Figure 19b, bottom**), down from the natural distance of 14 kb, arguably making fusion formation possible via splicing. Overall, we identified 75 bridged fusions, 284 inter-composite fusions generated by a translocation linking two genes from different chromosomes followed by a second intra-chromosomal rearrangement, and 125 intra-composite fusions generated by multiple intra-chromosomal rearrangements.

While most fusions had direct or composite SV support, for the remaining 18%, including known fusions like RHOH-BCL6, we did not detect SV evidence. Thus, either these genes were fused directly at the RNA level or the underlying supporting SVs escaped detection. The latter was evidenced by the observation that known fusions, such as TMPRSS2-ERG, did not have consistent SV support in all samples where it was detected (in 4 out of 6 samples this fusion was supported by a deletion, while in the other two samples it did not have any SV support). On the other hand, the 340 SV independent, intra-chromosomal fusions had significantly closer breakpoints than those with SV support (**Extended Data Figure 19d**).

As already noted, 105 fusion transcripts involved the UTR region of one gene and the complete coding sequences of another, possibly resulting from SVs in promoter regions. For example, in a novel fusion *CTBP2-CTNNB1* in a gastric tumor, its intact oncogene *CTNNB1* coding region is connected to the upstream regulatory sequences from *CTBP2*. Accordingly, the expression of *CTNNB1* is elevated at a level similar to those with *CTNNB1* amplification, consistent with a promoter-swapping activation mechanism of oncogenes **(Supplementary Figure 17c)**.

Pan-cancer unified analysis reveal diverse modes of RNA-level alterations

To make use of our comprehensive set of RNA alterations, we sought to characterize the heterogeneous mechanisms of cancer genome and transcriptome alterations. To enable joint analyses of RNA and DNA alterations, we created a binary gene-level table, indicating the

presence or absence of possibly functional events for each gene and patient. Alterations at the nucleotide, amino acid, exon, transcript, or gene level were all mapped onto the most likely affected gene, and were filtered to exclude types of events that were unlikely to cause functional changes, such as synonymous substitutions or short in-frame insertions or deletions. In particular, we only retained non-synonymous SNVs and RNA editing events as well as splicing events that either induce a frameshift or the alternative region contains a variant in the Human Genome Mutation Database[43] of the category "damaging". For quantitative alteration types (expression, splicing, alternative promoters, allele specific expression), the most extreme samples with outlying values within histotype were selected (**Supplementary Figure 18a**), to account for tissue-specific variability. The exact number of alterations for each of the alteration types does depend on filter parameters and those were chosen to have a low observed alteration frequency across the samples. The identification of an event as an outlier alone is not sufficient evidence of it being functionally relevant. The resulting binary gene-level table of RNA alterations enables meta-analyses of aberrations across patients, genes, pathways and in specific histotypes (**Supplementary Figure 18b-c**). We found no significant correlation between the sample purity, gene length, or GC-content with the frequency of outliers (**Supplementary Figure 19, Supplementary Figure 20).**

To check the quality of the gene-level tables, we tested whether each of the alteration types exhibits cancer specificity. We performed gene set enrichment analysis for top genes ranked by their recurrence within each alteration type against the union of COSMIC cancer census genes[44] and driver genes identified in the PCAWG cohort[11]. We found that each of the six RNA- and two DNA-alteration types had a significant enrichment for cancer census genes as well as PCAWG driver genes (FDR ≤ 5%, hypergeometric test).

The gene alteration frequencies across all histotypes showed that while the overall numbers of gene fusions are dwarfed by other types of alterations across cancer types, breast & ovarian adenocarcinomas and soft tissue-leiomyosarcoma are more profoundly impacted by gene fusions (Wilcoxon Rank Sum Test, $P < 1.2 \cdot 10^{-6}$).

In examining DNA- and RNA-level alterations in sets of genes in pathways with known roles in cancer (**Extended Data Figure 21**), we found that even for pathways typically associated with high non-synonymous alterations, such as the TP53 pathway, there are also a sizable proportion of RNA alterations. Among the 739 samples altered in the TP53 pathway, 238 (32.2%) of them carried only RNA alterations, indicating that neglecting transcriptomes would underestimate the degree of cancer pathway alterations. While *TP53* is altered primarily through non-synonymous SNVs, other genes in the TP53 pathway such as *MDM2* is more frequently altered via RNA alterations than DNA alterations (**Extended Data Figure 22b**)

Co-occurrence of RNA and DNA alterations

When we investigate trans-associations between different genetic and expression characteristics, known genetic associations, such as the co-occurring mutations of KRAS and

PIK3CA[45], and those between LATS2 and NF2[46], were recapitulated in this study (**Supplementary Figure 21a**). While some of the co-occurrences could be confounded by the cancer subtype characteristics and their association with certain mutations, notable co-occurrences were present in multiple cancer types. For example, *B2M* and *EIF4G2* alterations were simultaneously observed in both Lymph-BNHL and Lung-SCC. We also observed that tumors with B2M alterations tend to have more non-synonymous mutations (Wilcoxon Rank Sum Test, P = 0.0028) (**Supplementary Figure 21b**), albeit a weaker association, in other cancer types, like Kidney-RCC (Wilcoxon Rank Sum Test, P = 0.072) and breast adenocarcinoma (Wilcoxon Rank Sum Test, P = 0.087). This is consistent with the notion that *B2M* alterations may be associated with altered DNA repair or higher mutation tolerance.

MYC was the COSMIC gene whose variants showed the most frequent co-occurrence with alternative splicing events, such as FLNB alternative splicing in Lymph-BNHL (**Supplementary Figure 21c**). Their simultaneous presence was also observed in a breast tumor and a Head-SCC tumor. This is consistent with the reported role of MYC in regulating the core pre-mRNA splicing machinery in lymphomagenesis[47]. Since an alternative splicing switch in FLNB has been reported to promote the mesenchymal cell state in human breast cancer[48], its role in lymphoma should also be investigated.

When we examined how cancer genes could be impacted by others through detected co-occurrence, we focused on genes involved in splicing and found HNRNPL, previously reported to alter the splicing of a set of RNAs in human prostate tumors and thus drive cancer growth[49], to be linked to 9 alternative splicing events of cancer genes (FDR ≤ 5%) (**Supplementary Figure 21d**). Due to sample size of the PCAWG cohort and extent of heterogeneity between and within cancer types, we did not pursue a trans-analysis with germline variants.

Known and novel candidate driver genes are recurrently altered at the RNA-level

Known driver genes were found to have diverse RNA-level alterations with an associated cis-acting mutation (**Extended Data Figure 24**); therefore, we looked for novel candidate driver genes that had the same feature. A gene with a somatic eQTL, a splicing associated variant, and a fusion event was *PTGFRN*, a gene currently not in the COSMIC cancer gene census (**Extended Data Figure 24c**). Interestingly, both the fusion event and splicing event preserve the frame of the resulting gene products. Further investigation is necessary to understand the functional impact of these RNA alterations.

We aimed to identify genes that are both recurrently and heterogeneously altered, under the hypothesis that these genes have increased functional relevance, and would be driver genes. As a control, a comparative analysis on recurrent expression outliers was also done using GTEx samples, but was not found to be enriched for the union of cancer census genes[44] and PCAWG-defined driver genes (FDR < 10%) (**Supplementary Figure 22, Supplementary Table 22**). *TP53*

has nearly the highest proportion of DNA alterations (75.3% DNA alterations, 414/550) and *GAS7* has a high proportion of RNA alterations (96.5% RNA alterations, 299/310) (**Figure 4d**). Furthermore, when we specifically look at the two most frequent alterations for each gene, a majority (73.53%) of the alterations are at the RNA-level (**Extended Data Figure 25b**). While the total number of RNA alterations does depend on the selected filter parameters, the RNA alterations account for a significant portion of changes in all tested cases.

While our analysis identified 731 genes with significant recurrent aberrations (FDR < 5%, **Extended Data Figure 25a,** permutation-based significance estimation), and is enriched for the union of cancer census genes[44] (60/603) and PCAWG-defined driver genes (33/157, unioned: 72/674 $P=4.6 \cdot 10^{-13}$, enrichment 2.45, **Figure 4e**). Our recurrence analysis of heterogeneous RNA alterations also identifies 659 genes that are neither known cancer census nor driver genes. This includes IRF5[50–53], ZFAT[54], BCAS3[55,56], TLK2[57–59], and COL6A3[60,61], providing new hypotheses for follow-up studies. Those genes may have received less attention because they harbor only rare DNA alterations. The results of our study can also help to understand which parts of the transcripts are altered and functionally affected.

Supplementary code availability

Analysis	Software/Code	Reference	Source
RNA-Seq alignment (STAR)	STAR version 2.4.0i, 2-pass; icgc_rnaseq_align	[1]; this study	https://github.com/ICGC-TCGA-PanCancer/pcawg3-rnaseq-align-star
RNA-Seq alignment (TopHat2)	TopHat2 version 2.0.12; irap for PCAWG	[2,62]	https://hub.docker.com/r/nunofonseca/irap_pcawg/
Gene expression quantification	HTSeq v0.6.1p1	[3]	https://github.com/ICGC-TCGA-PanCancer/pcawg3-rnaseq-align-star; https://hub.docker.com/r/nunofonseca/irap_pcawg/
Transcript quantification	Kallisto v0.42.1	[7]	
eQTL	Limix v0.8.0<br>PLINK v1.07<br>Bedtools v2.25.0<br>Vcftools v0.1.14<br>Bcftools v1.2<br>Samtools v0.1.18<br>Tabix v0.2.6	[63–67]	https://github.com/Functional-Genomics/eQTL
Allele-specific expression	GATK ASEReadCounter 3.8	[27,68]	https://github.com/ICGC-TCGA-PanCancer/pcawg3-ase-sigqtl
Mutational signatures associated with gene expression	Limix v0.8.0<br>Lavaan v0.5-23.1097<br>Mediate v4.4.7		https://github.com/ICGC-TCGA-PanCancer/pcawg3-ase-sigqtl
Alternative promoter			https://github.com/ICGC-TCGA-PanCancer/pcawg3-alternative-promoter

RNA splicing events	SplAdder v1.1.0	32	https://github.com/ratschlab/spladder
Splicing causing mutation detection	SAVNet	37	https://github.com/friend1ws/SAVNet
Gene fusions and structural variants	Sv2gf v0.1.0		https://github.com/nunofonseca/sv2gf
Generation of gene-centric table and recurrence analysis.			https://github.com/ICGC-TCGA-PanCancer/pcawg3-transcriptome-integration

References

1. Dobin, A. *et al.* STAR: ultrafast universal RNA-seq aligner. *Bioinformatics* **29**, 15–21 (2013).
2. Kim, D. *et al.* TopHat2: accurate alignment of transcriptomes in the presence of insertions, deletions and gene fusions. *Genome Biol.* **14**, R36 (2013).
3. Anders, S., Pyl, P. T. & Huber, W. HTSeq--a Python framework to work with high-throughput sequencing data. *Bioinformatics* **31**, 166–169 (2015).
4. Bullard, J. H., Purdom, E., Hansen, K. D. & Dudoit, S. Evaluation of statistical methods for normalization and differential expression in mRNA-Seq experiments. *BMC Bioinformatics* **11**, 94 (2010).
5. Dillies, M.-A. *et al.* A comprehensive evaluation of normalization methods for Illumina high-throughput RNA sequencing data analysis. *Brief. Bioinform.* **14**, 671–683 (2013).
6. Li, B. & Dewey, C. N. RSEM: accurate transcript quantification from RNA-Seq data with or without a reference genome. *BMC Bioinformatics* **12**, 323 (2011).
7. Bray, N. L., Pimentel, H., Melsted, P. & Pachter, L. Near-optimal probabilistic RNA-seq quantification. *Nat. Biotechnol.* **34**, 525–527 (2016).
8. Stegle, O., Parts, L., Piipari, M., Winn, J. & Durbin, R. Using probabilistic estimation of expression residuals (PEER) to obtain increased power and interpretability of gene expression analyses. *Nat. Protoc.* **7**, 500–507 (2012).
9. Roadmap Epigenomics Consortium *et al.* Integrative analysis of 111 reference human epigenomes. *Nature* **518**, 317–330 (2015).
10. Zhang, X. A., Lane, W. S., Charrin, S., Rubinstein, E. & Liu, L. EWI2/PGRL Associates with the Metastasis Suppressor KAI1/CD82 and Inhibits the Migration of Prostate Cancer Cells. *Cancer Res.* **63**, 2665–2674 (2003).
11. Rheinbay, E. *et al.* Analyses of non-coding somatic drivers in 2,693 cancer whole genomes.

Nature (2019).

12. Zhang, W. *et al.* A global transcriptional network connecting noncoding mutations to changes in tumor gene expression. *Nat. Genet.* **50**, 613–620 (2018).
13. Pasqualucci, L. *et al.* Hypermutation of multiple proto-oncogenes in B-cell diffuse large-cell lymphomas. *Nature* **412**, 341–346 (2001).
14. Corces, M. R. *et al.* The chromatin accessibility landscape of primary human cancers. *Science* **362**, (2018).
15. Smith, K. S. *et al.* Signatures of accelerated somatic evolution in gene promoters in multiple cancer types. *Nucleic Acids Res.* **43**, 5307–5317 (2015).
16. Fishilevich, S. *et al.* GeneHancer: genome-wide integration of enhancers and target genes in GeneCards. *Database* **2017**, (2017).
17. Fabregat, A. *et al.* The Reactome pathway Knowledgebase. *Nucleic Acids Res.* **44**, D481–7 (2016).
18. Milacic, M. *et al.* Annotating cancer variants and anti-cancer therapeutics in reactome. *Cancers* **4**, 1180–1211 (2012).
19. Woenckhaus, M. *et al.* Smoking and cancer-related gene expression in bronchial epithelium and non-small-cell lung cancers. *J. Pathol.* **210**, 192–204 (2006).
20. Ingram, W. J. *et al.* ABC transporter activity linked to radiation resistance and molecular subtype in pediatric medulloblastoma. *Exp. Hematol. Oncol.* **2**, 26 (2013).
21. Waszak, S. M. *et al.* Germline determinants of the somatic mutation landscape in 2,642 cancer genomes. *bioRxiv* 208330 (2017) doi:10.1101/208330.
22. PCAWG Group 8. PCAWG-8 Marker Paper. *Nature* (2017).
23. Middlebrooks, C. D. *et al.* Association of germline variants in the APOBEC3 region with cancer risk and enrichment with APOBEC-signature mutations in tumors. *Nat. Genet.* **48**, 1330–1338 (2016).
24. Wallace, C. Statistical testing of shared genetic control for potentially related traits. *Genet.*

Epidemiol. **37**, 802–813 (2013).

25. Baron, R. M. & Kenny, D. A. The moderator-mediator variable distinction in social psychological research: conceptual, strategic, and statistical considerations. *J. Pers. Soc. Psychol.* **51**, 1173–1182 (1986).
26. Preacher, K. J. & Hayes, A. F. SPSS and SAS procedures for estimating indirect effects in simple mediation models. *Behav. Res. Methods Instrum. Comput.* **36**, 717–731 (2004).
27. Castel, S. E., Levy-Moonshine, A., Mohammadi, P., Banks, E. & Lappalainen, T. Tools and best practices for data processing in allelic expression analysis. *Genome Biol.* **16**, 195 (2015).
28. Lindeboom, R. G. H., Supek, F. & Lehner, B. The rules and impact of nonsense-mediated mRNA decay in human cancers. *Nat. Genet.* **48**, 1112–1118 (2016).
29. Morison, I. M., Ramsay, J. P. & Spencer, H. G. A census of mammalian imprinting. *Trends Genet.* **21**, 457–465 (2005).
30. Gerstung, M. *et al.* The evolutionary history of 2,658 cancers. *Nature* (2019).
31. Demircioğlu, D. *et al.* A Pan-cancer Transcriptome Analysis Reveals Pervasive Regulation through Alternative Promoters. *Cell* **178**, 1465–1477.e17 (2019).
32. Kahles, A., Ong, C. S., Zhong, Y. & Rätsch, G. SplAdder: identification, quantification and testing of alternative splicing events from RNA-Seq data. *Bioinformatics* **32**, 1840–1847 (2016).
33. Wang, L. *et al.* Transcriptomic Characterization of SF3B1 Mutation Reveals Its Pleiotropic Effects in Chronic Lymphocytic Leukemia. *Cancer Cell* **30**, 750–763 (2016).
34. Seiler, M. *et al.* Somatic Mutational Landscape of Splicing Factor Genes and Their Functional Consequences across 33 Cancer Types. *Cell Rep.* **23**, 282–296.e4 (2018).
35. Yoshida, K. *et al.* Frequent pathway mutations of splicing machinery in myelodysplasia. *Nature* **478**, 64–69 (2011).
36. Wang, L. *et al.* SF3B1 and other novel cancer genes in chronic lymphocytic leukemia. *N.*

Engl. J. Med. **365**, 2497–2506 (2011).

37. Shiraishi, Y. *et al.* A comprehensive characterization of cis-acting splicing-associated variants in human cancer. *Genome Res.* **28**, 1111–1125 (2018).
38. Xu, W. S., Liang, R. H. & Srivastava, G. Identification and characterization of BCL6 translocation partner genes in primary gastric high-grade B-cell lymphoma: heat shock protein 89 alpha is a novel fusion partner gene of BCL6. *Genes Chromosomes Cancer* **27**, 69–75 (2000).
39. Le Tallec, B. *et al.* Common fragile site profiling in epithelial and erythroid cells reveals that most recurrent cancer deletions lie in fragile sites hosting large genes. *Cell Rep.* **4**, 420–428 (2013).
40. Nacu, S. *et al.* Deep RNA sequencing analysis of readthrough gene fusions in human prostate adenocarcinoma and reference samples. *BMC Med. Genomics* **4**, 11 (2011).
41. Jia, Y., Xie, Z. & Li, H. Intergenically Spliced Chimeric RNAs in Cancer. *Trends Cancer Res.* **2**, 475–484 (2016).
42. Greger, L. *et al.* Tandem RNA chimeras contribute to transcriptome diversity in human population and are associated with intronic genetic variants. *PLoS One* **9**, e104567 (2014).
43. Stenson, P. D. *et al.* The Human Gene Mutation Database: towards a comprehensive repository of inherited mutation data for medical research, genetic diagnosis and next-generation sequencing studies. *Hum. Genet.* **136**, 665–677 (2017).
44. Forbes, S. A. *et al.* COSMIC: somatic cancer genetics at high-resolution. *Nucleic Acids Res.* **45**, D777–D783 (2017).
45. Thomas, R. K. *et al.* High-throughput oncogene mutation profiling in human cancer. *Nat. Genet.* **39**, 347–351 (2007).
46. Tranchant, R. *et al.* Co-occurring Mutations of Tumor Suppressor Genes, and , in Malignant Pleural Mesothelioma. *Clin. Cancer Res.* **23**, 3191–3202 (2017).
47. Koh, C. M. *et al.* MYC regulates the core pre-mRNA splicing machinery as an essential

step in lymphomagenesis. *Nature* **523**, 96–100 (2015).

48. Li, J. *et al.* An alternative splicing switch in FLNB promotes the mesenchymal cell state in human breast cancer. *Elife* **7**, (2018).

49. Fei, T. *et al.* Genome-wide CRISPR screen identifies HNRNPL as a prostate cancer dependency regulating RNA splicing. *Proc. Natl. Acad. Sci. U. S. A.* **114**, E5207–E5215 (2017).

50. Garaud, S. & Willard-Gallo, K. IRF5: a rheostat for tumor-infiltrating lymphocyte trafficking in breast cancer? *Immunology and cell biology* vol. 93 425–426 (2015).

51. Hu, G. & Barnes, B. J. IRF-5 is a mediator of the death receptor-induced apoptotic signaling pathway. *J. Biol. Chem.* **284**, 2767–2777 (2009).

52. Hu, G., Mancl, M. E. & Barnes, B. J. Signaling through IFN regulatory factor-5 sensitizes p53-deficient tumors to DNA damage-induced apoptosis and cell death. *Cancer Res.* **65**, 7403–7412 (2005).

53. Yanai, H. *et al.* Role of IFN regulatory factor 5 transcription factor in antiviral immunity and tumor suppression. *Proc. Natl. Acad. Sci. U. S. A.* **104**, 3402–3407 (2007).

54. Tsunoda, T. & Shirasawa, S. Roles of ZFAT in haematopoiesis, angiogenesis and cancer development. *Anticancer Res.* **33**, 2833–2837 (2013).

55. Bärlund, M. *et al.* Cloning of BCAS3 (17q23) and BCAS4 (20q13) genes that undergo amplification, overexpression, and fusion in breast cancer. *Genes Chromosomes Cancer* **35**, 311–317 (2002).

56. O'Malley, B. W. & Kumar, R. Nuclear receptor coregulators in cancer biology. *Cancer Res.* **69**, 8217–8222 (2009).

57. Kim, J.-A. *et al.* Comprehensive functional analysis of the tousled-like kinase 2 frequently amplified in aggressive luminal breast cancers. *Nat. Commun.* **7**, 12991 (2016).

58. Zarow, C. & Victoroff, J. Increased apolipoprotein E mRNA in the hippocampus in Alzheimer disease and in rats after entorhinal cortex lesioning. *Exp. Neurol.* **149**, 79–86

(1998).

59. Mertins, P. *et al.* Proteogenomics connects somatic mutations to signalling in breast cancer. *Nature* **534**, 55–62 (2016).

60. Arafat, H. *et al.* Tumor-specific expression and alternative splicing of the COL6A3 gene in pancreatic cancer. *Surgery* **150**, 306–315 (2011).

61. Xie, X., Liu, X., Zhang, Q. & Yu, J. Overexpression of collagen VI α3 in gastric cancer. *Oncol. Lett.* **7**, 1537–1543 (2014).

62. Fonseca, N. A., Petryszak, R., Marioni, J. & Brazma, A. iRAP - an integrated RNA-seq Analysis Pipeline. (2014) doi:10.1101/005991.

63. Lippert, C., Casale, F. P., Rakitsch, B. & Stegle, O. LIMIX: genetic analysis of multiple traits. *bioRxiv* 003905 (2014) doi:10.1101/003905.

64. Quinlan, A. R. & Hall, I. M. BEDTools: a flexible suite of utilities for comparing genomic features. *Bioinformatics* **26**, 841–842 (2010).

65. Danecek, P. *et al.* The variant call format and VCFtools. *Bioinformatics* **27**, 2156–2158 (2011).

66. Li, H. *et al.* The Sequence Alignment/Map format and SAMtools. *Bioinformatics* **25**, 2078–2079 (2009).

67. Li, H. Tabix: fast retrieval of sequence features from generic TAB-delimited files. *Bioinformatics* **27**, 718–719 (2011).

68. McKenna, A. *et al.* The Genome Analysis Toolkit: a MapReduce framework for analyzing next-generation DNA sequencing data. *Genome Res.* **20**, 1297–1303 (2010).

Supplementary Figure 1

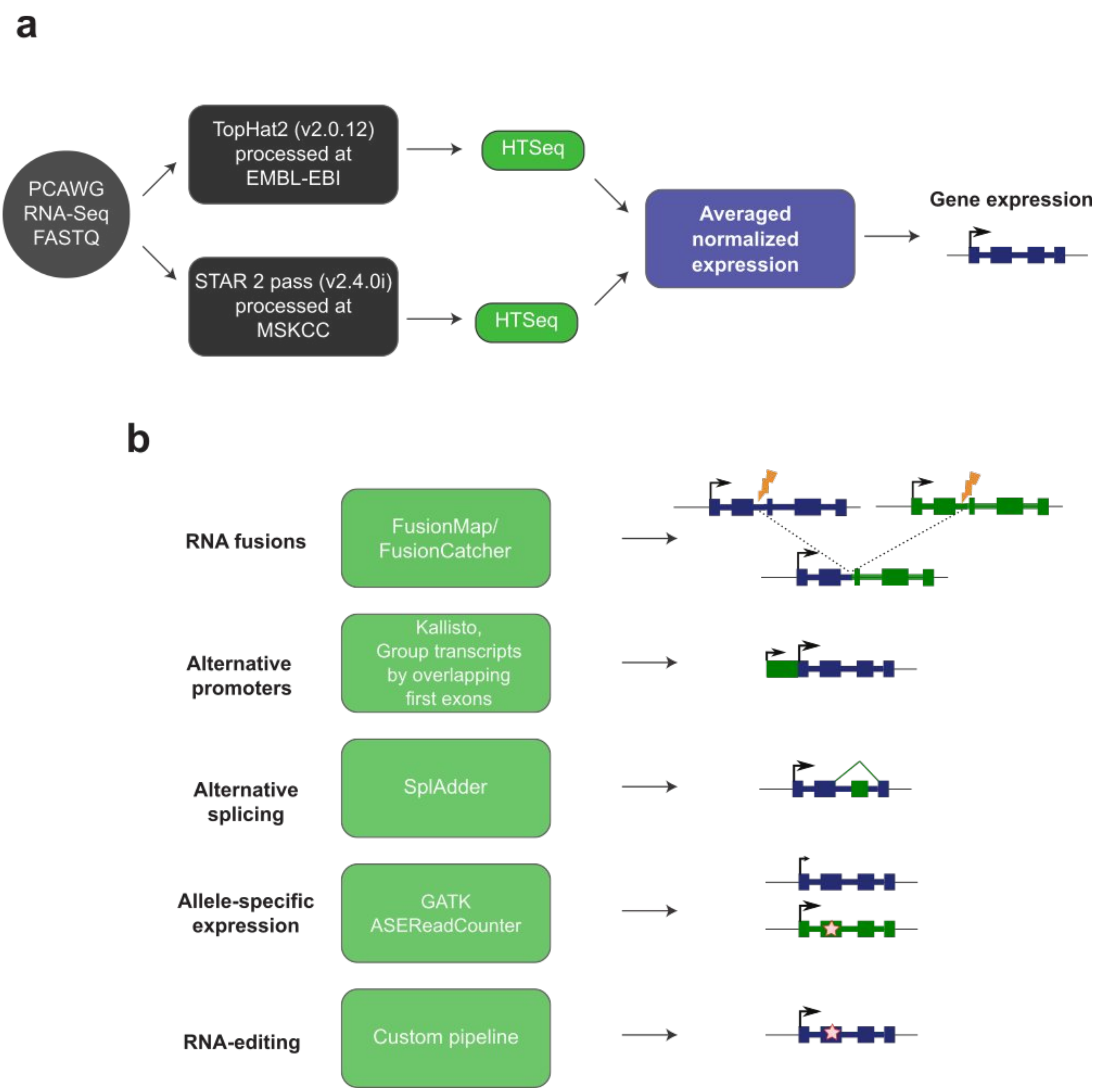

Supplementary Figure 1 | Unified RNA-Seq analysis to identify RNA-level alterations. a, Workflow of RNA-Seq alignment and quantification of gene expression. **b,** Computational methods used to detect additional types of RNA alterations including RNA fusions, alternative promoters, alternative splicing, allele-specific expression, and RNA editing.

Supplementary Figure 2

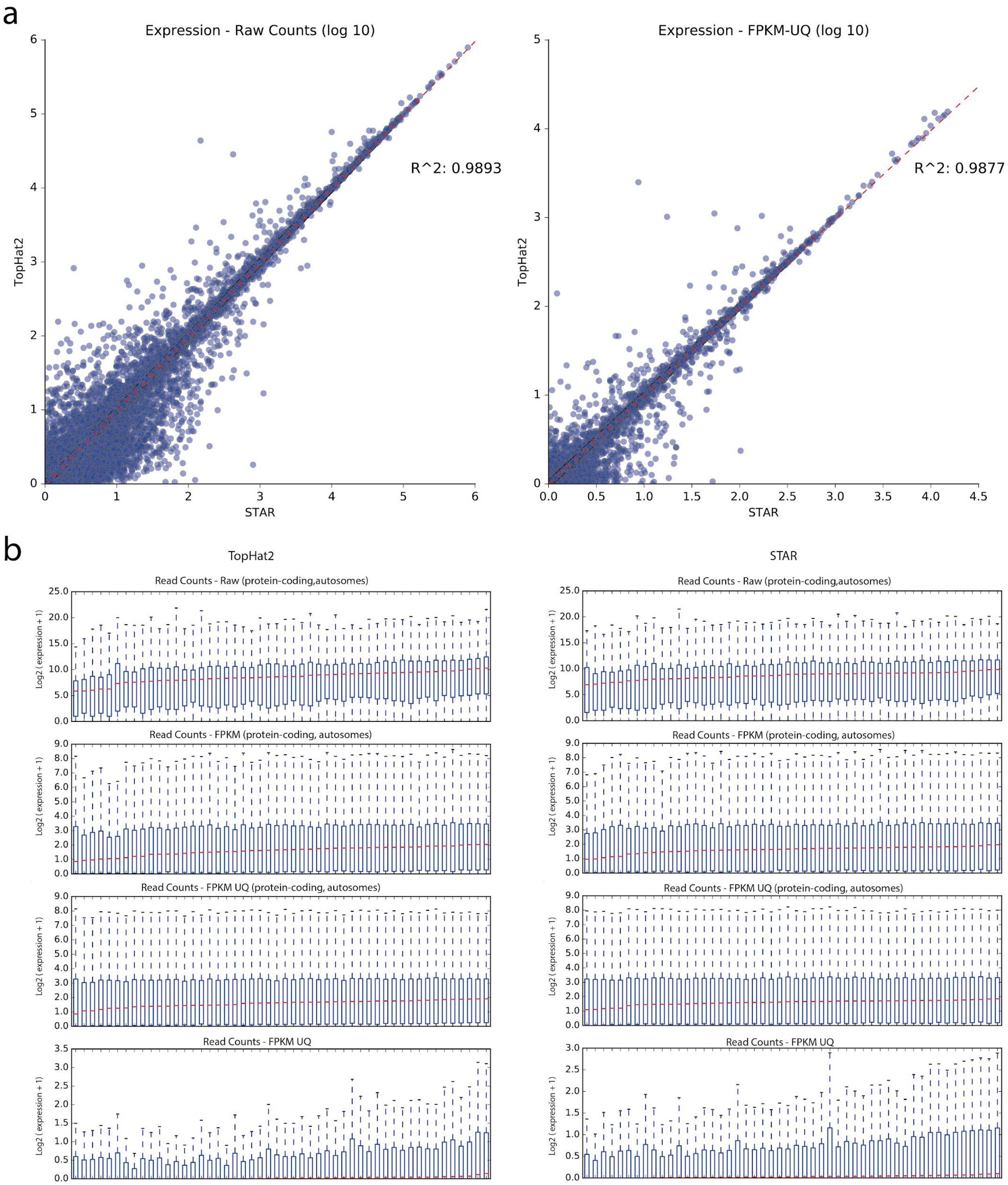

Supplementary Figure 2 | Consensus gene expression quantification and upper-quartile normalization. a, Correlation of STAR vs TopHat2 HTSeq counts for both raw counts (left) and FPKM-UQ normalized counts (right). **b,** Boxplots of raw protein-coding gene quantification on autosomes (top), FPKM on protein-coding genes (second row) and upper-quartile normalized FPKM values on protein coding genes (FPKM-UQ, third row) as well as upper-quartile normalized FPKM on all genes for the same random subset of 50 samples taken from the cohort. Redline is the median.

Supplementary Figure 3

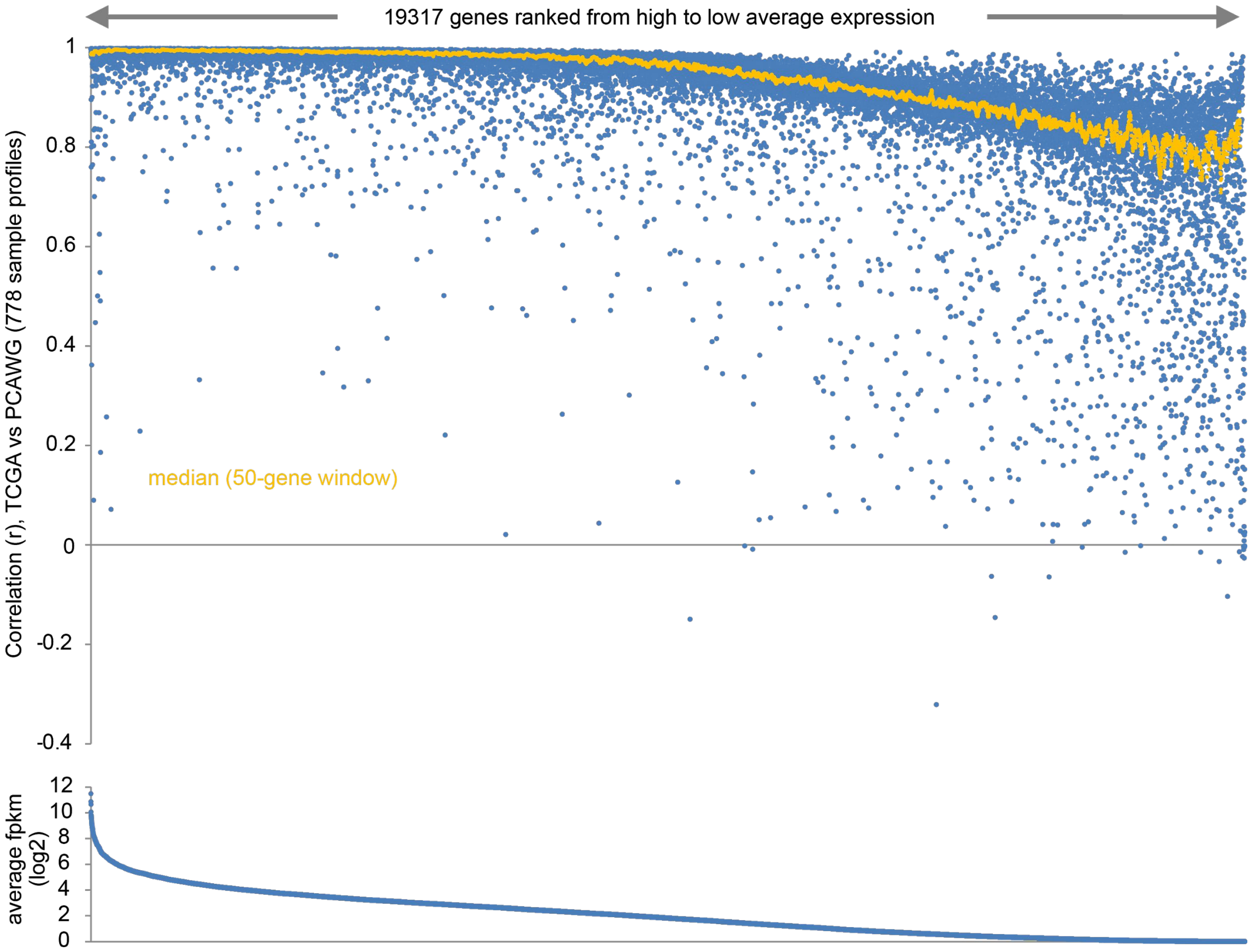

Supplementary Figure 3 | Comparison of TCGA RSEM and FPKM-UQ quantification. For 778 tumor expression profiles represented in both PCAWG and TCGA datasets, gene-level correlations (Pearson's using log-transformed values) between the two datasets were computed (top scatter plot). Genes represented in both datasets are ranked from high to low average log2 fpkm values (PCAWG dataset).

Supplementary Figure 4

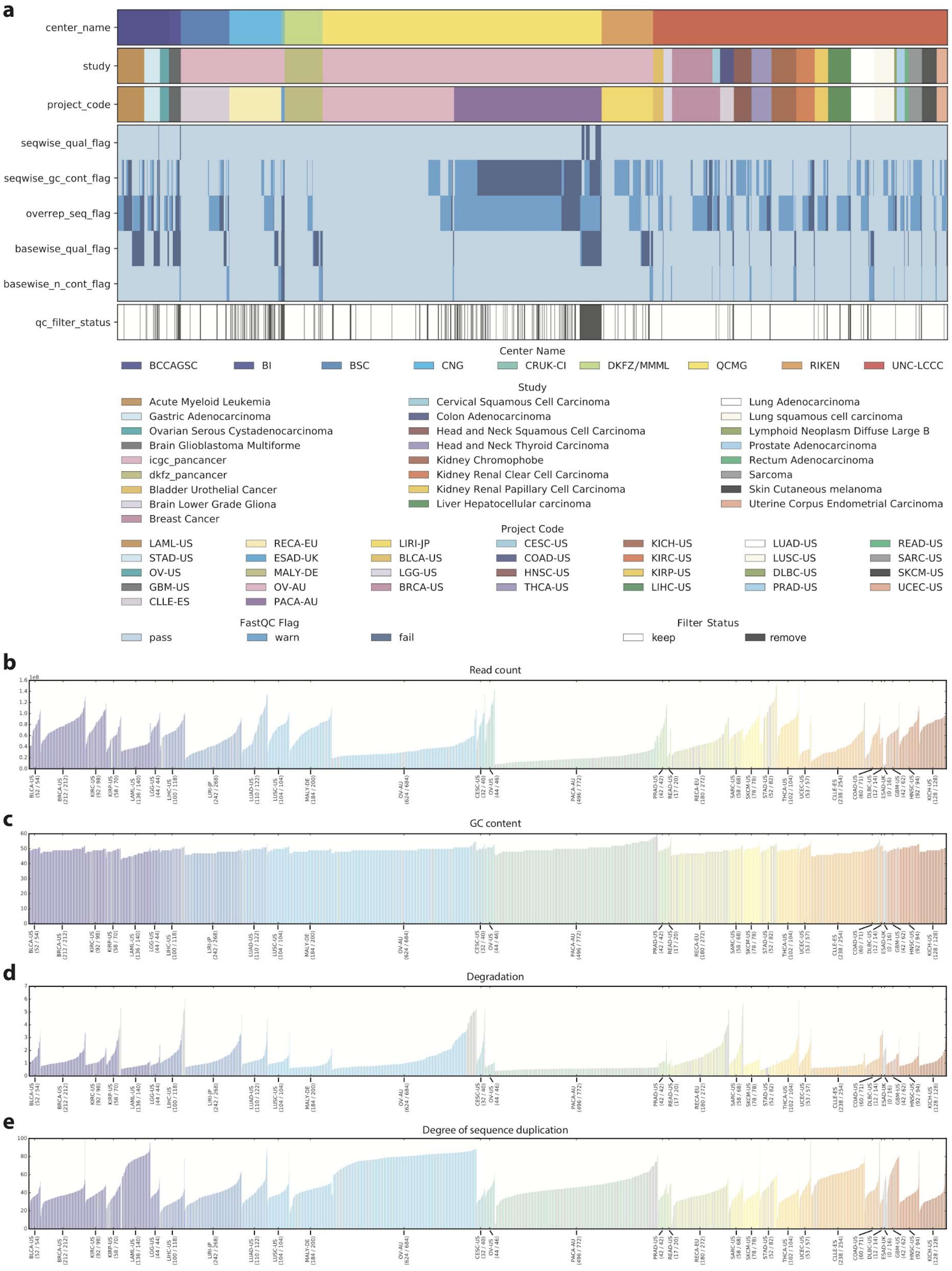

Supplementary Figure 4 | Quality control analysis of RNA-Seq data. **a,** Overview of the subset of QC measures that were used for whitelisting based on output of the FastQC tool. Top bar encodes sequencing center of the library, middle top bar encodes the study metadata as used for tracking, lower top bar labels the project code of a library. The lower block of 5 bars represents presence or absence of FastQC flags. From top to bottom: overall sequence quality, GC content, amount of overrepresented sequences, quality per base, content of N bases. The bottom bar indicates whether a library was filtered based on QC (black) or not (white). **b-e,** Distributions of QC statistics over all libraries shown as histograms. Libraries are colored by project code. Libraries excluded from the whitelist are marked in grey. **b,** Total read count per library. **c.** GC content per library. **d,** Sample degradation scores (3’->5’ bias) per library. **e,** Degree of sequence duplication per library.

Supplementary Figure 5 (Former Fig. 1D)

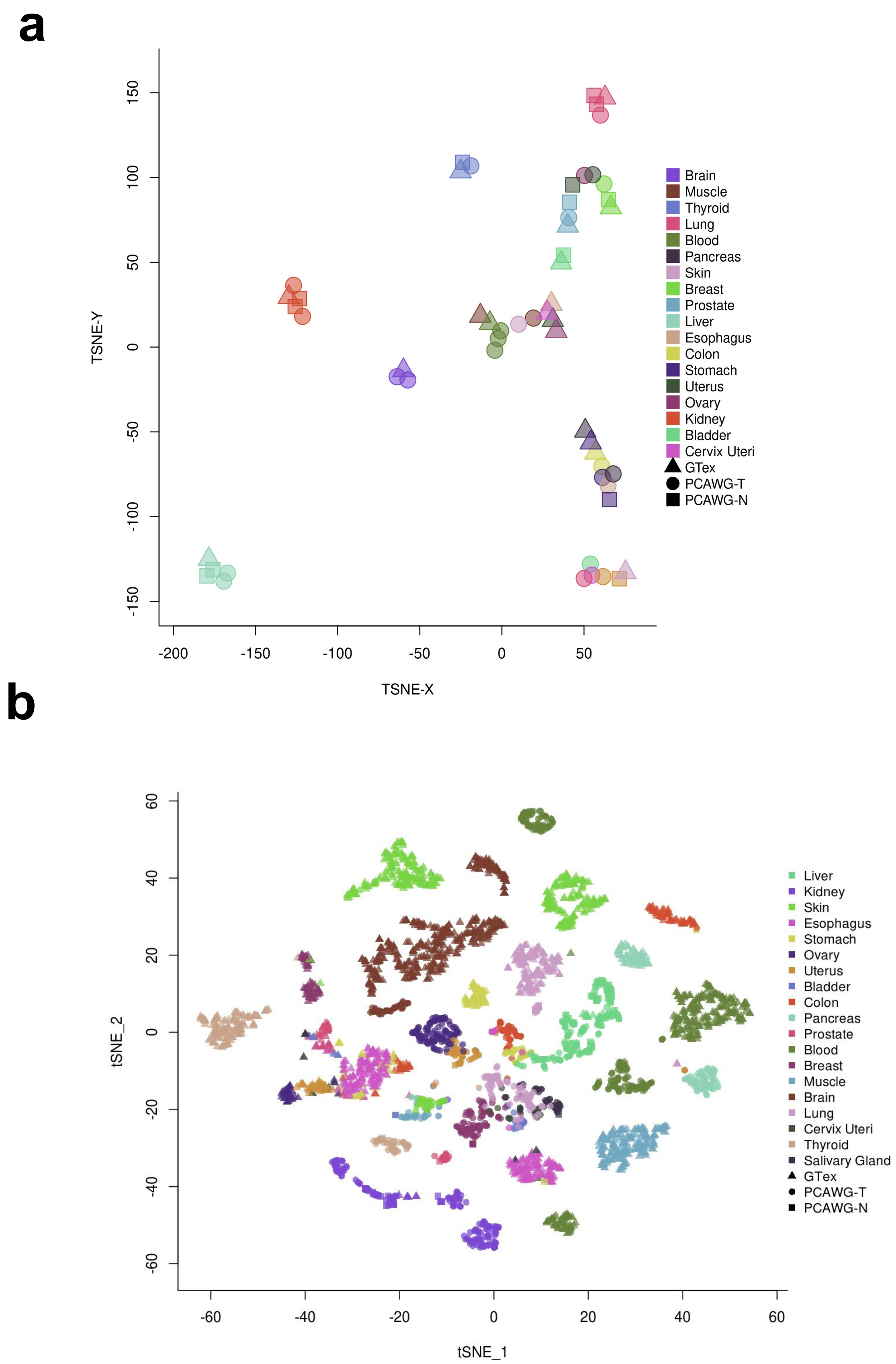

Supplementary Figure 5 | Pan-cancer expression profiling of 1,188 PCAWG donors. a, t-SNE analysis of median gene expression aggregated within each project and each GTEx tissue. **b,** t-SNE plot based on gene expression from samples from GTEx (normal samples) and PCAWG (normal and tumor samples) coloured by tissue.

Supplementary Figure 6

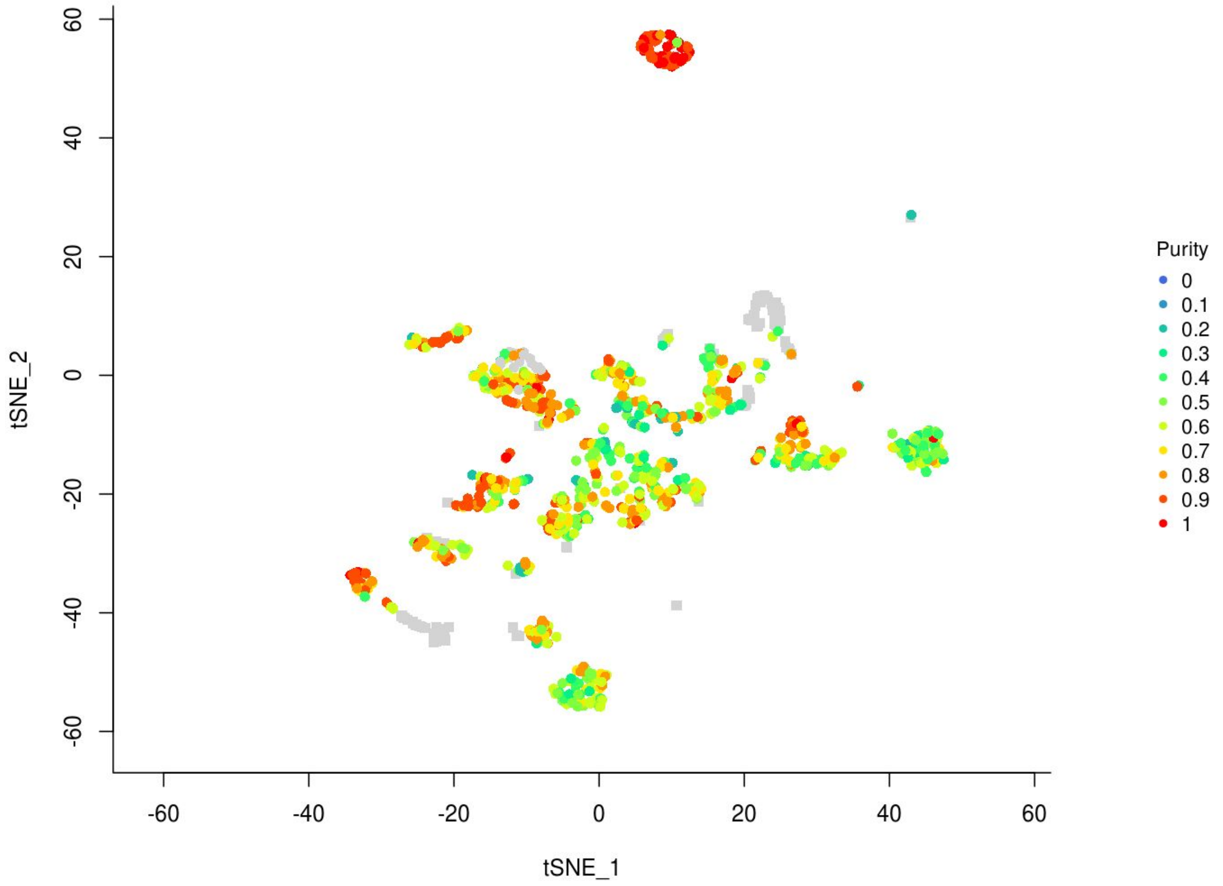

Supplementary Figure 6 | t-SNE analysis of gene expression with estimated tumor purity. t-SNE plot (same as in Supplementary Figure 5b) with the PCAWG samples coloured based on the estimated tumor purity.

Supplementary Figure 7 [EDF8_germline.ai]

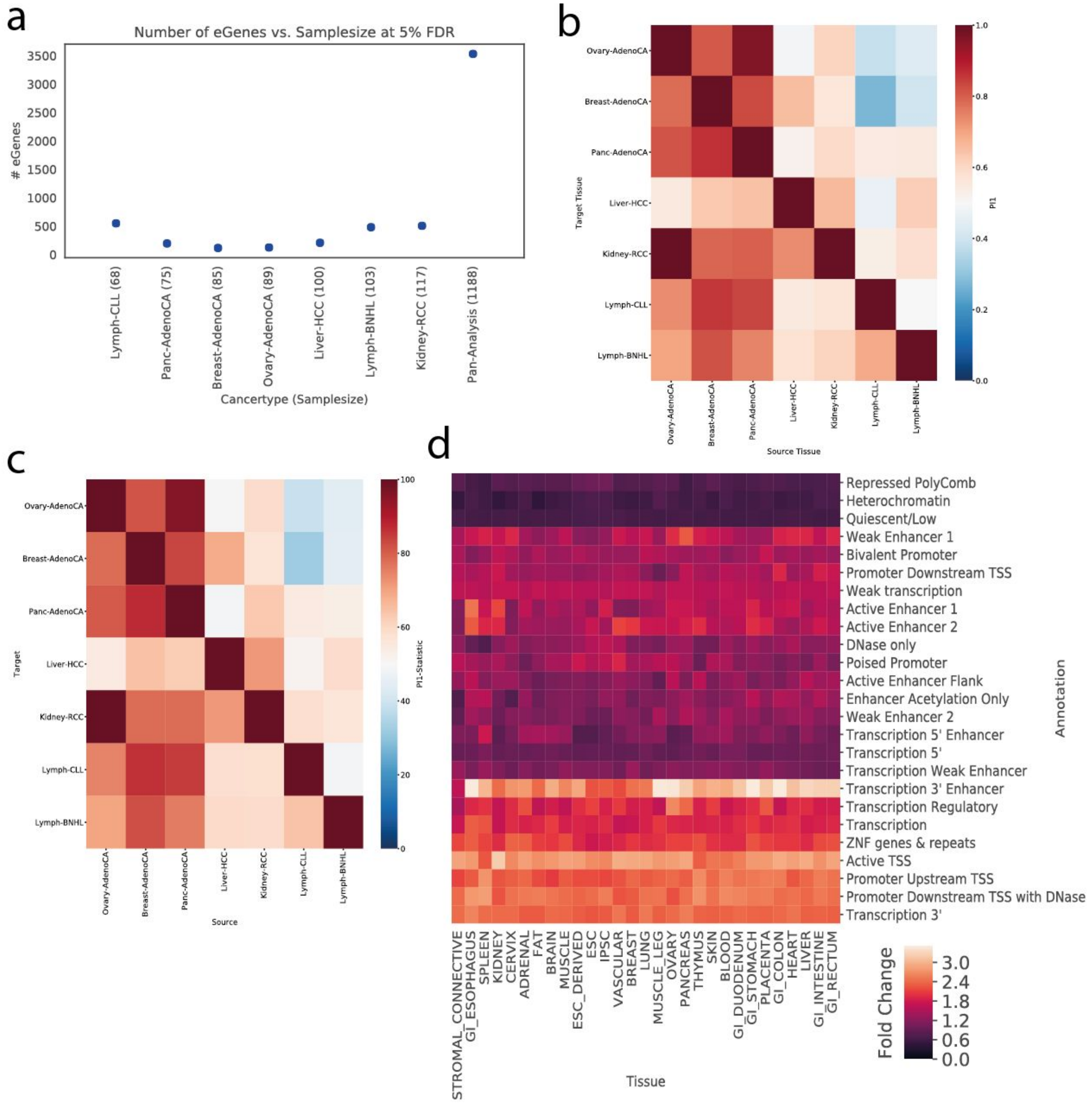

Supplementary Figure 7 | Germline eGenes. a, Number of germline eGenes (genes with at least one germline eQTL, FDR ≤ 5%) per cancer type, sorted by sample size (in parenthesis). **b,** Proportion of eQTL shared (pi1 statistic) between different tissue types. **c**, Proportion of eQTL shared between different histotypes (subsampled to 20 rounds of 100 randomly selected lead variants) **d,** Enrichment of lead variants of Pan-Analysis against a matched background of Roadmap Factors across roadmap cell lines (summarized by tissue identity).

Supplementary Figure 8 [EDF_germline_icgcspecific.ai]

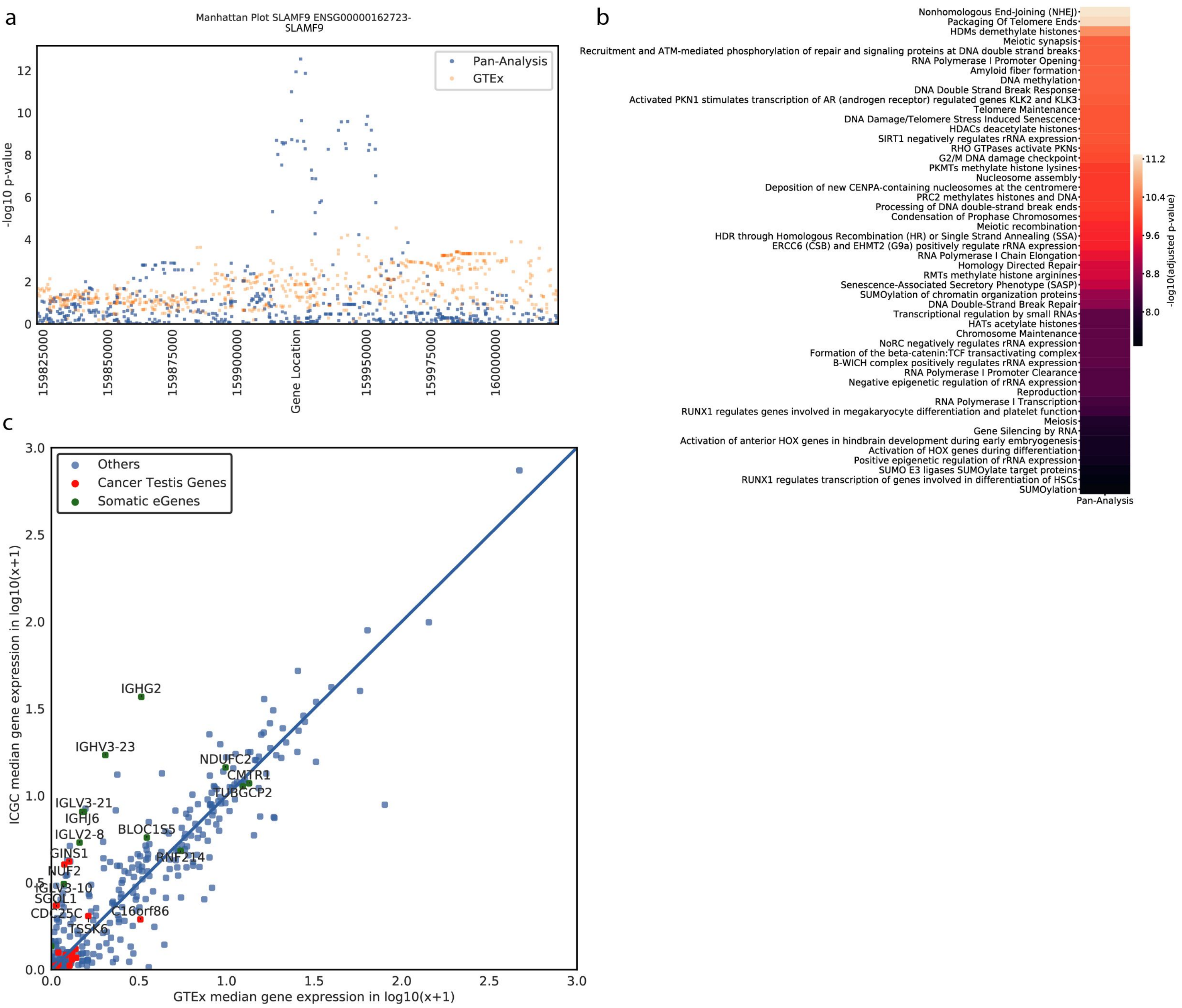

Supplementary Figure 8 | PCAWG specific eGenes. **a,** Manhattan plot of *SLAMF9* as an example of an pan-analysis specific eQTL. as well as *SLX1A* **b,** Top 50 Reactome Pathway enrichments for PCAWG specific eGenes which do not replicate in any GTEx Tissue **c,** Median gene expression across the PCAWG and GTEx cohort for 422 PCAWG-specific eQTL (green: somatic eGenes, red: cancer testis genes)**.**

Supplementary Figure 9

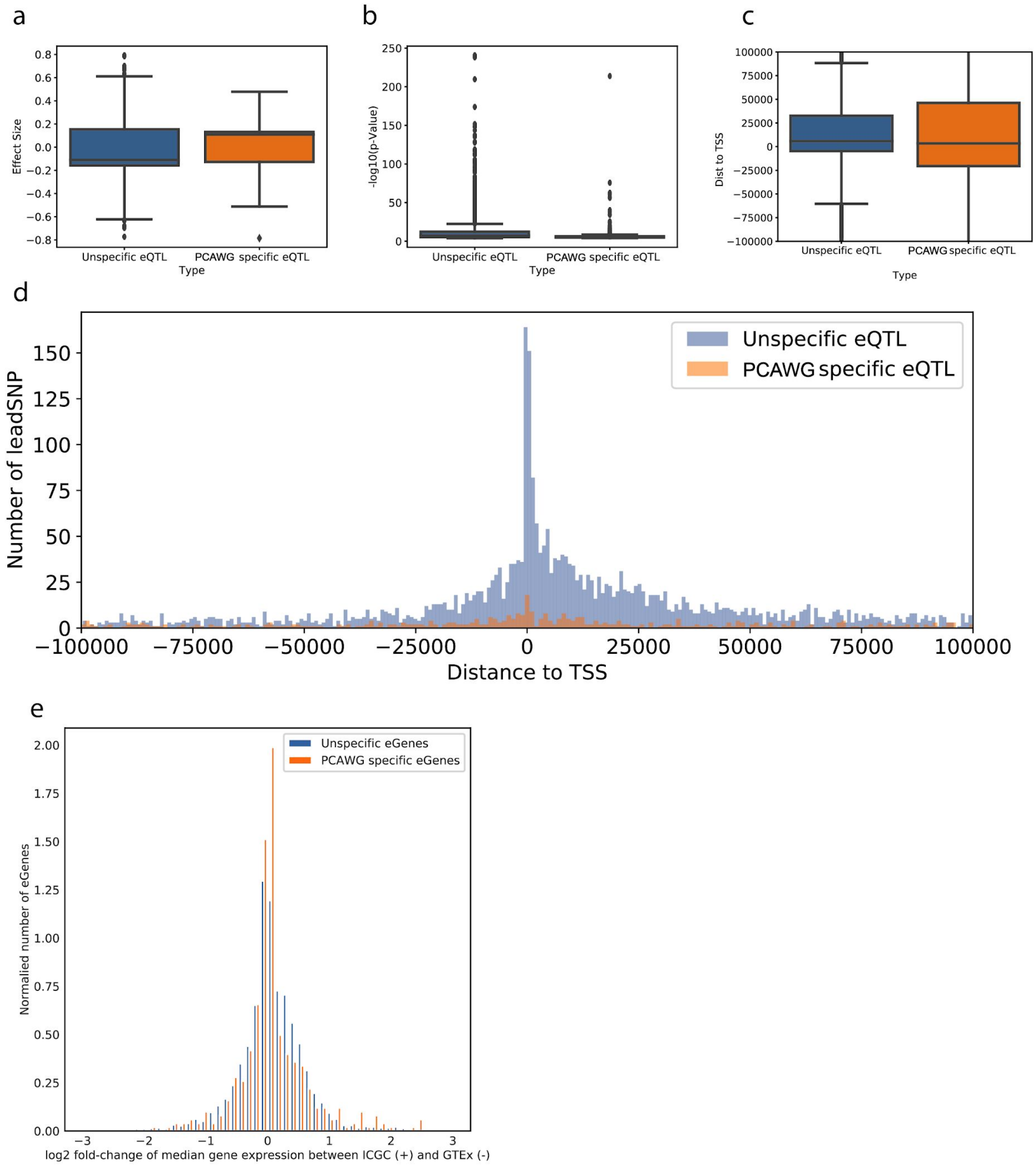

Supplementary Figure 9 | Quality Control of PCAWG specific eGenes. Boxplots of effect size, p-value and distance to TSS. Box indicates interquartile range with median shown as a line. Whiskers indicate 1.5 interquartile range **a,** Boxplot showing effect size distribution of PCAWG-specific and unspecific eQTLs (e.g.: PCAWG eQTL that show evidence of replication in GTEx tissues). **b,** Boxplot showing p-Value distribution of PCAWG-specific and unspecific eQTLs. **c,** Boxplot of distribution of distance to transcription start site (TSS) between specific and unspecific eQTLs. **d,** Distribution of distance to TSS presented as a histogram. **e,** log2 fold change of median gene expression of all ICGC eGenes (orange) and PCAWG specific eGenes (blue) in comparison to GTEx. Positive fold-change indicates higher gene expression in the PCAWG cohort.

Supplementary Figure 10

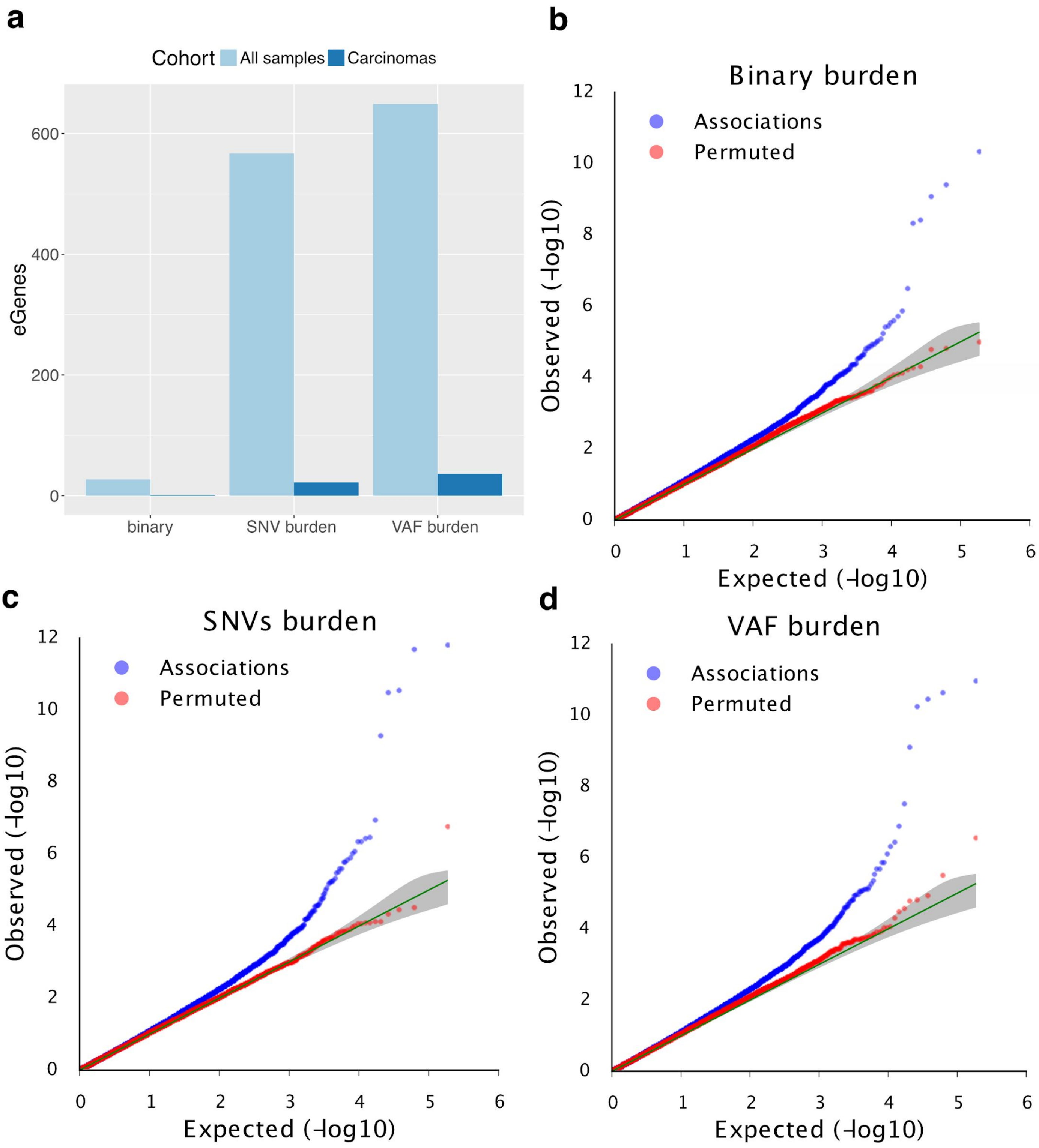

Supplementary Figure 10 | Power of different strategies for estimating somatic mutational burden for eQTL analysis. a, Number of significant somatic eQTL (FDR ≤ 5%) identified with different mutational burden estimates using all 1,188 patients and a subset of 899 carcinomas patients. VAF = variant allele frequency. **b-d**, QQ plots of nominal and permuted p-values (generated after one random permutation of individuals) of the somatic eQTL analysis. Considered were mutational burden calculated as **b**, binary burden (presence or absence of at least one somatic mutation), **c**, total SNVs load (number of somatic mutations per element) or **d**, weighted burden (sum of VAFs over the genomic region tested, **Methods**).

Supplementary Figure 11

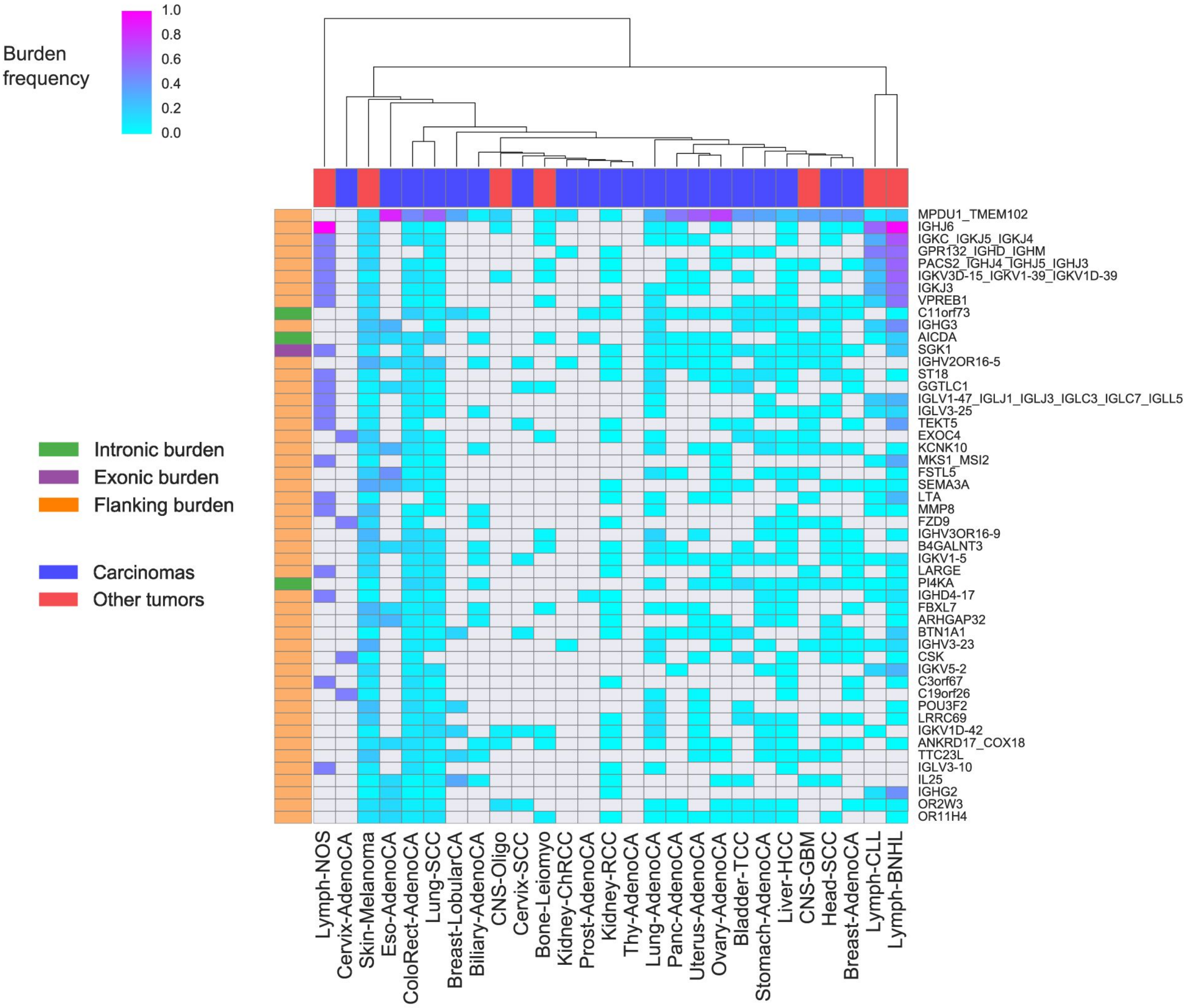

Supplementary Figure 11 | Somatic burden prevalence in the cohort. Clustering of somatic *cis* eQTL (FDR ≤ 5%) by mean burden frequency estimated in each cancer type. The heatmap shows the first top 50 associations, sorted by mean burden frequency of the lead element across all cancer types. Row labels describe the HGCN names of the eGenes associated to leading somatic burden. Multiple eGenes associated to the same genomic interval are joined by an underscore. Row colors indicate the genomic region of the burden (flanking, intronic or exonic). Column colors distinguish the two main tumor types in the cohort, namely carcinomas and other tumors (lymphomas, skin melanoma and glial tumors). Grey cells indicate burden frequency = 0.

Supplementary Figure 12

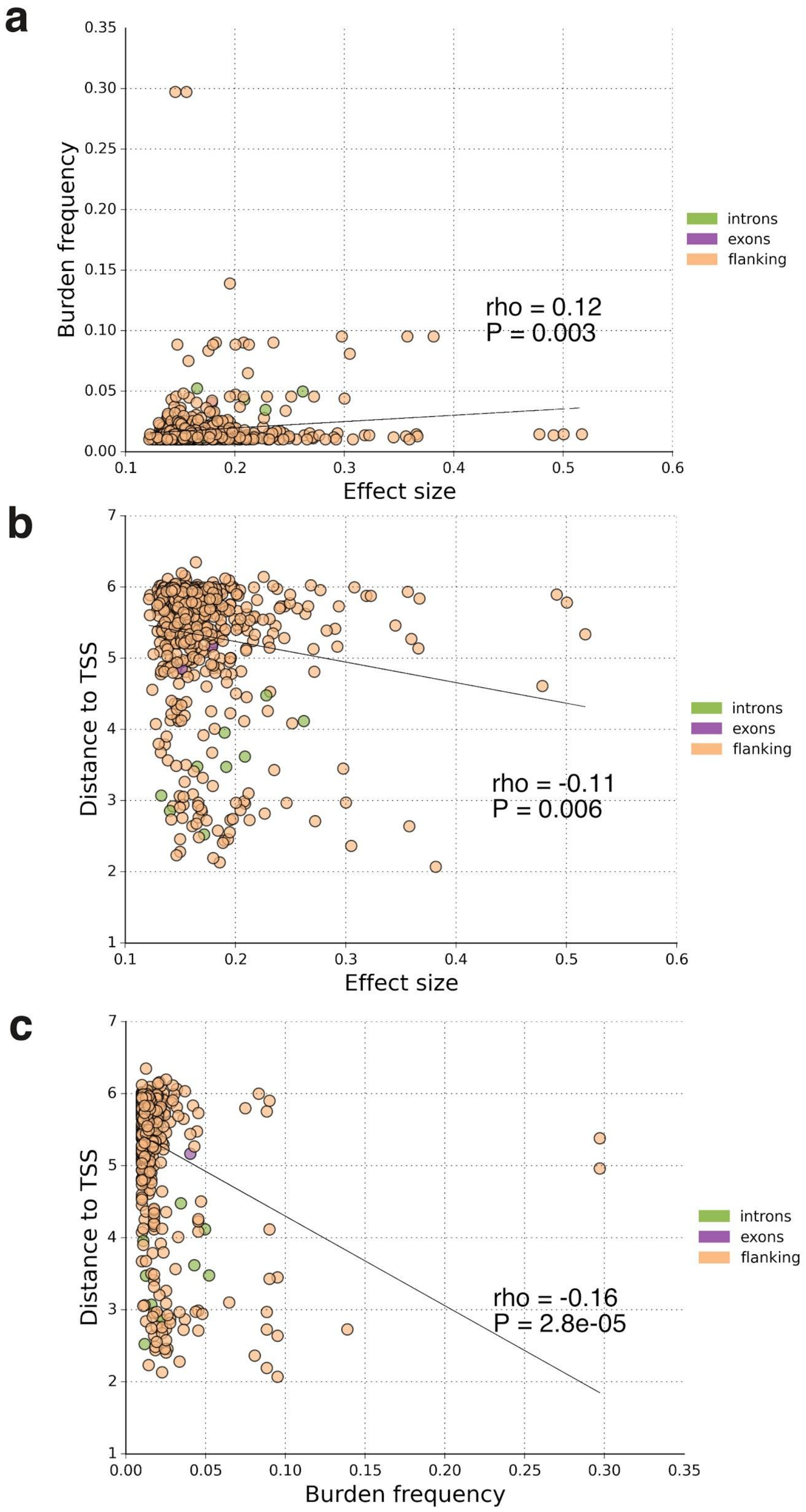

Supplementary Figure 12 | Standardized effect size, distance to TSS and burden frequency of somatic eQTL
a, Lead interval burden frequency and **b**, absolute distance to the eGene TSS shown as function of the absolute value of the effect size for each of the 649 somatic eQTL associations identified (FDR ≤ 5%), stratified by type of interval. **c**, Absolute distance to the eGene TSS shown as function of lead burden frequency. Spearman rho correlation and P value are also shown.

Supplementary Figure 13

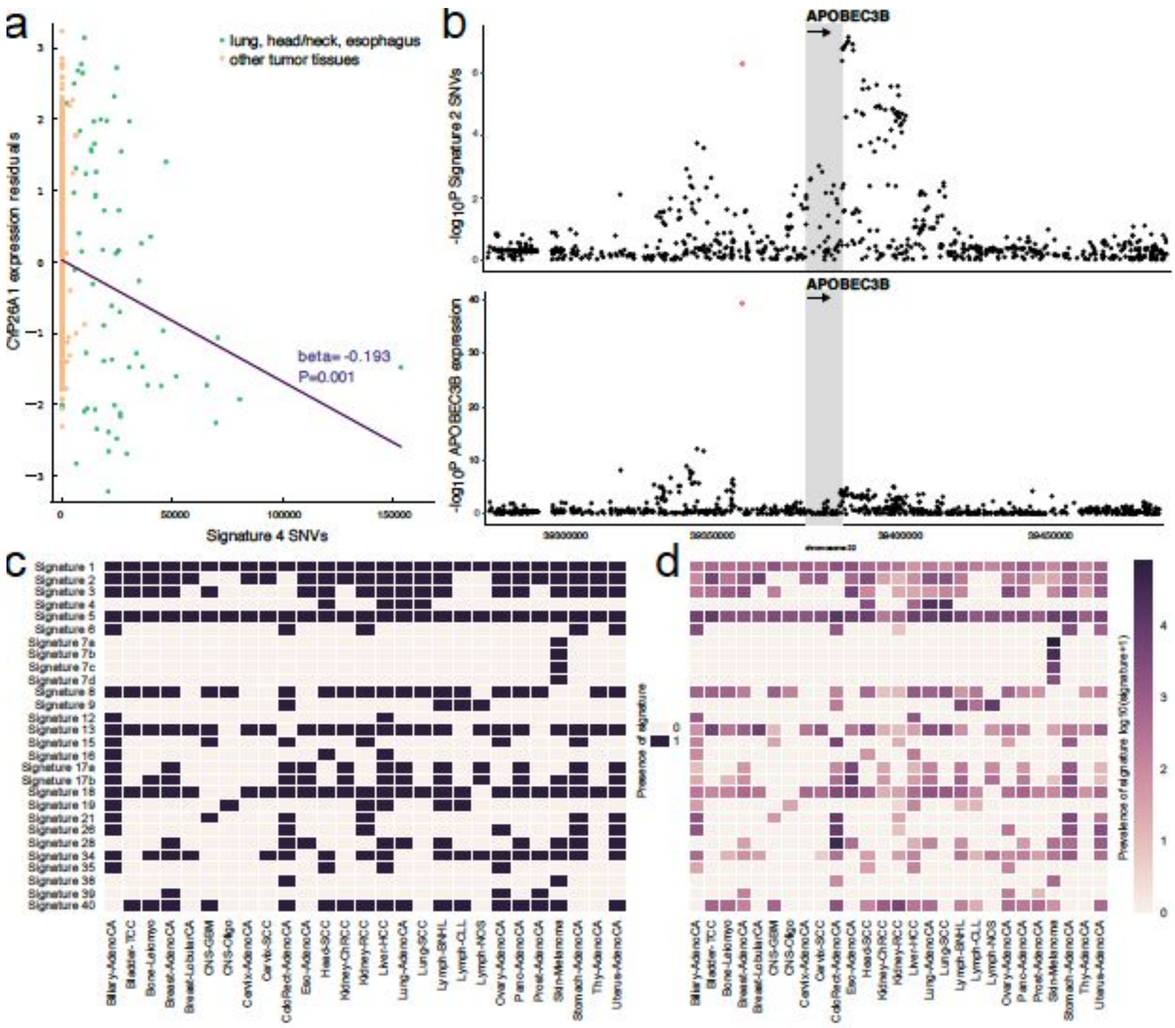

Supplementary Figure 13 | Mutational signature-gene expression-germline variant associations, and prevalence of mutational signatures across cancer types. a, Representative signature-gene association, depicting a negative association between *CYP26A1* expression and Signature 4. **b,** Manhattan plots of associations between *cis* germline variants proximal to *APOBEC3B* (plus or minus 100kb from the gene boundaries) and Signature 2 (top panel) or *APOBEC3B* gene expression level (bottom panel). The gray region denotes the gene body, the orange variant the lead eQTL variant rs12628403. **c,** The heatmap shows the presence of each signature in a specific cancer type (at least one mutation of the respective signature occurs in at least one patient with the specific cancer type). Signatures 1 and 5 occur in all cancer types, signatures 2, 13 and 18 are common signatures and signatures 4, 7, 12, 16, 38 and 39 occur in specific cancer types. **d,** The heatmap shows the prevalence of each signature, i.e. the mean signature count ($\log_{10}$(count + 1)) across all patients of one cancer type.

Supplementary Figure 14

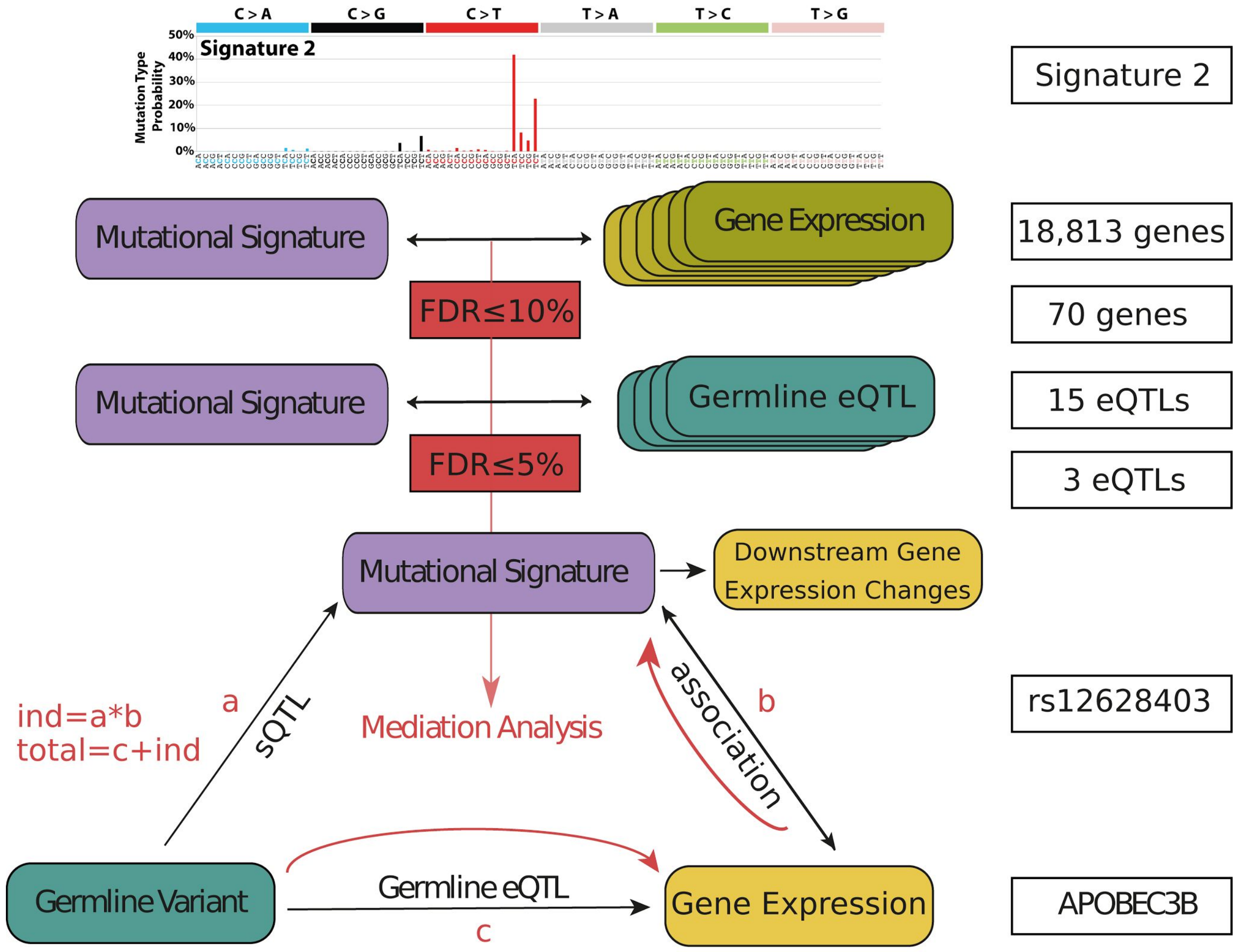

Supplementary Figure 14 | Workflow of gene expression-mutational signature association studies with subsequent mediation analysis including germline eQTL of associated genes. Genes involved in significant mutational signatures and gene expression associations are queried for germline eQTL. If the germline eQTL lead variant is significantly associated with the mutational signature, mediation analysis is applied to each potential triple of germline eQTL lead variant, gene expression and associated mutational signature. Here, the mediating effect of the mutational signature is assessed by comparing the indirect (ind) and total effect of the germline variant onto gene expression (the same analysis has been conducted for gene expression as mediator). a, b and c denote the effect sizes of the individual associations. The boxes on the right show the numbers of genes and eQTL for the *APOBEC3B* case.

Supplementary Figure 15

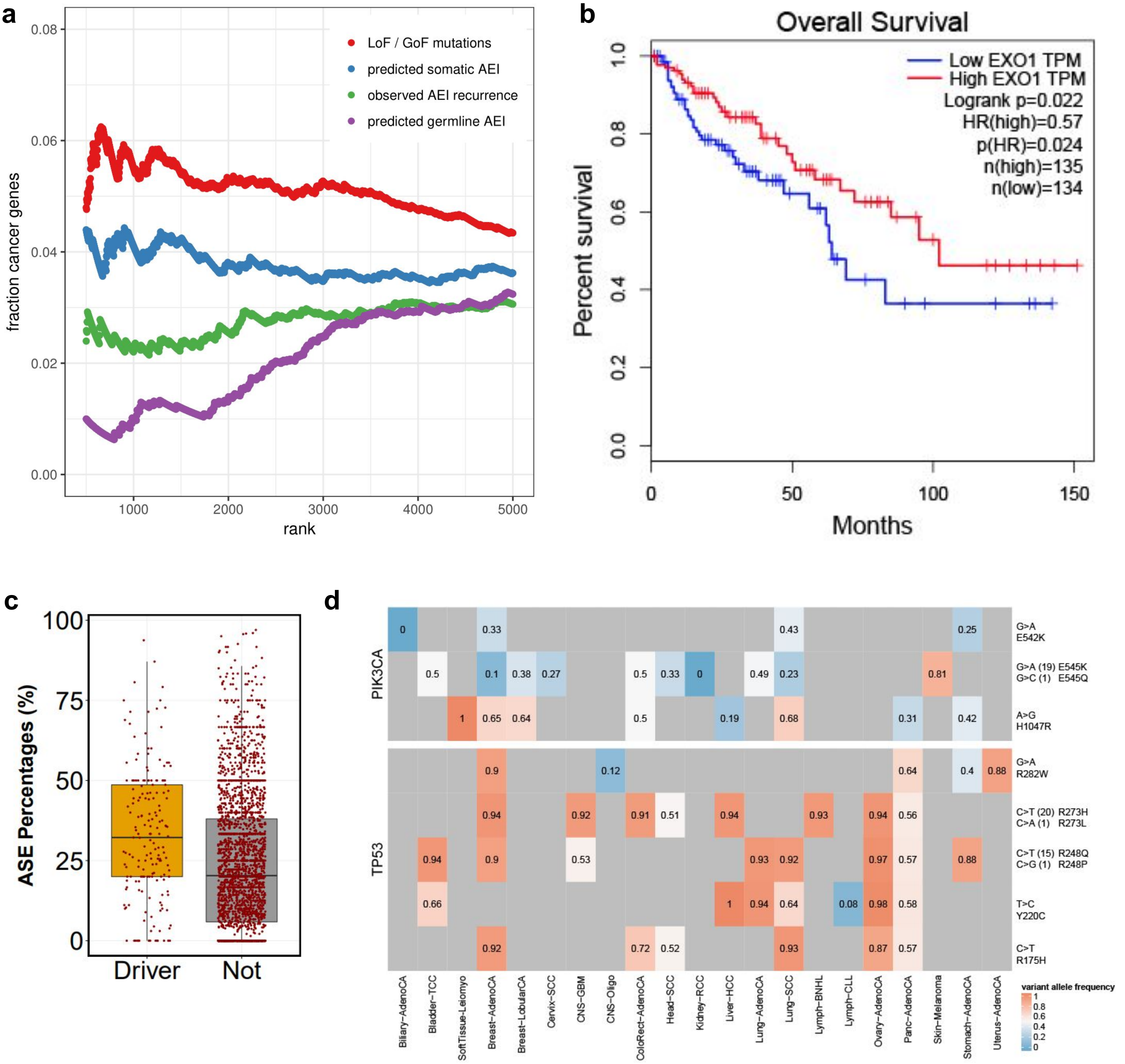

Supplementary Figure 15 | Somatic allelic imbalance predicts cancer-relevant genes: a, Using our model for AEI, we ranked all measurable genes in the cohort according to their average prediction scores from somatic sources (SCNAs + SNVs, blue) and germline sources (SNPs, purple). For comparison, we also ranked genes based on the number of loss-of-function / gain-of-function mutations (red) across the cohort and based on the observed AEI recurrence in the cohort. The plot shows the fraction of cancer census genes (y-axis) in the set of ranked genes up to a specific rank (x-axis). Unsurprisingly, LoF/GoF mutations most clearly indicate cancer genes, followed by predicted somatic AEI. Observed total AEI as well as germline predicted AEI show a clear negative enrichment with only few cancer genes in the top ranked genes. **b,** Kaplan-Meier curves for TCGA COAD patients according to EXO1 gene expression. **c,** Boxplot comparing ASE percentages for driver genes in their functional cancer types and other cancer types. **d,** We used the positions mutated in more than 10 ICGC tumors as hotspots and we looked at the COSMIC genes with more than 2 hotspots. Thus we got the *TP53* and the *PIK3CA* genes. The heatmap showed the variant allele frequency from RNA-seq data for each hotspot of *TP53* or *PIK3CA* in each tumor type.

Supplementary Figure 16

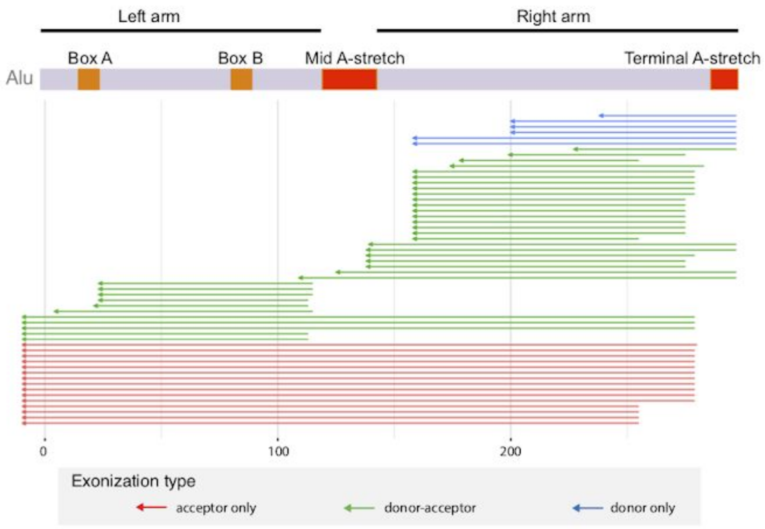

Supplementary Figure 16 | Pairwise alignment of Alu sequences with an SAV and the Alu reference sequence. SAVs aligned to overlapping ALUs aligned to the ALU reference, showing exonizations creating a novel acceptor (red), a novel donor (green) or both (blue).

Supplementary Figure 17

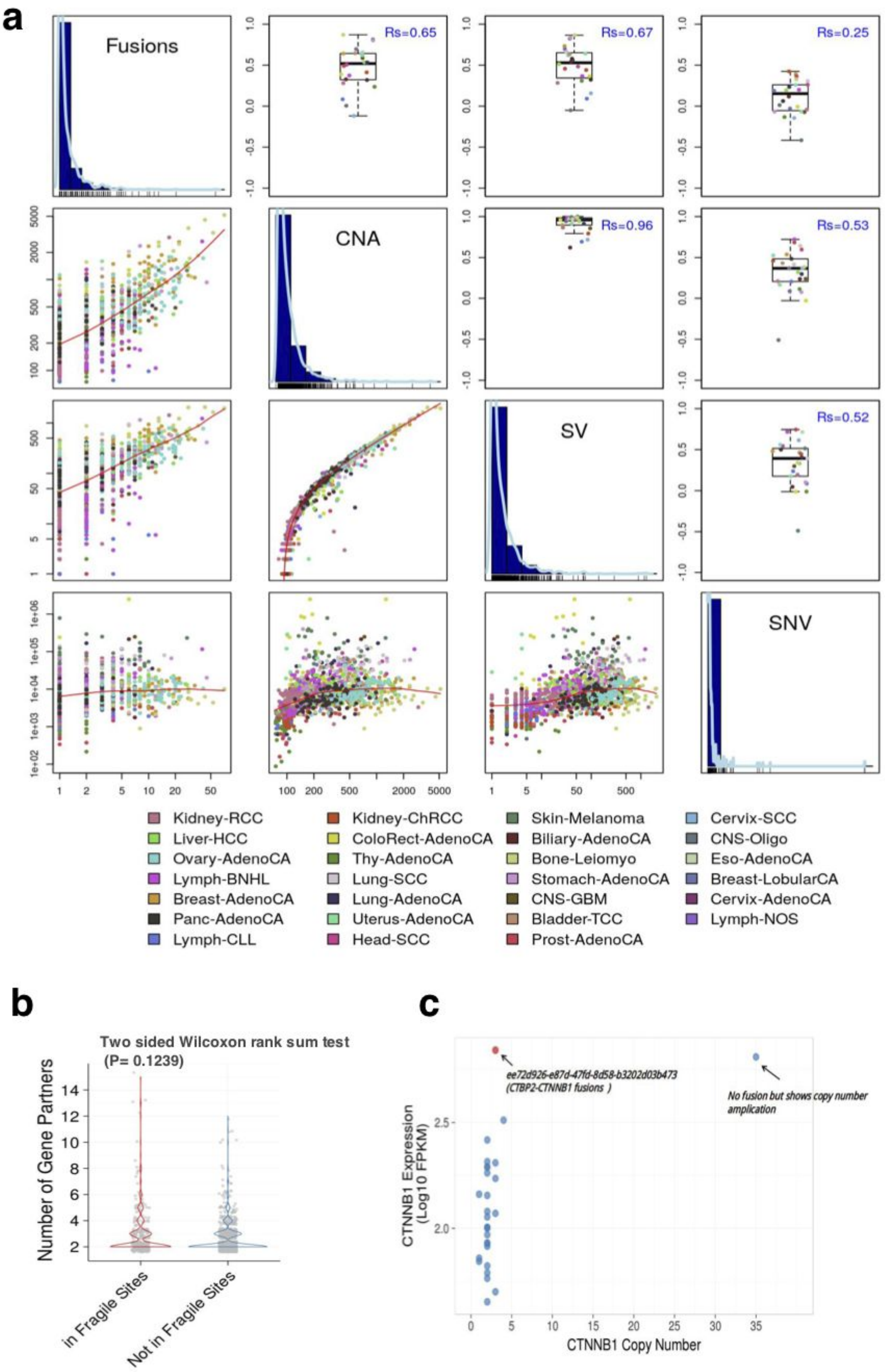

Supplementary Figure 17 | Structural rearrangements associated with RNA fusions. **a,** Number of gene fusions per sample and respective number of fusions, structural variants (SV), copy number alterations (CNA), and single nucleotide variants (SNV). The diagonal histograms shows the distribution of the number of alterations per sample. The upper triangle presents the Spearman correlation between two types of alterations per histological type (dot) and together with the overall spearman correlation (in blue). The bottom triangle contains scatter plots contrasting the number of alterations for each sample (dot). **B,** Fusion genes with promiscuous gene partners overlapped with human common fragile sites do not show different number of gene partners. **c,** *CTBP2-CTNNB1* as an example of "Retained ORF" fusion. A scatter plot of *CTNNB1* DNA copy number versus mRNA expression across all ICGC gastric cancer samples

Supplementary Figure 18

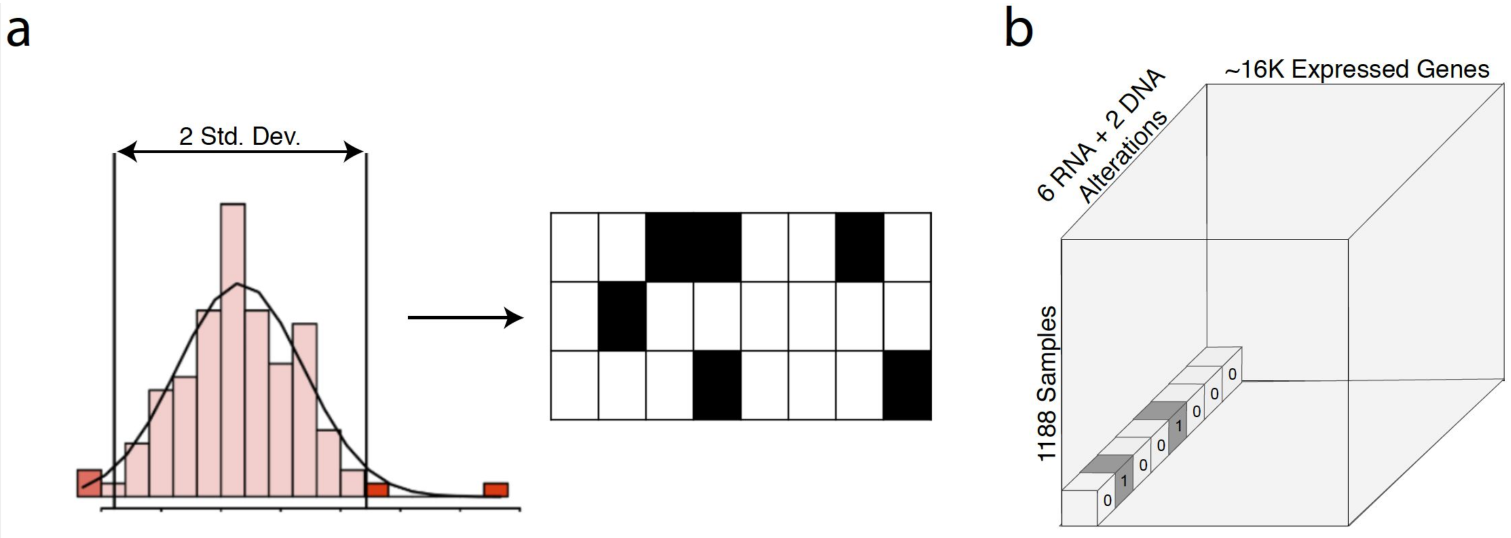

c

Ensembl_Gene_ID	hgnc_symbol	ICGC_DONOR_ID	gene_sample_pairs_curr	Cancer_Type	alt_prom	expr_outlier	rna_edit	variants	ase_all	fusion	isSplice	cn
**ENSG00000141510**	TP53	DO51594	ENSG00000141510_DO51594	Cervix-SCC	0	1	0	0	1	0	0	0
**ENSG00000141510**	TP53	DO52029	ENSG00000141510_DO52029	Head-SCC	0	0	0	0	0	0	1	0
**ENSG00000167244**	IGF2	DO52029	ENSG00000167244_DO52029	Head-SCC	0	0	0	0	0	0	0	0
**ENSG00000171862**	PTEN	DO51594	ENSG00000171862_DO51594	Cervix-SCC	0	0	0	0	0	0	0	1
**ENSG00000254709**	IGLL5	DO51594	ENSG00000254709_DO51594	Cervix-SCC	0	0	0	0	1	0	0	0

Supplementary Figure 18 | Example of the gene-centric outlier table. To unify analysis of alterations across all RNA phenotypes, a gene-centric binary table was created for each RNA phenotype indicating if a sample had an alteration in a given gene for a given sample. For quantitative RNA phenotypes (gene expression, alternative promoters, alternative polyadenylation, alternative splicing, and allele-specific expression), for a given gene, samples with extreme values, when compared to the samples in the same histotype, were considered altered. **a,** depicts the identification of outlying events for a single alteration type. **b,** visual representation of the gene-centric outlier table for the sample D051594 and gene TP53, corresponding to the first row in **c**. We see here that each gene-sample-alteration triple is a binary value, here depicted as a cube. **c,** depicts an example of the gene-centric outlier table for five genes and two samples. The first five columns denote the gene and sample information, the following columns denote if an alteration occurred within the gene-sample-alteration triple. It is important to note that gene-sample pair is included in the table only if it has at least one observed alteration.

Supplementary Figure 19

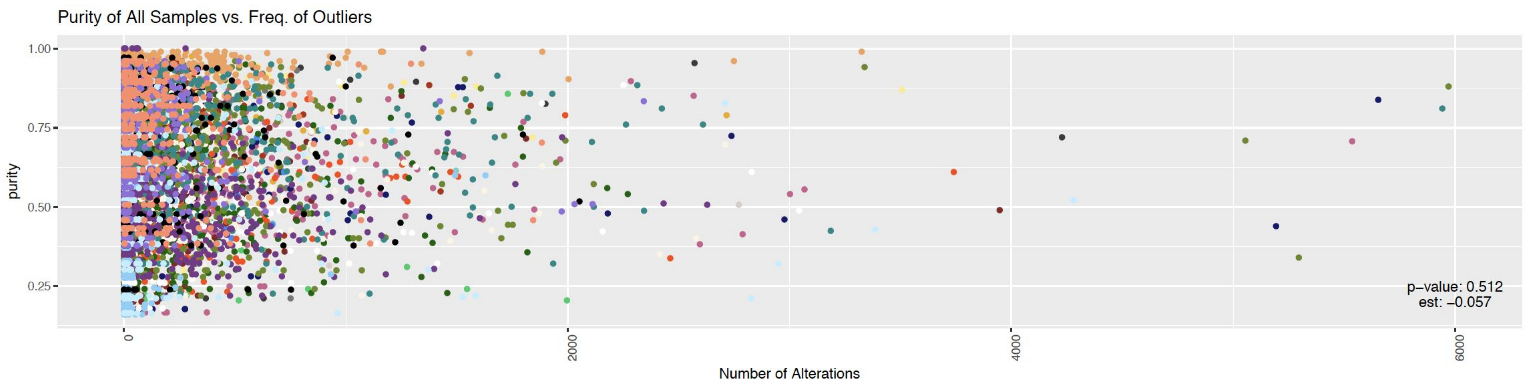

Supplementary Figure 19 | Correlation of Purity and Alteration Frequencies
Each point is a sample-alteration pair and colored by histotype, x-axis depicted the number of genes a specific alteration occurs within a sample. When estimating the relationship between purity and frequency of outliers, using histotype, alteration type, and ploidy as confounding variables, we find that there is no significant correlation (likelihood ratio test, negative binomial distribution: P=0.512).

Supplementary Figure 20

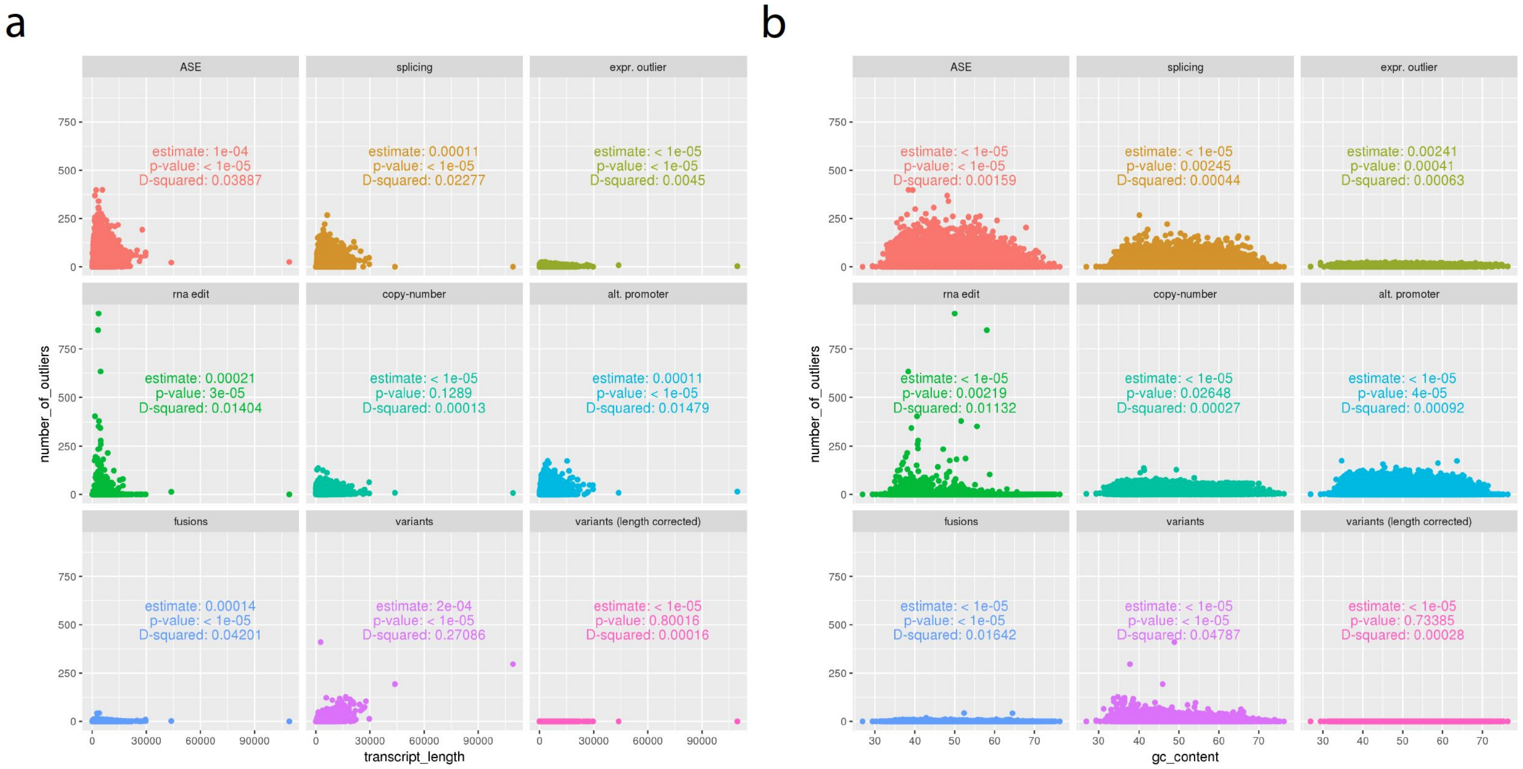

Supplementary Figure 20 | Correlation of frequency of outlier events in a gene with gene length and GC-content. a, comparison of frequency of outliers in a gene with the transcript length. To test strength of correlation, we regress the frequency of outliers against the gene length assuming a negative binomial distribution. We find that while all alteration types except for copy number have a significant correlation, but for only for variants is the amount of variation explained by the gene length greater than 5%. Using this information, we scaled the number of variants detected by the gene length. **b,** compares the frequency of outliers against GC content. We find that after length correction of variants, no alteration has more than 2% of its variance explained by GC content.

Supplementary Figure 21

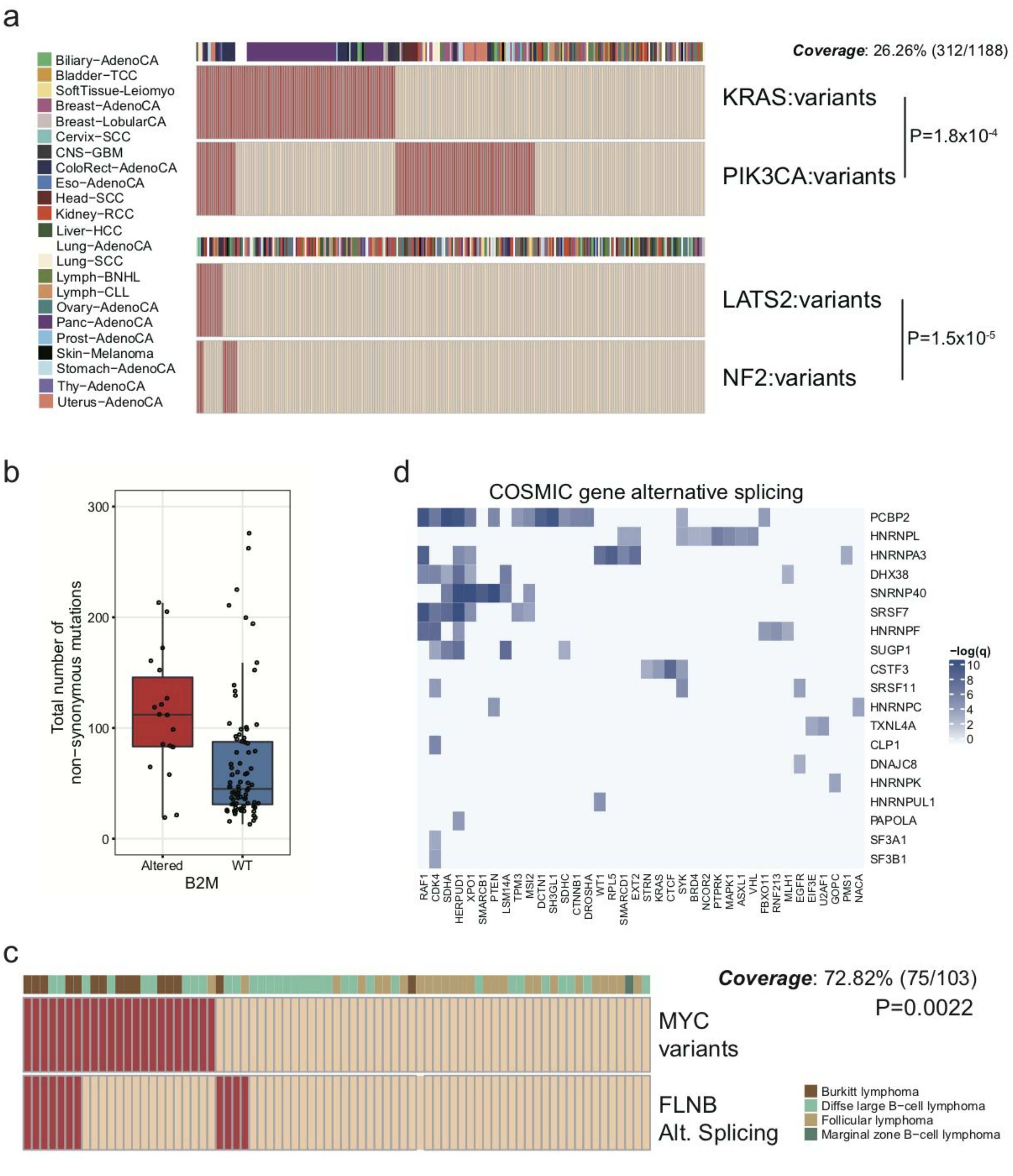

Supplementary Figure 21 | Trans-associations of RNA alterations in cancer genes. a, Heatmap showing the known co-occurrence between mutations of *KRAS* and *PIK3CA*, and those between *LATS2* and *NF2*. Each column indicates a specific tumor with tumor types annotated to the left. Most samples without the listed alterations are not shown for space considerations. **b,** Boxplots showing the total number of non-synonymous mutations acquired for patients with B2M alterations versus those without. **c,** Heatmap showing the co-occurrence between the MYC variants and the FLNB alternative splicing in Lymph-BNHL. Each column indicates a specific tumor. Multiple samples without the listed alterations are not shown for space considerations. **d,** Heatmap showing the extent of associations between alterations of known splicing-related genes and the alternative splicing of COSMIC genes. Each column indicates one COSMIC gene, and the color intensity shows the significance of trans-association. Splicing related genes labeled to the right are ordered by the number of significant associations.

Supplementary Figure 22

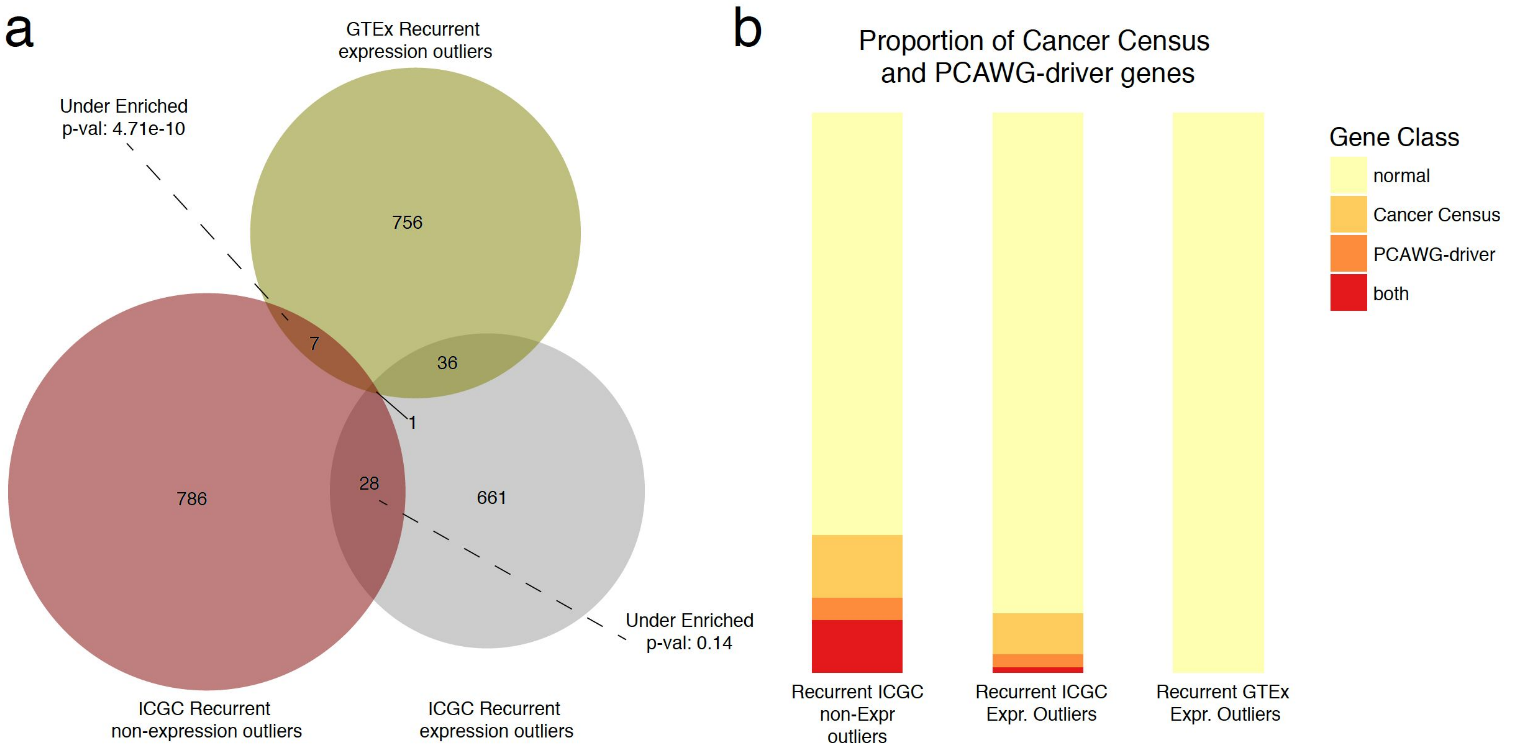

Supplementary Figure 22 | Comparison of recurrent expression outliers in GTEx and ICGC a, represents three sets of significantly recurrent gene sets. The GTEx recurrent expression outliers were derived from the GTEx cohort (version phs000424.v4.p1), totaling 3323 samples across 31 tissues. Expression outliers were identified within each cohort as described in (**Methods**) for the ICGC expression outliers. The recurrent ICGC non-expression outliers contains all alterations except for expression outliers. A permutation test of ~160K, was done to identify a recurrence cut-off score for each of the three gene sets individually. We also find a higher enrichment of ICGC recurrent expression outliers in comparison to the GTEx recurrent expression outliers with other ICGC alteration outliers. **b,** depicts the proportion of cancer census genes and PCAWG-defined driver genes in each of the three recurrent gene sets. We find that the ranked-list of significant ICGC expression and non-expression outliers are significantly enriched (FDR < 5%), but GTEx recurrent expression outliers were not enriched.

Supplementary Figure 23

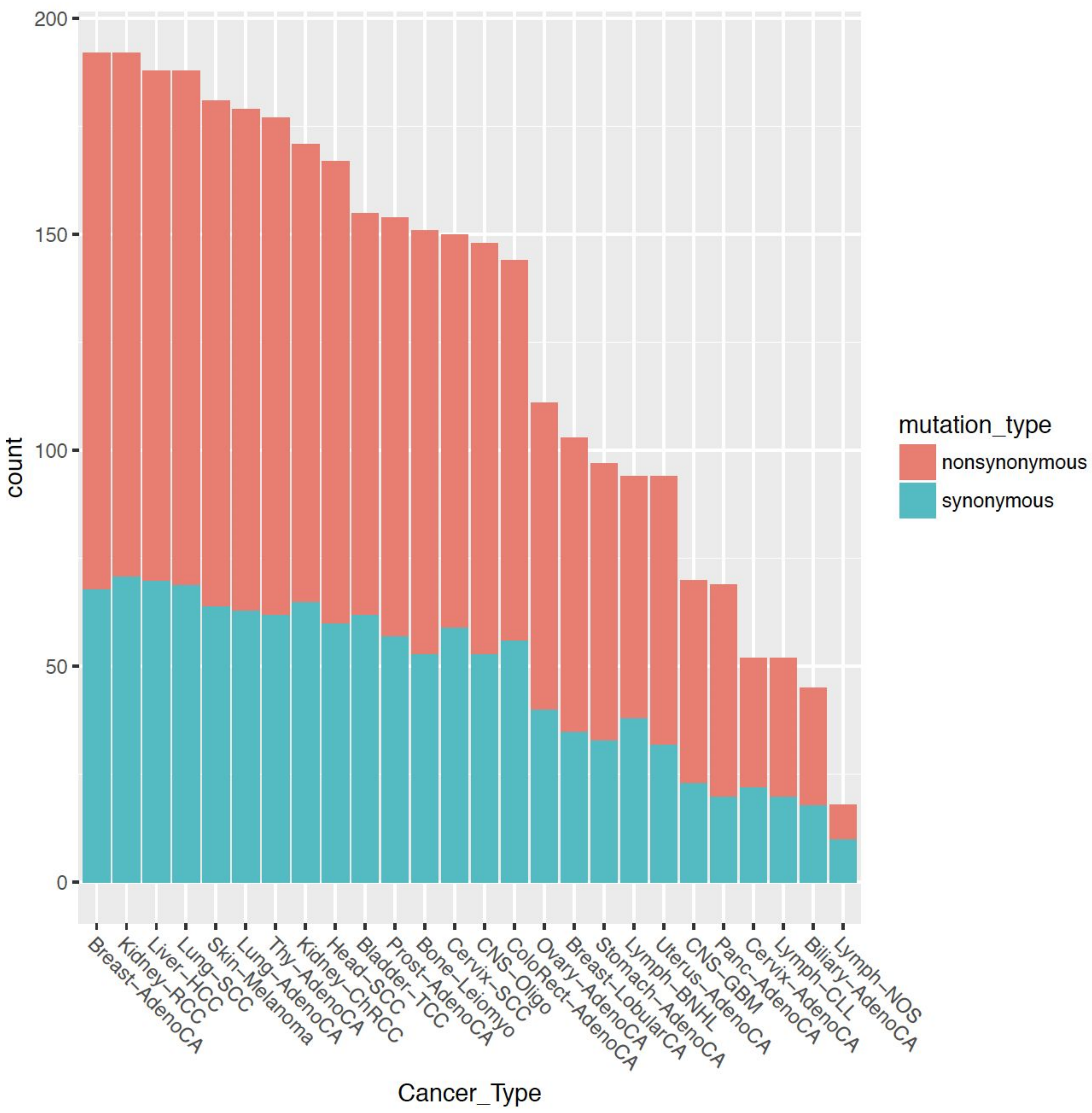

Extended Data Figure 23 | RNA-Editing frequencies within coding regions across tumor types. The bar plot displays the number of rna-editing events that lead to either a synonymous or nonsynonymous mutation across cancer types after application of all filters. An additional filter of seeing an event in at least 30 samples is included in order to make this figure more comparable to the frequencies observed in the TCGA cohort in Han et al. 2018, supplementary figure S1, D.

Supplementary Figure 24

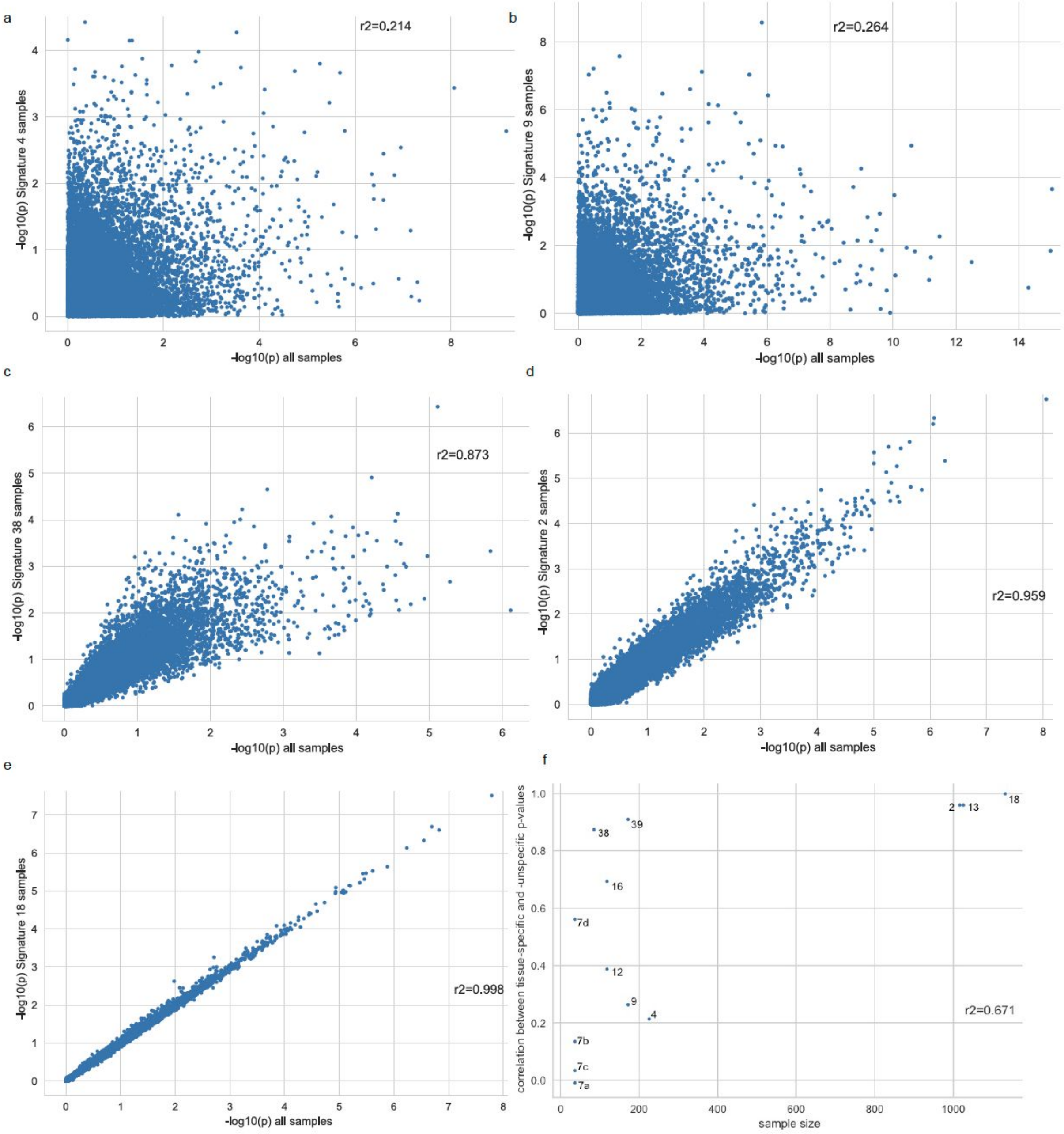

Extended Data Figure 24 | Comparison of the analysis of the whole cohort with cancer type-specific analyses. a-e, The p-values ($-\log_{10}P$) of cancer type-specific analyses are compared against the p-values of the analysis applied to the whole cohort and the Pearson correlation coefficient (r^2) is calculated. Per signature, all cancer types are taken into account that show presence of the specific signature (see Figure S16). The presented signatures are **a-c,** cancer type-specific signatures that occur in up to 4 cancer types and **d-e,** common signatures that are not present in up to 5 cancer types. **f,** Correlations between cancer type-specific and whole-cohort p-values (r^2) are plotted over the sample size of the respective cancer types.

Supplementary Notes

Analysis of gene expression confounders

In this study we used PEER to adjust for hidden confounders to calibrate our statistical models and avoid false associations. This approach has been used previously across multiple studies and for deeper insights between the correlation of technical variation and covariates please refer to (Lappalainen et al. 2013; GTEx Consortium et al. 2017). These studies showed that using PEER factor can increase the detection of eQTLs. However there is a risk that broad biological effects may be removed as well. Given that we mostly focus on local associations, this is an acceptable side effect here.

To analyse the effect of PEER in our cohort we applied ordinary least-square regression to correlate each of the 35 PEER factors with *per* sample covariates, including cancer project codes (reflecting cancer histotypes), gender, tumor purity, somatic burden and several sequencing metrics, to understand the proportion of variance explained by known biological and technical covariates. Sequencing metrics include library depth and number of unmapped reads as well as sample degradation levels (3'/5' bias). Adjusted R^2 was used to show the proportion of variance explained by each known covariate (**Figure 1**). This analysis showed that most of known covariates tested did not correlate with PEER factors (on average $R^2 = 0.01$ per covariate across all factors), with the exception of few cancer histotypes showing strong correlation with the first top PEER factors (e.g. CLLE-ES and factors 2, with $R^2 = 0.59$).

Analysis of co-localization of structural variants and somatic burden linked to eGenes

The analysis of co-localization of structural variants (SVs) and somatic burden was performed *per* aliquot id, looking for the closest SV to the leading genomic interval identified for each eGene, using the consensus WGS-based somatic structural variants (version 1.6; https://dcc.icgc.org/releases/PCAWG). The analysis identified 110 (17%) eGenes (**Supplementary Table 5**) with at least one SV close to the individual mutational burden (with a maximum distance observed between the burden and the SV of 40kb). Among the eGenes with SVs, we found immunoglobulin (Ig) genes to be the most prevalent class of eGenes with structural alterations (85/110 eGenes).

Genes significantly altered through splicing

We sought to identify genes under positive selection for somatic mutations associated with splicing alterations. For multi-exon genes, we reasoned that selection at the gene-level could be achieved through splicing alteration at different exons. Therefore, we decided to measure positive selection by considering all exons within a gene rather than an individual exon. To do this, we used a permutation based approach to compare the gene-level splicing alterations against background levels (**Figure 2a**). We utilized splicing quantification data from 1359 RNA-seq datasets from primary tumor and matched normal tissue for samples with available data. From our splicing quantification, we computed a percent spliced in (PSI) derived z-score for each exon across all multi-exon genes for each patient. Next, we filtered for exons with proximal

somatic mutations (50 base pairs (bp) into the intron, 5 bp into the exon), which represent cases in that are likely to to have altered splicing. We then assigned an overall gene impact score by computing the average z-score of all exons with proximal mutations (**Figure 2b**). As a negative control, we used the same method considering mutations distal to exonic regions, which are not likely to have a strong effect on splicing (a 55 bp window from 295 to 250 bp into introns) (**Figure 2b**). Our results revealed two genes under strong positive selection, *TP53* and *FANCA* (pval < 0.01). Both of these genes are known tumor suppressor genes, and genes of the cancer gene census.

GTEx Consortium, Laboratory, Data Analysis &Coordinating Center (LDACC)—Analysis Working Group, Statistical Methods groups—Analysis Working Group, Enhancing GTEx (eGTEx) groups, NIH Common Fund, NIH/NCI, NIH/NHGRI, et al. 2017. “Genetic Effects on Gene Expression across Human Tissues.” *Nature* 550 (7675): 204–13.

Lappalainen, Tuuli, Michael Sammeth, Marc R. Friedländer, Peter A. C. ’t Hoen, Jean Monlong, Manuel A. Rivas, Mar Gonzàlez-Porta, et al. 2013. “Transcriptome and Genome Sequencing Uncovers Functional Variation in Humans.” *Nature* 501 (7468): 506–11.

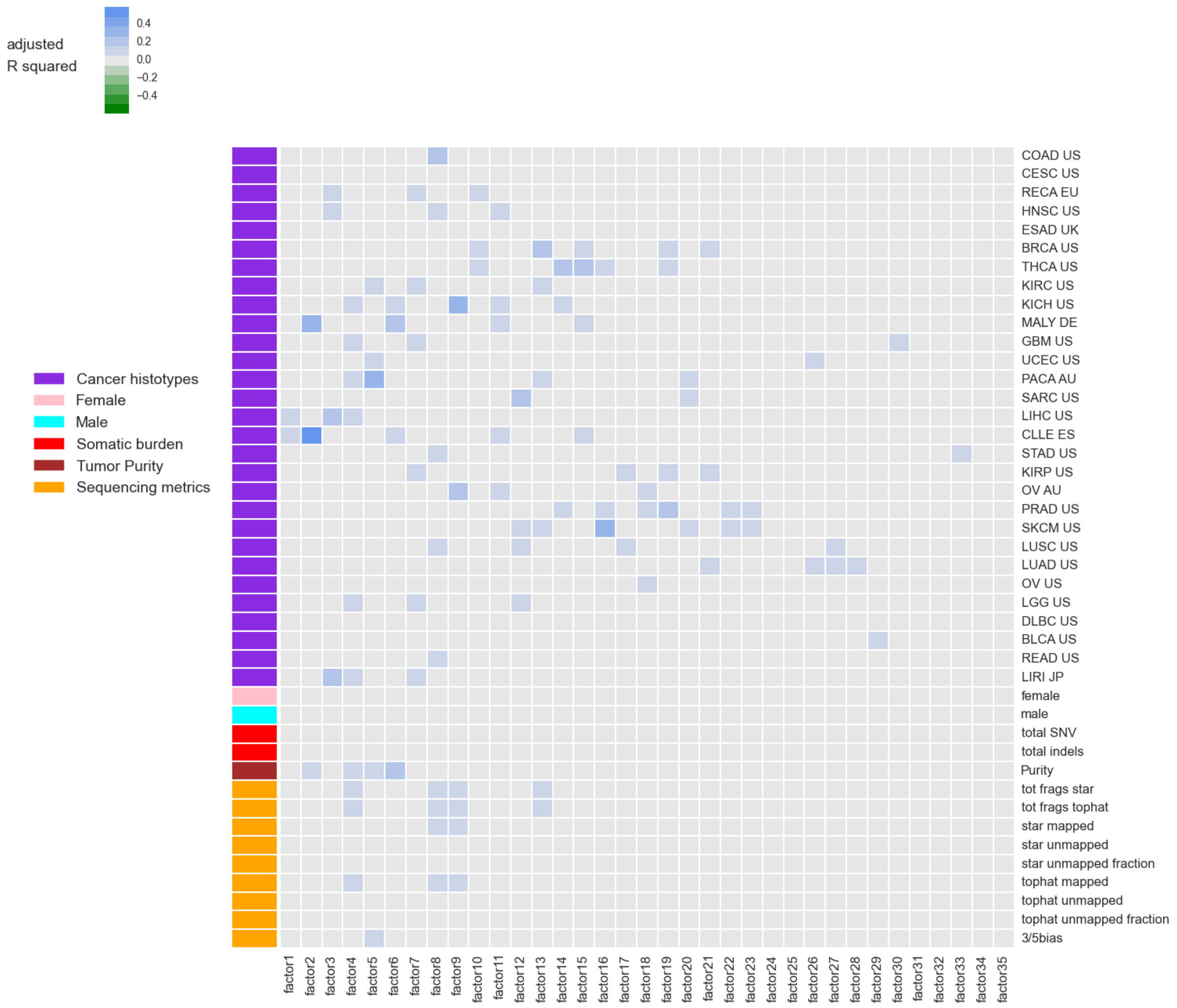

Figure 1: Heatmap showing the proportion of variance (measured as adjusted R^2) of PEER gene expression residuals explained by known *per* sample covariates.

SF1a

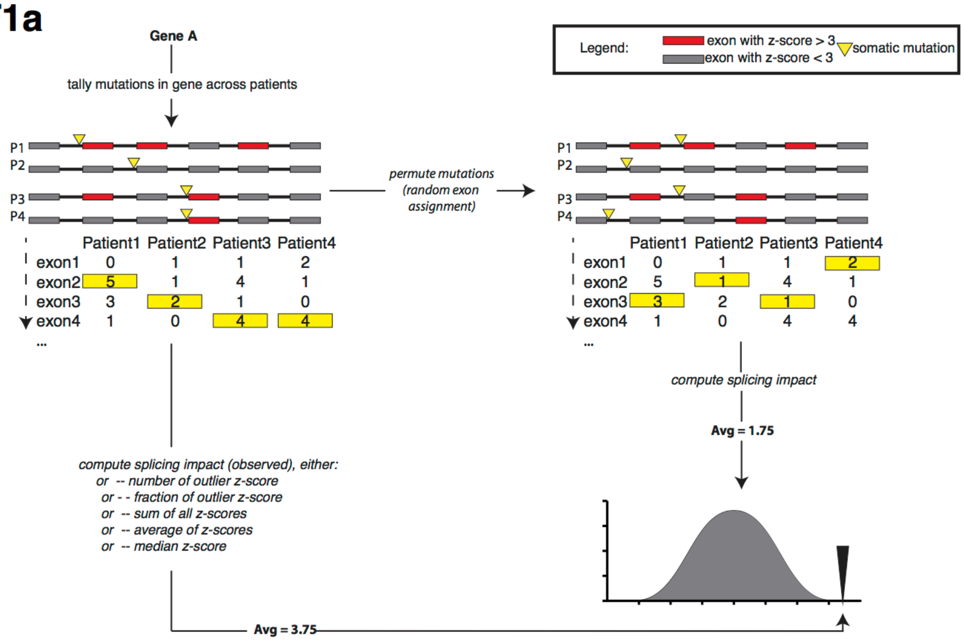

SF1b

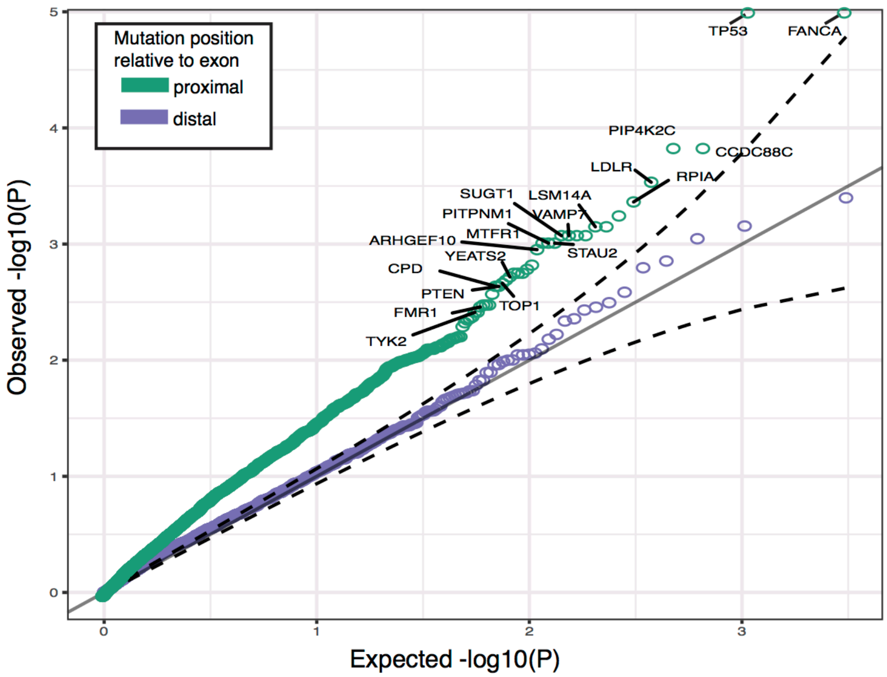

Figure 2: **Genes significantly altered through splicing.** (a) Permutation approach to identify genes with mutations associated outlier splicing more than would be expected by chance. (b) QQ-plot comparing approach using mutations proximal to the exons (50 base pairs (bp) into the intron, 5 bp into the exon), or distal to the exons (a 55bp window farther into the intron).
