# Supplementary material for: Genomic basis for RNA alterations in cancer
Source: Nature. 2020 Feb 5;578(7793):129–36. doi: 10.1038/s41586-020-1970-0 (PMC7054216; doi:10.1038/s41586-020-1970-0)
Supplement: Supplementary file 2 — Reporting Summary [file 41586_2020_1970_MOESM2_ESM.pdf]

nature research

Corresponding author(s): Alvis Brazma, Gunnar Raetsch, Angela N Brooks

Reporting Summary

Nature Research wishes to improve the reproducibility of the work that we publish. This form provides structure for consistency and transparency in reporting. For further information on Nature Research policies, see Authors & Referees and the Editorial Policy Checklist.

Statistical parameters

When statistical analyses are reported, confirm that the following items are present in the relevant location (e.g. figure legend, table legend, main text, or Methods section).

n/a	Confirmed	
☐	☒	The exact sample size (*n*) for each experimental group/condition, given as a discrete number and unit of measurement
☐	☒	An indication of whether measurements were taken from distinct samples or whether the same sample was measured repeatedly
☐	☒	The statistical test(s) used AND whether they are one- or two-sided<br>*Only common tests should be described solely by name; describe more complex techniques in the Methods section.*
☐	☒	A description of all covariates tested
☐	☒	A description of any assumptions or corrections, such as tests of normality and adjustment for multiple comparisons
☐	☒	A full description of the statistics including central tendency (e.g. means) or other basic estimates (e.g. regression coefficient) AND variation (e.g. standard deviation) or associated estimates of uncertainty (e.g. confidence intervals)
☐	☒	For null hypothesis testing, the test statistic (e.g. *F*, *t*, *r*) with confidence intervals, effect sizes, degrees of freedom and *P* value noted<br>*Give P values as exact values whenever suitable.*
☐	☒	For Bayesian analysis, information on the choice of priors and Markov chain Monte Carlo settings
☒	☐	For hierarchical and complex designs, identification of the appropriate level for tests and full reporting of outcomes
☐	☒	Estimates of effect sizes (e.g. Cohen's *d*, Pearson's *r*), indicating how they were calculated
☐	☒	Clearly defined error bars<br>*State explicitly what error bars represent (e.g. SD, SE, CI)*

Our web collection on statistics for biologists may be useful.

Software and code

Policy information about availability of computer code

Data collection	Core data was collected through Pan-cancer Analysis of Whole Genomes Data Coordination center through https://dcc.icgc.org/releases/PCAWG/
Data analysis	Core RNA-Seq alignment pipelines are available through Github/Docker: https://github.com/akahles/icgc_rnaseq_align, https://hub.docker.com/r/nunofonseca/irap_pcawg/. STAR, TopHat2, HTSeq, Kallisto, Limix, PLINK, Bedtools, Vcftools, Bcftools, Samtools, Tabix, GATK, ASEReadCounter, Lavaan, Mediate, SplAdder, SAVNet, Sv2gf

For manuscripts utilizing custom algorithms or software that are central to the research but not yet described in published literature, software must be made available to editors/reviewers upon request. We strongly encourage code deposition in a community repository (e.g. GitHub). See the Nature Research guidelines for submitting code & software for further information.

Data

March 2018

Policy information about availability of data

All manuscripts must include a data availability statement. This statement should provide the following information, where applicable:

- Accession codes, unique identifiers, or web links for publicly available datasets
- A list of figures that have associated raw data
- A description of any restrictions on data availability

Somatic and germline variant calls, mutational signatures, subclonal reconstructions, transcript abundance, splice calls and other core data generated by the ICGC/TCGA Pan-cancer Analysis of Whole Genomes Consortium is described here58 and available for download at https://dcc.icgc.org/releases/PCAWG. Additional information on accessing the data, including raw read files, can be found at https://docs.icgc.org/pcawg/data/. In accordance with the data access policies of the ICGC and TCGA projects, most molecular, clinical and specimen data are in an open tier which does not require access approval. To access potentially identification information, such as germline alleles and underlying sequencing data, researchers will need to apply to the TCGA Data Access Committee (DAC) via dbGaP (https://dbgap.ncbi.nlm.nih.gov/aa/wga.cgi?page=login) for access to the TCGA portion of the dataset, and to the ICGC Data Access Compliance Office (DACO; http://icgc.org/daco) for the ICGC portion. In addition, to access somatic single nucleotide variants derived from TCGA donors, researchers will also need to obtain dbGaP authorisation. Data derived specifically from RNA-Seq analysis can be found at https://dcc.icgc.org/releases/PCAWG/transcriptome. Subfolders contain identification and quantification of alternative promoter usage, alternative splicing, RNA fusions, gene expression, transcript-level expression, and RNA editing. Identified eQTLs are in https://dcc.icgc.org/releases/PCAWG/transcriptome/eQTL and a binarized table indicating all RNA and DNA alterations for each gene can be found in the subfolder https://dcc.icgc.org/releases/PCAWG/transcriptome/recurrence_analyses/. Additionally, QC metrics and metadata are also included.

Field-specific reporting

Please select the best fit for your research. If you are not sure, read the appropriate sections before making your selection.

☒ Life sciences ☐ Behavioural & social sciences

For a reference copy of the document with all sections, see nature.com/authors/policies/ReportingSummary-flat.pdf

Life sciences

Study design

All studies must disclose on these points even when the disclosure is negative.

Sample size	This study represents the analysis of 1,188 donors from the Pan-Cancer Analysis of Whole Genomes project. The sample size was limited to the availability of RNA-Seq data from matched donors with WGS data from the PCAWG project.
Data exclusions	A larger set of 2,217 RNA-Seq libraries were initially collected and data were excluded after QC analysis. The QC criteria was standard and pre-established before excluding data.
Replication	Reproducibility of the analysis is ensured through data-sharing and code-sharing. Unfortunately, at the time of the analysis there were no approrpriate datasets to use for replication studies of associations, since this is one of the largest collections of pan-cancer whole genomes and matched transcriptomes. For the somatic eQTL analysis, there were some related studies that came to similar conclusions and are noted in the Supplementary Information.
Randomization	Cancer histotypes were defined by the PCAWG Pathology and Clinical Correlates Working Group based on tumor histology. These tumor subtypes were accounted for as covariates in all applicable analyses of association.
Blinding	Blinding was not relevant to our study as it was essential to understand underlying confounding variables in our associations, such as tumor subtype, sex, etc.

Materials & experimental systems

Policy information about availability of materials

n/a	Involved in the study
☒	☐ Unique materials
☒	☐ Antibodies
☒	☐ Eukaryotic cell lines
☒	☐ Research animals
☒	☐ Human research participants

Method-specific reporting

n/a	Involved in the study
☒	☐ ChIP-seq
☒	☐ Flow cytometry
☒	☐ Magnetic resonance imaging
