# Supplementary material for: Genomic basis for RNA alterations in cancer
Source: Nature. 2020 Feb 5;578(7793):129–36. doi: 10.1038/s41586-020-1970-0 (PMC7054216; doi:10.1038/s41586-020-1970-0)
Supplement: Supplementary file 3 — This zipped file contains Supplementary Tables 1-24 and a Supplementary Table Guide. [file 41586_2020_1970_MOESM3_ESM.zip › Supplementary_Tables/Supplementary Table Guide.pdf]

Supplementary Table Guide

Supplementary Table 1. Matrix of covariates used across different analysis

Supplementary Table 2. Catalogue of germline eQTL
Number of eQTLs across all cancer histotypes as well as annotation of all germline eQTL lead variants including effect sizes, p-values and genomic positions for pan-cancer analysis as well as subset of cancer-specific germline eQTL in each of seven cancer types with at least 60 patients.

Supplementary Table 3. Summary of germline eQTL specific to the tumor cohort in comparison to GTEx
Summary of all ICGC cohort specific eQTL contrasted to GTEx. The table includes gene name, nominal p-value in the ICGC cohort, smallest nominal p-value in GTEx cohort across all tissues and in matching tissues only, as well as \`best\` (see Methods) nominal p-value in every single GTEx tissue.

Supplementary Table 4. Gemline eQTL enrichment of cancer pathways.

Supplementary Table 5. Somatic eGenes
ENSEMBL and HGCN names of the 649 eGenes identified in the somatic eQTL analysis (FDR ≤ 5%). The table includes chromosome, genomic interval middle position, coordinates and genomic context (flanking, intronic or exonic) of the leading associated mutated genomic region, the lead variant nominal, the distance of the middle interval position to eGene TSS and adjusted p-values and effect size. Leading interval burden frequency across all patients ('Total_burden_prevalence') and mean frequency across the 27 cancer types are also reported. The 'SV counts per eGene' spreadsheet shows the number of aliquot ids with somatic burden per eGene, number of aliquots with an SV close to burden and the corresponding percentage. The 'GeneHancer overlap' spreadsheet shows the 33 somatic eQTL overlapping GeneHancer "double-elite" elements, while 'Pan-can studies overlap' shows the overlaps with associations identified in previous cancer studies.

Supplementary Table 6. Roadmap Epigenomics enrichment
Enrichment in Roadmap Epigenomics marks over 127 cell lines. The table contains the abbreviated epigenetic mark, the cell line, the number of significant flanking leading genomic regions of the somatic eQTL analysis that overlap the respective mark in the respective cell line, the average number of matched flanking regions that overlap the mark in 1k random samplings, the fold enrichment, nominal and adjusted p-values.

Supplementary Table 7. TFBS enrichment
Enrichment in ENCODE transcription factor binding sites (TFBS) over 9 selected cell lines. The table contains the transcription factor bound by the site, the cell line, the number of significant flanking leading genomic regions of the somatic eQTL analysis that overlap the respective

TFBS in the respective cell line, the average number of matched flanking regions that overlap the site in the 1k random samplings, the fold enrichment, empirical and adjusted p-values.

Supplementary Table 8. GO enrichment in somatic eGenes
The table contains the top 15 GO categories with q-value ≤ 10%, the category description, the nominal p-value and the number of eGenes found per category ('Count').

Supplementary Table 9. Aggregate results of the linear model predicting ASE
Aggregate table describing average predictors, outcome and fitted terms of the multivariate generalised linear model for prediction of AEI. Effect sizes begin with 'term', input somatic burden categories begin with 'type', predicted effects begin with 'pred'. The different haplotypes are referred to with 'ht1' and 'ht2'. Germline effects are included via a binary indicator of heterozygosity of the lead germline eQTL variant ('ishet'). Counts refer to RNA-seq read counts over the respective haplotypes. Cancer-testis / testis-specific genes are marked via a binary indicator 'istsg'.

Supplementary Table 10. List of exon skipping events with somatic mutations adjacent to exon-intron boundaries
Catalogue of somatic mutations adjacent to exon-intron boundaries with measured impact on exon skipping. Somatic mutations within a 20 basepair window of the exon-intron boundary (-4 to 14) are included in this table. Each mutation is labeled with its corresponding donor, aliquot and histology ID. For exon skipping events, the associated exon positions (SplAdder event), Ensembl ID, and strand information is included. Percent spliced in (PSI) values for each exon skipping event are included per sample, as well as the average PSI for a samples histology cohort. Genomic 0-based start coordinate for the corresponding somatic mutation are labeled as "mut_pos1", and relative position to nearest splice site (closest_feature) are also included.

Supplementary Table 11. List of exon skipping events with somatic mutations adjacent to identified branchpoints.
Catalogue of somatic mutations adjacent to branchpoints with measured impact on exon skipping. Somatic mutations within a 20 basepair window of branchpoints (-10 to 10) are included in this table. Each mutation is labeled with its corresponding donor, aliquot and histology ID. For exon skipping events, the associated exon positions (SplAdder event), Ensembl ID, and strand information is included. Percent spliced in (PSI) values for each exon skipping event are included per sample, as well as the average PSI for a sample's histology cohort. Genomic 0-based start coordinate for the corresponding somatic mutation are labeled as "mut_pos1", and relative position to nearest splice site (closest_feature) are also included. Last, 0-based coordinates for the branchpoint window sequence is included as "bp_coords."

Supplementary Table 12. Gene-level splicing recurrence analysis
This table contains results from the gene-level splicing recurrence analysis used to find genes under positive selection for splicing alterations. Genes are labeled by hugo and ensembl ID. Observed values represent the average z-score for exons with mutations within a 55 nucleotide window around splice sites (from the -5 exonic position up to 50 nucleotides into the intron).

Total mutants describe how many samples were used in the significance test, and the number of exons represents the total number of exons with available splicing quantification. Last, the tests represent the number of permutations for each gene.

Supplementary Table 13. List of novel exonization candidate events in proximity to somatic alterations
The table contains all novel exonization events, that is cassette exons exclusively detected in the PCAWG tumor samples, together with information on somatic variants nearby. For each event, we provide a unique ID, its genomic coordinates, the ENSEMBL ID and common name of the annotated gene, the maximal absolute difference between min and max PSI over all samples as well as the coding state of the gene. If there was a somatic variant within a +/- 25bp window around the novel exon, we added its position and state NA, otherwise.

Supplementary Table 14. List of splicing associated variants near exonization events.
The table contains a list of mutations per PCAWG tumor sample that were found nearby (+/- 25bp) novel exonization events. For each mutation, we provide the mutation coordinate, chromosome, exonization coordinates, ensembl gene ID, hugo gene ID, sample donor ID, and histology type.

Supplementary Table 15. Bridged fusions identified
For each bridged fusion identified the following information is provided: the aliquot id of the RNA-seq sample; histology; whole-genome-sequencing id; frameshift; and information about the breakpoints at RNA and DNA level.

Supplementary Table 16. Complete RNA and DNA outlier alteration table.
This table contains all identified RNA and DNA outlier alterations in each gene-sample pair. For each gene-sample pair, it includes an indicator of 1 if an alteration is present, otherwise 0; ICGC donor id; histology type; ENSEMBL ID; and HGNC symbol. Each alteration type is distinguished by column: expression outliers are indicated as “expr_outlier”; splice outliers are indicated as “isSplice”; alternative promoter outliers are indicated as “alt_prom”; fusions are indicated as “fusion”; copy-number outliers are indicated as “cn”; non-synonymous variants are indicated as “variants”; allele specific expression outliers are indicated as “ase_all”; and rna editing events are indicated as “rna_edit”.

Supplementary Table 17. Comparison of alteration incidences for different cancer types
This table contains the median alteration incidences and the significance of alteration incidence differences for each two cancer types. The comparisons were based on Wilcoxon Rank Sum Test.

Supplementary Table 18. Co-occurred alteration pairs in pan-cancer level
This table contains significant co-occurred alteration pairs in pan-cancer level ($P < 0.05$) with significant co-occurrence within at least one cancer type. “Gene.1” indicates COSMIC genes and “alt.1” indicates the alteration type of “gene.1”. “Gene.2” indicates all genes and “alt.2” indicates the alteration type of “gene.2”. The “n_tissue” column indicates the number of cancer

types within which the pairs are significantly co-occurred. “P-values” indicates p-values of pan-cancer level co-occurrence and “q-values” indicates q-values based on Benjamini-Hochberg correction.

Supplementary Table 19. Results of mutational signatures analyses
Significant gene expression-mutational signature association (FDR ≤ 10%). A) Association study across all patients. B) Association study across the carcinoma patients. C) Association study across the European patients. D) GO and Reactome Pathways enrichment analyses (FDR correction across categories, FDR ≤ 10%) of signature-associated genes for signatures that significantly affect more than 20 genes at FDR ≤ 10%. E) Number of significantly associated genes (FDR ≤ 10%), known aetiology and significantly associated germline variant (FDR ≤ 10%), if available, of each mutational signature. In the case of an associated germline variant, the germline rs ID, the respective associated gene (Ensembl ID) and the p-values for the null hypothesis of colocalisation are listed. F) Correlations between p-values (-log10P) of gene expression associations of the total number of SNVs and the respective signature. The absolute Pearson correlation coefficient is always smaller than 0.1.

Supplementary Table 20. Genes with heterogeneous mechanisms of alteration
Table indicates genes that are in the following categories: a Cancer Gene Census Gene, a cancer pathway gene [119], is a PCAWG driver gene based on nonsynonymous mutations, is found to be recurrently altered at the DNA and RNA level, has an associated eQTL, has a somatic contribution to allele specific expression, is part of an RNA fusion with DNA-level structural variant support, has a splicing alteration associated with a mutation at splice sites or branchpoints, and the total number of RNA alterations associated with a cis-acting mutation

Supplementary Table 21. Recurrently altered genes at the DNA and RNA level
This table has the frequency and recurrence score calculated over the entire cohort for all genes considered in the analysis. The genes are sorted by recurrence score, where the smallest score indicates the highest recurrence. The recurrence score is indicated by column labeled “rank”. Each of the columns indicated by alteration type is the number of samples with the indicated alteration for each gene.

Supplementary Table 22. Recurrent outliers identified in our analysis and GTEx
This table contains the genes we found to have a significantly recurrent expression outlier in GTEx and our complete recurrence analysis. The table contains the frequency at which the outlier was observed, as well as its rank within each alteration type.

Supplementary Table 23. Cancer subtype abbreviations. Full names of cancer subtype abbreviations.

Supplementary Table 24. Base-Pair substitution frequencies of observed RNA-editing events This table contains the frequencies for each possible base-pair substitution observed from RNA-Editing. The frequencies are reported after two steps of filtering: first filtering for exonic edits only (column 2); secondly for non-synonymous and exonic edits (column 3).
